# Quantitative *In Silico* Prediction of the Rate of Protodeboronation by a Mechanistic Density Functional Theory-Aided Algorithm

**DOI:** 10.1021/acs.jpca.2c08250

**Published:** 2023-03-14

**Authors:** Daniel S. Wigh, Matthieu Tissot, Patrick Pasau, Jonathan M. Goodman, Alexei A. Lapkin

**Affiliations:** † Department of Chemical Engineering and Biotechnology, University of Cambridge, CB3 0AS Cambridge, U.K.; ‡ UCB Biopharma SPRL, 1420 Braine l’Alleud, Belgium; ¶ Yusuf Hamied Department of Chemistry, University of Cambridge, CB2 1EW Cambridge, U.K.

## Abstract

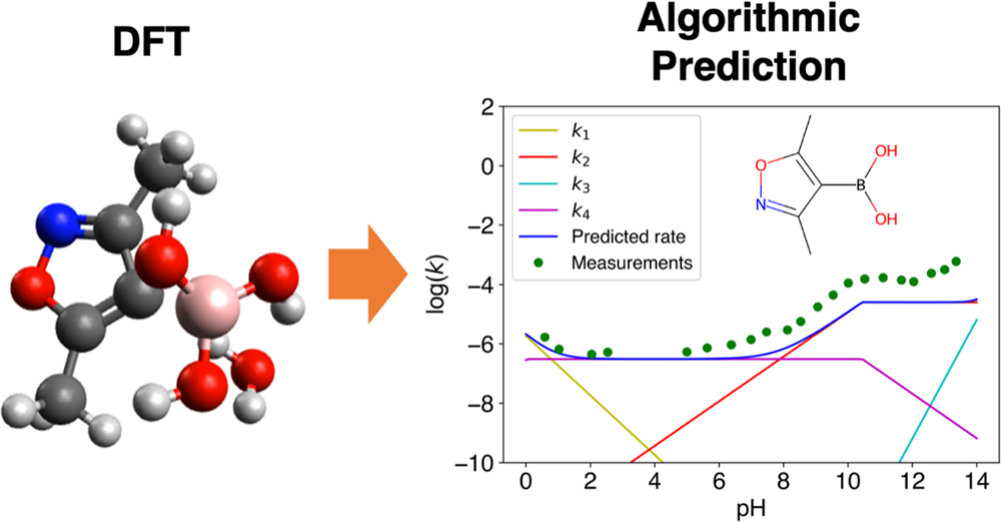

Computational reaction prediction has become a ubiquitous task in chemistry due to the potential value accurate predictions can bring to chemists. Boronic acids are widely used in industry; however, understanding how to avoid the protodeboronation side reaction remains a challenge. We have developed an algorithm for *in silico* prediction of the rate of protodeboronation of boronic acids. A general mechanistic model devised through kinetic studies of protodeboronation was found in the literature and forms the foundation on which the algorithm presented in this work is built. Protodeboronation proceeds through 7 distinct pathways, though for any particular boronic acid, only a subset of mechanistic pathways are active. The rate of each active mechanistic pathway is linearly correlated with its characteristic energy difference, which in turn can be determined using Density Functional Theory. We validated the algorithm using leave-one-out cross-validation on a data set of 50 boronic acids and made a further 50 rate predictions on academically and industrially important boronic acids out of sample. We believe this work will provide great assistance to chemists performing reactions that feature boronic acids, such as Suzuki–Miyaura and Chan–Evans–Lam couplings.

## Introduction

Boronic acids (BAs) have long been of interest to the chemical sciences community^1^ due to their irreplaceable role in communications,^2^ materials,^3−7^ and medicine.^8^ BAs also feature as the nucleophilic coupling partner in a number of key organic reactions including the Suzuki–Miyaura reaction,^9,10^ Chan–Evans–Lam coupling,^11−13^ Liebeskind–Srogl coupling,^14^ and oxidative Heck^15^ and can also undergo addition with carbonyls, imines,^16,17^ and enones.^18,19^ A reaction will not work if one of the coupling partners degrades at a significantly faster rate than the rate of the intended reaction, and protodeboronation is one of the most significant, if not the most significant, degradation pathways/side reactions in the aforementioned named organic reactions. Being able to accurately predict the rate of protodeboronation would therefore be hugely beneficial to anyone working with BAs, as it can help with reaction planning.

Computational approaches to reaction planning and reaction prediction are becoming increasingly sophisticated, perhaps most notably at the intersection of machine learning and synthetic organic chemistry. However, there remains a rift between approaches in the big data regime, typically being more general but less accurate, and small data regime, with the opposite characteristics, though both approaches rely on effective molecular representation.^20^ Machine learning architectures involving deep neural networks can work effectively with large data sets,^21,22^ while small data approaches may use a machine learning approach such as multitask learning,^23^ an understanding of the kinetics,^24,25^ and/or Density Functional Theory (DFT) calculations.^26^ However, recent work suggests that more detailed descriptors do not necessarily lead to better predictions.^27^ With just 469 data points, the algorithm presented in this work for protodeboronation prediction fits into the small-data regime, and it utilizes a mechanistic understanding of protodeboronation in combination with DFT calculations.

Kinetic studies are typically conducted to achieve total process understanding, which enables rapid identification of optimal reaction conditions for complex chemical systems.^28^ The complexity of such systems may be simplified by “lumping” reaction paths (and therefore kinetic constants) together to identify observed reaction rates.^29^ Kinetic studies typically involve running experiments at lab scale to determine the order of reaction with respect to each reactant, and estimate the kinetic constants given experimental data collected in a carefully designed campaign. Kinetic parameter prediction (*in silico*) is less common than kinetic parameter estimation (given experimental data) since the prediction is inaccurate without a good understanding of the reaction system. In the case of protodeboronation, previously published work^30−32^ allows the prediction of what the reaction pathways for a novel boronic acid will look like, which thereby provides context to the kinetic parameter prediction. This is not the case for many other systems, where kinetic data does not exist or where changing the reactants will lead to unpredictable changes in the reaction mechanism. To the best of our knowledge, this is the first purpose-built algorithm to be published in the literature capable of making *in silico* predictions of the rate of protodeboronation.

**Scheme 1:**
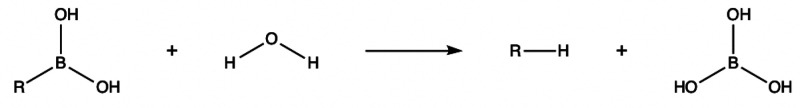
General Scheme for Protodeboronation in Aqueous Solution

In the protodeboronation reaction the carbon–boron bond of a BA is broken and replaced with a carbon–hydrogen bond, as in Scheme 1. It is an irreversible reaction and will compete with the intended reaction. Protodeboronation is the most significant side reaction in Suzuki–Miyaura reactions, and since Suzuki–Miyaura reactions are often carried out in aqueous solution, understanding protodeboronation in aqueous solution is of significant interest. There are several different mechanistic pathways for protodeboronation in aqueous solution, most, if not all, of which have been outlined in previous literature, and a general mechanistic model was built to describe these simultaneous mechanistic pathways as a function of pH.^30−32^

The general scheme for the Suzuki–Miyaura reaction in the presence of excess water is

A + B → C Intended reaction
A → D Protodeboronation side reaction

In the special case where the self-catalytic protodeboronation mechanistic pathway is significant, the general scheme becomes

A + B → C Intended reaction
A + A → A + D Self-catalytic protodeboronation side reaction
A → D Protodeboronation side reaction

This is quite similar to the intensely studied Van de Vusse kinetic scheme:^33,34^

A → B → C
A + A → D

Van de Vusse kinetics explores how changes in kinetic constants affect the formation of a competing product and so change the yield of the desired product. This is a simplification of experimental chemistry, as Van de Vusse kinetics only looks at cases where there are competing reactions and low yield is due to the production of a different product rather than because the starting material is unreacted (which may be caused by slowness of the reaction, the degradation of the reagents in the reaction vessel, or other issues). The Van de Vusse kinetic scheme can nonetheless be adapted to the specific kinetic scheme of the Suzuki reaction to explore the intricate relationship between choice of BA, reaction conditions, protodeboronation rate, and yield. While Van de Vusse kinetics deals with both sequential and parallel reaction pathways, the Suzuki coupling/protodeboronation system exclusively features parallel pathways. There may be one or many mechanistic pathways for protodeboronation happening simultaneously, and in this work this is dealt with by calculating the rate of each mechanistic pathway separately, before then adding up all contributions to arrive at the overall rate which can be observed in the lab. A linear model was set up for each mechanistic pathway, fitting the maximum observed rate of reaction against the results of quantum chemistry calculations carried out using DFT. DFT is a widely used method for calculating energies associated with chemical reactions^35^ and generating relevant descriptors,^36^ and it is also used beyond computational chemistry.

In this work, an algorithm combining the general mechanistic model for protodeboronation with DFT calculations fitted to experimental data was developed to predict the rate of aqueous protodeboronation as a function of pH for unseen BAs without the need for further wet lab experiments.

## Methodology

An algorithm is a sequence of steps to be performed in pursuit of some goal. In this work the goal is to predict the rate of protodeboronation for novel boronic acids using a small amount of experimental data. The data used is described below, with each algorithmic step then being presented in chronological order. An overview of how the quantum chemistry calculations were performed can be found at the end of the methodology section, and a full description can be found in the Supporting Information.

### Data

The data set used in this work comes from the Ph.D. thesis of Cox,^30^ where he collected 484 data-points across 51 different BAs measuring rate of protodeboronation as a function of pH. An overview of the types of molecules considered in this study is visible in Figure 2, with the full list of molecules available in the Supporting Information. Most of the molecules were 5- or 6-membered ring aromatic BAs. In this work we used data on 50 of the 51 BAs, totalling 469 data points, excluding information only of the tosylate BA due to its bulky size which would complicate DFT calculations. The experiments were carried out in a 50/50 water/dioxane mixture between pH 0 and 14. The rate of reaction observed in the experiments, log_10_(*k*_*obs*_), ranged from −9 (approximately a 22 year half-life) to +2 (approximately a 7 ms half-life). The half-life (in seconds) of a boronic acid can be calculated given the rate using the following formula: *t*_1/2_ = ln(2)/*k* (valid for first-order reactions).

### General Mechanistic Model for Protodeboronation

**Figure 1:**
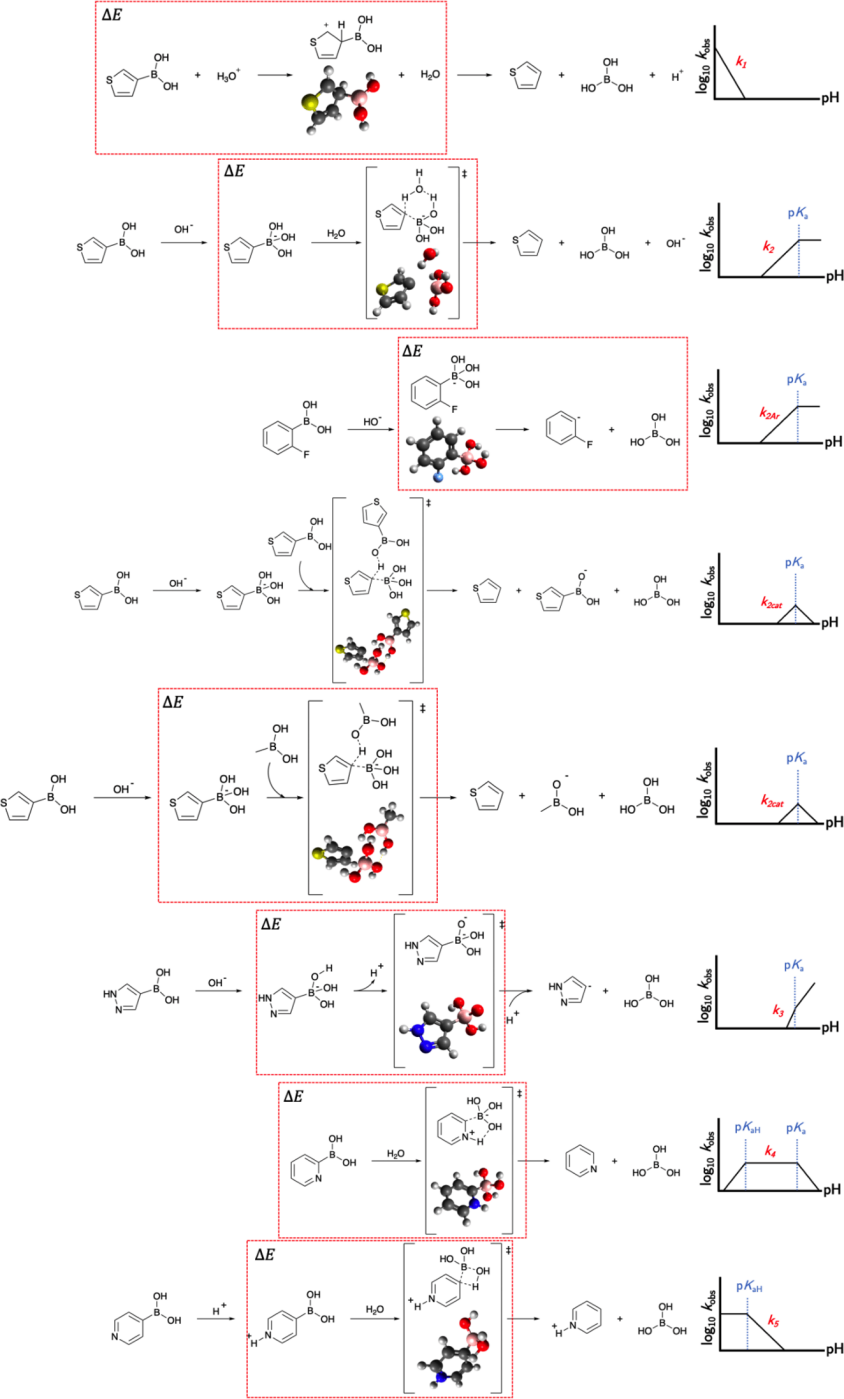
Example of each of the mechanistic pathways of protodeboronation relevant to the boronic acids considered in this work.^30^ A red box encloses the two stages used for calculation of Δ*E* for each mechanism; *k*_*2cat*_ in the fourth row has no red box, since Δ*E* was calculated using a simplified version of this mechanism (shown in the fifth row). A detailed description of each mechanistic pathway can be found in the Supporting Information.

A general kinetic model for aqueous protodeboronation as a function of pH was developed by Cox et al.^30^ and can be seen in Figure 1. The general model arose from a combination of chemical insight and experimental data and was used to estimate the kinetic parameters of each mechanism. Traditional kinetics is an important tool to build an understanding of the underlying chemistry. However, it is not possible to extrapolate a traditional kinetics model to novel substrates without additional wet lab experiments. While Cox et al. were the first to publish a general mechanistic model for protodeboronation which is valid for a range of boronic acids across the whole aqueous pH spectrum, research into protodeboronation has been going on for decades. The mechanisms they propose are backed by previous literature,^10,37−44^ and coupling this with their experimental validation yields great credibility to the veracity of their proposed mechanistic pathways.

In this work, log(*k*_*obs*_) refers to the overall protodeboronation rate of reaction, i.e., the observed/measured rate. Conversely, log(*k*_*n*_) (e.g., log(*k*_1_)) refers to the rate of reaction of a particular protodeboronation mechanism, e.g., mechanism 1, which cannot be measured directly. However, with knowledge of how the rate of each of the mechanisms changes with pH, the pH at which log(*k*_*n*_) ≈ log(*k*_*obs*_) can be deduced. As an example, for a molecule with active mechanisms *k*_2_ and *k*_4_, log(*k*_4_) ≈ log(*k*_*obs*_) at pH = p*K*_aH_ (since the rate of *k*_2_ is negligible at this pH), and log(*k*_2_) ≈ log(*k*_*obs*_) at pH = 14 (since the rate of *k*_4_ is negligible at this pH).

Each mechanism depends on a number of factors which in turn can be used to understand the shape of the pH – log(*k*_*obs*_) curve. As an example, the *k*_4_ mechanism only occurs in the zwitterionic state; hence, only BAs with a basic site capable of abstracting a proton can decompose through the *k*_4_ mechanism. The *k*_4_ mechanism is at its maximum rate when all of the substrate is in the zwitterionic form, i.e., when pH is between p*K*_a_ and p*K*_aH_. The rate of the *k*_4_ mechanism linearly decreases as the pH moves either below p*K*_aH_ or above p*K*_a_, because the proportion of substrate in zwitterionic form decreases.

### Determining Active Mechanisms

The general mechanistic model for protodeboronation features 7 distinct mechanistic pathways: *k*_1_, *k*_2_, *k*_*2Ar*_, *k*_*2cat*_, *k*_3_, *k*_4_, and *k*_5_. *k*_*2Ar*_ refers to the mechanistic pathway for aryl boronic acids. *k*_*2cat*_ refers to the mechanistic pathway with a transition state featuring two reactant BAs coupled together during protodeboronation, so we believe autoprotodeboronation may be a more accurate labeling of this pathway than “autocatalysis” as Cox used, since the later typically refers to catalysis by a reaction product, but we have nevertheless decided to keep Cox’s *k*_*2cat*_ labeling.

**Figure 2:**
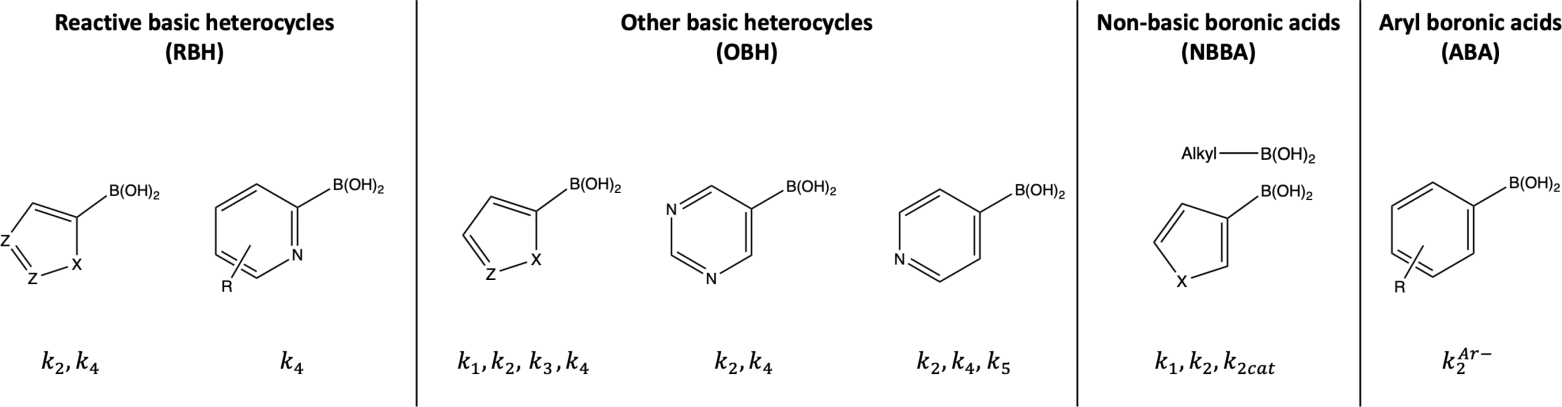
Heuristics for determining which mechanistic pathways are active for a particular boronic acid (Z = basic nitrogen, X = S, O).^30^

As previously described, not all 7 mechanistic pathways will be significant for any one BA, and a heuristic initially developed by Cox was used to determine which pathways are significant for each BA. These mechanism heuristics can be seen in Figure 2.

### Algorithmic Protodeboronation Prediction

**Scheme 2:**
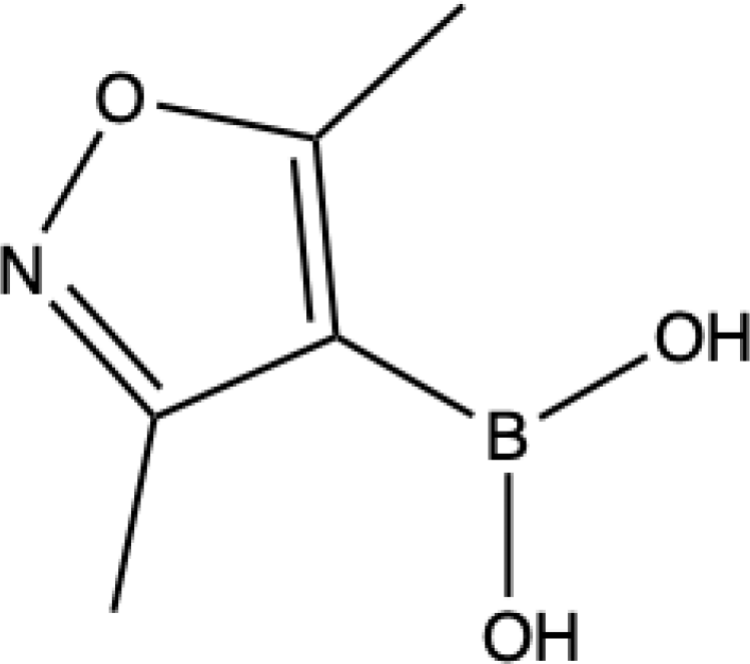
Structure of (3,5-Dimethyl-1,2-oxazol-4-yl)boronic Acid (DMOBA)

The journey of (3,5-dimethyl-1,2-oxazol-4-yl)boronic acid (DMOBA), shown in Scheme 2, through the algorithm will act as a case study to showcase how the model works.

#### 1 Choice of Boronic Acid

The chosen BA for which one would like to know the rate of protodeboronation in aqueous solution should fall within the scope of the model; i.e., the BA must be of type R–B–(OH)_2_, where the boron is directly bonded to a carbon atom. Predictive performance is better when the queried BA is similar to the BAs present in the data set, so the BA should ideally be small (fewer than 15 heavy atoms) and have the boron bonded to an aromatic carbon.

#### 2 Determine Active Mechanistic Pathways

Using the heuristics presented in Figure 2, the significant mechanisms for the queried molecule can be determined; from this it is apparent that DMOBA is of type “Other Basic Heterocycle” (OBH) with active mechanisms *k*_1_, *k*_2_, *k*_3_, and *k*_4_. A more detailed written description of the heuristics used to determine which category of boronic acid a molecule belongs to can be found in the Supporting Information.

#### 3 Calculate Δ*E* for Each Mechanistic Pathway

**Scheme 3:**
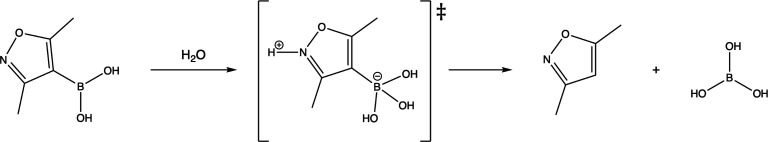
Mechanistic Pathway *k*_4_ for (3,5-Dimethyl-1,2-oxazol-4-yl)boronic Acid

An overview of the protodeboronation mechanisms can be seen in Figure 1, with the red box enclosing the structures used for the calculation of the characteristic energy difference (Δ*E*). For *k*_2_, *k*_*2cat*_, *k*_3_, *k*_4_, and *k*_5_, the characteristic energy difference is calculated as the difference in energy between the transition state and the reactant or preceding reaction intermediate, as per the Arrhenius equation. For *k*_1_ it is the difference in energy between the reaction intermediate and the reactant, while for *k*_*2Ar*_ it is between two reaction intermediates. As an example, the molecular structures used for the Δ*E* calculations for the *k*_4_ mechanistic pathway for DMOBA can be seen in Scheme 3.

Naturally, the DFT energy calculations of the transition state was a transition state optimization, while calculations for reactants and reaction intermediates were a local energy minimum optimization. Further details for each mechanism can be found in the Quantum Chemistry Calculations section and in the Supporting Information.

#### 4 Linear Regression of Δ*E* vs log(*k*_*n*_)

Each of the 7 mechanisms has a particular pH (or pH range), where the rate of reaction is at its maximum. For DMOBA the *k*_1_, *k*_2_, *k*_3_, and *k*_4_ mechanisms are active, and for these four mechanisms the maximum rate of reaction can be found at pH = 0, pH > p*K*_a_, pH = 14, and p*K*_aH_ < pH < p*K*_a_, respectively (see also Figure 1). p*K*_a_ refers to the pH at which the acidic site on a molecule is 50% protonated, while p*K*_aH_ refers to the pH at which the basic site on a molecule is 50% protonated. While in general p*K*_a_ + p*K*_b_ = 14 in water, the same is not generally true for p*K*_aH_, i.e., p*K*_a_ + p*K*_aH_ ≠ 14.

The observed rate of reaction at pH = p*K*_a_ will be the result of constructive interference between *k*_2_ and *k*_4_. However, we can deduce the rate of each mechanism by observing the reaction at the right pH: log(*k*_*obs*_) = log(*k*_2_) at pH = 14 and log(*k*_*obs*_) = log(*k*_4_) at pH = p*K*_aH_. Similar logic can be applied to each of the other mechanisms.

**Figure 3:**
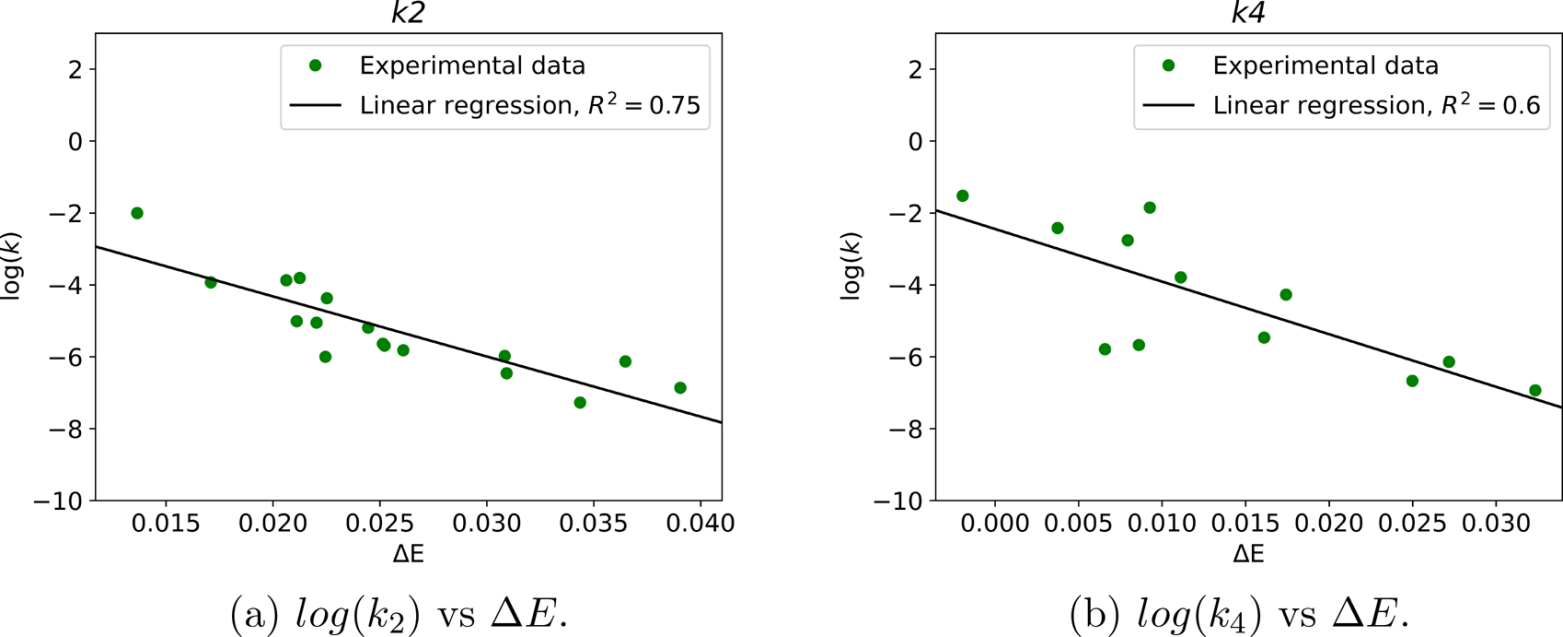
Linear regression of log(*k*) vs Δ*E* for the *k*_2_ and *k*_4_ mechanistic pathways.

This analysis is repeated for all mechanisms of all other BAs, and we find that there is a linear relationship between the maximum observed rate of a mechanism and the associated Δ*E* for each BA. This was already known to be true for the log(*k*_*2Ar*_) mechanism,^31^ and in this work we show that it is true for the other mechanisms as well. Linear regression for the *k*_2_ and *k*_4_ mechanisms can be seen in Figure 3. Using these linear relationships, the maximum rate of a mechanism for a novel BA can be predicted given the associated Δ*E*, which can be calculated using DFT. Leave-one-out cross-validation (LOOCV) was used to assess the quality of the fit for each molecule and for the construction of the parity plot for Cox’s molecules, while all data points were used for the prediction of protodeboronation rate for the novel molecules.

#### 5 Building a System of Linear Equations Using p*K*_a_, p*K*_aH_, and Predicted log(*k*_*n*_)

The rate curve as a function of pH can now be fully specified. For DMOBA the *k*_1_, *k*_2_, *k*_3_, and *k*_4_ mechanisms are active. The rate curve for *k*_2_ is a linear increase in rate with increasing pH until pH = p*K*_a_, after which the slope changes to 0. The rate function for *k*_2_ consists of 2 intersecting linear equations and can therefore be fully specified with 4 pieces of information: p*K*_a_ (measured experimentally), the maximum rate (calculated in the previous step), the slope when pH > p*K*_a_ (known to be 0), and the slope when pH < p*K*_a_ (determined by inspection of prior data). One can follow a similar argument to construct the rate curve for the other mechanisms, though of course the *k*_4_ mechanism consists of three intersecting linear equations rather than just two.

The slope of the linear increase/decrease in rate for each mechanism was determined by inspection of the data set and is reported below in units of log(*k*_*n*_)/pH:

1. *k*_1_: – 1
2. *k*_2_: 0.75
3. *k*_*2Ar*_: 0.75
4. *k*_*2cat*_: 2, – 2
5. *k*_3_: 2
6. *k*_4_: 0.75, – 0.75
7. *k*_5_: – 0.75

**Figure 4:**
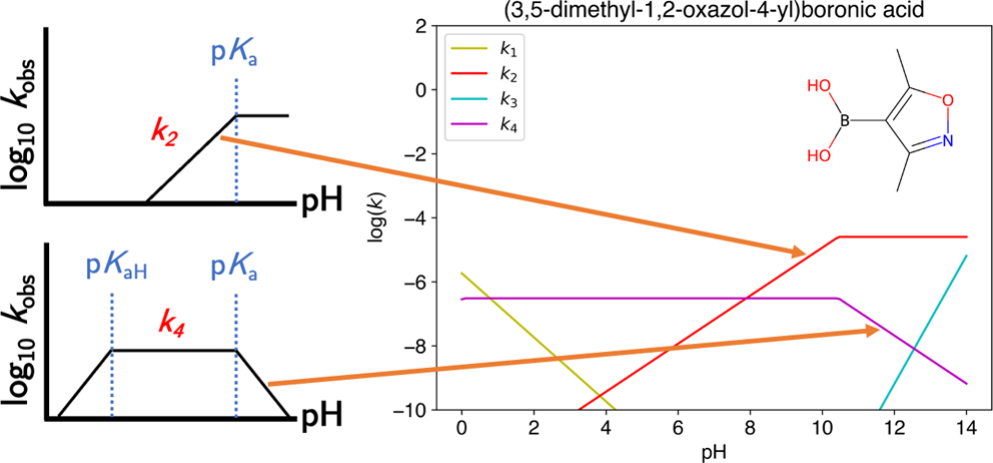
Constructing rate curves for each mechanism given the p*K*_a_, p*K*_aH_, and slope of mechanistic deactivation with pH.

With a fully specified system, we can now construct a predicted rate curve for each mechanism, as exemplified in Figure 4.

#### 6 Sum All Rate Curves

**Figure 5:**
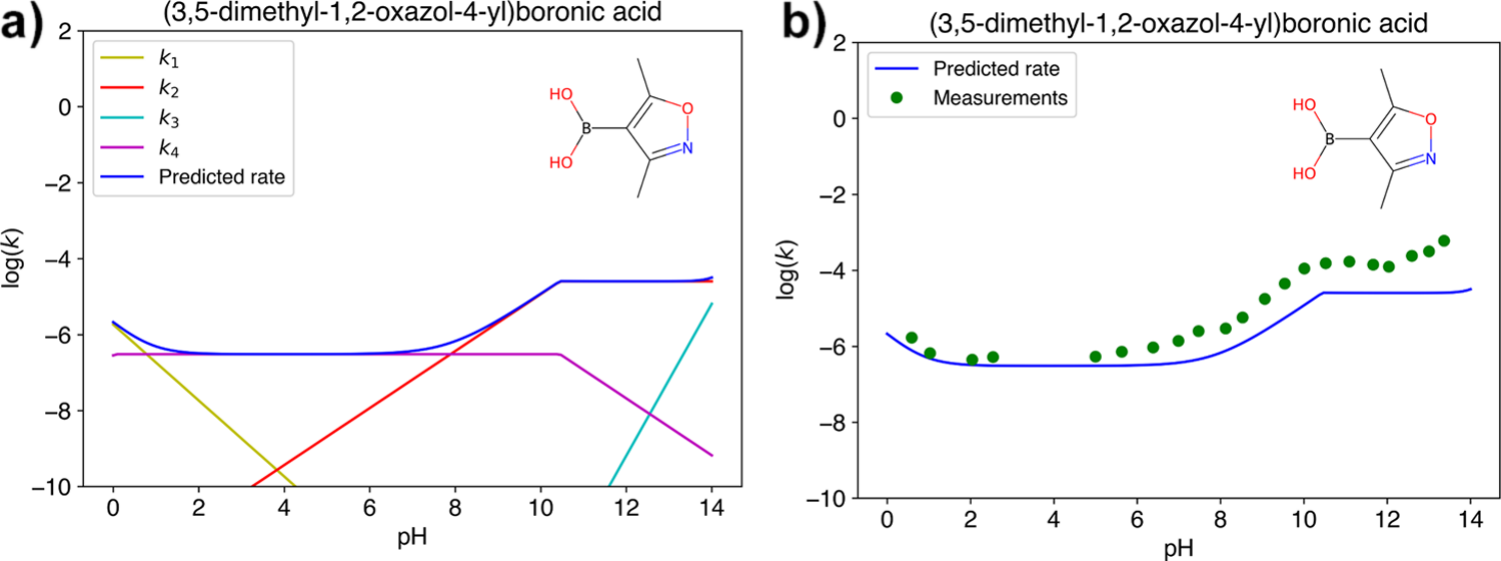
Predicted rate vs measured rate for DMOBA.

The rate of any one mechanism cannot be measured directly, and the rate which is observed in an experiment is the combined rate of all active mechanisms. Thus, the final step in predicting the observed rate of protodeboronation for a BA is to sum over the rate of all active mechanisms. See also Figure 5 for an example of this. The experimental rate measurements span from +2 (half-life of roughly 7 ms) to −9 (half-life of roughly 22 years), and the predicted overall rate has been capped to fall within this region as well.

##### Quantum Chemistry Calculations

The transition state and ground state energies for all substrates across all mechanisms except mechanism *k*_*2cat*_ were calculated using DFT in Gaussian using M06L/6-311++G** in water (using the keywords “SCRF = (Solvent = Water)”). The larger size of the *k*_*2cat*_ transition state presented convergence issues when optimizing with M06L/6-311++G**, so instead an optimized geometry was found using B3LYP/6-31G(d), and this was followed by a single point energy calculation with M06L/6-311++G**. M06L/6-311++G** was the preferred computational approach due to its relatively high accuracy; meanwhile, the B3LYP/6-31G(d) computational approach is faster but less accurate (e.g., because it underbinds if no dispersion corrections are used). Local energy minimum calculations were initiated with the keyword “Opt”, while transition state calculations were initiated with the keywords “Opt =(TS, CalcFC, noeigentest)”. Full details how the DFT calculations were performed for each mechanism, including example input files and example mechanistic schemes, can be found in the Supporting Information.

The *k*_*2cat*_ mechanism transition state, perhaps more accurately regarded as self-protodeboronation as opposed to autocatalytic, is a remarkably large structure featuring many atoms for large boronic acids, since the transition state for this mechanism involves the interaction between two boronic acid structures. Therefore, the *k*_*2cat*_ mechanism transition state needed to be simplified further, i.e., beyond just using a cheaper computational approach. The number of atoms involved in the transition state was reduced by replacing the boronic acid molecule which was not directly involved in bond breakage with a methyl group, as seen in Figure 1.

## Results and Discussion

Using the algorithm presented in this work enables the prediction of protodeboronation as a function of pH of BAs while only requiring knowledge of p*K*_a_, p*K*_aH_, and energy difference calculations using DFT. It thus enables *in silico* prediction of the protodeboronation rate as a function of pH. There is a linear relationship between the rate of a particular mechanism, *k*_*n*_, and the characteristic energy difference associated with that mechanism, and plots of these linear relationships can be found in the Supporting Information.

**Figure 6:**
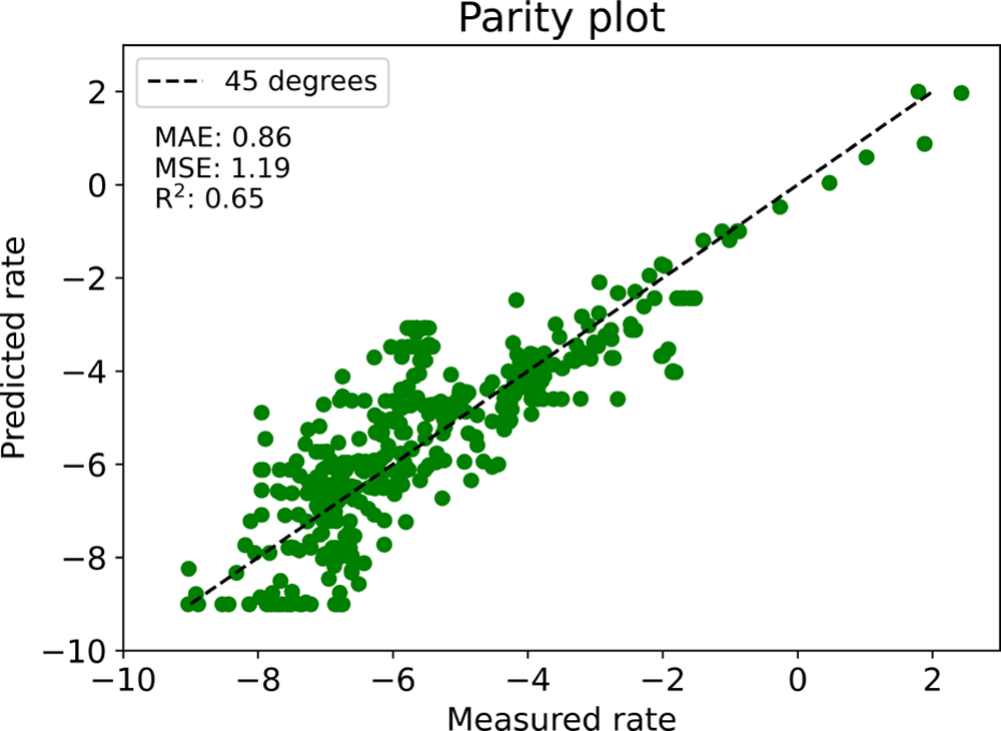
Parity plot showing the predictive accuracy of the algorithm. MAE: Mean Absolute Error. MSE: Mean Squared error. *R*^2^: Coefficient of Determination.

Given the limited amount of available data, the performance of the algorithm was assessed using leave-one-out cross-validation (LOOCV). In LOOCV each molecule is iteratively held out to form the validation set, while the remaining molecules act as the training set. The algorithm was used to generate predictions for all molecules in a manner consistent with LOOCV, and the rate predictions were then plotted against rate measurements in a parity plot, as in Figure 6.

We envision the ability to make *in silico* predictions of protodeboronation particularly useful to chemists running reactions featuring boronic acids, and we have used the algorithm to generate protodeboronation predictions for 50 novel boronic acids. Figures showing these predictions can be found in the Supporting Information and can be used “out of the box”. The associated code can be found on GitHub, should you want to generate predictions of your own. We also hope that this work may serve as inspiration as a method for predicting the rate of reaction when the mechanistic details about a reaction are known and can be simplified as a set of interacting linear equations.

### Predictions for Novel Boronic Acids

We have generated predictions of protodeboronation rate vs pH for 50 novel BAs, the graphs of which can be found in the Supporting Information. Of these 50, 38 BAs were selected from the top 100 most used boronic acids in the Reaxys database (after excluding boronic acids for which data already exists), while the remaining 12 were selected out of personal interest.

Thus, when a chemist is planning a reaction which involves a boronic acid, they will be able to look up the predicted rate of protodeboronation, and this may assist them in their reaction planning. Given how strongly the rate of protodeboronation depends on pH, it is important to know the pH of a reaction to understand how significant protodeboronation will be. Suzuki reactions are typically run under basic conditions, and prior work has shown that the pH can decrease by upward of 3 pH units over the course of the reaction.^45^ This makes it difficult to predict the extent of protodeboronation during a Suzuki reaction, even when the protodeboronation rate as a function of pH is known exactly.

Applying this algorithm to understand literature data may be difficult for reasons beyond the pH varying over the course of a reaction. The vast majority of Suzuki reactions do not report any pH value. Our analysis revealed over 200 000 reactions on Reaxys labeled as a “Suzuki Coupling”; however, only 399 of these reported a pH value, and of these 399 reactions, many reported a low pH value (below 7). Suzuki reactions are typically run in the presence of a base, and the reason for the majority of Suzuki reactions with a reported pH value being in the acidic region may be because the pH is only measured and reported when deviating from the norm.

### Model Explainability and Extrapolation

The protodeboronation prediction algorithm relies on an accurate understanding of the active mechanisms, precise DFT calculations, and (measured) p*K*_a_ and p*K*_aH_ values (if applicable). Each of these aspects of the model contributes its own domain of applicability to the model, which can make the overall domain of applicability difficult to understand; however, as a guiding principle, the closer a novel molecule is to a molecule which already appears in the training data set, the more trustworthy the prediction. The rate predictions were capped to always fall between −9 and +2 (≈22 years and 7 ms), since this was the range of rate values reported in the data, and also because this would cover a sufficiently large space for any practical considerations.

The highest accuracy predictions can be made for molecules which are active with the *k*_*2Ar*_ mechanism, since only one mechanism is active, it has the most data, as well as a near perfect correlation (*R*^2^ = 0.97) in the linear regression of Δ*E* against log(*k*) (see Figure 1c in the Supporting Information). The particular case of halogens attached to an aromatic ring containing a boronic acid was studied in detail by Cox,^30^ where he observed that the more halogens attached to the ring, and the closer these halogens are to the boronic acid, the faster the rate of protodeboronation. The trend of increasing rate of protodeoboronation as a halogen moves closer to the boronic acid can be observed by considering rate measurements and predictions for molecules 74–76 (see Section 4 of the Supporting Information). The fastest rate is observed with molecule 72, which has fluoride occupying all five positions on the ring (with the sixth position being the boronic acid). It is therefore reassuring to see the algorithm predicting high rates for molecules 102/112/148 and 103/111/149, as these are also boronic acids with halogens on the ring, with the fluorides having been replaced with chloride and bromide, respectively.

It is noteworthy that all the molecules dealt with in this work are boronic acids. It is unlikely that the algorithm would extend to molecules containing Bpins or other cousins of the boronic acids, as the mechanistic pathways are likely to be different. Each of the linear regression models of Δ*E* against log(*k*) (see Figure 1 in the Supporting Information) also provides guidance of the domain of applicability of the model. The *k*_3_ and *k*_5_ mechanisms have only two data points each, which is not enough to produce a convincing relationship. However, the reason for this lack of data is that molecules active with these mechanisms are quite uncommon, which in turn decreases the need for such predictions to be made. Furthermore, the range of energy difference (Δ*E*) observed for each mechanism may also indicate the domain of applicability for each mechanism. As an example, the range of Δ*E* values observed for the *k*_*2cat*_ mechanism ranges from −0.0137 to +0.0065 hartree. The energy difference calculated for the *k*_*2cat*_ mechanism for molecule 134 is −0.0658 hartree, which is quite far outside the range of values observed for the training molecules, and results in the rate prediction to reach the cap of log(*k*) = +2 (≈7 ms) at pH = p*K*_a_. The rate prediction for molecule 134 certainly looks interesting, so should the aforementioned reasons be grounds to dismiss the prediction or should it be trusted since the prediction conforms to chemical expectations? (In other words, we know that electron density pulled away from the bond attached to the boronic acid will speed up protodeboronation; this is most notable for the fluorinated boronic acids, and in the case of molecule 134, the phenyl ring pulls electron density away from the conjugated double bond that the boronic acid is attached to.) Only lab experimentation would be able to settle this debate. Regardless, this molecule, as well as molecules 132 and 143–147, were included to explore how the algorithm behaves near the edge of its domain of applicability.

Overall, the algorithm is capable of making remarkably good predictions on the rate of protodeboronation, given the complexity of the many simultaneous mechanistic pathways. The coefficient of determination (*R*^2^) being 0.65 indicates that the algorithm is capable of explaining a majority of the variability in the data set, and with a mean absolute error of 0.86, one can expect predictions from the algorithm to typically be within 1 log(*k*) of the true rate. This can be valuable information for a chemist to help them understand whether the rate of protodeboronation for their boronic acid of interest under certain pH conditions will be on the order of magnitude of seconds, days, or years.

### Future Work

Protodeboronation is a side reaction, and a significant challenge remains in understanding the rate of the intended reaction, be that the Suzuki reaction or any other. Ideally, the rate of the intended reaction should be significantly faster than the rate of the side reaction. This work allows for *in silico* prediction of the rate of protodeboronation. However, we do not know of any work which allows for *in silico* predictions of the rate of intended reactions.

The protodeboronation prediction algorithm relies on knowing p*K*_a_ and p*K*_aH_ of the queried BA. For the 50 BAs included in the initial model building, these values had been measured in a lab. However, finding experimentally measured p*K*_a_ and p*K*_aH_ values either in the literature or in a database such as DataWarrior^46^ may not be possible for novel BAs. Using machine learning to predict p*K*_a_ and p*K*_aH_ is a potential solution for extrapolating from existing data. However, the uncertainty associated with machine learning models in chemistry can be significant, particularly when the domain of applicability is unclear (i.e., it is not known which molecules fall within the scope and beyond the scope of the model). p*K*_a_ and p*K*_aH_ varies greatly between solvents, and we were unable to find a ML model built to predict these values in a 50/50 water/dioxane mixture. Therefore, the p*K*_a_ and p*K*_aH_ values used for the 50 novel BAs were simply the mean values of Cox’s molecules. Given the notable overlap in structure between the two sets of boronic acids, simply using the mean will likely still yield highly informative predictions.

This work sprung out from a general mechanistic model for protodeboronation, and we believe if general mechanistic models are developed for other reactions, it may become easier to create workflows similar to this for generating *in silico* rate predictions. We look forward to applying this methodology to other systems.

## Conclusions

Protodeboronation is a significant and unintended side reaction of numerous notable reactions, including the Suzuki–Miyaura reaction, and any work which can aid in our understanding of how to avoid it may provide much value to the chemistry community. In this work we present a 6 step algorithm for *in silico* prediction of the rate of protodeboronation of boronic acids. The algorithm relies on mechanistic insight from previously published literature. Protodeboronation can occur along 7 distinct mechanistic pathways, each having its own characteristic energy difference. There is linear correlation between this energy difference and the reaction rate of the associated mechanistic pathway. Adding the rate of all mechanistic pathways finally yields the observed rate of reaction. Leave-one-out cross-validation revealed that this algorithmic approach provides predicted protodeboronation rates with high accuracy. The algorithm requires two inputs from the user: acidity information (p*K*_a_ and possibly p*K*_aH_) and energy difference calculations using Density Functional Theory (DFT) for each active mechanism. Data on p*K*_a_ and p*K*_aH_ are not available for all boronic acids, and predicting these values may introduce additional uncertainty into the algorithm. The need to perform quite a few DFT calculations for each novel boronic acid is a significant bottleneck for the widespread use of this algorithm, so to make this work more accessible, we have made protodeboronation predictions for 50 academically and industrially important boronic acids, which can be found in the Supporting Information.

## Data Availability

All the code and data associated with this project is freely available on https://github.com/sustainable-processes/protodeboronation-prediction, allowing anyone to generate a protodeboronation rate predictions for BAs that fall within the scope of the model, given appropriate DFT calculations and approximate values for p*K*_a_ and p*K*_aH_ (if applicable).

## Supporting Information Available

A detailed explanation of each protodeboronation mechanism, protodeboronation predictions for 50 novel boronic acids, and optimized structures and energies for each relevant mechanism for all 100 molecules (PDF)
