# Supplementary material for: Quantitative In Silico Prediction of the Rate of Protodeboronation by a Mechanistic Density Functional Theory-Aided Algorithm
Source: J Phys Chem A. 2023 Mar 14;127(11):2628–36. doi: 10.1021/acs.jpca.2c08250 (PMC10041635; doi:10.1021/acs.jpca.2c08250)
Supplement: Supplementary file 1 — jp2c08250_si_001.pdf [file jp2c08250_si_001.pdf]

Supplementary Information: Quantitative *In Silico* Prediction of the Rate of Protodeboronation by a Mechanistic Density Functional Theory-Aided Algorithm

Daniel S. Wigh,[†] Matthieu Tissot,[‡] Patrick Pasau,[‡] Jonathan M. Goodman,[¶] and Alexei A. Lapkin[*,†]

†*Department of Chemical Engineering and Biotechnology, University of Cambridge, Cambridge, UK*

‡*UCB Biopharma SPRL, 1420 Braine l'Alleud, Belgium*

¶*Yusuf Hamied Department of Chemistry, University of Cambridge, Cambridge, UK*

E-mail: aal35@cam.ac.uk

Contents

1 Detailed Description of Mechanisms **3**

1.1 k_1: Acid-Catalysed Mechanism 3

1.2 k_2: Base-Catalysed Mechanism 3

1.3 k_{2Ar}: Aryl Boronic Acid Mechanism 4

1.4 k_{2cat}: Self-Protodeboronation Mechanism 5

1.5 k_3: Double Negative Charge Mechanism 5

1.6 k_4: Zwitterionic Mechanism 6

1.7 k_5: Protonated Basic Site Mechanism . . . 7

2 Categorisation of Boronic Acids **7**

2.1 From Cox's Study . . . 10

2.2 Novel molecules . . . 12

3 Quantum Chemical Calculations **14**

3.1 Transition State Exploration . . . 14

3.1.1 Molecule 50: k_2 transition state calculation . . . 15

3.1.2 Molecule 50: k_{2cat} transition state calculation . . . 17

3.1.3 Molecule 60: k_3 transition state calculation . . . 18

3.1.4 Molecule 60: k_4 transition state calculation . . . 20

3.1.5 Molecule 64: k_5 transition state calculation . . . 21

4 Cox's Molecules: Predicted vs Measured Rate **22**

5 Novel Molecules: Predicted Rate **28**

6 DFT Calculations **34**

6.1 Optimised Geometries and Energies for Cox's Molecules . . . 34

6.1.1 k_1 . . . 34

6.1.2 k_2 . . . 46

6.1.3 k_{2Ar} . . . 71

6.1.4 k_{2cat} (B3LYP) . . . 110

6.1.5 k_{2cat} (M06L) . . . 123

6.1.6 k_3 . . . 136

6.1.7 k_4 . . . 139

6.1.8 k_5 . . . 156

6.2 Optimised Geometries and Energies for Novel Molecules . . . 160

6.2.1 k_1 . . . 160

6.2.2 k_2 . . . 163

6.2.3 k_{2Ar} . . . 174

6.2.4 k_{2cat} (B3LYP) . . . 230

6.2.5 k_{2cat} (M06L) . . . 234

6.2.6 k_3 . . . 238

6.2.7 k_4 . . . 238

6.2.8 k_5 . . . 252

References **252**

1 Detailed Description of Mechanisms

1.1 k_1: Acid-Catalysed Mechanism

The acid catalysed mechanism is initiated by a proton attacking an aromatic double bond close to the boron, which creates a carbocation. The boronic acid then combines with a water molecule, before finally splitting in two: an aromatic heterocycle and $B(OH)_3$, as seen in Scheme 1.

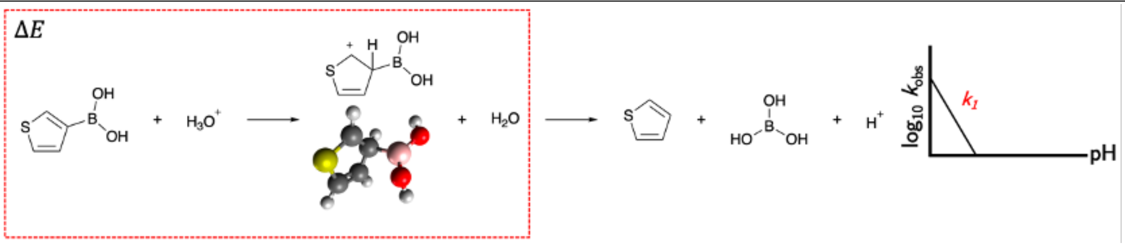

Scheme 1: The k_1 mechanistic pathway for molecule 50.

1.2 k_2: Base-Catalysed Mechanism

The base-catalysed mechanism is initiated through the addition of OH^- to the boronic acid as well as attachment of a water molecule near the $C-B$ bond; the order at which this

happens is irrelevant, since both need to happen before the k_2 mechanism can progress. Finally, the boronic acid detaches from the rest of the molecule, as per Scheme 2. It occurs at the maximum rate in a basic environment, when pH>pKa.

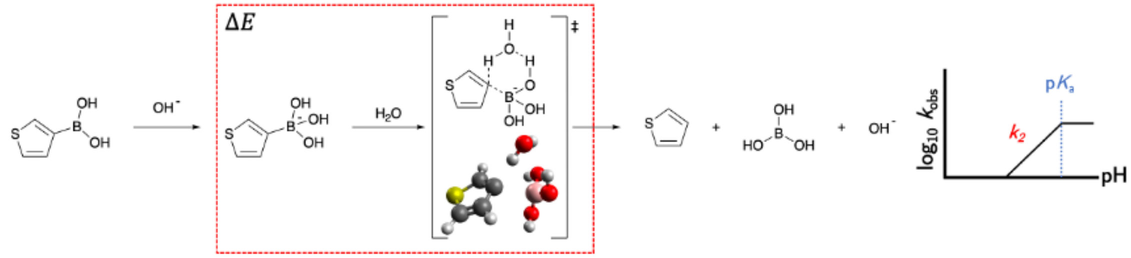

Scheme 2: The k_2 mechanistic pathway for molecule 50.

1.3 k_{2Ar}: Aryl Boronic Acid Mechanism

The aryl boronic acid (k_{2Ar}) mechanistic pathway occurs when dealing with aryl boronic acids. The data from Cox's study focuses on the effect of placing strongly electron withdrawing atoms on the ring, particularly fluorine atoms, and generally the rate of protodeboronation increases as the electron withdrawing effect becomes stronger (e.g. with the strongly electronegative fluorine close to the boronic acid in the ortho-position). The model also applies to aryl boronic acids with electron donating substituents on the ring, though in this case the rate of protodeboronation is exceedingly slow. Of the 30 aryl boronic acids in the data set, a majority (19) only featured fluorine (and hydrogen) atoms on the ring, so perhaps unsurprisingly the model predictions for the fluorinated aryl boronic acids are slightly better than for other types of aryl boronic acids. However, with an overall $R^2 = 0.97$ (see Figure 1c), the model is clearly also useful for predicting the rate of protodeboronation for other types of aryl boronic acids. The reaction scheme for the k_{2Ar} mechanism can be seen in Scheme 3. It occurs at maximum rate when $pH \geq pK_a$.

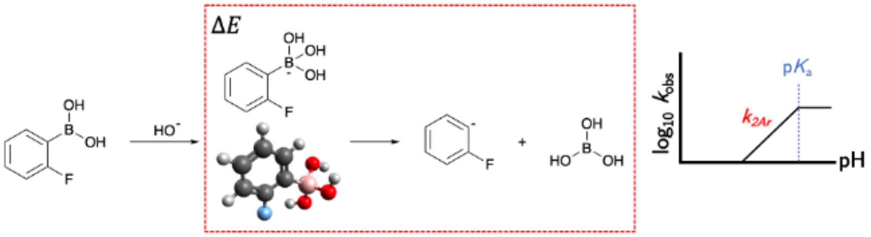

Scheme 3: The k_{2Ar} mechanistic pathway for molecule 74.

1.4 k_{2cat}: Self-Protodeboronation Mechanism

Labelled k_{2cat} by Cox in reference to autocatalysis, this mechanistic pathway features a transition state with two boronic acids reactants, as in Scheme 4 and Scheme 5. Since this mechanism effectively represents catalysis of protodeboronation by a reactant we believe self-protodeboronation would be a more appropriate name, as autocatalysis typically refers to catalysis by the reaction product. This distinction has important implications when considering how the rate changes with time: autocatalytic reactions speed up with time (as more product is formed and becomes available to catalyse the reaction), whereas self-protodeboronation slows down with time (as reactant BAs degrade). It occurs at maximum rate when pH ≈ pKa.

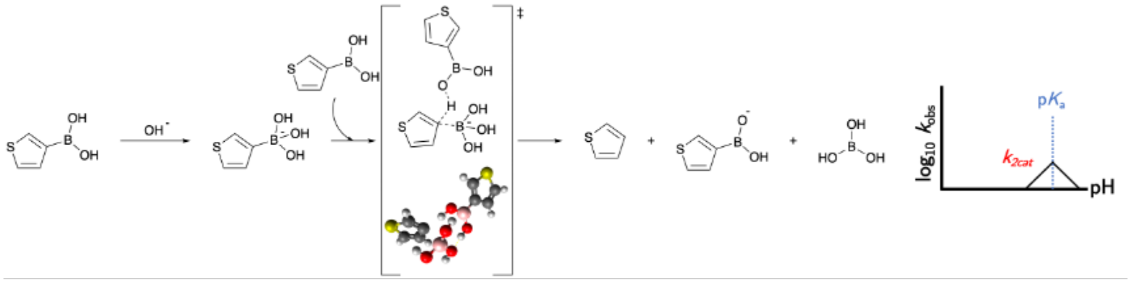

Scheme 4: The k_{2cat} mechanistic pathway for molecule 50.

1.5 k_3: Double Negative Charge Mechanism

The k_3 mechanism only occurs at a high rate when the pH is very high, due to the boronic acid being doubly negatively charged, as evident in Scheme 6. It is worth noting that there

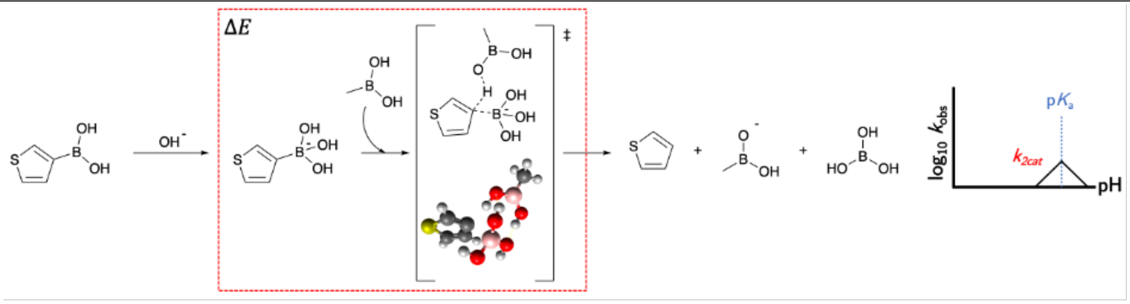

Scheme 5: The "compromise" mechanistic pathway for k_{2cat}, featuring a methyl group substitution, alongside an example showing what the compromise k_{2cat} transition state looks like for molecule 50.

are only two molecules (60 and 61) that undergo k_3, so the leave-one-out cross-validation approach causes the predicted rate of k_3 for the held out molecule to be equal to that of the one that is not held out. The k_3 mechanism only applies to niche boronic acids, and since none of the novel boronic acids feature the k_3 protodeboronation mechanism, the lack of data for this mechanism does not impact the out of sample predictions.

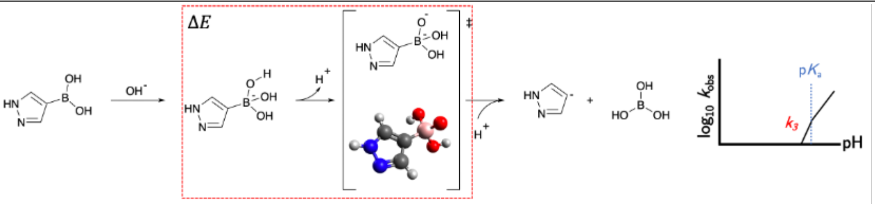

Scheme 6: The k_3 mechanistic pathway for molecule 60.

1.6 k_4: Zwitterionic Mechanism

The k4 mechanism is active when the boronic acid has a basic site. The basic sites will be mostly protonated when $pH \leq pK_{aH}$. Most of the acidic sites (boronic acids) will have an additional OH attached and become negatively charged when $pH \geq pK_a$ (though of course addition of H+ and OH- won't be 100%). The k4 mechanism only works when there is simultaneously H+ on the basic site and OH- on the boronic acid (as seen on Scheme 7, so it will be at maximum rate when pH is between pK_{aH} and pK_a. Furthermore, the k4 mechanism happens faster the closer the basic site is to the boronic acid.

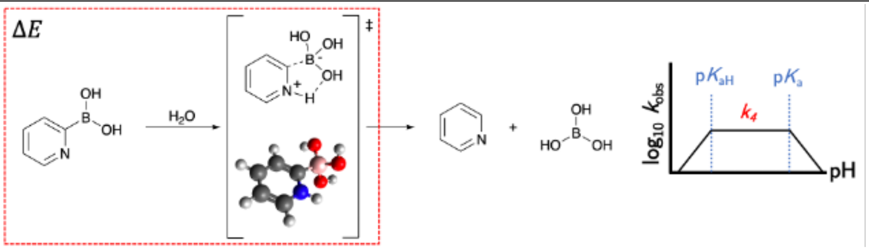

Scheme 7: The k_4 mechanistic pathway for molecule 44.

1.7 k_5: Protonated Basic Site Mechanism

The k5 mechanism occurs when the boronic acid has a basic site, and progresses at the maximum rate when $pH \leq pKaH$ since it requires the basic site to stay protonated throughout the course of the reaction, as seen on figure 8. It is worth noting that there are only two molecules (64 and 65) that undergo k_5, so the leave-one-out cross-validation approach causes the predicted rate of k_5 for the held out molecule to be equal to that of the one that is not held out. The k_5 mechanism only applies to niche boronic acids, and since none of the novel boronic acids feature the k_5 protodeboronation mechanism, the lack of data for this mechanism does not impact the out of sample predictions.

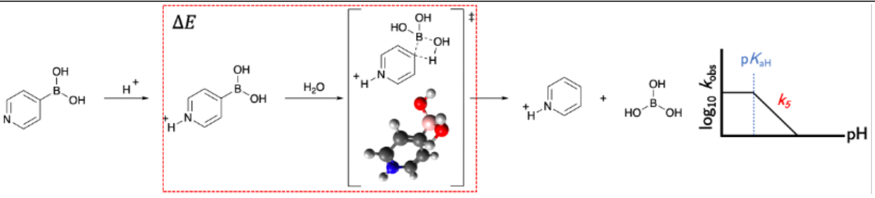

Scheme 8: The k_5 mechanistic pathway for molecule 64.

2 Categorisation of Boronic Acids

A total of 100 boronic acids were handled in this study. 50 boronic acids from Cox's study (for which ground truth measurements exist), and 50 novel boronic acids (for which no ground truth measurements exist). Each boronic acid was categorised into one of the following four groups (in line with Figure 2):

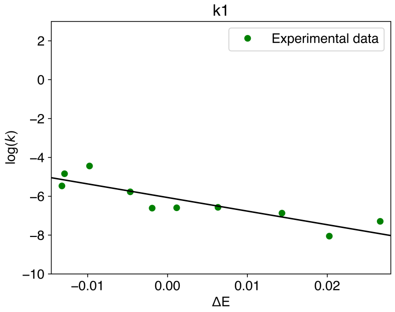

(a) k_1

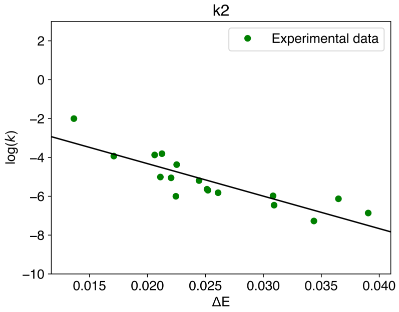

(b) k_2

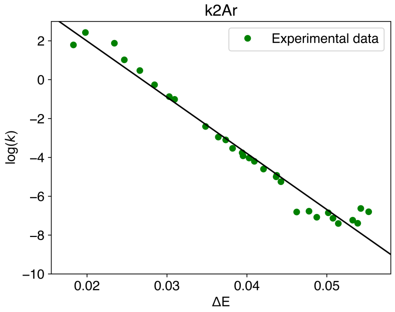

(c) k_{2Ar}

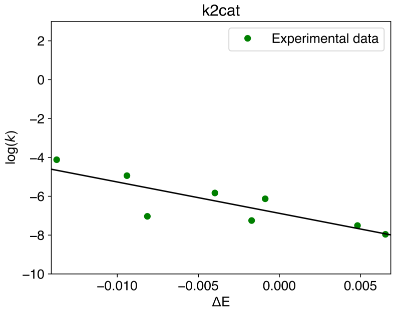

(d) k_{2cat}

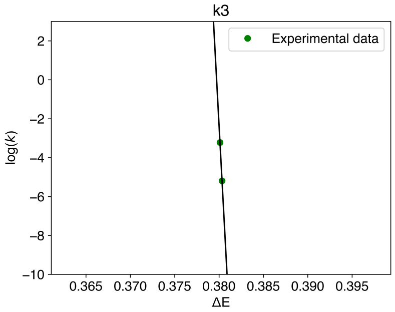

(e) k_3

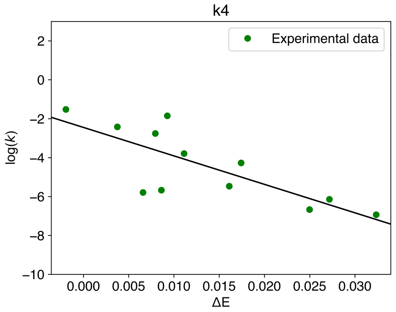

(f) k_4

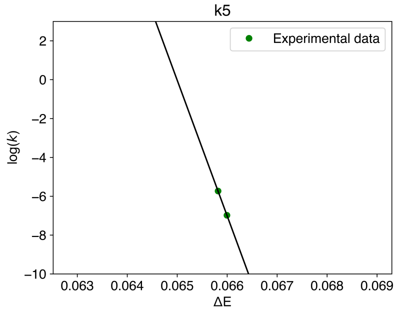

(g) k_5

Figure 1: Linear regression of ΔE against $log(k_n)$, where ΔE is the characteristic energy difference of each mechanistic pathway. As expected, the rate becomes slower as the energy barrier (ΔE) grows. We would also expect (ΔE) to be positive, since the energy of the TS should be higher than that of the reactants. ΔE in k_1 and k_{2cat} is probably negative due to the DFT calculations of the reactants being performed separately (implying infinite separation, which removes favourable interactions that could lower the energy).

- Reactive basic heterocycles (RBH)
- Other basic heterocycles (OBH)
- Non-basic boronic acids (NBBA)
- Aryl boronic acids (ABA)

The tables below show which group each boronic acid belongs to, as well as which mechanisms are active for that molecule. 38 of the novel molecules were selected as a subset from the 100 most commonly used boronic acids in Reaxys, while the remaining were selected out of personal interest.

2.1 From Cox's Study

#	Type	Mechanisms	SMILES
28	ABA	*k2Ar*	COc1ccc(B(O)O)cc1
44	RBH	*k4*	OB(O)c1ccccn1
50	NBBA	*k1, k2, k2cat*	OB(O)c1ccsc1
51	NBBA	*k1, k2, k2cat*	OB(O)c1ccco1
52	NBBA	*k1, k2, k2cat*	OB(O)c1cccs1
54	NBBA	*k1, k2, k2cat*	C=CB(O)O
55	NBBA	*k1, k2, k2cat*	OB(O)C1CC1
56	NBBA	*k1, k2, k2cat*	CB(O)O
57	NBBA	*k1, k2, k2cat*	OB(O)C1CCC1
58	NBBA	*k1, k2, k2cat*	OB(O)C1CCCCC1
60	OBH	*k1, k2, k3, k4*	OB(O)c1cn[nH]c1
61	OBH	*k1, k2, k3, k4*	Cc1noc(C)c1B(O)O
62	OBH	*k2, k4*	OB(O)c1cncnc1
63	OBH	*k2, k4*	OB(O)c1cccnc1
64	OBH	*k2, k4, k5*	OB(O)c1ccncc1
65	OBH	*k2, k4, k5*	OB(c1c2c(NC=C2)ncc1)O
66	RBH	*k2, k4*	OB(O)c1cncs1
67	RBH	*k2, k4*	Cn1nccc1B(O)O
68	RBH	*k2, k4*	OB(O)c1ccn[nH]1
69	RBH	*k4*	COc1cccc(B(O)O)n1
70	RBH	*k4*	OB(O)c1cccc(C(F)(F)F)n1
71	ABA	*k2Ar*	OB(O)c1c(F)cccc1F
72	ABA	*k2Ar*	OB(O)c1c(F)c(F)c(F)c(F)c1F
73	ABA	*k2Ar*	OB(O)c1ccccc1
74	ABA	*k2Ar*	OB(O)c1ccccc1F

#	Type	Mechanisms	SMILES
75	ABA	*k2Ar*	OB(O)c1cccc(F)c1
76	ABA	*k2Ar*	OB(O)c1ccc(F)cc1
77	ABA	*k2Ar*	OB(O)c1cccc(F)c1F
78	ABA	*k2Ar*	OB(O)c1ccc(F)cc1F
79	ABA	*k2Ar*	OB(O)c1cc(F)ccc1F
80	ABA	*k2Ar*	OB(O)c1ccc(F)c(F)c1
81	ABA	*k2Ar*	OB(O)c1cc(F)cc(F)c1
82	ABA	*k2Ar*	OB(O)c1ccc(F)c(F)c1F
83	ABA	*k2Ar*	OB(O)c1cc(F)cc(F)c1F
84	ABA	*k2Ar*	OB(O)c1cc(F)c(F)cc1F
85	ABA	*k2Ar*	OB(O)c1cc(F)c(F)c(F)c1
86	ABA	*k2Ar*	OB(O)c1cc(F)c(F)c(F)c1F
87	ABA	*k2Ar*	OB(O)c1c(F)ccc(F)c1F
88	ABA	*k2Ar*	OB(O)c1c(F)cc(F)cc1F
89	ABA	*k2Ar*	OB(O)c1c(F)cc(F)c(F)c1F
90	ABA	*k2Ar*	OB(O)c1c(F)c(F)cc(F)c1F
91	ABA	*k2Ar*	COc1ccc(B(O)O)c(F)c1
92	ABA	*k2Ar*	OB(O)c1ccc(C(F)(F)F)cc1F
93	ABA	*k2Ar*	FC1=C(B(O)O)C=C([N+]([O-])=O)C=C1
94	ABA	*k2Ar*	COc1cc(F)c(B(O)O)c(F)c1
95	ABA	*k2Ar*	COc1c(F)c(F)c(B(O)O)c(F)c1F
96	ABA	*k2Ar*	OB(O)c1cc(C(F)(F)F)cc(C(F)(F)F)c1
97	ABA	*k2Ar*	OB(O)C1=CC([N+]([O-])=O)=CC([N+]([O-])=O)=C1
98	ABA	*k2Ar*	Cc1ccc(B(O)O)cc1
99	ABA	*k2Ar*	OB(O)c1cccc(Cl)c1

2.2 Novel molecules

#	Type	Mechanisms	Origin	SMILES
101	ABA	*k2Ar*	Reaxys	OB(O)C1=CC=C(OC(F)(F)F)C=C1
102	ABA	*k2Ar*	Own selection	OB(O)C1=C(Cl)C(Cl)=C(Cl)C(Cl)=C1Cl
103	ABA	*k2Ar*	Own selection	OB(O)C1=C(Br)C(Br)=C(Br)C(Br)=C1Br
104	ABA	*k2Ar*	Reaxys	OB(O)C1=CC(=CC=C1)C#N
105	ABA	*k2Ar*	Reaxys	CN(C)C1=CC=C(C=C1)B(O)O
106	OBH	*k2, k4*	Own selection	OB(O)C1=CC=CN=C1C
107	ABA	*k2Ar*	Reaxys	OB(O)C1=CC=C(Cl)C(Cl)=C1
108	ABA	*k2Ar*	Reaxys	CSC1=CC=C(C=C1)B(O)O
109	ABA	*k2Ar*	Reaxys	OB(O)C1=CC(O)=CC=C1
110	ABA	*k2Ar*	Reaxys	OB(O)C1=CC=CC=C1C(F)(F)F
111	ABA	*k2Ar*	Own selection	OB(O)C1=C(Br)C=CC=C1Br
112	ABA	*k2Ar*	Reaxys	OB(O)c1ccc(Cl)cc1
113	ABA	*k2Ar*	Reaxys	OB(O)c1ccc(C(F)(F)F)cc1
114	ABA	*k2Ar*	Reaxys	Cc1ccccc1B(O)O
115	ABA	*k2Ar*	Reaxys	OB(O)c1cccc2ccccc12
116	ABA	*k2Ar*	Reaxys	COc1cccc(B(O)O)c1
117	ABA	*k2Ar*	Reaxys	OB(O)c1ccc2ccccc2c1
118	ABA	*k2Ar*	Reaxys	O=Cc1ccc(B(O)O)cc1
119	ABA	*k2Ar*	Reaxys	COc1ccccc1B(O)O
120	ABA	*k2Ar*	Reaxys	N#Cc1ccc(B(O)O)cc1
121	ABA	*k2Ar*	Reaxys	Cc1cccc(B(O)O)c1
122	ABA	*k2Ar*	Reaxys	OB(O)c1ccc(Br)cc1
123	ABA	*k2Ar*	Reaxys	CC(C)(C)c1ccc(B(O)O)cc1
124	ABA	*k2Ar*	Reaxys	COC(=O)c1ccc(B(O)O)cc1
125	RBH	*k4*	Own selection	OB(C1=C(C=CC=N1)C=O)O

#	Type	Mechanisms	Origin	SMILES
126	ABA	*k2Ar*	Reaxys	O=[N+]([O-])c1cccc(B(O)O)c1
127	ABA	*k2Ar*	Reaxys	CC(=O)c1ccc(B(O)O)cc1
128	ABA	*k2Ar*	Own selection	OB(c1cc(Cl)cc(F)c1)O
129	ABA	*k2Ar*	Reaxys	OB(O)c1ccc(O)cc1
130	ABA	*k2Ar*	Reaxys	OB(O)c1cccc(C(F)(F)F)c1
131	ABA	*k2Ar*	Reaxys	OB(O)c1ccccc1Cl
132	RBH	*k4*	Own selection	OB(C1=C(C=CC=N1)CC(OCC)=O)O
133	NBBA	*k2Ar*	Reaxys	Cc1cc(C)cc(B(O)O)c1
134	NBBA	*k1, k2, k2cat*	Reaxys	OB(O)/C=C/c1ccccc1
135	ABA	*k2Ar*	Reaxys	O=[N+]([O-])c1ccc(B(O)O)cc1
136	NBBA	*k2Ar*	Reaxys	OB(O)c1ccc2c(c1)OCO2
137	NBBA	*k2Ar*	Reaxys	COc1ccc(B(O)O)cc1OC
138	NBBA	*k2Ar*	Reaxys	O=C(O)c1ccc(B(O)O)cc1
139	ABA	*k2Ar*	Reaxys	O=Cc1ccccc1B(O)O
140	NBBA	*k2Ar*	Reaxys	C=Cc1ccc(B(O)O)cc1
141	NBBA	*k1, k2, k2cat*	Reaxys	OB(O)c1ccoc1
142	ABA	*k2Ar*	Reaxys	OB(O)c1cc(C=O)ccc1
143	OBH	*k2, k4*	Own selection	OB(O)C1=C(CC(OCC)=O)N=CC=C1
144	OBH	*k2, k4*	Own selection	NC1=C(B(O)O)C=NC(C)=C1
145	RBH	*k4*	Own selection	OB(O)C1=CC(N)=C(C)C=N1
146	RBH	*k4*	Own selection	OB(O)C1=C(OC)C=C(OC)C=N1
147	RBH	*k2, k4*	Own selection	OB(O)C1=NC=CO1
148	ABA	*k2Ar*	Own selection	OB(O)C1=C(Cl)C=CC=C1Cl
149	ABA	*k2Ar*	Reaxys	OB(O)C1=C(Br)C=CC=C1
150	ABA	*k2Ar*	Reaxys	OB(O)C1=CC(Br)=CC=C1

3 Quantum Chemical Calculations

3.1 Transition State Exploration

Quantum Chemical Calculations using Density Functional Theory in Gaussian were performed to calculate ΔE for each mechanism for each molecule, where ΔE is the characteristic energy difference. For each characteristic energy difference featuring a transition state, it was first necessary to verify that a valid transition state could exist. Three molecules were selected such that at least one transition state for each mechanism could be calculated. To verify that a given transition state can exist, only one example is necessary, so only the results of one transition state is shown for each mechanism (highlighted in **bold** below).

The chosen molecules were:

1. Molecule 50: k_1, $\mathbf{k_2}$, $\mathbf{k_{2cat}}$
2. Molecule 60: k_1, k_2, $\mathbf{k_3}$, $\mathbf{k_4}$
3. Molecule 64: k_2, k_4, $\mathbf{k_5}$

Energy calculations were performed using Gaussian using the same specifications as:[1] Computational approach: *M06/6-311+G***, solvent: water (using the *SCRF = (Solvent = Water)* keywords).

Below is an example Gaussian input file for optimising to a transition state (TS).

```
%NProcShared=2
#n M06L/6-311++G** SCRF=(Solvent=Water) Opt=(TS,CalcFC,noeigentest)

 60_k4_TS

0 1
C          1.06226        0.54933       -1.82951
C          1.95702       -0.43788       -1.46054
N          1.46252       -1.07654       -0.38484
```

```
C           0.02270        0.45907       -0.92119
N           0.29932       -0.53717       -0.06051
H          -0.29648       -0.84705        0.73895
H           2.89875       -0.67460       -1.93872
B           1.21256        1.56157       -3.04010
H          -0.86952        1.07071       -0.88654
O           1.29068        0.80178       -4.30563
O           0.03952        2.46010       -3.09973
H           2.17945        0.36176       -4.31937
H           0.12373        3.08638       -2.33511
O           2.44491        2.35967       -2.85516
H           2.49897        2.96659       -3.63747
H           1.91112       -1.87062        0.12343


```

Listing 1: Gaussian input file for the k_4 transition state of molecule 60. Generated using Avogadro. Note that Gaussian expects two empty lines at the bottom of an input file.

To instead optimise to a local energy minimum, we simply replaced *Opt=(TS, CalcFC, noeigentest)* with *Opt*. Do note the need for two empty lines at the bottom of the input file.

For the transition state to be valid, we first check that it has exactly one negative eigenvalue, then that it looks "reasonable".

3.1.1 Molecule 50: k_2 transition state calculation

On the first run of this mechanism, we encountered a timeout error. The last geometry from this run was fed to a second run. In the second run, we successfully found a valid transition state (see below).

```

                            Standard orientation:
 ---------------------------------------------------------------------
 Center     Atomic      Atomic             Coordinates (Angstroms)
```

```
 Number     Number       Type             X           Y           Z
 ---------------------------------------------------------------------
      1         16          0       2.764177    0.109865   -0.550787
      2          6          0       1.142945   -0.348938   -0.958102
      3          6          0       0.270104   -0.285523    0.099296
      4          6          0       0.970288    0.151877    1.267689
      5          6          0       2.303131    0.401453    1.081148
      6          1          0       0.939718   -0.638890   -1.982042
      7          5          0      -1.315223   -0.663127    0.039377
      8          1          0       0.492886    0.290507    2.233745
      9          1          0       3.036513    0.742964    1.798555
     10          8          0      -1.971583   -0.195651   -1.217216
     11          8          0      -2.100353    0.045622    1.091104
     12          1          0      -1.824503   -0.271433    1.952644
     13          1          0      -1.524431   -0.587345   -1.969353
     14          8          0      -1.409836   -2.131831    0.191274
     15          1          0      -2.341741   -2.367446    0.172040
     16          1          0      -2.017389    1.713942   -0.967131
     17          8          0      -2.073175    2.440533   -0.320871
     18          1          0      -2.091002    1.892892    0.482737
 ---------------------------------------------------------------------

(...)

     Eigenvalues ---   -0.00197   0.00114   0.00202   0.00362   0.00389
     Eigenvalues ---    0.00588   0.00813   0.01065   0.01305   0.01536
     Eigenvalues ---    0.02206   0.02718   0.03557   0.04286   0.04925
     Eigenvalues ---    0.05447   0.06227   0.06898   0.08314   0.09072
     Eigenvalues ---    0.09634   0.10548   0.10785   0.11483   0.11750
     Eigenvalues ---    0.13193   0.16051   0.16197   0.16597   0.19643
     Eigenvalues ---    0.20048   0.20945   0.21899   0.23256   0.26795
     Eigenvalues ---    0.28250   0.32909   0.35431   0.36001   0.36517
     Eigenvalues ---    0.40285   0.43892   0.46673   0.48415   0.53178
```

```
     Eigenvalues ---    0.53765   0.55048   0.55151
```

Listing 2: Gaussian output (log) file for the k_2 transition state of molecule 50.

3.1.2 Molecule 50: k_{2cat} transition state calculation

On the first run of this mechanism, we encountered a timeout error. The last geometry from this run was fed to a second run, after which a valid transition state was successfully found. The geometry optimisation was done with B3LYP/6-31G(d), and a single point energy calculation using this optimised geometry was then performed using M06L/6-311++G**.

```
                           Standard orientation:
---------------------------------------------------------------------
Center     Atomic      Atomic             Coordinates (Angstroms)
Number     Number       Type             X           Y           Z
---------------------------------------------------------------------
      1          6           0       -1.784257   -0.438454   -1.022380
      2          6           0       -0.853465   -0.183073   -0.053209
      3          5           0        0.249293    1.942992   -0.120289
      4          8           0        0.904163    1.872557   -1.338208
      5          8           0       -0.905153    2.720781   -0.007737
      6          1           0       -1.444338    2.554569   -0.792961
      7          8           0        0.986819    1.821020    1.086304
      8          1           0        0.810683   -0.544968   -0.517330
      9          8           0        1.728791   -0.848855   -0.894110
     10          6           0       -1.407429   -0.589792    1.221180
     11          1           0        0.342124    1.854149    1.808028
     12          1           0        1.451996    1.067759   -1.377361
     13          5           0        2.754731   -0.960286    0.005690
     14          8           0        2.798087   -0.292170    1.201455
     15          1           0        2.087585    0.387560    1.251334
     16          6           0        3.981349   -1.911640   -0.364086
     17          1           0       -0.857052   -0.501863    2.159289
```

```
    18          1          0       -1.710998   -0.276296   -2.093443
    19         16          0       -3.307726   -1.144317   -0.421621
    20          6          0       -2.675009   -1.107619    1.199444
    21          1          0       -3.276624   -1.469308    2.026172
    22          1          0        4.345335   -1.703427   -1.379285
    23          1          0        3.664722   -2.964444   -0.365849
    24          1          0        4.821277   -1.811389    0.333014
---------------------------------------------------------------------

(...)

    Eigenvalues ---   -0.02430   0.00011   0.00293   0.00470   0.00624
    Eigenvalues ---    0.00793   0.01084   0.01207   0.01344   0.01456
    Eigenvalues ---    0.01576   0.01733   0.01861   0.02012   0.02349
    Eigenvalues ---    0.02422   0.02991   0.03251   0.03964   0.04507
    Eigenvalues ---    0.04891   0.05038   0.05208   0.05280   0.05880
    Eigenvalues ---    0.08745   0.09289   0.10036   0.10422   0.11289
    Eigenvalues ---    0.11387   0.11558   0.12155   0.14429   0.15067
    Eigenvalues ---    0.17220   0.18025   0.18234   0.19679   0.20043
    Eigenvalues ---    0.20574   0.23124   0.26763   0.26859   0.31069
    Eigenvalues ---    0.31180   0.31363   0.31805   0.33776   0.35089
    Eigenvalues ---    0.36047   0.36676   0.37856   0.39967   0.40498
    Eigenvalues ---    0.41741   0.43572   0.44751   0.48091   0.49354
    Eigenvalues ---    0.52208   0.54528   0.55277   0.64136   1.43518
    Eigenvalues ---    2.49993
```

Listing 3: Gaussian output (log) file for the k_{2cat} transition state of molecule 50.

3.1.3 Molecule 60: k_3 transition state calculation

A transition state for the k_3 mechanism of molecule 60 was successfully found; it has exactly 1 negative eigenvalue, and the optimised structure looks like what was reported by Cox.

```
                         Standard orientation:
```

```
 ---------------------------------------------------------------------
 Center     Atomic      Atomic             Coordinates (Angstroms)
 Number     Number       Type             X           Y           Z
 ---------------------------------------------------------------------
      1          6           0       -1.182767   -1.108638   -0.003461
      2          7           0       -2.481293   -0.768522   -0.018573
      3          7           0       -2.441941    0.578800    0.001072
      4          6           0       -1.165035    1.058934    0.022079
      5          1           0       -3.305811    1.099409   -0.011630
      6          6           0       -0.284341   -0.012717    0.024658
      7          1           0       -0.934443   -2.165092   -0.017123
      8          5           0        1.365282   -0.014943    0.155186
      9          8           0        1.885241   -0.309534    1.402203
     10          8           0        1.851334    1.389215   -0.328902
     11          1           0        1.502849    1.521811   -1.214365
     12          8           0        1.821281   -1.019550   -0.955125
     13          1           0        2.692845   -1.286322   -0.649152
     14          1           0       -0.989196    2.126435    0.033773
 ---------------------------------------------------------------------

(...)

     Eigenvalues ---   -0.00658   0.00151   0.00289   0.00730   0.01006
     Eigenvalues ---    0.01512   0.02591   0.05028   0.05904   0.06865
     Eigenvalues ---    0.09013   0.09371   0.09777   0.10214   0.11207
     Eigenvalues ---    0.11501   0.11565   0.13504   0.15410   0.17899
     Eigenvalues ---    0.22464   0.23020   0.23701   0.26159   0.27375
     Eigenvalues ---    0.35468   0.35734   0.36389   0.37014   0.37798
     Eigenvalues ---    0.39072   0.42684   0.43135   0.47492   0.49931
     Eigenvalues ---    0.55211
```

Listing 4: Gaussian output (log) file for the k_3 transition state of molecule 60.

3.1.4 Molecule 60: k_4 transition state calculation

A transition state for the k_4 mechanism of molecule 60 was successfully found; it has exactly 1 negative eigenvalue, and the optimised structure looks like what was reported by Cox.

```
                          Standard orientation:
 ---------------------------------------------------------------------
 Center     Atomic      Atomic             Coordinates (Angstroms)
 Number     Number       Type             X           Y           Z
 ---------------------------------------------------------------------
      1          6           0       -0.273729    0.000207   -0.010566
      2          6           0       -1.138411   -1.093190   -0.006407
      3          7           0       -2.414047   -0.670449    0.002664
      4          6           0       -1.138696    1.093382   -0.006449
      5          7           0       -2.414223    0.670300    0.002642
      6          1           0       -3.282519    1.188219   -0.005574
      7          1           0       -0.938598   -2.153889   -0.014453
      8          5           0        1.370719    0.000008   -0.020225
      9          1           0       -0.939371    2.154170   -0.014450
     10          8           0        1.902733   -1.163298   -0.750885
     11          8           0        1.903187    1.162724   -0.751463
     12          1           0        1.743157   -1.956583   -0.236936
     13          1           0        1.743154    1.956455   -0.238403
     14          8           0        1.755348    0.000259    1.410420
     15          1           0        2.715529    0.001401    1.455494
     16          1           0       -3.282187   -1.188640   -0.005750
 ---------------------------------------------------------------------


(...)


     Eigenvalues ---   -0.00503   0.00095   0.00174   0.00383   0.00462
     Eigenvalues ---    0.00478   0.00825   0.01552   0.01968   0.04622
     Eigenvalues ---    0.04891   0.05918   0.06623   0.08614   0.08799
     Eigenvalues ---    0.10046   0.10137   0.10797   0.11108   0.11472
```

```
    Eigenvalues ---    0.11896   0.12347   0.16051   0.16581   0.22699
    Eigenvalues ---    0.23228   0.24323   0.24599   0.25005   0.33856
    Eigenvalues ---    0.36205   0.36422   0.37433   0.37768   0.38720
    Eigenvalues ---    0.38826   0.43343   0.43401   0.44136   0.44952
    Eigenvalues ---    0.50909   0.56167
```

Listing 5: Gaussian output (log) file for the k_4 transition state of molecule 60.

3.1.5 Molecule 64: k_5 transition state calculation

A transition state for the k_5 mechanism of molecule 64 was successfully found; it has exactly 1 negative eigenvalue, and the optimised structure looks like what was reported by Cox.

```
                         Standard orientation:
---------------------------------------------------------------------
Center     Atomic      Atomic             Coordinates (Angstroms)
Number     Number       Type             X           Y           Z
---------------------------------------------------------------------
     1          6           0       -2.152375   -1.185488    0.035300
     2          6           0       -0.777146   -1.199921    0.056859
     3          6           0       -0.033655   -0.008250    0.042951
     4          6           0       -0.774412    1.186824    0.003629
     5          6           0       -2.147585    1.176698   -0.019436
     6          7           0       -2.801953   -0.004654   -0.002780
     7          1           0       -3.814980   -0.003068   -0.015914
     8          1           0       -0.263764   -2.153887    0.092546
     9          5           0        1.598158    0.016599    0.009908
    10          1           0       -0.263146    2.142932   -0.001836
    11          1           0       -2.766910    2.062960   -0.047348
    12          1           0       -2.774260   -2.070478    0.050114
    13          8           0        2.052331    1.149477    0.833348
    14          8           0        2.034887    0.283309   -1.385440
    15          1           0        2.785962    0.858249    1.375920
    16          1           0        1.766744   -0.444732   -1.951613
```

```
     17          8          0        2.074055   -1.287759    0.497099
     18          1          0        2.974093   -1.421784    0.192185
 ---------------------------------------------------------------------

(...)

     Eigenvalues ---   -0.00239   0.00123   0.00287   0.00453   0.01207
     Eigenvalues ---    0.01367   0.01493   0.01881   0.02147   0.02309
     Eigenvalues ---    0.02366   0.02641   0.02838   0.05568   0.06634
     Eigenvalues ---    0.08483   0.10443   0.10535   0.10885   0.11156
     Eigenvalues ---    0.11571   0.12207   0.12452   0.12770   0.14647
     Eigenvalues ---    0.16140   0.16908   0.18541   0.19418   0.20687
     Eigenvalues ---    0.22623   0.23744   0.25006   0.29076   0.33262
     Eigenvalues ---    0.35978   0.36220   0.36264   0.36525   0.37570
     Eigenvalues ---    0.40080   0.40219   0.43338   0.43442   0.43836
     Eigenvalues ---    0.44525   0.46691   0.49767
```

Listing 6: Gaussian output (log) file for the k_5 transition state of molecule 64.

4 Cox's Molecules: Predicted vs Measured Rate

Please see next page for the predicted vs measure rate of protodeboronation for the boronic acids included in Cox's study. Note that Cox's 50 molecules are labelled with two-digit numbers between 28 and 99 (both inclusive). The numbering of the molecules has been carried over from Cox's study for the reader's convenience, should they want to read this work as well and cross-reference the molecules.

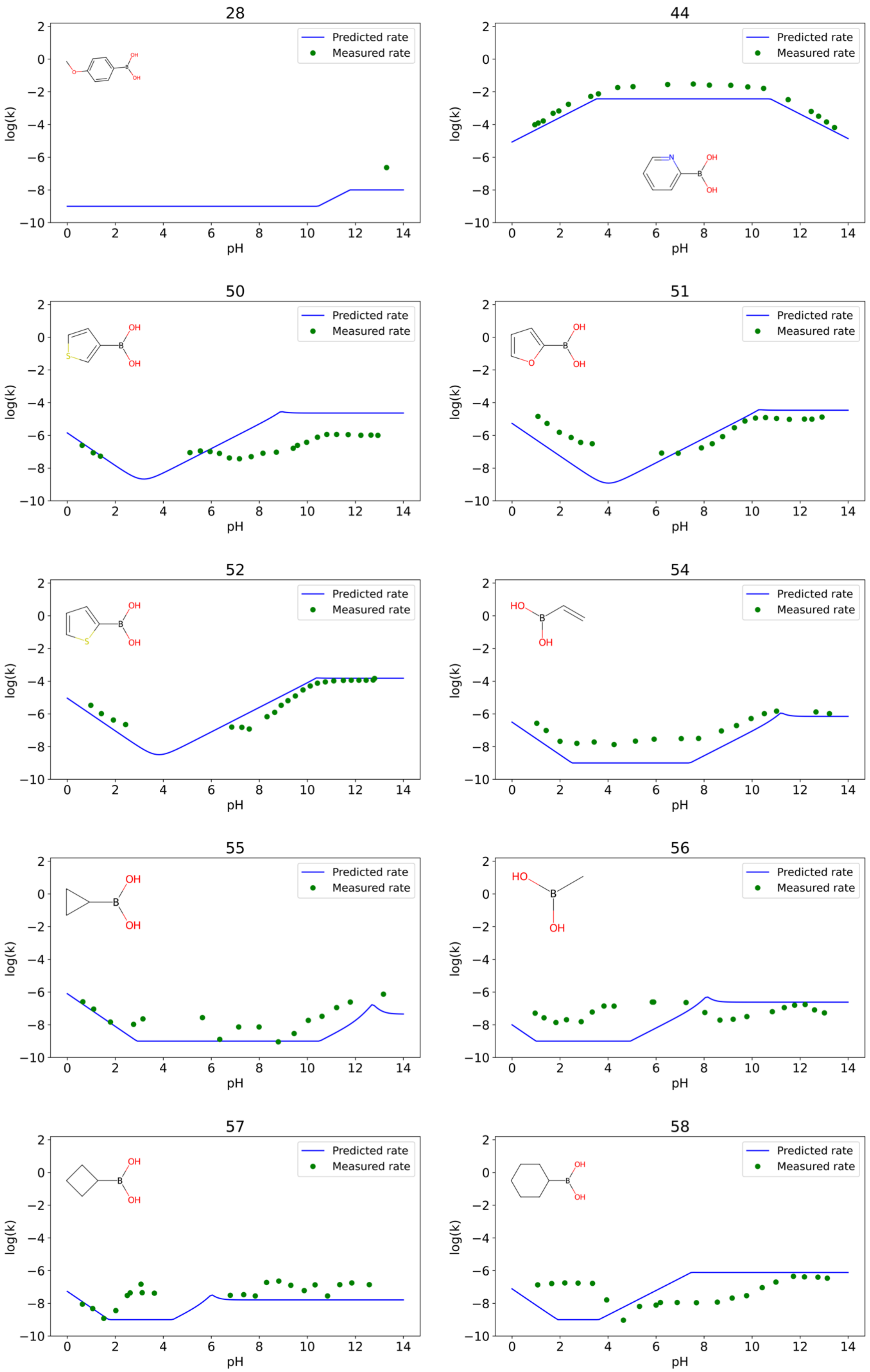
28
44
50
51
52
54
55
56
57
58
Predicted rate
Measured rate
log(k)
pH
OH
B
HO
N
S
O

60

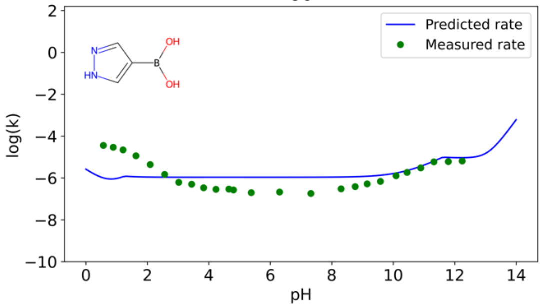

61

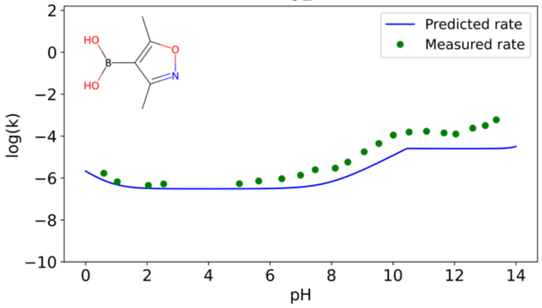

62

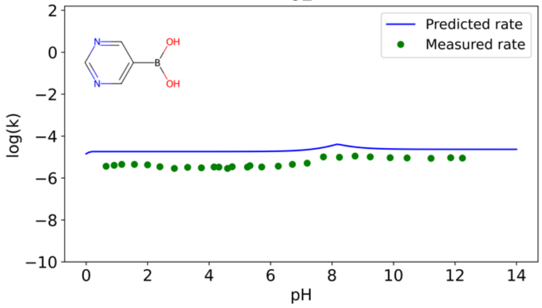

63

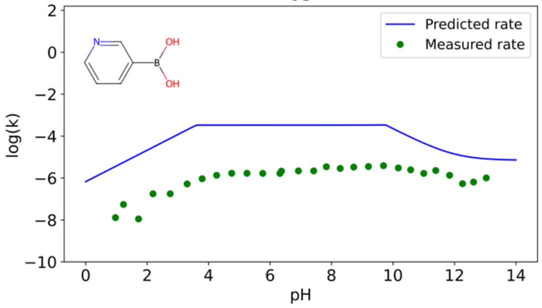

64

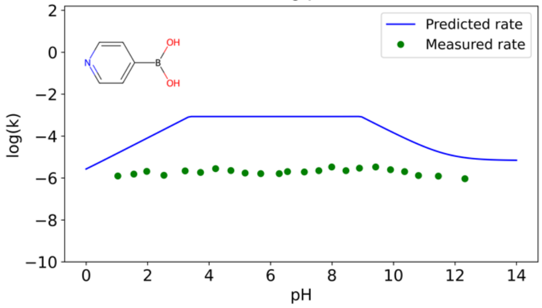

65

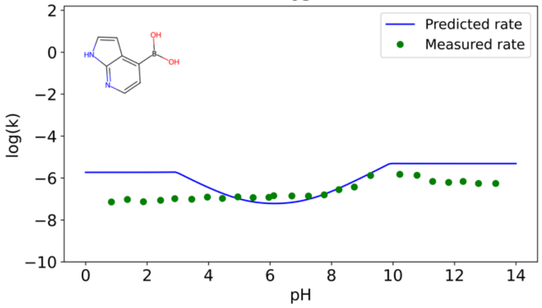

66

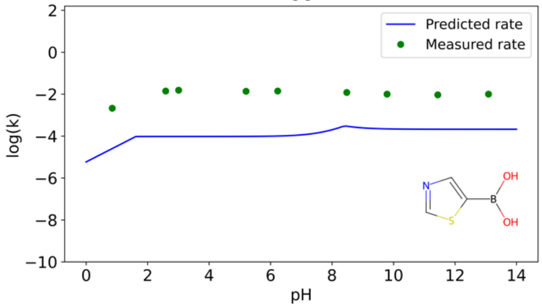

67

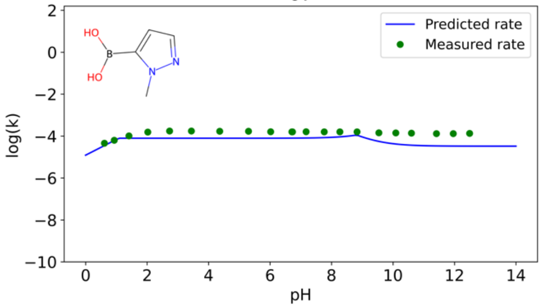

68

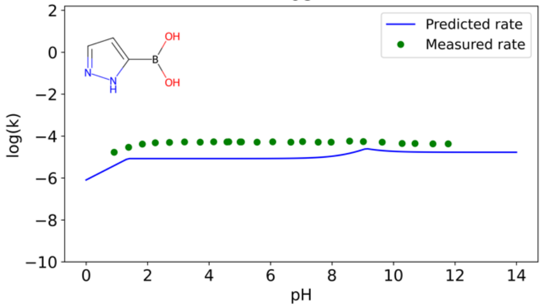

69

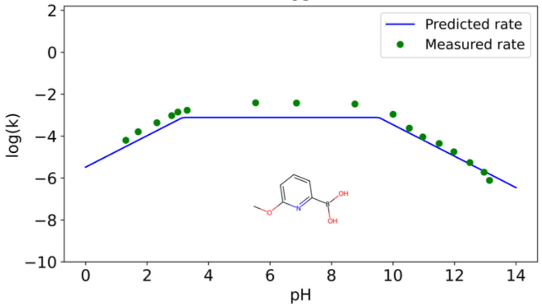

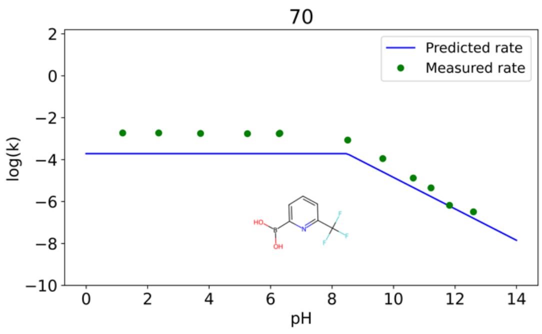
70
Predicted rate
Measured rate
log(k)
pH

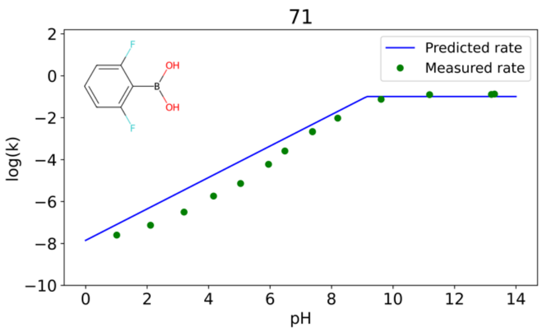
71
Predicted rate
Measured rate
log(k)
pH

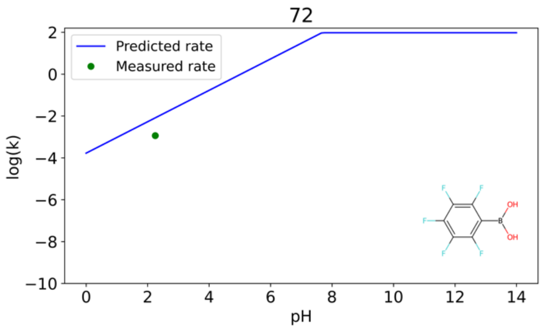
72
Predicted rate
Measured rate
log(k)
pH

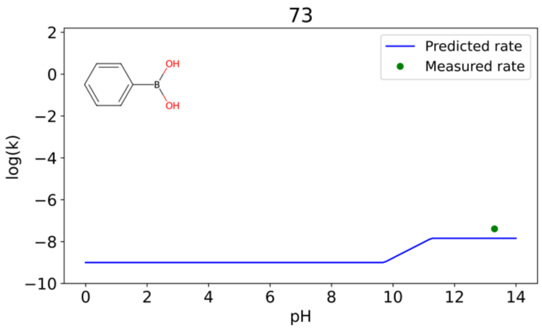
73
Predicted rate
Measured rate
log(k)
pH

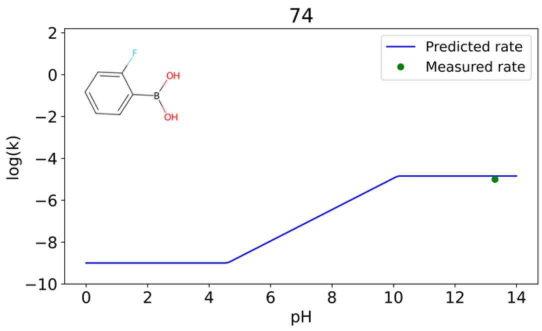
74
Predicted rate
Measured rate
log(k)
pH

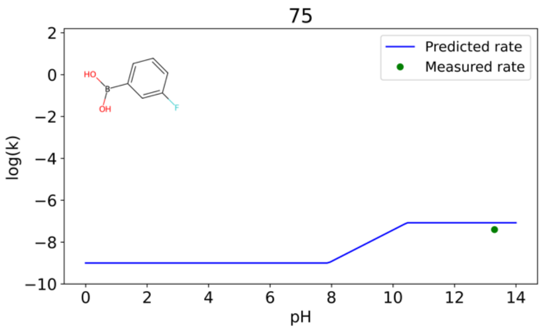
75
Predicted rate
Measured rate
log(k)
pH

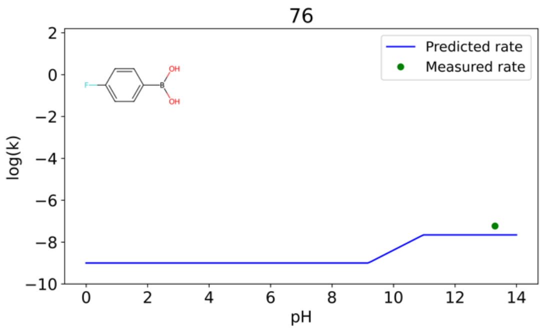
76
Predicted rate
Measured rate
log(k)
pH

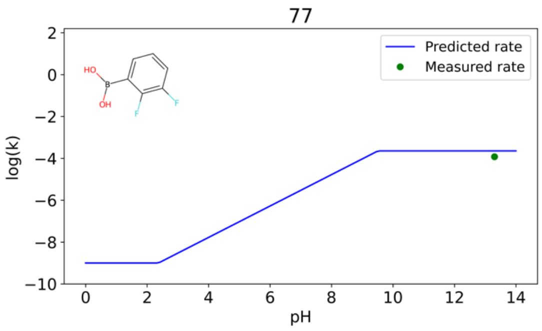
77
Predicted rate
Measured rate
log(k)
pH

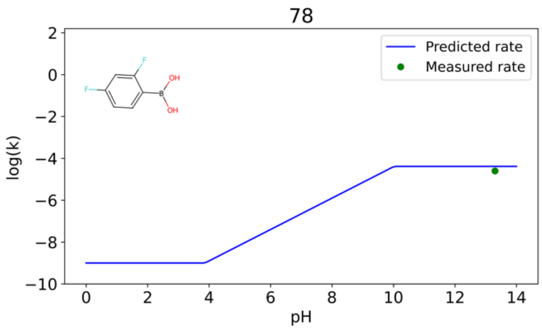
78
Predicted rate
Measured rate
log(k)
pH

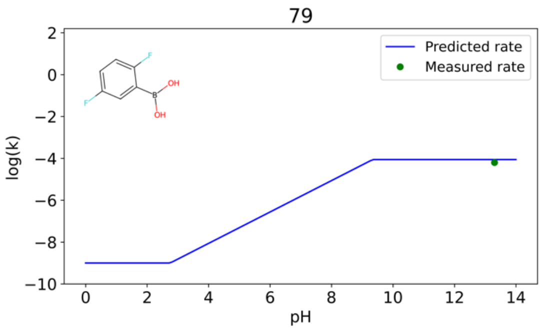
79
Predicted rate
Measured rate
log(k)
pH

80

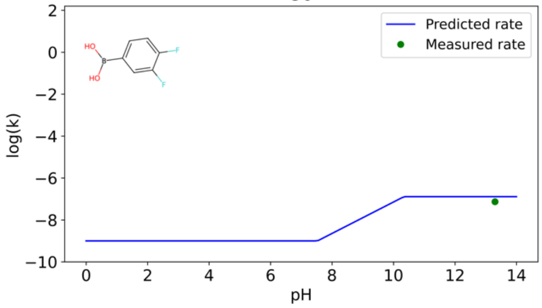

81

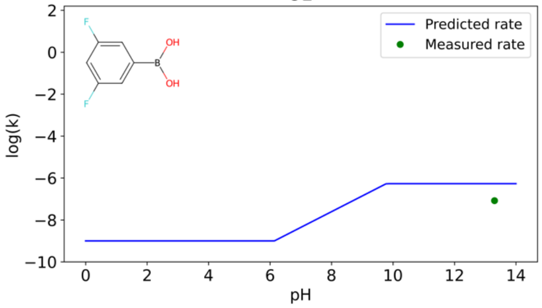

82

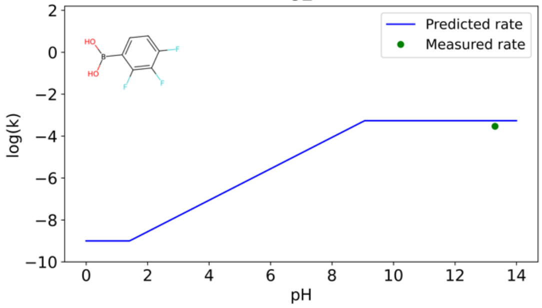

83

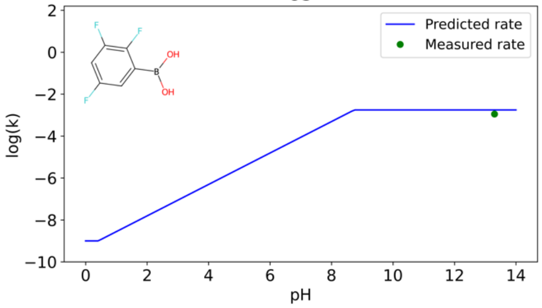

84

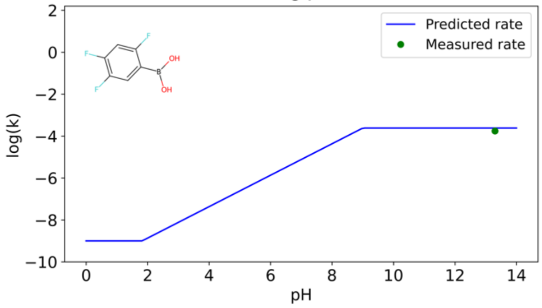

85

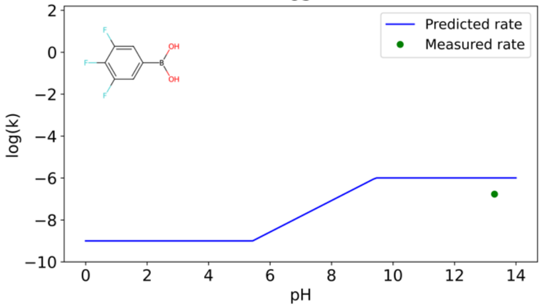

86

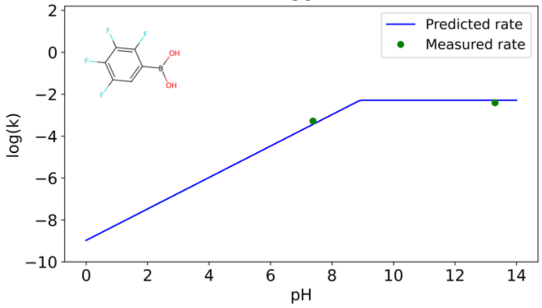

87

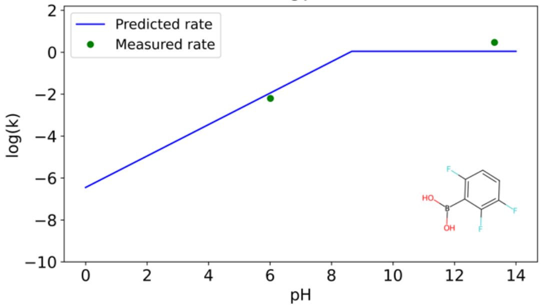

88

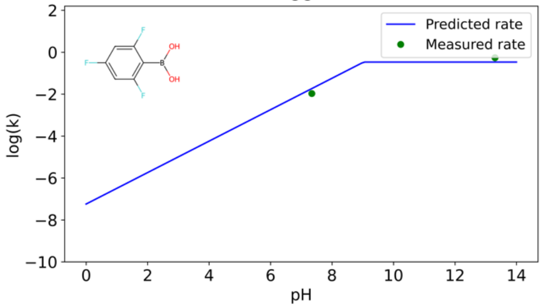

89

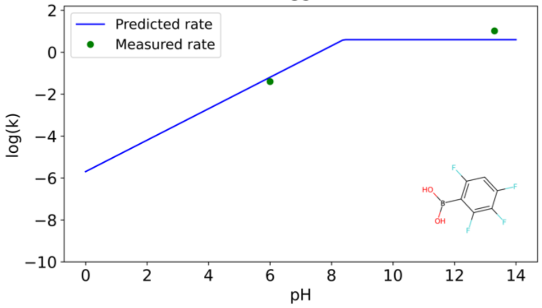

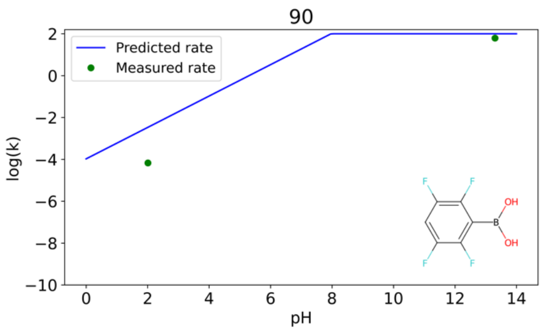
90
Predicted rate
Measured rate
log(k)
pH

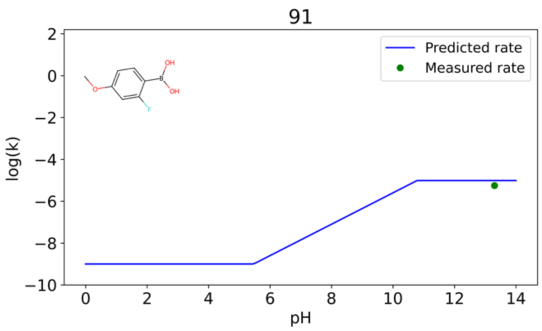
91
Predicted rate
Measured rate
log(k)
pH

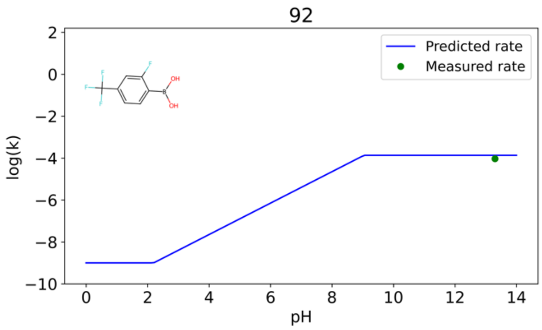
92
Predicted rate
Measured rate
log(k)
pH

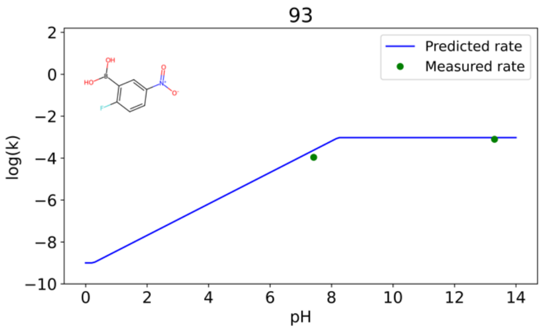
93
Predicted rate
Measured rate
log(k)
pH

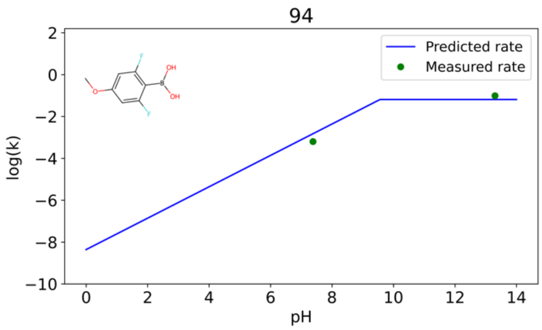
94
Predicted rate
Measured rate
log(k)
pH

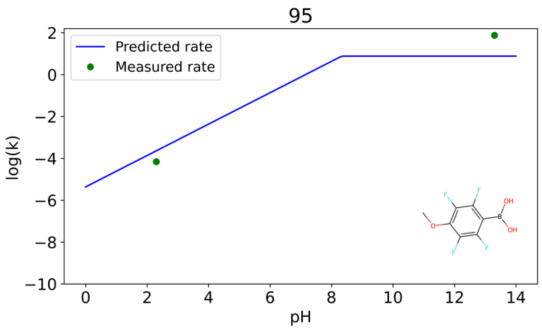
95
Predicted rate
Measured rate
log(k)
pH

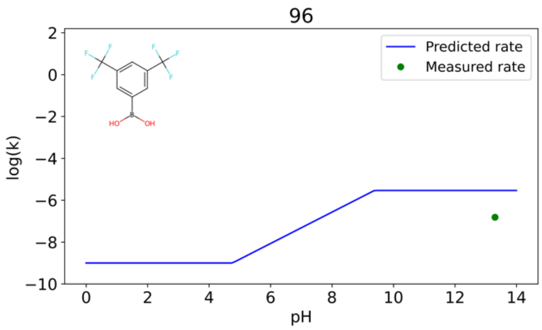
96
Predicted rate
Measured rate
log(k)
pH

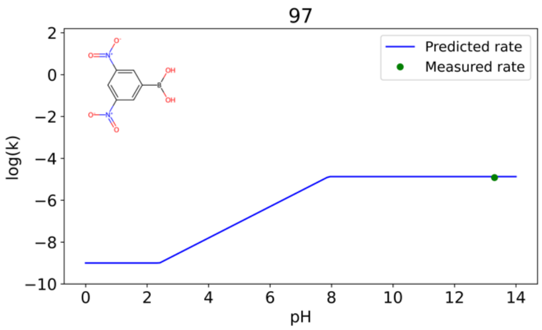
97
Predicted rate
Measured rate
log(k)
pH

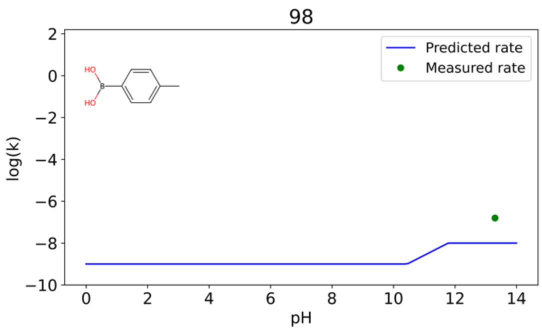
98
Predicted rate
Measured rate
log(k)
pH

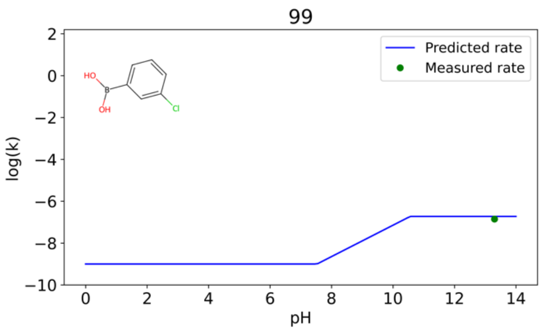
99
Predicted rate
Measured rate
log(k)
pH

5 Novel Molecules: Predicted Rate

Please see next page for the predicted rate of protodeboronation for the novel boronic acids considered in this study. Note that the 50 novel molecules are labelled with three-digit numbers running from 101 to 150.

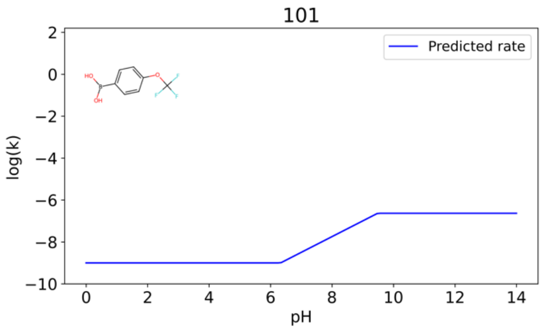
101
Predicted rate
log(k)
pH

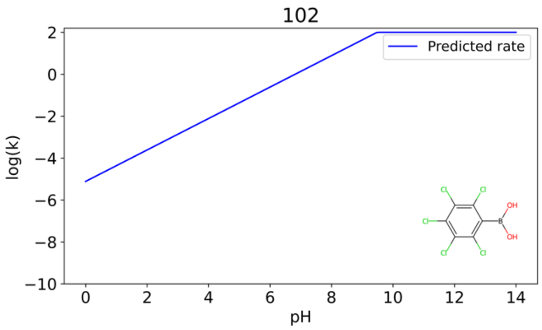
102
Predicted rate
log(k)
pH

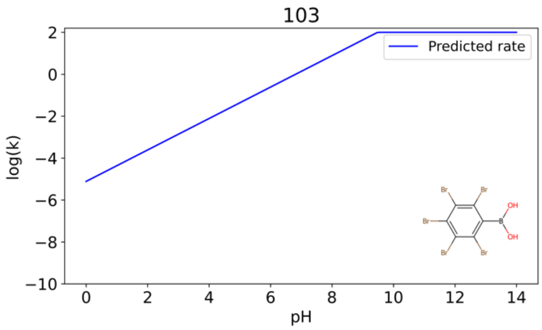
103
Predicted rate
log(k)
pH

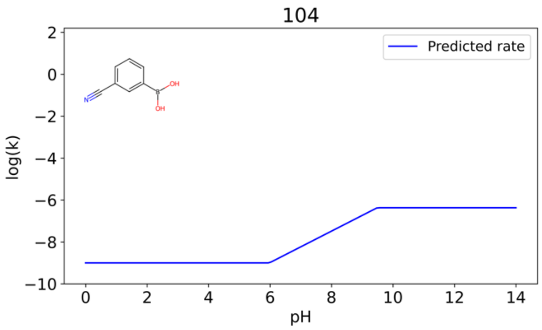
104
Predicted rate
log(k)
pH

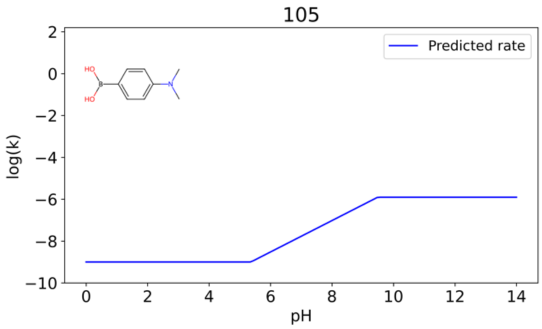
105
Predicted rate
log(k)
pH

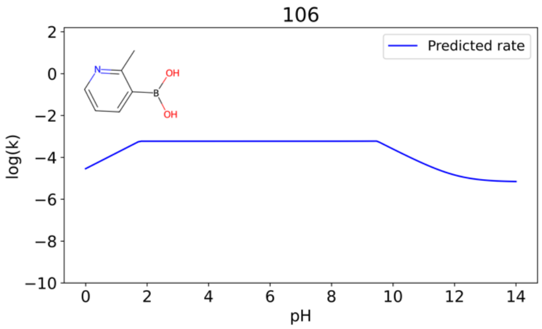
106
Predicted rate
log(k)
pH

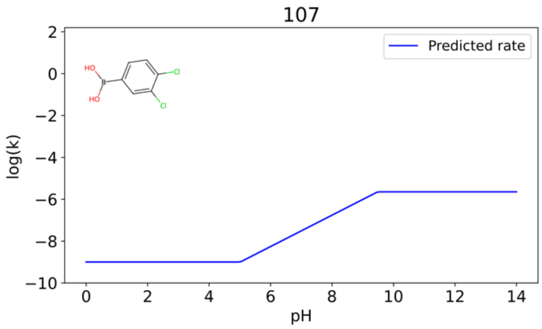
107
Predicted rate
log(k)
pH

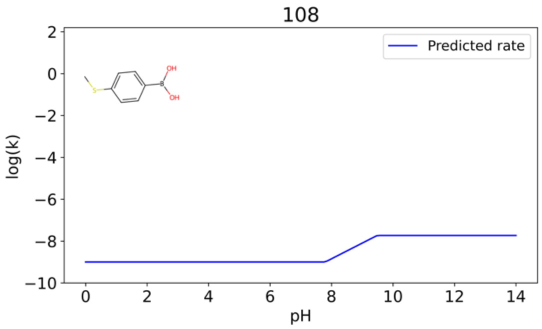
108
Predicted rate
log(k)
pH

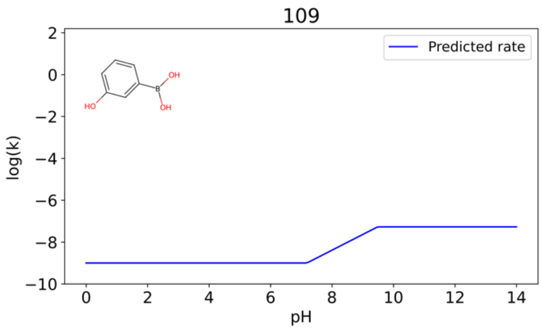
109
Predicted rate
log(k)
pH

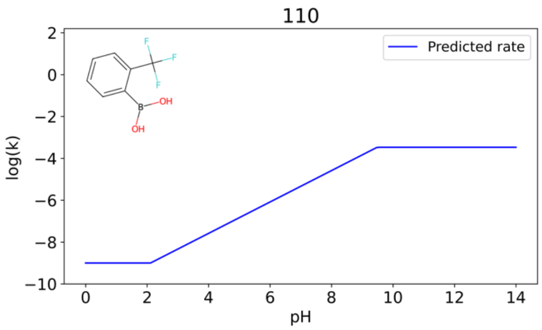
110
Predicted rate
log(k)
pH

111

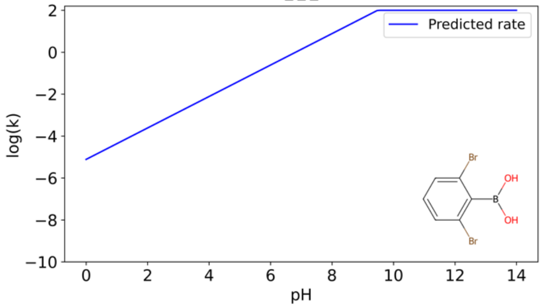

112

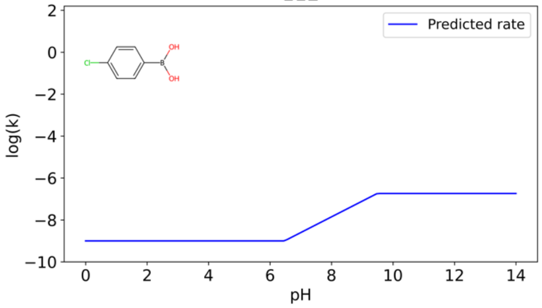

113

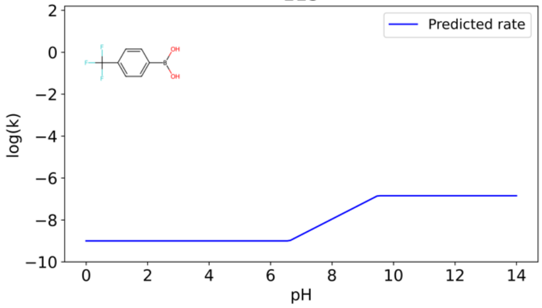

114

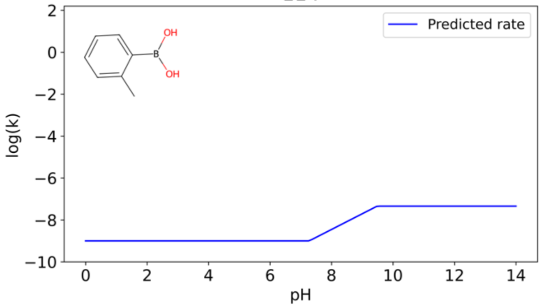

115

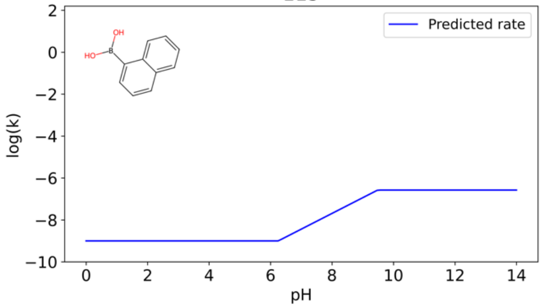

116

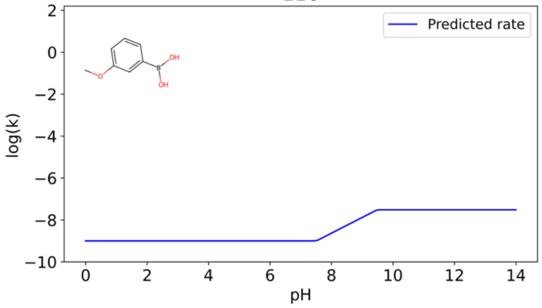

117

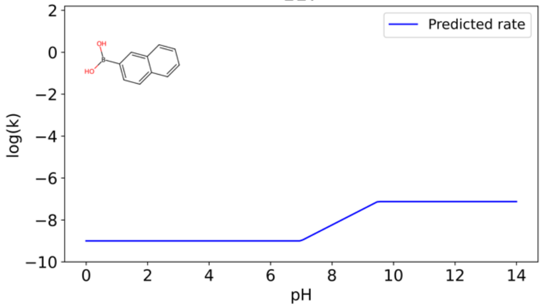

118

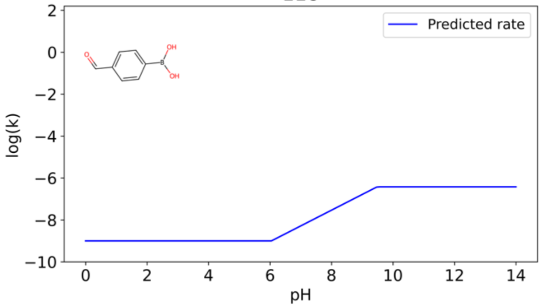

119

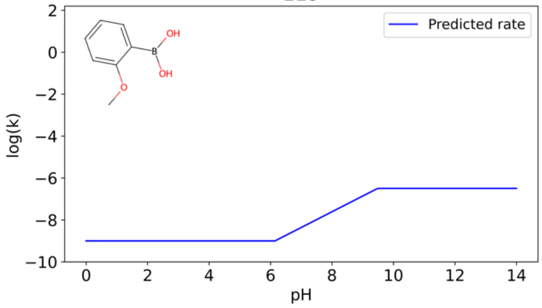

120

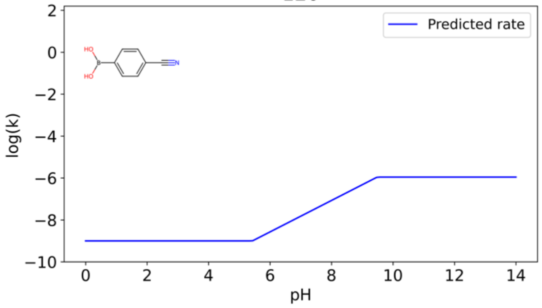

121

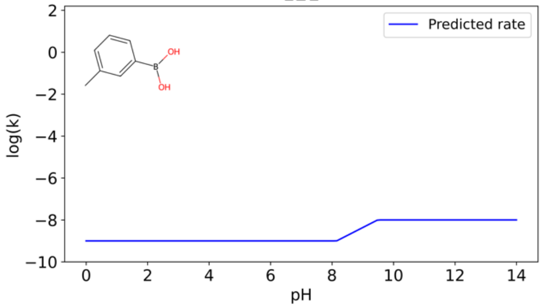

122

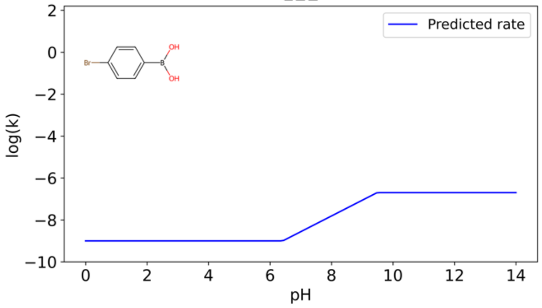

123

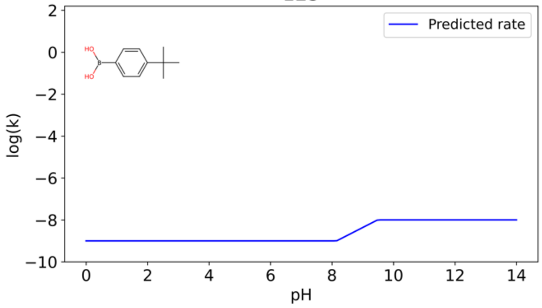

124

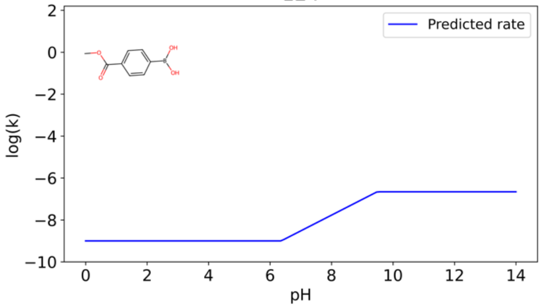

125

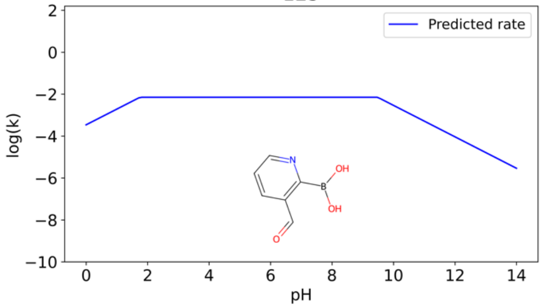

126

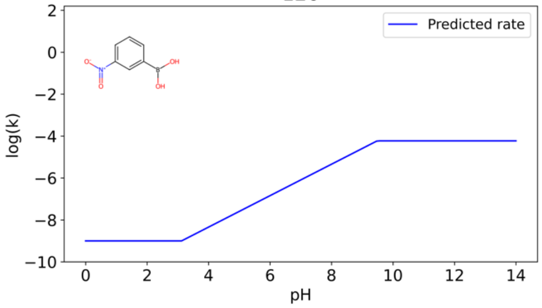

127

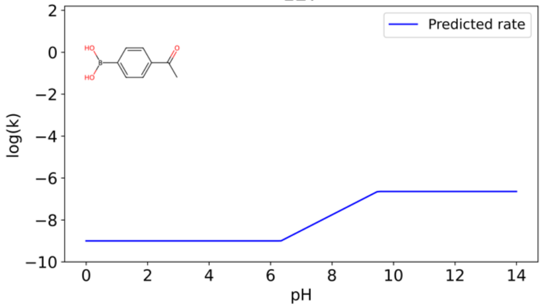

128

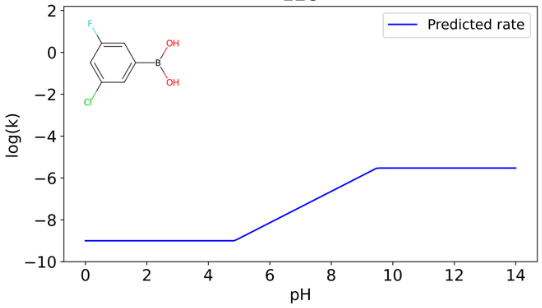

129

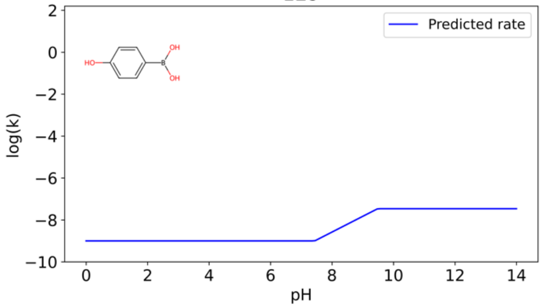

130

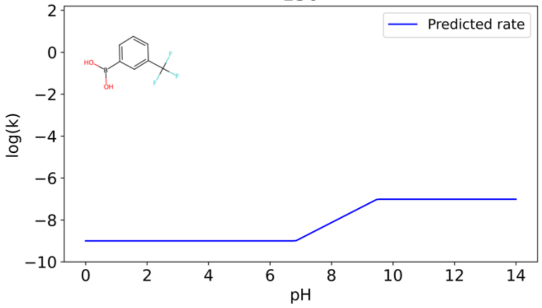

131

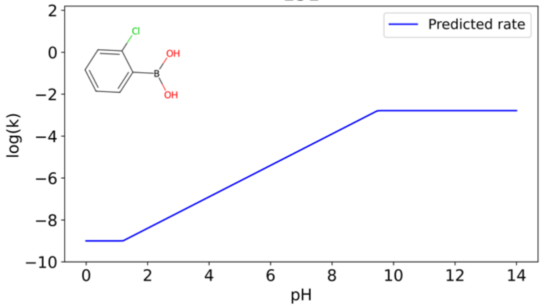

132

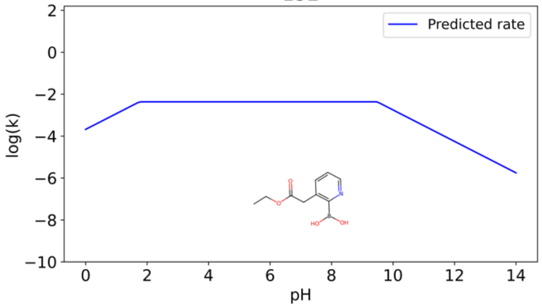

133

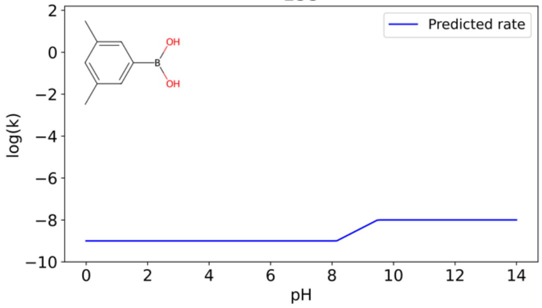

134

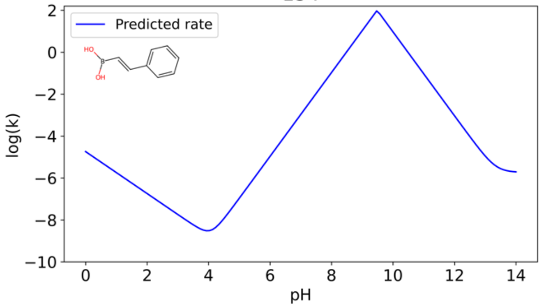

135

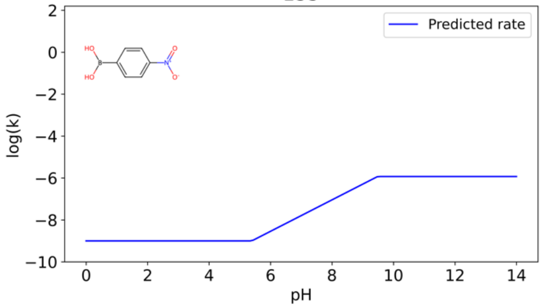

136

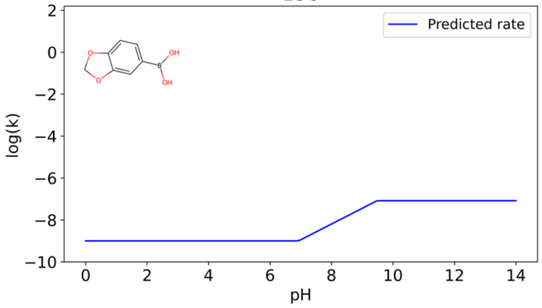

137

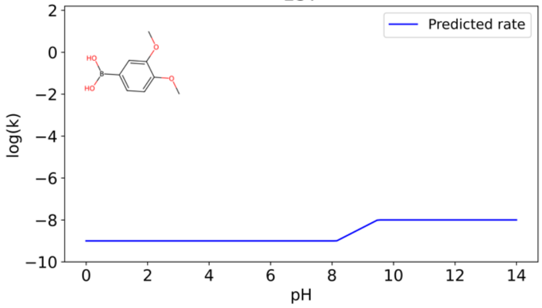

138

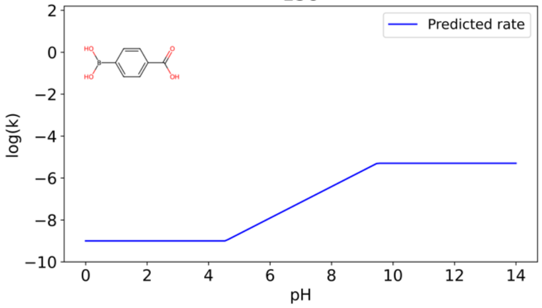

139

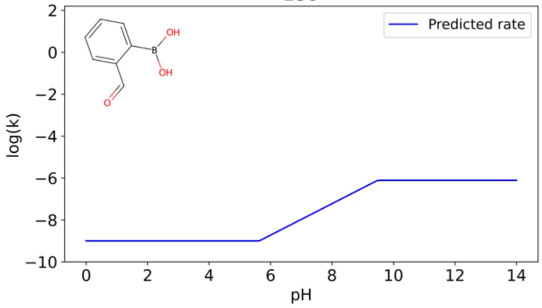

140

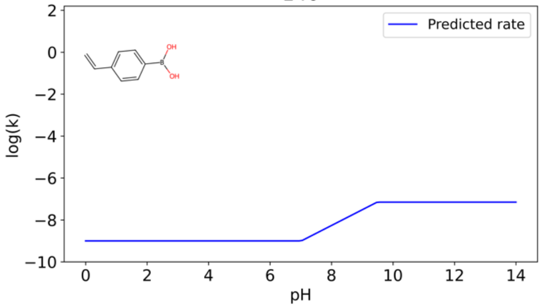

141

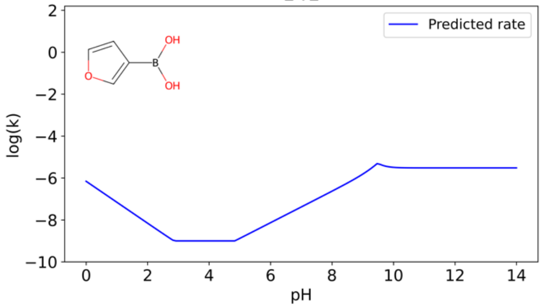

142

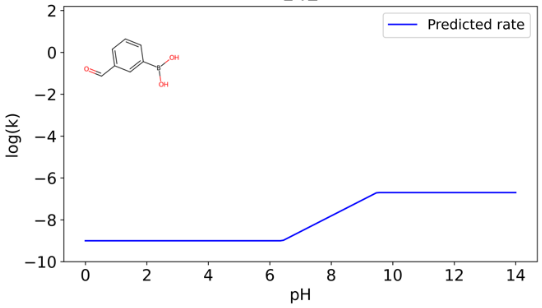

143

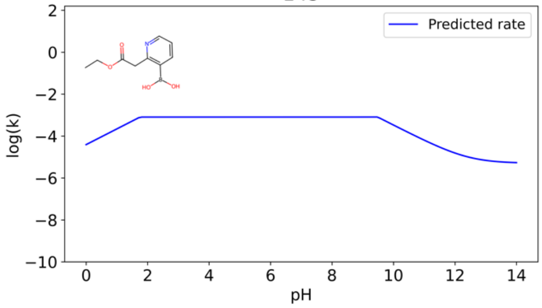

144

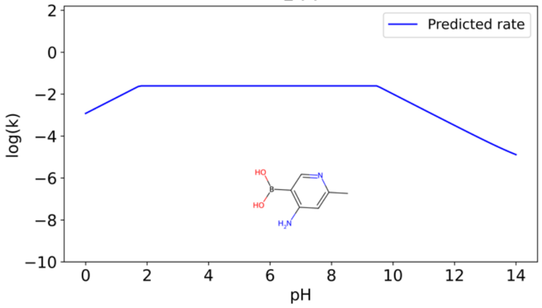

145

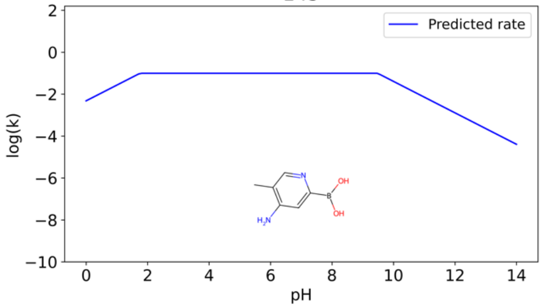

146

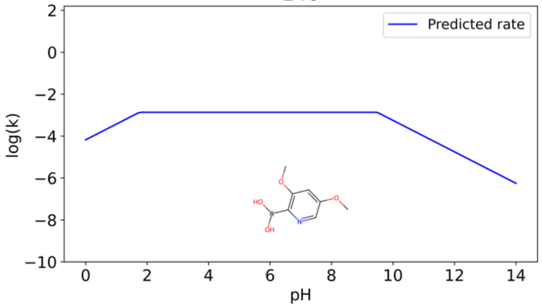

147

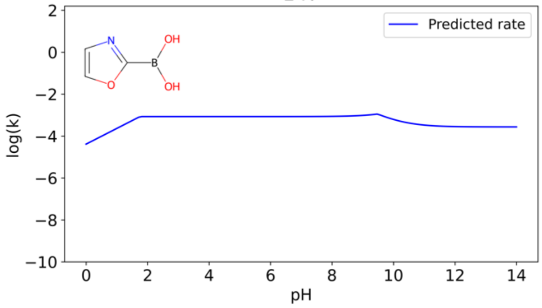

148

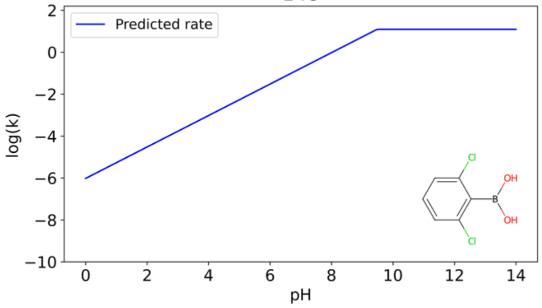

149

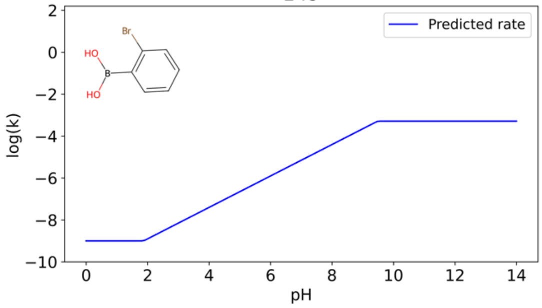

150

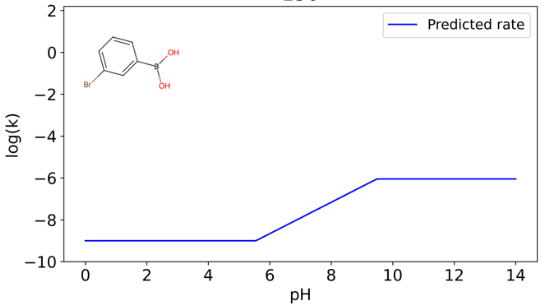

6 DFT Calculations

6.1 Optimised Geometries and Energies for Cox's Molecules

6.1.1 k_1

```
Molecule 0: k1 - Misc. small molecule
#n M06L/6-311++G** SCRF=(Solvent=Water) Opt
E(RM06L) =  -76.4487444811
Charge, multiplicity: 0, 1
Geometry:
O  0.000000    0.000000    0.11889
H -0.000000   -0.755103   -0.47558
H  0.000000    0.755103   -0.47558
```

```
Molecule 0: k1 - Misc. small molecule
#n M06L/6-311++G** SCRF=(Solvent=Water) Opt
E(RM06L) =  -76.8403699674
Charge, multiplicity: 1, 1
Geometry:
O -0.000024    0.000000   -0.08797
H -0.898204    0.206601    0.23464
H  0.628198    0.674286    0.23456
H  0.270199   -0.880887    0.23458
```

```
Molecule 50: k1 - Intermediate
#n M06L/6-311++G** SCRF=(Solvent=Water) Opt
E(RM06L) =  -729.489402778
Charge, multiplicity: 1, 1
Geometry:
C -0.234722    0.087540    0.73021
C  0.497674    1.326166    0.44187
C  1.687438    1.121577   -0.14578
S  2.024041   -0.587619   -0.33903
```

```
C  0.597730   -1.032992    0.36580
H  2.414744    1.838526   -0.49624
H  0.090598    2.306388    0.65092
B -1.694866   -0.034934   -0.01381
O -2.192723   -1.284520   -0.07094
O -2.216802    1.132796   -0.42787
H -3.094317    1.080195   -0.82110
H -3.070681   -1.368553   -0.45624
H  0.340379   -2.077331    0.48836
H -0.603574   -0.002598    1.76585
```

```
Molecule 50: k1 - Reactant
#n M06L/6-311++G** SCRF=(Solvent=Water) Opt
E(RM06L) =  -729.095858059
Charge, multiplicity: 0, 1
Geometry:
O  2.671780    1.013590    0.00024
B  1.806837   -0.050381    0.00000
O  2.351378   -1.306077   -0.00030
C  0.261119    0.083721    0.00004
C -0.491403    1.300284   -0.00020
C -1.842883    1.111123   -0.00018
S -2.240096   -0.564639    0.00008
C -0.583341   -1.004813    0.00017
H  2.238528    1.870203    0.00065
H  3.313043   -1.279972   -0.00027
H -0.051780    2.292390   -0.00033
H -2.628747    1.852397   -0.00033
H -0.309906   -2.050881    0.00036
```

```
Molecule 51: k1 - Intermediate
#n M06L/6-311++G** SCRF=(Solvent=Water) Opt
E(RM06L) =  -406.527942133
Charge, multiplicity: 1, 1
```

```
Geometry:
C  0.087069   0.205077   0.63904
C  0.853455   1.225940  -0.05434
C  2.000652   0.668569  -0.53923
C  1.973045  -0.668363  -0.11926
O  0.922167  -0.972272   0.56837
B -1.385056  -0.067414   0.01275
O -1.611568  -1.304293  -0.45961
O -2.176459   1.020675   0.06126
H -3.065297   0.914295  -0.29164
H -2.490576  -1.454926  -0.82271
H -0.065070   0.423533   1.70454
H  0.516915   2.247660  -0.16709
H  2.688644  -1.464787  -0.28466
H  2.782208   1.131072  -1.11954
```

```
Molecule 51: k1 - Reactant
#n M06L/6-311++G** SCRF=(Solvent=Water) Opt
E(RM06L) =  -406.123420416
Charge, multiplicity: 0, 1
Geometry:
O -2.315189   1.079769  -0.00012
B -1.461918   0.012612  -0.00016
O -2.063482  -1.215016   0.00034
C  0.075921   0.150507   0.00004
C  0.971656   1.186979  -0.00024
C  2.277121   0.627924   0.00075
C  2.104352  -0.722180  -0.00031
O  0.790736  -1.034156  -0.00041
H -1.870410   1.929972  -0.00084
H -1.422076  -1.931062   0.00140
H  0.723663   2.238801   0.00041
H  3.220246   1.151877   0.00067
```

```
H  2.787353   -1.556801   -0.00078
```

```
Molecule 52: k1 - Intermediate
#n M06L/6-311++G** SCRF=(Solvent=Water) Opt
E(RM06L) =  -729.501332004
Charge, multiplicity: 1, 1
Geometry:
C -0.122386   -0.083291    0.78736
C  0.674968    1.129211    0.85943
C  1.825268    1.108702    0.12254
C  2.022140   -0.135117   -0.49268
S  0.843461   -1.275329   -0.17711
B -1.517189    0.160621   -0.03136
O -1.439984    1.049877   -1.04205
O -2.655824   -0.494191    0.26388
H -2.265975    1.145821   -1.52869
H -2.621621   -1.110815    0.99995
H  2.855733   -0.396964   -1.13418
H  0.331242    1.981193    1.43514
H  2.518036    1.931850    0.01793
H -0.360320   -0.511441    1.76591
```

```
Molecule 52: k1 - Reactant
#n M06L/6-311++G** SCRF=(Solvent=Water) Opt
E(RM06L) =  -729.096488769
Charge, multiplicity: 0, 1
Geometry:
O  2.509189    1.123822    0.00049
B  1.663943    0.046709   -0.00004
O  2.316078   -1.157494   -0.00043
C  0.121670    0.202096   -0.00018
C -0.631695    1.358713   -0.00034
C -2.028022    1.143045   -0.00023
C -2.347253   -0.188905    0.00013
```

```
S -0.944655   -1.171093    0.00027
H  2.054920    1.968702    0.00113
H  1.737591   -1.922852   -0.00105
H -0.191660    2.350968   -0.00076
H -2.769717    1.932036   -0.00038
H -3.326719   -0.645239    0.00034
```

```
Molecule 54: k1 - Intermediate
#n M06L/6-311++G** SCRF=(Solvent=Water) Opt
E(RM06L) =  -255.043630145
Charge, multiplicity: 1, 1
Geometry:
C  1.324813   -0.018289    0.67966
C  1.326218   -0.015391   -0.67927
B -0.470809   -0.006267    0.00042
O -0.898263    1.248533    0.00054
O -1.160713   -1.134518   -0.00101
H -1.858179    1.359035    0.00007
H -0.701100   -1.979955   -0.00150
H  1.345303    0.915598   -1.23729
H  1.395639   -0.939174   -1.24694
H  1.343877    0.910316    1.24164
H  1.394129   -0.944526    1.24336
```

```
Molecule 54: k1 - Reactant
#n M06L/6-311++G** SCRF=(Solvent=Water) Opt
E(RM06L) =  -254.658326161
Charge, multiplicity: 0, 1
Geometry:
C -2.043336   -0.062676    0.00006
C -0.915106    0.653240   -0.00009
B  0.502971    0.018064   -0.00003
O  1.657880    0.757702    0.00007
O  0.623366   -1.344891   -0.00004
```

```
H -2.020289   -1.150029    0.00008
H -3.030435    0.392467    0.00015
H -1.007129    1.740697   -0.00017
H  1.499789    1.704900   -0.00001
H  1.543889   -1.624226    0.00010
```

```
Molecule 55: k1 - Intermediate
#n M06L/6-311++G** SCRF=(Solvent=Water) Opt
E(RM06L) =  -294.365183501
Charge, multiplicity: 1, 1
Geometry:
C -1.065787   -0.020738   -0.86615
C -2.138760    0.020620    0.06290
B  0.877174    0.002963    0.12919
O  1.378796    1.239405   -0.00321
O  1.433045   -1.208496   -0.01270
H  2.305893    1.291023   -0.25602
H  2.366225   -1.216387   -0.24947
C -0.687851   -0.040277    0.73706
H -0.652958   -0.985587    1.26640
H -0.630252    0.874977    1.31791
H -0.813100   -0.957386   -1.34822
H -2.706505   -0.877855    0.25860
H -2.637133    0.960589    0.25323
H -0.758381    0.890918   -1.36399
```

```
Molecule 55: k1 - Reactant
#n M06L/6-311++G** SCRF=(Solvent=Water) Opt
E(RM06L) =  -293.974707135
Charge, multiplicity: 0, 1
Geometry:
O  2.033299   -0.749780    0.00001
B  0.876548   -0.012452    0.00003
O  1.102165    1.342126   -0.00006
```

```
C -0.533578   -0.653457    0.00011
C -1.678508    0.015418    0.74119
C -1.678453    0.015166   -0.74128
H  1.864098   -1.694137    0.00002
H  0.298027    1.866055   -0.00000
H -0.582986   -1.739579    0.00031
H -1.471306    0.944408    1.26274
H -2.380017   -0.629811    1.25596
H -2.379916   -0.630209   -1.25592
H -1.471122    0.943996   -1.26306
```

```
Molecule 56: k1 - Intermediate
#n M06L/6-311++G** SCRF=(Solvent=Water) Opt
E(RM06L) =  -216.942416245
Charge, multiplicity: 1, 1
Geometry:
C  1.584007   -0.015894    0.00004
B -0.274278    0.007094   -0.00011
H  1.051791   -0.017797   -0.97796
H  2.173735    0.895060   -0.00026
H  2.140165   -0.948589   -0.00075
O -0.698908   -1.238804    0.00032
O -0.868538    1.178027    0.00000
H -1.656770   -1.356405   -0.00178
H -0.354105    1.991960    0.00026
H  1.052098   -0.018117    0.97817
```

```
Molecule 56: k1 - Reactant
#n M06L/6-311++G** SCRF=(Solvent=Water) Opt
E(RM06L) =  -216.577414059
Charge, multiplicity: 0, 1
Geometry:
C  1.479859    0.000156   -0.00412
B -0.085659   -0.000075   -0.01051
```

```
O -0.843925    1.143063    0.00233
O -0.843719   -1.143125    0.00221
H  1.844075   -0.002776    1.02939
H  1.909679    0.884954   -0.48010
H  1.909650   -0.882143   -0.48484
H -0.306622    1.937705   -0.01202
H -0.306488   -1.937802   -0.01146
```

```
Molecule 57: k1 - Intermediate
#n M06L/6-311++G** SCRF=(Solvent=Water) Opt
E(RM06L) =  -333.657198079
Charge, multiplicity: 1, 1
Geometry:
C  0.340580   -0.071937    0.10028
B -1.518906   -0.011920    0.00190
O -1.857760    1.263878   -0.02042
O -2.205529   -1.135437   -0.06847
H -1.745585   -1.980400   -0.02884
C  1.341681    1.082088    0.12076
C  2.423077    0.047541   -0.25328
H  1.461944    1.480413    1.12812
H  1.152185    1.903935   -0.56762
C  1.475914   -1.106433    0.13244
H  3.360281    0.105220    0.29800
H  2.637744    0.054354   -1.32273
H  1.641253   -1.473133    1.14509
H  1.388345   -1.953356   -0.54595
H -0.162560   -0.126022   -0.89923
H -2.800329    1.443345   -0.11918
H -0.319953   -0.129834    1.01281
```

```
Molecule 57: k1 - Reactant
#n M06L/6-311++G** SCRF=(Solvent=Water) Opt
E(RM06L) =  -333.285822864
```

```
Charge, multiplicity: 0, 1
Geometry:
O   1.970054    1.143049   -0.12035
B   1.244180   -0.004168    0.08906
O   1.904269   -1.195227   -0.05604
C  -0.270391   -0.015053    0.47560
C  -1.244165    1.061446   -0.06845
C  -2.335331   -0.017962   -0.12954
C  -1.229366   -1.083333   -0.10448
H   1.446482    1.941406   -0.01317
H   2.831217   -1.068984   -0.28281
H  -0.355853   -0.033843    1.57159
H  -0.954377    1.393189   -1.07174
H  -1.429413    1.948891    0.54298
H  -2.920069   -0.036203    0.79485
H  -3.027351   -0.006701   -0.97577
H  -1.399951   -1.992879    0.47714
H  -0.930658   -1.377194   -1.11598
```

```
Molecule 58: k1 - Intermediate
#n M06L/6-311++G** SCRF=(Solvent=Water) Opt
E(RM06L) =  -412.324268008
Charge, multiplicity: 1, 1
Geometry:
C   0.216890    0.051476    0.38740
B   2.008528    0.005233   -0.10039
O   2.453959   -1.238956   -0.08148
O   2.415565    1.239325   -0.34083
H   3.334733    1.354190   -0.60794
C  -0.600476   -1.189592    0.71814
C  -2.045448    1.111085    0.19014
C  -0.555263    1.375963    0.42632
H  -0.390412    1.860218    1.39092
```

```
H -0.156023    2.050022   -0.33477
H  0.561269   -0.097538   -0.68197
H  3.381590   -1.376514   -0.30533
C -1.747038   -1.349490   -0.28988
H  0.035201   -2.075176    0.75309
H -0.990446   -1.041633    1.72905
C -2.236500    0.000742   -0.83497
H -2.565938   -1.875129    0.20792
H -1.427539   -1.991878   -1.11534
H -3.282866   -0.074912   -1.13809
H -1.682955    0.262730   -1.74626
H -2.523957    2.037460   -0.13404
H -2.529038    0.828991    1.13225
H  1.044563    0.108962    1.14808
```

```
Molecule 58: k1 - Reactant
#n M06L/6-311++G** SCRF=(Solvent=Water) Opt
E(RM06L) =  -411.946971403
Charge, multiplicity: 0, 1
Geometry:
O -2.616914   -1.163642    0.01974
B -1.863113   -0.023748   -0.09301
O -2.616674    1.122254   -0.07542
C -0.297813   -0.026194   -0.26762
C  0.366786    1.317641    0.10555
C  1.869806    1.173791    0.38392
C  2.468059    0.064115   -0.46904
C  1.886009   -1.296774   -0.06398
C  0.439069   -1.172898    0.43590
H -2.078159   -1.958578    0.01441
H -2.078975    1.915261   -0.14232
H -0.142493   -0.179702   -1.35044
H  0.212957    2.036662   -0.70862
```

```
H -0.120772    1.749890    0.98928
H  2.379773    2.126217    0.21237
H  2.031185    0.932630    1.44236
H  3.559088    0.050605   -0.39092
H  2.245219    0.269629   -1.52400
H  2.504384   -1.749683    0.71888
H  1.926211   -1.983045   -0.91691
H -0.073778   -2.132460    0.30046
H  0.428133   -0.985669    1.51764
```

```
Molecule 60: k1 - Intermediate
#n M06L/6-311++G** SCRF=(Solvent=Water) Opt
E(RM06L) =  -402.706590951
Charge, multiplicity: 1, 1
Geometry:
C -0.089276    0.047767    0.65622
C -0.916237    1.165669    0.17176
N -2.007946    0.780120   -0.40516
N -1.980859   -0.611329   -0.28959
C -0.937769   -1.081946    0.31902
H -2.752070   -1.141388   -0.68302
H -0.671944    2.217333    0.22880
B  1.409994   -0.022750    0.02356
H -0.785238   -2.139557    0.48027
O  2.001593    1.175437   -0.13308
O  1.863854   -1.265832   -0.22530
H  2.904746    1.159624   -0.46592
H  2.764758   -1.326153   -0.55904
H  0.147536    0.096582    1.72945
```

```
Molecule 60: k1 - Reactant
#n M06L/6-311++G** SCRF=(Solvent=Water) Opt
E(RM06L) =  -402.305197822
Charge, multiplicity: 0, 1
```

```
Geometry:
O  2.248202  -1.131228   0.04472
B  1.490065   0.010486   0.00034
O  2.245278   1.154216  -0.04314
C -0.056007  -0.000360  -0.00064
C -0.939050  -1.109593  -0.04063
N -2.220899  -0.758971  -0.02797
N -2.180552   0.589865   0.02189
C -0.929208   1.081786   0.03902
H  1.728904  -1.936059   0.09186
H  1.724878   1.957966  -0.09392
H -0.692337  -2.162959  -0.08216
H -3.045622   1.109741   0.04478
H -0.758241   2.147714   0.08127
```

```
Molecule 61: k1 - Intermediate
#n M06L/6-311++G** SCRF=(Solvent=Water) Opt
E(RM06L) =  -501.195539036
Charge, multiplicity: 1, 1
Geometry:
O  1.566958  -1.162343  -0.42686
C  1.353330  -0.112653   0.27273
C -0.056507   0.000306   0.61830
C -0.576068  -1.272102   0.04604
N  0.321882  -1.943142  -0.58815
C -1.964465  -1.772433   0.11399
H -2.042348  -2.757085  -0.34161
H -2.638342  -1.088044  -0.40723
H -2.291781  -1.827110   1.15444
C  2.462767   0.783205   0.57111
H  2.472594   1.006920   1.64029
H  2.279838   1.732055   0.05639
H  3.413656   0.364598   0.25349
```

```
B -0.767315    1.311710   -0.05224
O -2.003425    1.707101    0.29669
O -0.040855    1.929251   -1.00217
H -0.473071    2.699977   -1.38757
H -2.421110    1.258442    1.03685
H -0.211796    0.103688    1.69877
```

```
Molecule 61: k1 - Reactant
#n M06L/6-311++G** SCRF=(Solvent=Water) Opt
E(RM06L) =  -500.799246976
Charge, multiplicity: 0, 1
Geometry:
O -2.142303   -0.396145    0.00045
C -0.929882   -0.974757    0.00015
C  0.055454   -0.020180    0.00019
C -0.686925    1.205286   -0.00012
N -1.985116    1.003197   -0.00003
C -0.174036    2.599337   -0.00020
H -0.999682    3.311397   -0.00052
H  0.451655    2.782343   -0.87613
H  0.451228    2.782582    0.87597
C -0.924304   -2.451365   -0.00031
H  0.097375   -2.822769    0.01129
H -1.432939   -2.840059   -0.88632
H -1.454505   -2.840756    0.87251
B  1.588267   -0.210845    0.00004
O  2.360690    0.920316   -0.00001
O  2.087186   -1.487343    0.00009
H  3.046542   -1.546399    0.00084
H  3.308379    0.760953   -0.00026
```

6.1.2 k_2

```
Molecule 0: k2 - Misc. small molecule
#n M06L/6-311++G** SCRF=(Solvent=Water) Opt
E(RM06L) =  -76.4487444811
Charge, multiplicity: 0, 1
Geometry:
O  0.000000   0.000000   0.11889
H -0.000000  -0.755103  -0.47558
H  0.000000   0.755103  -0.47558
```

```
Molecule 50: k2 - Transition State
#n M06L/6-311++G** SCRF=(Solvent=Water) Opt=(TS,CalcFC,noeigentest)
E(RM06L) =  -881.496906118
Charge, multiplicity: -1, 1
Geometry:
C -1.089307  -0.350945   1.24018
C -0.314862   0.327646   0.23629
B  1.829662  -0.707096  -0.22508
O  2.464284  -0.503787   0.99219
O  1.322779  -1.973570  -0.50297
H  1.051171  -2.409264   0.30823
O  2.222152   0.075134  -1.31504
H  2.250068   2.160591  -0.24601
H  1.045518   1.584497   0.48723
O  1.807481   2.237042   0.60616
C -1.161841   0.670958  -0.78368
H  1.679631  -0.123479  -2.08198
H  2.566170   0.450184   1.12882
S -2.845137   0.181130  -0.54703
C -2.427060  -0.508585   0.97554
H -3.190312  -0.984489   1.57954
H -0.660624  -0.741372   2.16355
H -0.942896   1.187730  -1.71412
```

```
Molecule 50: k2 - Intermediate
```

```
#n M06L/6-311++G** SCRF=(Solvent=Water) Opt
E(RM06L) =  -805.070607843
Charge, multiplicity: -1, 1
Geometry:
O  2.202714  -0.872377   1.02035
B  1.649908   0.065163  -0.00958
O  2.233669  -0.329870  -1.30768
O  2.109251   1.459284   0.17887
C  0.017038  -0.039948   0.00491
C -0.719020  -1.269817   0.00647
C -2.079442  -1.127189  -0.00826
S -2.528006   0.535991  -0.02131
C -0.859820   1.015554  -0.00951
H  1.605429  -0.921556   1.76884
H  2.123450  -1.277301  -1.40403
H  1.666354   1.835008   0.94110
H -0.241999  -2.246256   0.01921
H -2.843106  -1.893098  -0.00906
H -0.629176   2.073627  -0.02114
```

```
Molecule 51: k2 - Transition State
#n M06L/6-311++G** SCRF=(Solvent=Water) Opt=(TS,CalcFC,noeigentest)
E(RM06L) =  -558.528241782
Charge, multiplicity: -1, 1
Geometry:
C -0.605347   0.462986   0.29513
B  1.397771  -0.786459   0.02240
O  2.236615  -0.303020   1.01658
O  0.822408  -2.043570   0.20077
H  0.706464  -2.227641   1.13571
O  1.611763  -0.365505  -1.29437
H  1.934505   1.963760  -0.85762
H  0.804844   1.685601   0.12461
```

```
O  1.605409    2.284191   -0.01109
H  0.826728   -0.551118   -1.81863
H  2.381496    0.642060    0.85745
O -1.195415    0.154158   -0.95784
C -2.519041   -0.108391   -0.79163
C -2.858509    0.019931    0.51837
C -1.651214    0.381929    1.19454
H -1.558095    0.558333    2.25995
H -3.080322   -0.361530   -1.67991
H -3.846042   -0.125930    0.93555
```

```
Molecule 51: k2 - Intermediate
#n M06L/6-311++G** SCRF=(Solvent=Water) Opt
E(RM06L) =  -482.100609764
Charge, multiplicity: -1, 1
Geometry:
O  1.733581   -0.754070   -1.19102
B  1.303869    0.014238    0.01830
O  1.974124    1.317706   -0.01265
O  1.760913   -0.618288    1.28050
C -0.314540    0.160233    0.01064
C -1.210456    1.193997    0.03364
C -2.530979    0.644515    0.01368
C -2.374173   -0.703225   -0.01994
O -1.048484   -1.019653   -0.02362
H  1.097435   -1.447673   -1.37687
H  1.894500    1.674056   -0.89877
H  1.189582   -1.359405    1.48803
H -0.948209    2.242336    0.06256
H -3.471778    1.175767    0.02315
H -3.061062   -1.534954   -0.04337
```

```
Molecule 52: k2 - Transition State
#n M06L/6-311++G** SCRF=(Solvent=Water) Opt=(TS,CalcFC,noeigentest)
```

```
E(RM06L) =  -881.508069691
Charge, multiplicity: -1, 1
Geometry:
C -0.420843   0.254682   0.32075
B  1.693503  -0.727599  -0.18988
O  2.296650  -0.583212   1.05263
O  1.208524  -1.980671  -0.55398
H  0.884851  -2.448810   0.21911
O  2.116859   0.109330  -1.22810
H  2.145658   2.106436  -0.11215
H  0.917130   1.574294   0.59568
O  1.684430   2.204736   0.72835
H  1.628956  -0.077947  -2.03360
H  2.406910   0.361136   1.23552
S -1.492618   0.575705  -1.02015
C -2.922040  -0.042152  -0.25829
C -2.602910  -0.483685   0.99842
C -1.219962  -0.312022   1.30366
H -0.804794  -0.628367   2.26086
H -3.887118  -0.047049  -0.74855
H -3.334390  -0.915378   1.67671
```

```
Molecule 52: k2 - Intermediate
#n M06L/6-311++G** SCRF=(Solvent=Water) Opt
E(RM06L) =  -805.076415003
Charge, multiplicity: -1, 1
Geometry:
O  1.906940  -1.104631  -0.83732
B  1.514235   0.063077   0.00630
O  2.157698   1.330122  -0.39454
O  2.033939  -0.203264   1.35900
C -0.115404   0.236477  -0.02452
C -0.906335   1.362543   0.01305
```

```
C -2.307533    1.117015    0.04421
C -2.605563   -0.217456    0.03401
S -1.159571   -1.158706   -0.02292
H  1.410872   -1.096724   -1.65828
H  1.698584    1.695606   -1.15209
H  1.814032   -1.107491    1.58972
H -0.479342    2.361464    0.02180
H -3.064849    1.893647    0.07056
H -3.576958   -0.691886    0.04596
```

```
Molecule 54: k2 - Transition State
#n M06L/6-311++G** SCRF=(Solvent=Water) Opt=(TS,CalcFC,noeigentest)
E(RM06L) =  -407.051563487
Charge, multiplicity: -1, 1
Geometry:
C -1.351190   -0.473556   -0.70077
B  0.988623   -0.289788    0.11473
O  0.711271    0.201936    1.37928
O  1.255298   -1.644534   -0.04804
H  0.761954   -2.156588    0.59695
O  1.542915    0.580068   -0.82551
H  0.045514    2.401866   -0.47025
H -0.944649    1.232132   -0.30242
O -0.758011    2.191576    0.01625
H  1.680534    0.131088   -1.66272
H  0.286146    1.068247    1.27568
C -2.305861   -0.956695    0.11675
H -1.388930   -0.957202   -1.69873
H -3.047460   -1.730886   -0.13121
H -2.405706   -0.590570    1.14730
```

```
Molecule 54: k2 - Intermediate
#n M06L/6-311++G** SCRF=(Solvent=Water) Opt
E(RM06L) =  -330.633661196
```

```
Charge, multiplicity: -1, 1
Geometry:
C  2.258389   0.013049  -0.03606
C  1.081585  -0.622778  -0.06726
B -0.404249   0.010028  -0.01448
O -1.242433  -0.428995  -1.16393
O -0.309643   1.479921  -0.05371
O -1.126372  -0.479663   1.20893
H  2.305887   1.100099   0.02462
H  3.221881  -0.496324  -0.06928
H  1.136080  -1.718597  -0.12816
H -1.475358  -1.350829  -1.03526
H -1.204671   1.824166  -0.00764
H -0.574836  -0.320373   1.97785
```

```
Molecule 55: k2 - Transition State
#n M06L/6-311++G** SCRF=(Solvent=Water) Opt=(TS,CalcFC,noeigentest)
E(RM06L) =  -446.360598309
Charge, multiplicity: -1, 1
Geometry:
C  1.111402  -0.129008  -0.62870
B -1.264517  -0.403725   0.00953
O -1.934977   0.498596  -0.79577
O -1.090415  -0.063131   1.35864
H -0.248296   2.219137   0.94949
H  0.381122   1.412618  -0.22337
O -0.005901   2.347774   0.02742
H -0.415363  -0.607611   1.77164
H -1.592929   1.382144  -0.58178
C  2.549175   0.157804  -0.21781
C  1.831079  -1.016451   0.36784
H  1.091647  -0.612825  -1.61446
H  2.192156  -2.008134   0.09265
```

```
H  1.551350   -0.965673    1.42513
H  2.745752    1.017286    0.42557
H  3.383255   -0.058161   -0.88669
O -1.323547   -1.722577   -0.42881
H -0.877326   -2.319526    0.17433
```

```
Molecule 55: k2 - Intermediate
#n M06L/6-311++G** SCRF=(Solvent=Water) Opt
E(RM06L) =  -369.948332323
Charge, multiplicity: -1, 1
Geometry:
O -0.745121    0.089822    1.45308
B -0.718344   -0.024694   -0.02891
O -1.379067   -1.301477   -0.39731
O -1.493742    1.162461   -0.52870
C  0.776904   -0.051358   -0.64264
C  1.881917    0.742216    0.00302
C  1.893400   -0.756124    0.08135
H -0.606745    1.011232    1.68102
H -2.274990   -1.271142   -0.05224
H -1.344060    1.256660   -1.47149
H  0.861224   -0.106739   -1.73093
H  1.643095    1.292329    0.91031
H  2.635495    1.221624   -0.61507
H  1.650916   -1.210312    1.03815
H  2.656900   -1.285028   -0.48207
```

```
Molecule 56: k2 - Transition State
#n M06L/6-311++G** SCRF=(Solvent=Water) Opt=(TS,CalcFC,noeigentest)
E(RM06L) =  -368.964719881
Charge, multiplicity: -1, 1
Geometry:
C -0.445249    1.838651    0.02967
B  0.718126   -0.337907   -0.01806
```

```
O  0.232891   -0.891534   -1.19612
O  1.984295    0.240360   -0.02655
H  2.178513    0.603784   -0.89316
O  0.284925   -0.888659    1.19325
H -2.032901   -0.867036    0.67767
H -1.596537    0.435623   -0.02301
O -2.289069   -0.323899   -0.07453
H  0.654901   -0.401177    1.93274
H -0.725463   -1.003105   -1.09793
H  0.172399    2.203635   -0.80807
H  0.086650    2.136191    0.94899
H -1.361020    2.459575    0.00669
```

```
Molecule 56: k2 - Intermediate
#n M06L/6-311++G** SCRF=(Solvent=Water) Opt
E(RM06L) =  -292.550342301
Charge, multiplicity: -1, 1
Geometry:
C -1.596213    0.002479   -0.01970
B  0.026743    0.000053   -0.01594
O  0.613795   -1.165489   -0.73804
O  0.455060    0.003837    1.42065
O  0.617845    1.158883   -0.74522
H -2.001291    0.001550   -1.03942
H -2.016705    0.882986    0.48544
H -2.019708   -0.874979    0.48823
H  0.280403   -1.964910   -0.32815
H  1.415872    0.000397    1.42960
H  0.291388    1.961971   -0.33686
```

```
Molecule 57: k2 - Transition State
#n M06L/6-311++G** SCRF=(Solvent=Water) Opt=(TS,CalcFC,noeigentest)
E(RM06L) =  -485.667880954
Charge, multiplicity: -1, 1
```

```
Geometry:
C -0.836057    0.050620   -0.45068
B  1.553096   -0.489948   -0.00959
O  1.651698   -0.082478    1.31367
O  1.373161   -1.842720   -0.28315
H  0.877327   -2.259110    0.42570
O  2.211583    0.265448   -0.98644
H  1.330936    2.344602   -0.26210
H  0.109417    1.401963    0.00366
O  0.576138    2.297652    0.33284
H  2.044428   -0.099266   -1.85856
H  1.462904    0.870340    1.33692
C -1.537810   -0.968158    0.49314
C -2.906106   -0.446244    0.04138
H -1.353420   -0.729394    1.55306
H -1.344902   -2.042796    0.36637
C -2.196215    0.826621   -0.42710
H -3.724694   -0.368617    0.77566
H -3.273986   -1.022485   -0.81573
H -2.211795    1.595532    0.36114
H -2.554291    1.305241   -1.34857
H -0.770918   -0.426520   -1.44540
```

```
Molecule 57: k2 - Intermediate
#n M06L/6-311++G** SCRF=(Solvent=Water) Opt
E(RM06L) =  -409.258182916
Charge, multiplicity: -1, 1
Geometry:
O  1.823832   -1.235153    0.49068
B  1.101424   -0.013198    0.02622
O  1.189651    0.149574   -1.46592
O  1.877916    1.111467    0.60595
C -0.448204   -0.037358    0.51191
```

```
C -1.432750    1.078607    0.07954
C -2.555771    0.033560    0.02487
C -1.469509   -1.045337   -0.08251
H  1.225514   -1.982702    0.44823
H  0.456498   -0.307990   -1.88315
H  1.631320    1.904453    0.12689
H -0.498645   -0.124671    1.60924
H -1.181833    1.443076   -0.92521
H -1.567811    1.949179    0.73110
H -3.310990    0.105203   -0.76669
H -3.075795   -0.033226    0.98635
H -1.634538   -2.000615    0.42865
H -1.244628   -1.270652   -1.13532
```

```
Molecule 58: k2 - Transition State
#n M06L/6-311++G** SCRF=(Solvent=Water) Opt=(TS,CalcFC,noeigentest)
E(RM06L) =  -564.337662071
Charge, multiplicity: -1, 1
Geometry:
C -0.276346    0.116371   -0.25096
B  2.126179   -0.492688   -0.23802
O  2.418324   -0.458276    1.12125
O  1.931185   -1.745559   -0.82477
H  1.573802   -2.359155   -0.17884
O  2.668991    0.506462   -1.05935
H  1.899118    2.383002    0.42252
H  0.780035    1.297691    0.51896
O  1.271146    2.045210    1.06797
H  2.376076    0.385944   -1.96543
H  2.258275    0.447813    1.43394
C -0.865226   -0.799767    0.81772
C -2.271099   -1.325712    0.50800
C -3.230639   -0.180706    0.20117
```

```
H -2.666215   -1.939408    1.33103
H -2.215114   -1.987526   -0.36961
H -3.364429    0.424620    1.11098
H -4.226053   -0.563852   -0.05558
C -2.689450    0.715715   -0.90787
C -1.288570    1.227378   -0.55566
H -3.387382    1.544569   -1.09762
H -2.635747    0.137046   -1.84237
H -0.197021   -1.652030    1.01474
H -0.913916   -0.251883    1.77798
H -1.391938    1.891375    0.32096
H -0.921129    1.876210   -1.36558
H -0.188441   -0.473337   -1.18112
```

```
Molecule 58: k2 - Intermediate
#n M06L/6-311++G** SCRF=(Solvent=Water) Opt
E(RM06L) =  -487.919842906
Charge, multiplicity: -1, 1
Geometry:
O  2.278344   -1.350293    0.43736
B  1.672207   -0.043300    0.05070
O  2.063332    0.130271   -1.37012
O  2.310970    1.103401    0.78999
C  0.056618   -0.048142    0.30666
C -0.690354   -1.145591   -0.45368
C -2.147065   -1.292818    0.01271
C -2.736875    0.052727    0.45957
C -2.109813    1.193067   -0.33156
C -0.609384    1.305607   -0.01467
H  1.754729   -1.740275    1.13855
H  1.958597    1.058410   -1.58633
H  1.841615    1.249468    1.61346
H -0.112158   -0.246776    1.38419
```

```
H -0.168806   -2.106093   -0.36763
H -0.668305   -0.897981   -1.52488
H -2.760205   -1.716957   -0.79294
H -2.204299   -2.009914    0.84092
H -3.827438    0.048720    0.35869
H -2.534295    0.214272    1.52659
H -2.254351    0.995449   -1.40261
H -2.622921    2.140617   -0.13291
H -0.097570    1.776079   -0.86762
H -0.465558    1.995344    0.82693
```

```
Molecule 60: k2 - Transition State
#n M06L/6-311++G** SCRF=(Solvent=Water) Opt=(TS,CalcFC,noeigentest)
E(RM06L) =  -554.703620272
Charge, multiplicity: -1, 1
Geometry:
C  0.624235    0.417667   -0.04960
B -1.500161   -0.741754    0.07469
O -2.021822   -0.363995   -1.15468
O -0.978990   -2.026084    0.20883
H -0.588499   -2.312957   -0.61989
O -2.031730   -0.152142    1.22601
H -2.024003    2.081369    0.47939
H -0.723570    1.644945   -0.20053
O -1.502181    2.293869   -0.30160
H -1.572081   -0.468737    2.00724
H -2.129529    0.599670   -1.15420
C  1.485733   -0.051664   -1.08108
N  2.764451   -0.268903   -0.72432
N  2.765709    0.068134    0.58252
C  1.531224    0.476691    1.00447
H  3.626077    0.000448    1.10749
H  1.221625   -0.267118   -2.11457
```

```
H  1.410296    0.767197    2.04305
```

```
Molecule 60: k2 - Intermediate
#n M06L/6-311++G** SCRF=(Solvent=Water) Opt
E(RM06L) =  -478.279325585
Charge, multiplicity: -1, 1
Geometry:
O  1.849674    1.308009   -0.49367
B  1.326454   -0.007402   -0.00331
O  1.907554   -0.216841    1.34002
O  1.840942   -1.162200   -0.78387
C -0.301182    0.000741   -0.00965
C -1.188456   -1.101597    0.03728
N -2.483154   -0.761186    0.04966
N -2.448289    0.585120    0.01251
C -1.180838    1.073484   -0.02591
H  1.318297    1.609906   -1.23271
H  1.754616    0.574687    1.85826
H  1.302708   -1.271341   -1.56865
H -0.929205   -2.153871    0.06319
H -3.314324    1.102183    0.00768
H -1.006773    2.140397   -0.05657
```

```
Molecule 61: k2 - Transition State
#n M06L/6-311++G** SCRF=(Solvent=Water) Opt=(TS,CalcFC,noeigentest)
E(RM06L) =  -653.204896475
Charge, multiplicity: -1, 1
Geometry:
C  0.432700   -0.033705    0.32222
B -1.729075    0.302128   -0.67570
O -2.303357    1.179482    0.23382
O -1.281550    0.811182   -1.89278
H -1.040741    1.735949   -1.80619
O -2.162617   -1.028647   -0.67105
```

```
H -2.094696   -1.405022    1.61996
H -0.870217   -0.505235    1.57167
O -1.617715   -0.788558    2.18656
H -1.702043   -1.536514   -1.34326
H -2.374446    0.731291    1.08964
C  1.394611    1.033825    0.26957
N  2.616833    0.675355   -0.09040
O  2.533179   -0.707277   -0.30226
C  1.232319   -1.079574   -0.04419
C  1.152549    2.475374    0.57079
C  0.975527   -2.530302   -0.23038
H  0.421706    2.906731   -0.11994
H  2.070973    3.062225    0.50190
H  0.737471    2.600228    1.57498
H  1.693993   -3.147715    0.31758
H  1.041324   -2.827398   -1.28327
H -0.025535   -2.775834    0.12593
```

```
Molecule 61: k2 - Intermediate
#n M06L/6-311++G** SCRF=(Solvent=Water) Opt
E(RM06L) =  -576.777405216
Charge, multiplicity: -1, 1
Geometry:
C  0.248119    2.578701    0.02688
C  0.855257    1.220068    0.00790
N  2.168919    1.102025    0.01298
O  2.406680   -0.278395    0.00984
C  1.205245   -0.922702    0.00050
C  1.291363   -2.400075   -0.01480
C  0.169354   -0.034380   -0.00114
B -1.437136   -0.246442   -0.01237
O -1.746322   -1.679050    0.16259
O -1.918069    0.310379   -1.31225
```

```
O -2.126208    0.461717    1.11110
H  0.995745    3.351387   -0.15926
H -0.539895    2.655212   -0.72585
H -0.213060    2.785530    0.99761
H  1.834574   -2.758510   -0.89452
H  1.821615   -2.778289    0.86462
H  0.285285   -2.814488   -0.02317
H -2.658322   -1.742153    0.45562
H -2.877583    0.258121   -1.32536
H -2.189787    1.394339    0.89488
```

```
Molecule 62: k2 - Transition State
#n M06L/6-311++G** SCRF=(Solvent=Water) Opt=(TS,CalcFC,noeigentest)
E(RM06L) =  -592.831173174
Charge, multiplicity: -1, 1
Geometry:
C  0.412270    0.335005   -0.12884
B -1.738033   -0.726574    0.16779
O -2.290486   -0.526986   -1.08890
O -1.216343   -1.980866    0.47100
H -0.917493   -2.423773   -0.32661
O -2.219996    0.034947    1.23573
H -2.225645    2.138073    0.21561
H -0.974722    1.573825   -0.44025
O -1.722175    2.228068   -0.60063
H -1.778103   -0.205639    2.05341
H -2.423889    0.422866   -1.22152
C  1.206365   -0.195007   -1.15527
N  2.535010   -0.424504   -1.10350
C  3.114518   -0.103677    0.05420
N  2.519677    0.410112    1.13156
C  1.192226    0.614412    1.00187
H  0.751688   -0.476170   -2.11204
```

```
H   0.726481    1.038518    1.89930
H   4.188771   -0.279786    0.12738
```

```
Molecule 62: k2 - Intermediate
#n M06L/6-311++G** SCRF=(Solvent=Water) Opt
E(RM06L) =  -516.404469516
Charge, multiplicity: -1, 1
Geometry:
O  -2.103214   -1.329986    0.34272
B  -1.558153    0.012172    0.00265
O  -2.069726    0.361356   -1.33374
O  -2.058838    1.093750    0.87978
C   0.083031   -0.015131    0.02330
C   0.864559   -1.171663    0.03086
N   2.208005   -1.188769    0.01039
C   2.792591    0.007737   -0.02216
N   2.186075    1.197104   -0.03691
C   0.846468    1.156462   -0.01364
H  -1.706493   -1.659534    1.15160
H  -1.939784   -0.390695   -1.91389
H  -1.598027    1.072958    1.71970
H   0.383741   -2.150908    0.05489
H   3.882034    0.017710   -0.03739
H   0.345066    2.125870   -0.02285
```

```
Molecule 63: k2 - Transition State
#n M06L/6-311++G** SCRF=(Solvent=Water) Opt=(TS,CalcFC,noeigentest)
E(RM06L) =  -576.779228252
Charge, multiplicity: -1, 1
Geometry:
C   0.404161    0.371118   -0.15700
B  -1.748094   -0.724912    0.21306
O  -2.324541   -0.576059   -1.03912
O  -1.203687   -1.959532    0.55366
```

```
H -0.894447   -2.417211   -0.23157
O -2.212685    0.073551    1.26091
H -2.222528    2.148709    0.11843
H -0.978648    1.528490   -0.52329
O -1.738781    2.176818   -0.71373
H -1.739232   -0.123083    2.07269
H -2.445657    0.370602   -1.20949
C  1.185881   -0.230539   -1.15808
N  2.508007   -0.501027   -1.12698
C  3.156484   -0.153384   -0.00776
C  2.527176    0.454924    1.07294
C  1.159162    0.710720    0.98008
H  0.707761   -0.545693   -2.09323
H  0.669575    1.189216    1.83465
H  4.224103   -0.371581    0.02293
H  3.103857    0.717035    1.95771
```

```
Molecule 63: k2 - Intermediate
#n M06L/6-311++G** SCRF=(Solvent=Water) Opt
E(RM06L) =  -500.355617885
Charge, multiplicity: -1, 1
Geometry:
O -2.088990    1.102739   -0.83713
B -1.567686   -0.016669    0.00444
O -2.083643    0.190014    1.37319
O -2.114634   -1.334510   -0.38494
C  0.071671   -0.005259   -0.02834
C  0.815482    1.182804   -0.02982
C  2.205570    1.157018   -0.00088
C  2.843695   -0.078177    0.02561
N  2.183280   -1.241924    0.02578
C  0.842062   -1.174911    0.00149
H -1.552064    1.201943   -1.62555
```

```
H -1.872100    1.086613    1.63900
H -1.741208   -1.599742   -1.22670
H  0.294363    2.139204   -0.05604
H  2.790766    2.071915   -0.00096
H  3.930924   -0.138337    0.04602
H  0.332045   -2.139590    0.00436
```

```
Molecule 64: k2 - Transition State
#n M06L/6-311++G** SCRF=(Solvent=Water) Opt=(TS,CalcFC,noeigentest)
E(RM06L) =  -576.780525090
Charge, multiplicity: -1, 1
Geometry:
C  0.400011    0.350306   -0.16685
B -1.765101   -0.717231    0.22059
O -2.333613   -0.591736   -1.03744
O -1.218240   -1.942925    0.58662
H -0.903137   -2.414227   -0.18821
O -2.231507    0.102808    1.24994
H -2.224281    2.159884    0.07519
H -0.986030    1.513453   -0.54670
O -1.735110    2.166007   -0.75426
H -1.760231   -0.077303    2.06686
H -2.458435    0.350874   -1.22543
C  1.183309   -0.258923   -1.16721
C  2.549901   -0.474932   -1.01761
N  3.244761   -0.128028    0.07731
C  2.530080    0.458940    1.04997
C  1.163192    0.705613    0.96283
H  0.686562    1.190822    1.82005
H  0.723339   -0.592799   -2.10178
H  3.129972   -0.950893   -1.81180
H  3.093216    0.743281    1.94204
```

```
Molecule 64: k2 - Intermediate
```

```
#n M06L/6-311++G** SCRF=(Solvent=Water) Opt
E(RM06L) =  -500.356993181
Charge, multiplicity: -1, 1
Geometry:
O -2.103414    1.161161   -0.74193
B -1.576536   -0.020893    0.00009
O -2.076374    0.078451    1.38550
O -2.119786   -1.306611   -0.48775
C  0.063437   -0.014502   -0.03385
C  0.814121    1.170990   -0.03578
C  2.202604    1.138585   -0.00371
N  2.923863    0.008738    0.02726
C  2.219807   -1.132100    0.02860
C  0.831605   -1.187206    0.00071
H -1.595532    1.305708   -1.54258
H -1.913326    0.970535    1.69707
H -1.695450   -1.543868   -1.31365
H  0.307287    2.133799   -0.06483
H  2.774772    2.066511   -0.00566
H  2.806383   -2.050730    0.05204
H  0.338655   -2.157260    0.00397
```

```
Molecule 65: k2 - Transition State
#n M06L/6-311++G** SCRF=(Solvent=Water) Opt=(TS,CalcFC,noeigentest)
E(RM06L) =  -708.379547905
Charge, multiplicity: -1, 1
Geometry:
C -0.199874    0.447537    0.47657
B -2.265170    0.020283   -0.74700
O -3.184860    0.667706    0.06430
O -1.832596    0.655324   -1.90743
H -1.828187    1.607656   -1.78523
O -2.243172   -1.376098   -0.74395
```

```
H -2.254391   -1.701026    1.61609
H -1.422184   -0.419694    1.55531
O -2.077406   -0.919030    2.14964
H -1.543103   -1.701442   -1.31548
H -3.208741    0.213922    0.92024
C  0.930768   -0.350249    0.18236
C  2.207234    0.242862   -0.01770
N  2.503244    1.542874    0.04694
C  1.436327    2.304486    0.33597
C  0.140393    1.811551    0.54662
H -0.631173    2.556520    0.75990
C  1.125553   -1.765113    0.01463
H  1.634215    3.376180    0.40181
C  2.449686   -1.971984   -0.26889
N  3.108144   -0.759307   -0.29141
H  4.091048   -0.629043   -0.47476
H  0.378251   -2.541989    0.10790
H  2.994150   -2.885221   -0.45752
```

```
Molecule 65: k2 - Intermediate
#n M06L/6-311++G** SCRF=(Solvent=Water) Opt
E(RM06L) =  -631.956896984
Charge, multiplicity: -1, 1
Geometry:
O -2.182250   -1.047263    1.23777
B -2.033967   -0.238240    0.00104
C -0.546434    0.445911   -0.03312
C  0.633650   -0.328827   -0.02550
C  1.890522    0.329065    0.00304
N  2.868912   -0.634648    0.02113
C  2.275865   -1.877602    0.00114
C  0.913230   -1.734461   -0.02803
N  2.109842    1.645020    0.01177
```

```
C   0.982767    2.366957   -0.01170
C  -0.309529    1.824813   -0.03296
O  -3.117235    0.765430   -0.01503
H  -1.393581   -1.577403    1.37309
H   3.860737   -0.456076    0.03951
H   2.885643   -2.768356    0.00320
H   0.198550   -2.543802   -0.07297
H   1.117374    3.448190   -0.00991
H  -1.151728    2.513553   -0.05022
H  -3.017105    1.353015    0.73697
O  -2.225597   -1.136450   -1.16304
H  -2.021094   -0.659396   -1.96989
```

```
Molecule 66: k2 - Transition State
#n M06L/6-311++G** SCRF=(Solvent=Water) Opt=(TS,CalcFC,noeigentest)
E(RM06L) =  -897.567188288
Charge, multiplicity: -1, 1
Geometry:
C  -0.429382    0.243374    0.23150
B   1.698625   -0.724838   -0.10300
O   2.253871   -0.444283    1.13931
O   1.251649   -2.019357   -0.35454
H   0.922842   -2.422415    0.45228
O   2.154042    0.008515   -1.20484
H   2.133244    2.090696   -0.33795
H   0.887751    1.657729    0.39116
O   1.642551    2.300433    0.46505
H   1.710999   -0.274035   -2.00841
H   2.363247    0.513129    1.22445
C  -1.226597   -0.141601    1.29279
N  -2.587660   -0.330452    1.11864
C  -2.905987   -0.088771   -0.12475
S  -1.543448    0.376370   -1.09808
```

```
H -3.914665   -0.165365   -0.51650
H -0.832876   -0.324773    2.29162
```

```
Molecule 66: k2 - Intermediate
#n M06L/6-311++G** SCRF=(Solvent=Water) Opt
E(RM06L) =  -821.132095588
Charge, multiplicity: -1, 1
Geometry:
O  1.910390   -1.060528   -0.90077
B  1.510876    0.050137    0.00813
O  2.137776    1.344719   -0.31638
O  2.017557   -0.283936    1.34607
C -0.124857    0.214061   -0.01770
C -0.920222    1.332217    0.01592
N -2.287362    1.167250    0.04116
C -2.577166   -0.104210    0.03023
S -1.199457   -1.151613   -0.01864
H  1.423704   -1.005535   -1.72550
H  1.730753    1.712646   -1.10209
H  1.812681   -1.203069    1.52583
H -0.523857    2.343031    0.02592
H -3.587127   -0.497136    0.04318
```

```
Molecule 67: k2 - Transition State
#n M06L/6-311++G** SCRF=(Solvent=Water) Opt=(TS,CalcFC,noeigentest)
E(RM06L) =  -594.028890739
Charge, multiplicity: -1, 1
Geometry:
C  0.500293   -0.453494    0.21197
B -1.572914    0.324654   -0.68625
O -2.063486    1.119607    0.34118
O -1.028679    0.958943   -1.80112
H -0.686271    1.823803   -1.56375
O -2.153340   -0.931805   -0.88376
```

```
H -2.183612   -1.644165    1.42308
H -0.865788   -0.879983    1.42913
O -1.638948   -1.125445    2.02453
H -1.700399   -1.404933   -1.58593
H -2.214389    0.561710    1.11925
N  1.469122    0.499677    0.37137
N  2.722359    0.165923   -0.06400
C  2.556021   -1.080061   -0.52282
C  1.226610   -1.505798   -0.37848
C  1.276746    1.819175    0.92564
H  0.217133    1.940430    1.14732
H  1.584294    2.590621    0.21295
H  1.856922    1.954517    1.84347
H  3.410027   -1.606354   -0.93537
H  0.823892   -2.467449   -0.67502
```

```
Molecule 67: k2 - Intermediate
#n M06L/6-311++G** SCRF=(Solvent=Water) Opt
E(RM06L) =  -517.600765454
Charge, multiplicity: -1, 1
Geometry:
C -0.881419    2.037806    0.04310
N -1.154840    0.620133   -0.02775
N -2.448290    0.203037    0.00968
C -2.350948   -1.128968    0.01427
C -1.013111   -1.549360   -0.00891
C -0.239590   -0.390475   -0.03228
B  1.368901   -0.165842   -0.02143
O  1.699538    0.643446   -1.22875
O  1.855817    0.533929    1.20534
O  1.995099   -1.498107   -0.01063
H -0.871391    2.389178    1.08006
H -1.653720    2.582419   -0.50018
```

```
H  0.088352   2.228005  -0.41563
H -3.254866  -1.725103   0.02613
H -0.638585  -2.562258  -0.01615
H  2.642107   0.829833  -1.21803
H  1.684689   1.474538   1.12505
H  2.887591  -1.397747   0.32771
```

```
Molecule 68: k2 - Transition State
#n M06L/6-311++G** SCRF=(Solvent=Water) Opt=(TS,CalcFC,noeigentest)
E(RM06L) =  -554.716650045
Charge, multiplicity: -1, 1
Geometry:
C  0.656302   0.338763   0.26866
B -1.484736  -0.717355   0.05257
O -1.656354  -0.358429  -1.28088
O -1.063593  -2.011238   0.34198
H -0.517717  -2.358180  -0.36732
O -2.271218  -0.076975   1.01254
H -2.095969   2.065178   0.33929
H -0.672656   1.692125  -0.01274
O -1.395743   2.334418  -0.26567
H -2.081657  -0.415223   1.89062
H -1.797800   0.598226  -1.32803
N  1.281929  -0.071906  -0.87425
N  2.632106  -0.268798  -0.81302
C  2.905657   0.046839   0.45753
C  1.745364   0.422605   1.15819
H  3.931250  -0.012709   0.80493
H  1.699210   0.723518   2.19781
H  0.812104  -0.272682  -1.74665
```

```
Molecule 68: k2 - Intermediate
#n M06L/6-311++G** SCRF=(Solvent=Water) Opt
E(RM06L) =  -478.290422643
```

```
Charge, multiplicity: -1, 1
Geometry:
O -1.893519    0.997345    0.93313
B -1.285710    0.008346    0.00508
O -1.885098    0.271945   -1.32198
O -1.565660   -1.399230    0.35902
C  0.336558    0.121791    0.01214
C  1.254709    1.171051   -0.00764
C  2.528177    0.577247   -0.02929
N  2.454348   -0.757441   -0.02149
N  1.118766   -0.986455    0.00054
H -1.425793    0.979233    1.77042
H -1.911267    1.220748   -1.46206
H -2.501489   -1.561944    0.22133
H  1.023659    2.226964   -0.00436
H  3.500820    1.052656   -0.04696
H  0.768380   -1.933139    0.03025
```

6.1.3 k_{2Ar}

```
Molecule 0: k2Ar - Misc. small molecule
#n M06L/6-311++G** SCRF=(Solvent=Water) Opt
E(RM06L) =  -252.544612170
Charge, multiplicity: 0, 1
Geometry:
B -0.003606    0.020891    0.00008
O  0.050934   -1.349120   -0.00005
O -1.230075    0.626202    0.00003
H  0.935459   -1.721156    0.00008
H -1.942286   -0.017511    0.00002
O  1.067085    0.872581   -0.00014
H  1.921297    0.436903    0.00084
```

```
Molecule 28: k2Ar - Intermediate, BA detached
#n M06L/6-311++G** SCRF=(Solvent=Water) Opt
E(RM06L) =  -346.252942668
Charge, multiplicity: -1, 1
Geometry:
C -0.000999   1.053361  -0.00000
C -1.376040   1.345441  -0.00000
C -2.417800   0.394083   0.00000
C -1.917685  -0.931805   0.00000
C -0.564399  -1.280818   0.00000
C  0.406705  -0.278633  -0.00000
O  1.720461  -0.695971  -0.00000
C  2.709017   0.313091   0.00000
H  3.671560  -0.196922   0.00001
H  2.640460   0.950246   0.89005
H  2.640479   0.950255  -0.89003
H  0.730094   1.857180  -0.00000
H -1.620558   2.413363  -0.00000
H -2.618175  -1.774084   0.00000
H -0.240341  -2.320583   0.00000
```

```
Molecule 28: k2Ar - Intermediate
#n M06L/6-311++G** SCRF=(Solvent=Water) Opt
E(RM06L) =  -598.851794667
Charge, multiplicity: -1, 1
Geometry:
C -1.448077  -0.968897  -0.00936
C -0.053329  -1.059562  -0.00556
C  0.791234   0.055718   0.00952
C  0.140163   1.300287   0.01262
C -1.243641   1.431518   0.00466
C -2.048395   0.289972  -0.00407
O -3.401286   0.504473  -0.00256
```

```
C -4.234586   -0.640341    0.00570
H -5.259471   -0.273159    0.01093
H -4.066651   -1.253994    0.89789
H -4.078094   -1.259919   -0.88437
H -2.049537   -1.871233   -0.01400
H  0.395063   -2.051819   -0.00444
H  0.732085    2.215344    0.03005
H -1.720604    2.407573    0.01003
B  2.414421   -0.107804   -0.00688
O  2.818615   -0.276200   -1.42376
O  3.104714    1.058675    0.62638
H  2.850039    1.864999    0.17309
H  3.773403   -0.386014   -1.43828
O  2.876781   -1.305414    0.73711
H  2.740847   -1.157201    1.67503
```

```
Molecule 71: k2Ar - Intermediate, BA detached
#n M06L/6-311++G** SCRF=(Solvent=Water) Opt
E(RM06L) =  -430.258946063
Charge, multiplicity: -1, 1
Geometry:
C  0.000005   -1.142836   -0.00000
C  1.127114   -0.349648    0.00000
C  1.203561    1.039798    0.00000
C -0.000000    1.742113    0.00000
C -1.203568    1.039795   -0.00000
C -1.127088   -0.349645   -0.00000
F -2.376387   -0.989599    0.00000
F  2.376384   -0.989588   -0.00000
H -0.000080    2.827536    0.00000
H  2.159816    1.553875    0.00000
H -2.159852    1.553811   -0.00000
```

```
Molecule 71: k2Ar - Intermediate
```

```
#n M06L/6-311++G** SCRF=(Solvent=Water) Opt
E(RM06L) =  -682.833847478
Charge, multiplicity: -1, 1
Geometry:
C  0.026917   0.005852   0.03006
C  0.800946  -1.150004   0.00918
C  2.185770  -1.212668   0.01552
C  2.893037  -0.015785   0.02685
C  2.203632   1.191215   0.01808
C  0.817694   1.149918   0.01077
F  0.188021   2.369490  -0.03632
H  2.718547   2.144702   0.00899
B -1.637928   0.007054   0.02867
H  2.686395  -2.173712   0.00429
F  0.150944  -2.358864  -0.03660
O -2.205600  -1.117787   0.78807
O -2.212766   1.181383   0.69994
H -1.843361   1.974988   0.31165
H -1.856579  -1.937099   0.43560
O -1.978391  -0.049355  -1.41972
H -2.937212  -0.041109  -1.48999
H  3.977247  -0.023772   0.03317
```

```
Molecule 72: k2Ar - Intermediate, BA detached
#n M06L/6-311++G** SCRF=(Solvent=Water) Opt
E(RM06L) =  -728.014049941
Charge, multiplicity: -1, 1
Geometry:
C -0.000003  -1.758983  -0.00000
C -1.146590  -1.010285  -0.00000
C -1.197531   0.375760  -0.00000
C -0.000001   1.076426  -0.00000
C  1.197528   0.375757  -0.00000
```

```
C  1.146585   -1.010289    0.00000
F  2.387260   -1.632335    0.00000
F -2.387261   -1.632333    0.00000
F  0.000014    2.421422    0.00000
F -2.360278    1.072166   -0.00000
F  2.360274    1.072155    0.00000
```

```
Molecule 72: k2Ar - Intermediate
#n M06L/6-311++G** SCRF=(Solvent=Water) Opt
E(RM06L) =  -980.578469223
Charge, multiplicity: -1, 1
Geometry:
C -0.671716    0.033269    0.02651
C  0.063394   -1.143534    0.00737
C  1.449056   -1.205732    0.00730
C  2.176330   -0.026058    0.01475
C  1.497819    1.181162    0.01157
C  0.109191    1.178899    0.01042
F -0.470112    2.406437   -0.02909
F  2.190093    2.329670    0.00083
B -2.352653    0.021741    0.02665
F  2.094482   -2.381073   -0.00558
F -0.575177   -2.341842   -0.02655
O -2.883148   -1.024757    0.90772
O -2.928879    1.250376    0.57314
H -2.607138    2.005021    0.07934
H -2.609418   -1.880861    0.57663
O -2.675653   -0.198083   -1.40256
H -3.633111   -0.197758   -1.48898
F  3.512928   -0.053572    0.01659
```

```
Molecule 73: k2Ar - Intermediate, BA detached
#n M06L/6-311++G** SCRF=(Solvent=Water) Opt
E(RM06L) =  -231.711306115
```

```
Charge, multiplicity: -1, 1
Geometry:
C -1.197876   -0.647606    0.00000
C -1.176965    0.752308   -0.00000
C  0.000000    1.539266   -0.00000
C  1.176965    0.752307    0.00000
C  1.197876   -0.647606   -0.00000
C -0.000000   -1.360607    0.00000
H -2.145144   -1.187581    0.00000
H -2.154337    1.247342    0.00000
H  2.154337    1.247342   -0.00000
H  2.145143   -1.187582   -0.00000
H -0.000000   -2.447897   -0.00000
```

```
Molecule 73: k2Ar - Intermediate
#n M06L/6-311++G** SCRF=(Solvent=Water) Opt
E(RM06L) =  -484.309783230
Charge, multiplicity: -1, 1
Geometry:
C -2.200879   -1.209309   -0.00319
C -0.807347   -1.185183    0.00584
C -0.074224    0.011908    0.01782
C -0.829302    1.194154    0.01901
C -2.223960    1.192772    0.00741
C -2.918384   -0.014511   -0.00357
H -2.731573   -2.158367   -0.00883
H -0.259851   -2.126219    0.01223
H -0.317124    2.156333    0.03488
H -2.771089    2.132272    0.00890
B  1.559169   -0.007214   -0.00783
O  1.954209   -0.125334   -1.43144
O  2.150770    1.206203    0.63161
H  1.811820    1.993696    0.20098
```

```
H  2.914574   -0.150810   -1.46062
O  2.128627   -1.168010    0.71746
H  2.017588   -1.028455    1.66001
H -4.004470   -0.024232   -0.00947
```

```
Molecule 74: k2Ar - Intermediate, BA detached
#n M06L/6-311++G** SCRF=(Solvent=Water) Opt
E(RM06L) =  -330.985732726
Charge, multiplicity: -1, 1
Geometry:
C -0.279191   -1.338362    0.00000
C  1.127866   -1.206802   -0.00000
C  1.812082    0.015954   -0.00000
C  1.093002    1.209306   -0.00000
C -0.300542    1.155994    0.00000
C -0.891221   -0.101779    0.00001
F -2.302413   -0.063359   -0.00000
H  1.748635   -2.108366   -0.00000
H  1.601518    2.169157   -0.00000
H  2.900428    0.040986   -0.00000
H -0.900837    2.062591    0.00000
```

```
Molecule 74: k2Ar - Intermediate
#n M06L/6-311++G** SCRF=(Solvent=Water) Opt
E(RM06L) =  -583.574002244
Charge, multiplicity: -1, 1
Geometry:
C  0.045023   -0.248225    0.01279
C  0.780823   -1.441643    0.03028
C  2.175202   -1.477152    0.01883
C  2.898337   -0.288247   -0.00797
C  2.216458    0.926269   -0.01963
C  0.831924    0.896266   -0.00964
F  0.197547    2.119832   -0.00965
```

```
H  2.741489   1.875433  -0.03520
B -1.597662  -0.207851   0.01950
H  2.697444  -2.429310   0.03181
H  0.229019  -2.379294   0.06027
O -2.193037  -1.526928   0.31539
O -2.121841   0.661208   1.09652
H -1.714127   1.524221   1.00358
H -2.065069  -2.095313  -0.44566
O -1.985552   0.270465  -1.33666
H -2.945112   0.321731  -1.35961
H  3.983574  -0.298272  -0.01585
```

```
Molecule 75: k2Ar - Intermediate, BA detached
#n M06L/6-311++G** SCRF=(Solvent=Water) Opt
E(RM06L) =  -330.974665195
Charge, multiplicity: -1, 1
Geometry:
C  1.151664   1.375700   0.00000
C -0.256110   1.235297  -0.00000
C -0.878147  -0.004161  -0.00000
C -0.186046  -1.202067  -0.00000
C  1.206629  -1.115831   0.00000
C  1.833096   0.133933   0.00000
H  2.927153   0.123033   0.00000
H -0.916621   2.106771   0.00000
H -0.714560  -2.149085  -0.00000
F -2.257268  -0.064820   0.00000
H  1.792926  -2.034557   0.00000
```

```
Molecule 75: k2Ar - Intermediate
#n M06L/6-311++G** SCRF=(Solvent=Water) Opt
E(RM06L) =  -583.570716190
Charge, multiplicity: -1, 1
Geometry:
```

```
C -0.248559    0.180907   -0.06202
C  0.719800   -0.832802   -0.05236
C  2.065108   -0.511650   -0.00578
C  2.531479    0.790086    0.03059
C  1.578913    1.808585    0.01828
C  0.221321    1.503474   -0.02627
H -0.505146    2.312430   -0.03714
H  1.910199    2.843212    0.04366
B -1.839932   -0.151294   -0.03172
F  2.981988   -1.525791    0.00132
H  0.420724   -1.876223   -0.08662
O -2.406069   -0.031798    1.35104
O -2.561519    0.852788   -0.83927
H -3.466060    0.889860   -0.52022
H -2.074726   -0.759247    1.88193
O -2.006247   -1.542729   -0.52980
H -2.936769   -1.771491   -0.47014
H  3.595860    0.992352    0.06494
```

```
Molecule 76: k2Ar - Intermediate, BA detached
#n M06L/6-311++G** SCRF=(Solvent=Water) Opt
E(RM06L) =  -330.971680514
Charge, multiplicity: -1, 1
Geometry:
C -1.980961   -0.000000    0.00000
C -1.194861   -1.175650   -0.00000
C  0.207738   -1.207476    0.00000
C  0.879817   -0.000001    0.00000
C  0.207736    1.207470   -0.00000
C -1.194868    1.175651    0.00000
H -1.684685    2.154367   -0.00000
H -1.684690   -2.154360    0.00000
F  2.253492    0.000004    0.00000
```

```
H  0.770172  -2.138284   0.00000
H  0.770173   2.138282  -0.00000
```

```
Molecule 76: k2Ar - Intermediate
#n M06L/6-311++G** SCRF=(Solvent=Water) Opt
E(RM06L) =  -583.569527234
Charge, multiplicity: -1, 1
Geometry:
C -0.349018  -0.027175  -0.00813
C  0.405011  -1.209105  -0.00886
C  1.801525  -1.213558  -0.00443
C  2.456270   0.003301   0.00029
C  1.774742   1.208333  -0.00009
C  0.382033   1.172014  -0.00421
H -0.158000   2.117215  -0.00446
H  2.327572   2.142080   0.00306
B -1.978948   0.006116  -0.00002
H  2.374120  -2.135429  -0.00435
H -0.117381  -2.162232  -0.01290
O -2.515875   0.718138   1.18992
O -2.531397   0.804112  -1.12585
H -2.222731   0.419378  -1.94763
H -2.199886   0.270665   1.97641
O -2.442686  -1.406386  -0.04791
H -3.402607  -1.389335  -0.03329
F  3.821105   0.019701   0.00519
```

```
Molecule 77: k2Ar - Intermediate, BA detached
#n M06L/6-311++G** SCRF=(Solvent=Water) Opt
E(RM06L) =  -430.243240955
Charge, multiplicity: -1, 1
Geometry:
C  0.620321  -1.534534  -0.00000
C  1.825693  -0.799326   0.00000
```

```
C  1.892352   0.597910   0.00000
C  0.716395   1.347838  -0.00000
C -0.482731   0.657050  -0.00000
C -0.492707  -0.729443  -0.00000
F -1.769201  -1.304750   0.00000
H  2.779881  -1.332869   0.00000
H  0.714619   2.432218   0.00000
H  2.851262   1.113244   0.00000
F -1.655433   1.365910   0.00000
```

```
Molecule 77: k2Ar - Intermediate
#n M06L/6-311++G** SCRF=(Solvent=Water) Opt
E(RM06L) =  -682.827374088
Charge, multiplicity: -1, 1
Geometry:
C  0.301580   0.385605   0.01460
C -0.159287   1.709888   0.02906
C -1.512869   2.040367   0.01998
C -2.479156   1.037991  -0.00109
C -2.048372  -0.275890  -0.00875
C -0.694637  -0.578101  -0.00097
F -0.360824  -1.902519   0.00437
F -2.951453  -1.285478  -0.02313
B  1.899979  -0.012797   0.01607
H -1.825571   3.079873   0.02977
H  0.579823   2.506978   0.05479
O  2.766091   1.157555   0.24824
O  2.233714  -0.924292   1.13139
H  1.673511  -1.699443   1.06188
H  2.735854   1.719459  -0.52769
O  2.146614  -0.626346  -1.31589
H  3.073437  -0.878731  -1.34980
H -3.541356   1.253336  -0.00739
```

```
Molecule 78: k2Ar - Intermediate, BA detached
#n M06L/6-311++G** SCRF=(Solvent=Water) Opt
E(RM06L) =  -430.244528713
Charge, multiplicity: -1, 1
Geometry:
C  1.280423    1.199098   -0.00000
C -0.006863    1.777363    0.00000
C -1.214662    1.062497    0.00000
C -1.139733   -0.317659   -0.00000
C  0.071432   -0.987273   -0.00000
C  1.205120   -0.177921   -0.00000
F  2.402963   -0.910190    0.00000
H -0.105047    2.866455    0.00000
F -2.291933   -1.054762    0.00000
H -2.183677    1.552929    0.00000
H  0.115156   -2.071446   -0.00000
```

```
Molecule 78: k2Ar - Intermediate
#n M06L/6-311++G** SCRF=(Solvent=Water) Opt
E(RM06L) =  -682.831214753
Charge, multiplicity: -1, 1
Geometry:
C -0.328942   -0.228693    0.01307
C  0.443557   -1.396830    0.03193
C  1.838921   -1.403028    0.02284
C  2.489513   -0.183786   -0.00242
C  1.805091    1.019208   -0.01586
C  0.420517    0.940375   -0.00842
F -0.243043    2.141222   -0.00955
H  2.325110    1.968911   -0.02931
B -1.973137   -0.243881    0.01831
H  2.410685   -2.323866    0.03639
H -0.076621   -2.351410    0.06106
```

```
O -2.517474   -1.587305    0.30048
O -2.526942    0.595330    1.10356
H -2.154747    1.475057    1.01945
H -2.390513   -2.134839   -0.47593
O -2.376286    0.234405   -1.33202
H -3.336655    0.269365   -1.35047
F  3.847719   -0.153082   -0.00997
```

```
Molecule 79: k2Ar - Intermediate, BA detached
#n M06L/6-311++G** SCRF=(Solvent=Water) Opt
E(RM06L) =  -430.246405301
Charge, multiplicity: -1, 1
Geometry:
C  0.731500   -1.336597    0.00000
C -0.675249   -1.231816   -0.00000
C -1.328197   -0.006252   -0.00000
C -0.659169    1.204719    0.00000
C  0.734912    1.156646    0.00000
C  1.336656   -0.094703    0.00000
F  2.743720   -0.048058   -0.00000
H -1.310970   -2.120655   -0.00000
H -1.202300    2.142592   -0.00000
F -2.705034    0.020810   -0.00000
H  1.322382    2.071311   -0.00000
```

```
Molecule 79: k2Ar - Intermediate
#n M06L/6-311++G** SCRF=(Solvent=Water) Opt
E(RM06L) =  -682.831950033
Charge, multiplicity: -1, 1
Geometry:
C -0.196916   -0.065058    0.00887
C  0.837000   -1.008767    0.02191
C  2.164981   -0.616032    0.00719
C  2.554387    0.710565   -0.01748
```

```
C  1.550363   1.676055  -0.02374
C  0.231009   1.257635  -0.00994
F -0.723901   2.247646  -0.00082
H  1.790452   2.733415  -0.03792
B -1.784567  -0.500901   0.01897
F  3.133634  -1.575994   0.01896
H  0.598120  -2.068428   0.05131
O -1.963464  -1.940032   0.28970
O -2.520137   0.162016   1.11611
H -2.383062   1.107888   1.03664
H -1.717197  -2.430766  -0.49585
O -2.299463  -0.128237  -1.32590
H -3.236981  -0.339313  -1.34529
H  3.603481   0.980481  -0.02724
```

```
Molecule 80: k2Ar - Intermediate, BA detached
#n M06L/6-311++G** SCRF=(Solvent=Water) Opt
E(RM06L) =  -430.227708039
Charge, multiplicity: -1, 1
Geometry:
C -1.943171  -0.873733  -0.00000
C -0.647255  -1.437711   0.00000
C  0.513508  -0.676844  -0.00000
C  0.453318   0.706439  -0.00000
C -0.778285   1.334771   0.00000
C -1.932168   0.540867   0.00000
H -2.879051   1.087482  -0.00000
H -0.495524  -2.520593   0.00000
F  1.607839   1.428361   0.00000
F  1.746658  -1.267465   0.00000
H -0.811572   2.422308   0.00000
```

```
Molecule 80: k2Ar - Intermediate
#n M06L/6-311++G** SCRF=(Solvent=Water) Opt
```

```
E(RM06L) =  -682.823088694
Charge, multiplicity: -1, 1
Geometry:
C -0.593740    0.129250   -0.03097
C  0.287687   -0.960684   -0.02900
C  1.658049   -0.770888   -0.01137
C  2.197471    0.505189    0.00504
C  1.363150    1.608742    0.00268
C -0.014964    1.407466   -0.01473
H -0.667586    2.277227   -0.02131
H  1.802920    2.600745    0.01318
B -2.214913   -0.057673    0.00072
F  2.503449   -1.831314   -0.01309
H -0.096946   -1.976150   -0.04734
O -2.768790    0.226171    1.35109
O -2.914119    0.931072   -0.85218
H -2.628711    0.805191   -1.75848
H -2.350288   -0.364018    1.97999
O -2.486287   -1.456263   -0.42279
H -3.435227   -1.593096   -0.36981
F  3.543002    0.655334    0.01991
```

```
Molecule 81: k2Ar - Intermediate, BA detached
#n M06L/6-311++G** SCRF=(Solvent=Water) Opt
E(RM06L) =  -430.236674547
Charge, multiplicity: -1, 1
Geometry:
C -0.000003    1.902260    0.00000
C -1.187131    1.132712    0.00000
C -1.168338   -0.252571    0.00000
C  0.000001   -0.998370   -0.00000
C  1.168334   -0.252562   -0.00000
C  1.187129    1.132719   -0.00000
```

```
H  2.171633   1.605382   0.00000
H -2.171632   1.605381  -0.00000
H  0.000011  -2.080426  -0.00000
F -2.353650  -0.950859  -0.00000
F  2.353654  -0.950859   0.00000
```

```
Molecule 81: k2Ar - Intermediate
#n M06L/6-311++G** SCRF=(Solvent=Water) Opt
E(RM06L) =  -682.830018100
Charge, multiplicity: -1, 1
Geometry:
C -0.444730  -0.006345  -0.04720
C  0.288413  -1.202377  -0.04105
C  1.670706  -1.169285  -0.01115
C  2.403640   0.006232   0.01104
C  1.659988   1.175755   0.00168
C  0.278296   1.195519  -0.02560
H -0.243424   2.147908  -0.03594
F  2.340426   2.354901   0.01790
B -2.078496   0.007817  -0.00024
F  2.361598  -2.342573  -0.00690
H -0.222246  -2.159650  -0.06617
O -2.563000   0.127312   1.39974
O -2.656500   1.201341  -0.65314
H -2.407526   1.193987  -1.57873
H -2.231290  -0.620368   1.89999
O -2.525536  -1.258280  -0.63303
H -3.484174  -1.282897  -0.58230
H  3.485337   0.011000   0.03066
```

```
Molecule 82: k2Ar - Intermediate, BA detached
#n M06L/6-311++G** SCRF=(Solvent=Water) Opt
E(RM06L) =  -529.495212074
Charge, multiplicity: -1, 1
```

```
Geometry:
C  1.137506   1.575215  -0.00000
C -0.186098   2.062078   0.00000
C -1.332589   1.257897   0.00000
C -1.162190  -0.114913  -0.00000
C  0.111562  -0.662110  -0.00000
C  1.197204   0.202721  -0.00000
F  2.433195  -0.445167   0.00000
H -0.363017   3.140344   0.00000
F -2.228758  -0.954987   0.00000
H -2.339366   1.666794   0.00000
F  0.252231  -2.014564  -0.00000
```

```
Molecule 82: k2Ar - Intermediate
#n M06L/6-311++G** SCRF=(Solvent=Water) Opt
E(RM06L) =  -782.078035933
Charge, multiplicity: -1, 1
Geometry:
C -0.578953  -0.340846   0.01279
C  0.033735  -1.600168   0.02868
C  1.415636  -1.784964   0.02134
C  2.230464  -0.669443   0.00098
C  1.679376   0.602869  -0.00834
C  0.297777   0.732805  -0.00260
F -0.180134   2.008381   0.00302
F  2.477680   1.685334  -0.02010
B -2.215450  -0.140836   0.01586
H  1.866964  -2.770650   0.03272
H -0.606025  -2.478129   0.05568
O -2.921941  -1.411299   0.25860
O -2.658023   0.727471   1.12630
H -2.203182   1.568355   1.05225
H -2.853859  -1.954114  -0.52844
```

```
O -2.540023    0.426984   -1.31863
H -3.488577    0.579979   -1.34448
F  3.575075   -0.785718   -0.00452
```

```
Molecule 83: k2Ar - Intermediate, BA detached
#n M06L/6-311++G** SCRF=(Solvent=Water) Opt
E(RM06L) =  -529.503203552
Charge, multiplicity: -1, 1
Geometry:
C  0.183929   -1.688126    0.00000
C -1.169628   -1.295443   -0.00000
C -1.540207    0.040954   -0.00000
C -0.621638    1.079430    0.00000
C  0.712357    0.704975    0.00000
C  1.065944   -0.634677    0.00000
F  2.442571   -0.878183   -0.00000
H -1.976901   -2.029965   -0.00000
H -0.920576    2.119366   -0.00000
F -2.871106    0.377963   -0.00000
F  1.663306    1.685545   -0.00000
```

```
Molecule 83: k2Ar - Intermediate
#n M06L/6-311++G** SCRF=(Solvent=Water) Opt
E(RM06L) =  -782.084244640
Charge, multiplicity: -1, 1
Geometry:
C -0.410321   -0.221857    0.01016
C  0.370317   -1.384588    0.01966
C  1.751882   -1.313282    0.00903
C  2.445873   -0.115080   -0.00694
C  1.678443    1.036881   -0.01056
C  0.293415    0.972820   -0.00269
F -0.377680    2.161204    0.00589
F  2.288479    2.241093   -0.02091
```

```
B -2.061279   -0.273091    0.01741
F  2.474212   -2.464785    0.01571
H -0.111995   -2.357000    0.04209
O -2.566631   -1.634812    0.25970
O -2.618612    0.519862    1.13072
H -2.294198    1.418922    1.05301
H -2.440060   -2.153746   -0.53618
O -2.463823    0.239546   -1.31604
H -3.424399    0.245130   -1.34737
H  3.526812   -0.071590   -0.01185
```

```
Molecule 84: k2Ar - Intermediate, BA detached
#n M06L/6-311++G** SCRF=(Solvent=Water) Opt
E(RM06L) =  -529.498584020
Charge, multiplicity: -1, 1
Geometry:
C  1.228639   -1.395302    0.00000
C -0.166462   -1.598495   -0.00000
C -1.086357   -0.558390    0.00000
C -0.658681    0.758985    0.00000
C  0.695626    1.043345    0.00000
C  1.551278   -0.054120    0.00000
F  2.908244    0.301066   -0.00000
H -0.593509   -2.604234   -0.00000
F -1.570978    1.762459   -0.00000
F -2.429783   -0.801749   -0.00000
H  1.041898    2.072120    0.00000
```

```
Molecule 84: k2Ar - Intermediate
#n M06L/6-311++G** SCRF=(Solvent=Water) Opt
E(RM06L) =  -782.082607163
Charge, multiplicity: -1, 1
Geometry:
C -0.528346   -0.098327    0.00863
```

```
C  0.408749   -1.137728    0.02156
C  1.771494   -0.898302    0.01107
C  2.257614    0.398828   -0.00897
C  1.380594    1.468717   -0.01622
C  0.025575    1.175154   -0.00772
F -0.820580    2.253180   -0.00151
H  1.748238    2.487287   -0.02601
B -2.151650   -0.379442    0.01756
F  2.654920   -1.923865    0.02252
H  0.066460   -2.168638    0.04727
O -2.463819   -1.796455    0.28304
O -2.820209    0.345558    1.11773
H -2.600230    1.275843    1.04115
H -2.284551   -2.300919   -0.51201
O -2.628012    0.045369   -1.32438
H -3.582088   -0.069542   -1.34271
F  3.589290    0.611503   -0.01620
```

```
Molecule 85: k2Ar - Intermediate, BA detached
#n M06L/6-311++G** SCRF=(Solvent=Water) Opt
E(RM06L) =  -529.483089824
Charge, multiplicity: -1, 1
Geometry:
C -0.000000   -2.193166   -0.00000
C -1.187256   -1.425417    0.00000
C -1.186448   -0.039660    0.00000
C  0.000000    0.677304   -0.00000
C  1.186448   -0.039660   -0.00000
C  1.187256   -1.425417    0.00000
H  2.171733   -1.898425    0.00000
H -2.171733   -1.898425   -0.00000
F  0.000000    2.028510   -0.00000
F -2.347911    0.678686   -0.00000
```

```
F  2.347911    0.678686    0.00000
```

```
Molecule 85: k2Ar - Intermediate
#n M06L/6-311++G** SCRF=(Solvent=Water) Opt
E(RM06L) =  -782.075462819
Charge, multiplicity: -1, 1
Geometry:
C -0.755474   -0.026230   -0.00788
C -0.013923   -1.214745   -0.00689
C  1.369425   -1.189303   -0.00270
C  2.068379    0.007131    0.00032
C  1.340406    1.189358   -0.00149
C -0.040106    1.181170   -0.00534
H -0.562675    2.133579   -0.00650
F  2.031891    2.351559    0.00081
B -2.391309    0.001059    0.00021
F  2.085890   -2.336531   -0.00139
H -0.518248   -2.175421   -0.00967
O -2.917333    0.713883    1.18875
O -2.934476    0.795879   -1.12643
H -2.651219    0.391819   -1.94820
H -2.621376    0.253341    1.97559
O -2.833812   -1.413851   -0.04603
H -3.793767   -1.415851   -0.03400
F  3.412947    0.024490    0.00452
```

```
Molecule 86: k2Ar - Intermediate, BA detached
#n M06L/6-311++G** SCRF=(Solvent=Water) Opt
E(RM06L) =  -628.749196703
Charge, multiplicity: -1, 1
Geometry:
C  0.688493   -1.846954   -0.00000
C -0.716566   -1.742947   -0.00000
C -1.370261   -0.521055   -0.00000
```

```
C -0.655825    0.668006   -0.00000
C  0.729753    0.608091   -0.00000
C  1.337667   -0.637754   -0.00000
F  2.731208   -0.592008    0.00000
H -1.355130   -2.628389   -0.00000
F -1.285578    1.859451    0.00000
F -2.730903   -0.433868    0.00000
F  1.427002    1.773544   -0.00000
```

```
Molecule 86: k2Ar - Intermediate
#n M06L/6-311++G** SCRF=(Solvent=Water) Opt
E(RM06L) =  -881.328639896
Charge, multiplicity: -1, 1
Geometry:
C -0.699935   -0.217012    0.01103
C  0.087054   -1.374530    0.02111
C  1.468203   -1.317565    0.01149
C  2.132014   -0.100804   -0.00480
C  1.380487    1.065049   -0.00885
C -0.004261    0.981023   -0.00144
F -0.675690    2.165224    0.00701
F  2.006914    2.251878   -0.01766
B -2.352000   -0.282413    0.01649
F  2.212889   -2.444334    0.01930
H -0.392735   -2.348322    0.04337
O -2.838418   -1.652861    0.24380
O -2.917574    0.492470    1.13722
H -2.611596    1.398408    1.06543
H -2.725711   -2.155024   -0.56482
O -2.757997    0.240406   -1.31113
H -3.718641    0.248685   -1.33946
F  3.471356   -0.038187   -0.01057
```

```
Molecule 87: k2Ar - Intermediate, BA detached
```

```
#n M06L/6-311++G** SCRF=(Solvent=Water) Opt
E(RM06L) =  -529.513937080
Charge, multiplicity: -1, 1
Geometry:
C -0.643156   -1.212780    0.00000
C -1.522847   -0.152019    0.00000
C -1.221731    1.204826    0.00000
C  0.126298    1.560849   -0.00000
C  1.066576    0.546296   -0.00000
C  0.659269   -0.782021   -0.00000
F  1.695951   -1.712523    0.00000
F -2.894688   -0.437552   -0.00000
H  0.446231    2.596285   -0.00000
H -1.999309    1.962753    0.00000
F  2.395028    0.866748   -0.00000
```

```
Molecule 87: k2Ar - Intermediate
#n M06L/6-311++G** SCRF=(Solvent=Water) Opt
E(RM06L) =  -782.085156366
Charge, multiplicity: -1, 1
Geometry:
C  0.253322    0.096072    0.02521
C -0.093132    1.444792    0.00678
C -1.377891    1.959872    0.00963
C -2.447285    1.069714    0.01884
C -2.169576   -0.281736    0.01508
C -0.856043   -0.736706    0.01050
F -0.696346   -2.088512   -0.02870
F -3.176794   -1.184434    0.00666
B  1.838732   -0.428911    0.02563
H -1.538632    3.031175   -0.00107
F  0.921305    2.367680   -0.02802
O  2.684939    0.392684    0.90372
```

```
O  2.002895  -1.780067   0.57209
H  1.441189  -2.388702   0.09172
H  2.640803   1.303419   0.60964
O  2.225788  -0.331876  -1.40555
H  3.139054  -0.623216  -1.47809
H -3.474908   1.411312   0.02167
```

```
Molecule 88: k2Ar - Intermediate, BA detached
#n M06L/6-311++G** SCRF=(Solvent=Water) Opt
E(RM06L) =  -529.516214766
Charge, multiplicity: -1, 1
Geometry:
C  0.000020  -1.522332  -0.00000
C  1.127269  -0.731791   0.00000
C  1.212959   0.659855   0.00000
C -0.000017   1.325954  -0.00000
C -1.212973   0.659822  -0.00000
C -1.127249  -0.731820  -0.00000
F -2.373027  -1.363883   0.00000
F  2.373062  -1.363824  -0.00000
F -0.000037   2.688250   0.00000
H  2.153239   1.198524   0.00000
H -2.153269   1.198464  -0.00000
```

```
Molecule 88: k2Ar - Intermediate
#n M06L/6-311++G** SCRF=(Solvent=Water) Opt
E(RM06L) =  -782.089282874
Charge, multiplicity: -1, 1
Geometry:
C -0.322097   0.008548   0.02840
C  0.453494  -1.145196   0.00497
C  1.839677  -1.213642   0.00724
C  2.512403  -0.006394   0.01646
C  1.852085   1.207370   0.00975
```

```
C  0.464781    1.154180    0.00677
F -0.159903    2.369716   -0.03814
H  2.386596    2.148009   -0.00147
B -1.989030    0.003264    0.03088
H  2.364501   -2.159694   -0.00557
F -0.186402   -2.352951   -0.03880
O -2.546635   -1.118276    0.80127
O -2.564626    1.179417    0.69684
H -2.217852    1.972947    0.28824
H -2.213571   -1.940484    0.44046
O -2.332734   -0.065166   -1.41482
H -3.291714   -0.064240   -1.48320
F  3.864870   -0.013416    0.02128
```

```
Molecule 89: k2Ar - Intermediate, BA detached
#n M06L/6-311++G** SCRF=(Solvent=Water) Opt
E(RM06L) =  -628.764973057
Charge, multiplicity: -1, 1
Geometry:
C  0.978495   -1.363481   -0.00000
C  1.639848   -0.156932   -0.00000
C  1.093708    1.123456   -0.00000
C -0.289433    1.185013   -0.00000
C -1.050299    0.026310    0.00000
C -0.381784   -1.192004    0.00000
F -1.221550   -2.298497    0.00000
F  3.035675   -0.172012    0.00000
F -0.924293    2.379660    0.00000
H  1.688041    2.030392   -0.00000
F -2.404415    0.117010    0.00000
```

```
Molecule 89: k2Ar - Intermediate
#n M06L/6-311++G** SCRF=(Solvent=Water) Opt
E(RM06L) =  -881.334254936
```

```
Charge, multiplicity: -1, 1
Geometry:
C -0.533925   -0.067821    0.02540
C  0.019942   -1.343174    0.00362
C  1.369797   -1.658525    0.00441
C  2.260412   -0.601493    0.01370
C  1.801080    0.703817    0.01160
C  0.429376    0.929921    0.00969
F  0.062433    2.236636   -0.02836
F  2.668628    1.729850    0.00124
B -2.183972    0.202839    0.02791
H  1.719734   -2.682491   -0.00798
F -0.829762   -2.413512   -0.03352
O -2.887623   -0.746049    0.90160
O -2.554770    1.508231    0.58294
H -2.118119    2.201505    0.08740
H -2.710408   -1.637223    0.59818
O -2.550426    0.056861   -1.40325
H -3.497198    0.206871   -1.47657
F  3.586293   -0.823148    0.01599
```

```
Molecule 90: k2Ar - Intermediate, BA detached
#n M06L/6-311++G** SCRF=(Solvent=Water) Opt
E(RM06L) =  -628.768674488
Charge, multiplicity: -1, 1
Geometry:
C -0.000002   -1.493379   -0.00000
C  1.144664   -0.737689   -0.00000
C  1.179552    0.649372   -0.00000
C  0.000003    1.376297   -0.00000
C -1.179563    0.649380    0.00000
C -1.144672   -0.737693    0.00000
F -2.388894   -1.359198   -0.00000
```

```
F  2.388896   -1.359182    0.00000
H -0.000023    2.458279    0.00000
F  2.365927    1.320514   -0.00000
F -2.365915    1.320532    0.00000
```

```
Molecule 90: k2Ar - Intermediate
#n M06L/6-311++G** SCRF=(Solvent=Water) Opt
E(RM06L) =  -881.331588722
Charge, multiplicity: -1, 1
Geometry:
C -0.400139    0.086974    0.00010
C  0.265750   -1.138138    0.00004
C  1.646148   -1.267347    0.00004
C  2.467102   -0.154503    0.00006
C  1.841209    1.075718    0.00003
C  0.454716    1.184778    0.00004
F -0.023206    2.451313   -0.00005
F  2.587650    2.201800   -0.00002
B -2.071770    0.083605    0.00002
F  2.196160   -2.500814    0.00000
F -0.438495   -2.293082   -0.00011
O -2.576982   -0.664711    1.16449
O -2.531250    1.483911    0.00026
H -3.491446    1.446349    0.00017
H -2.381375   -0.159495    1.95528
O -2.576794   -0.664253   -1.16482
H -2.380873   -0.158848   -1.95541
H  3.545043   -0.243447    0.00004
```

```
Molecule 91: k2Ar - Intermediate, BA detached
#n M06L/6-311++G** SCRF=(Solvent=Water) Opt
E(RM06L) =  -445.527107793
Charge, multiplicity: -1, 1
Geometry:
```

```
C   1.855174    1.115804    0.00000
C   0.618294    1.789502   -0.00000
C  -0.648071    1.177987   -0.00000
C  -0.720683   -0.213663   -0.00000
C   0.461776   -0.957400   -0.00000
C   1.656018   -0.251377    0.00000
F   2.791413   -1.085358    0.00000
H   0.424856   -2.042989    0.00000
H   0.605772    2.883902    0.00000
O  -1.887044   -0.939954   -0.00000
C  -3.095875   -0.203772    0.00000
H  -3.901865   -0.936209    0.00000
H  -3.183009    0.428807   -0.89077
H  -3.183007    0.428807    0.89078
H  -1.548909    1.783046   -0.00000
```

```
Molecule 91: k2Ar - Intermediate
#n M06L/6-311++G** SCRF=(Solvent=Water) Opt
E(RM06L) =  -698.115975737
Charge, multiplicity: -1, 1
Geometry:
C  -0.718879   -0.192032    0.01147
C   0.179277   -1.263201    0.02848
C   1.569893   -1.121555    0.01993
C   2.120497    0.159229   -0.00458
C   1.273539    1.270749   -0.01642
C  -0.089725    1.047952   -0.00861
F  -0.878812    2.175592   -0.00750
H   1.680576    2.275219   -0.02883
B  -2.348808   -0.379626    0.01815
H   2.203497   -2.000152    0.03439
H  -0.230185   -2.271185    0.05708
O  -2.752053   -1.772769    0.30396
```

```
O -2.994863    0.397474    1.10142
H -2.713895    1.310387    1.01596
H -2.527590   -2.314712   -0.45404
O -2.810595    0.050875   -1.33276
H -3.768766   -0.024814   -1.34451
O  3.457806    0.432005   -0.01396
C  4.340683   -0.677187    0.00364
H  5.347857   -0.264811   -0.00712
H  4.201123   -1.314781   -0.87588
H  4.206648   -1.281759    0.90703
```

```
Molecule 92: k2Ar - Intermediate, BA detached
#n M06L/6-311++G** SCRF=(Solvent=Water) Opt
E(RM06L) =  -668.098098381
Charge, multiplicity: -1, 1
Geometry:
C -2.573611    0.850283    0.00000
C -1.552463    1.823565    0.00000
C -0.181330    1.542942   -0.00000
C  0.238352    0.215148   -0.00000
C -0.714267   -0.807959   -0.00000
C -2.045742   -0.427919    0.00000
F -2.938320   -1.509119   -0.00000
H -0.416814   -1.853383   -0.00000
H -1.822362    2.883059    0.00000
C  1.677604   -0.151688   -0.00000
F  2.503698    0.913426   -0.00006
H  0.554523    2.341701   -0.00000
F  2.028044   -0.904298   -1.07935
F  2.028066   -0.904187    1.07942
```

```
Molecule 92: k2Ar - Intermediate
#n M06L/6-311++G** SCRF=(Solvent=Water) Opt
E(RM06L) =  -920.682988342
```

```
Charge, multiplicity: -1, 1
Geometry:
C -1.313227   -0.230837    0.01055
C -0.530573   -1.390727    0.03177
C  0.862922   -1.372883    0.02601
C  1.528129   -0.151850    0.00125
C  0.800129    1.039079   -0.01280
C -0.577942    0.950539   -0.00801
F -1.260052    2.138531   -0.00901
H  1.290536    2.006513   -0.02659
B -2.959589   -0.262688    0.01675
H  1.427735   -2.297951    0.03999
H -1.040658   -2.350549    0.05945
O -3.487444   -1.614194    0.27852
O -3.509831    0.552609    1.11959
H -3.167801    1.444235    1.03255
H -3.352085   -2.151583   -0.50355
O -3.360069    0.236100   -1.32405
H -4.320632    0.245447   -1.35524
C  3.016045   -0.073635   -0.00310
F  3.609849   -1.281171   -0.00990
F  3.501344    0.588958    1.07846
F  3.494047    0.598469   -1.08142
```

```
Molecule 93: k2Ar - Intermediate, BA detached
#n M06L/6-311++G** SCRF=(Solvent=Water) Opt
E(RM06L) =  -535.552272163
Charge, multiplicity: -1, 1
Geometry:
C -1.441598   -1.345152   -0.00003
C -0.040958   -1.246661   -0.00002
C  0.641199   -0.019705    0.00001
C -0.038516    1.200019    0.00003
```

```
C -1.420823   1.160943   0.00001
C -2.032104  -0.093886  -0.00000
F -3.419560  -0.033125   0.00000
H -2.007017   2.075097   0.00003
H  0.581036  -2.140840  -0.00005
N  2.094807   0.005759   0.00001
H  0.506058   2.134833   0.00005
O  2.672093   1.098534  -0.00007
O  2.706547  -1.066612   0.00005
```

```
Molecule 93: k2Ar - Intermediate
#n M06L/6-311++G** SCRF=(Solvent=Water) Opt
E(RM06L) =  -788.134231885
Charge, multiplicity: -1, 1
Geometry:
C -0.742099   0.039131   0.00326
C  0.490958  -0.610353   0.01128
C  1.691090   0.100804   0.00101
C  1.725675   1.493064  -0.01532
C  0.519605   2.171542  -0.01590
C -0.654889   1.428904  -0.00571
F -1.813577   2.141674   0.00920
H  0.480469   3.254432  -0.02235
B -2.174152  -0.779451   0.01492
N  2.953931  -0.628503   0.00759
H  0.521357  -1.694343   0.03228
O -1.973879  -2.220274   0.24523
O -3.027711  -0.350726   1.14025
H -3.176231   0.593653   1.06584
H -1.639608  -2.615944  -0.56123
O -2.781353  -0.504703  -1.31043
H -3.639108  -0.937549  -1.33422
H  2.671717   2.017681  -0.02248
```

```
O  4.002496    0.017127   -0.01804
O  2.919798   -1.858271    0.03797
```

```
Molecule 94: k2Ar - Intermediate, BA detached
#n M06L/6-311++G** SCRF=(Solvent=Water) Opt
E(RM06L) =  -544.800088684
Charge, multiplicity: -1, 1
Geometry:
C  1.867995    0.523741   -0.00000
C  0.738221    1.305574    0.00000
C -0.603150    0.917542    0.00000
C -0.853703   -0.454594    0.00000
C  0.218603   -1.350350    0.00000
C  1.495139   -0.804554   -0.00000
F  2.513355   -1.768611   -0.00000
H  0.040162   -2.420282   -0.00000
F  0.902651    2.699387   -0.00000
O -2.105267   -1.008814    0.00000
C -3.203080   -0.113590   -0.00000
H -4.100666   -0.729763    0.00000
H -3.202084    0.523627   -0.89134
H -3.202084    0.523628    0.89134
H -1.397390    1.653706    0.00000
```

```
Molecule 94: k2Ar - Intermediate
#n M06L/6-311++G** SCRF=(Solvent=Water) Opt
E(RM06L) =  -797.375661637
Charge, multiplicity: -1, 1
Geometry:
C -0.704167    0.017191    0.03021
C  0.203200   -1.031052    0.00364
C  1.590261   -0.943721    0.00314
C  2.154884    0.330252    0.01158
C  1.322680    1.451188    0.00609
```

```
C -0.043618    1.243505    0.00645
F -0.806374    2.382913   -0.03575
H  1.738628    2.450892   -0.00741
B -2.356730   -0.157722    0.03258
F -0.288878   -2.311749   -0.04235
O -2.801747   -1.375186    0.72889
O -3.044191    0.914507    0.77106
H -2.786191    1.759042    0.40062
H -2.349862   -2.130995    0.35259
O -2.711994   -0.164456   -1.41460
H -3.666743   -0.260528   -1.47457
O  3.492121    0.574081    0.01529
C  4.354901   -0.553295    0.01238
H  5.368878   -0.159004    0.02158
H  4.210877   -1.163146   -0.88543
H  4.199459   -1.176230    0.89923
H  2.183517   -1.847877   -0.01288
```

```
Molecule 95: k2Ar - Intermediate, BA detached
#n M06L/6-311++G** SCRF=(Solvent=Water) Opt
E(RM06L) =  -743.296501594
Charge, multiplicity: -1, 1
Geometry:
C  1.617575    1.331064    0.00002
C  0.289367    1.656219    0.00000
C -0.779730    0.766238   -0.00001
C -0.532804   -0.610218   -0.00002
C  0.817097   -0.983002   -0.00001
C  1.815163   -0.025894    0.00000
F  3.107939   -0.540265    0.00001
F  1.106122   -2.313342   -0.00001
F -0.095273    2.995068    0.00001
O -1.424214   -1.634488   -0.00004
```

```
C -2.824622   -1.369194    0.00007
H -3.299972   -2.348710    0.00009
H -3.130157   -0.818146   -0.89198
H -3.130006   -0.818188    0.89220
F -2.058614    1.244058   -0.00004
```

```
Molecule 95: k2Ar - Intermediate
#n M06L/6-311++G** SCRF=(Solvent=Water) Opt
E(RM06L) =  -995.864549764
Charge, multiplicity: -1, 1
Geometry:
C -0.978188   -0.045588    0.02049
C -0.107680   -1.120985   -0.03929
C  1.276415   -1.018385   -0.10839
C  1.898962    0.229084   -0.11064
C  1.048686    1.336073   -0.07439
C -0.325659    1.178860   -0.02423
F -1.050242    2.331578   -0.01864
F  1.588188    2.569358   -0.09344
B -2.647499   -0.161911    0.08686
F -0.592422   -2.393184   -0.07541
O -3.110522   -1.434167    0.64647
O -3.225584    0.838864    0.99446
H -3.016009    1.713755    0.66542
H -2.733470   -2.158902    0.14698
O -3.053702    0.036047   -1.32682
H -4.011016   -0.043285   -1.36630
O  3.228004    0.460276   -0.20201
C  4.116610   -0.435563    0.47777
H  5.044832    0.116765    0.61353
H  4.308350   -1.331943   -0.11357
H  3.714107   -0.717102    1.45369
F  2.015616   -2.143181   -0.21013
```

```
Molecule 96: k2Ar - Intermediate, BA detached
#n M06L/6-311++G** SCRF=(Solvent=Water) Opt
E(RM06L) =  -905.938298073
Charge, multiplicity: -1, 1
Geometry:
C -0.000000   2.494703   0.00000
C -1.172133   1.708539  -0.00000
C -1.188952   0.306163  -0.00000
C  0.000000  -0.416117  -0.00000
C  1.188952   0.306163   0.00000
C  1.172133   1.708539   0.00000
H  2.151351   2.196357   0.00000
H -2.151351   2.196357  -0.00000
C -2.498092  -0.403742  -0.00000
C  2.498092  -0.403742   0.00000
H  0.000000  -1.498707  -0.00000
F  2.386593  -1.750827  -0.00000
F  3.261019  -0.088396   1.07978
F -2.386593  -1.750827  -0.00001
F -3.261024  -0.088385  -1.07977
F -3.261014  -0.088402   1.07978
F  3.261020  -0.088392  -1.07978
```

```
Molecule 96: k2Ar - Intermediate
#n M06L/6-311++G** SCRF=(Solvent=Water) Opt
E(RM06L) =  -1158.52913257
Charge, multiplicity: -1, 1
Geometry:
C -1.378202  -0.150188  -0.04311
C -0.517900  -1.253899  -0.03313
C  0.868703  -1.110013  -0.00764
C  1.453882   0.152282   0.00338
C  0.616253   1.261767  -0.01068
```

```
C -0.770703    1.109524   -0.03212
H -1.401985    1.995427   -0.04974
C  1.186407    2.641332   -0.00780
B -2.998113   -0.333260   -0.00494
C  1.717593   -2.338133    0.00417
H -0.948480   -2.252564   -0.05587
O -3.362197   -1.628239   -0.61703
O -3.473006   -0.384401    1.40927
H -3.159779    0.393997    1.87614
H -4.080170   -1.472049   -1.23111
O -3.607192    0.811369   -0.72204
H -4.497791    0.929391   -0.38397
H  2.530035    0.266944    0.01868
F  3.038349   -2.073486    0.01399
F  1.490590   -3.128424   -1.07399
F  1.473139   -3.118739    1.08602
F  2.533061    2.662162    0.01058
F  0.775114    3.362869    1.06452
F  0.804381    3.355537   -1.09548
```

```
Molecule 97: k2Ar - Intermediate, BA detached
#n M06L/6-311++G** SCRF=(Solvent=Water) Opt
E(RM06L) =  -640.838287162
Charge, multiplicity: -1, 1
Geometry:
C  0.000097    2.372389    0.00005
C  1.175950    1.591581   -0.00011
C  1.172531    0.191153   -0.00004
C -0.000011   -0.548094   -0.00005
C -1.172450    0.191153   -0.00000
C -1.175817    1.591576    0.00016
H -2.156878    2.064649    0.00044
N -2.442358   -0.552692    0.00004
```

```
H  2.157068   2.064544  -0.00036
N  2.442147  -0.552925  -0.00012
H -0.000126  -1.627928  -0.00008
O  2.402444  -1.782614  -0.00016
O  3.490174   0.088843   0.00025
O -3.490288   0.088765  -0.00030
O -2.402378  -1.782554   0.00029
```

```
Molecule 97: k2Ar - Intermediate
#n M06L/6-311++G** SCRF=(Solvent=Water) Opt
E(RM06L) =  -893.426636678
Charge, multiplicity: -1, 1
Geometry:
C -1.117443  -0.061581  -0.00674
C -0.337642  -1.222216  -0.00656
C  1.054556  -1.151599  -0.00295
C  1.741878   0.050528  -0.00053
C  0.962266   1.197754  -0.00175
C -0.427995   1.157780  -0.00410
H -0.978558   2.092609  -0.00388
N  1.648779   2.499383   0.00033
B -2.755363  -0.070941   0.00057
N  1.841571  -2.395036  -0.00076
H -0.818102  -2.193532  -0.00896
O -3.279549   0.637975   1.18753
O -3.297749   0.714248  -1.12814
H -3.043848   0.289629  -1.94932
H -3.012225   0.160659   1.97483
O -3.153295  -1.494101  -0.04276
H -4.112473  -1.533146  -0.03417
H  2.821204   0.092989   0.00206
O  3.065668  -2.304506   0.00172
O  1.233790  -3.460249  -0.00116
```

```
O  2.876014    2.504518    0.00679
O  0.959454    3.513500   -0.00454
```

```
Molecule 98: k2Ar - Intermediate , BA detached
#n M06L/6-311++G** SCRF=(Solvent=Water) Opt
E(RM06L) =  -271.031767917
Charge , multiplicity: -1, 1
Geometry:
C  0.144288   -1.189678   -0.00000
C -1.254364   -1.175189    0.00000
C -2.050077   -0.003501    0.00000
C -1.262597    1.170603   -0.00000
C  0.138670    1.191452    0.00000
C  0.873566    0.004169    0.00000
H  0.686292   -2.137896   -0.00000
H -1.741843   -2.156626   -0.00000
H -1.754431    2.149874    0.00000
H  0.677258    2.141443    0.00000
C  2.375401    0.003024    0.00000
H  2.775849    1.019882   -0.00000
H  2.783772   -0.510981   -0.87670
H  2.783772   -0.510980    0.87670
```

```
Molecule 98: k2Ar - Intermediate
#n M06L/6-311++G** SCRF=(Solvent=Water) Opt
E(RM06L) =  -523.631614780
Charge , multiplicity: -1, 1
Geometry:
C -0.391388    0.025283   -0.00007
C  0.345159   -1.169414   -0.00002
C  1.738445   -1.191968    0.00000
C  2.478231   -0.005840   -0.00000
C  1.763913    1.195949   -0.00006
C  0.370042    1.204550   -0.00011
```

```
H -0.150176    2.159981   -0.00015
H  2.312258    2.137103   -0.00007
B -2.020000   -0.005826    0.00000
H  2.267800   -2.143575    0.00004
H -0.191917   -2.117588   -0.00000
O -2.570625   -0.759069    1.15942
O -2.487829    1.407450   -0.00094
H -3.447836    1.385162   -0.00084
H -2.250082   -0.347362    1.96338
O -2.570751   -0.760700   -1.15829
H -2.250262   -0.350145   -1.96286
C  3.978472   -0.017068    0.00005
H  4.372072   -1.035738    0.00006
H  4.387231    0.495442    0.87674
H  4.387306    0.495451   -0.87659
```

```
Molecule 99: k2Ar - Intermediate, BA detached
#n M06L/6-311++G** SCRF=(Solvent=Water) Opt
E(RM06L) =  -691.324800168
Charge, multiplicity: -1, 1
Geometry:
C -1.616988    1.357625    0.00000
C -0.207773    1.250757   -0.00000
C  0.442808    0.020360    0.00000
C -0.235946   -1.190007   -0.00000
C -1.629415   -1.130897    0.00000
C -2.277521    0.106715   -0.00000
H -3.371240    0.078734    0.00000
H  0.426021    2.141099   -0.00000
Cl  2.234845   -0.030129    0.00000
H -2.196443   -2.061728    0.00000
H  0.298309   -2.133232   -0.00000
```

```
Molecule 99: k2Ar - Intermediate
```

```
#n M06L/6-311++G** SCRF=(Solvent=Water) Opt
E(RM06L) =  -943.919584138
Charge, multiplicity: -1, 1
Geometry:
C -0.630516    0.255334   -0.00256
C  0.504199   -0.566931   -0.00142
C  1.780225   -0.015532   -0.00021
C  1.995460    1.355253    0.00054
C  0.875142    2.185651    0.00132
C -0.404880    1.641450    0.00018
H -1.262436    2.311101   -0.00019
H  1.017087    3.262997    0.00194
B -2.151492   -0.340153   -0.00048
Cl  3.181465   -1.091499   -0.00108
H  0.382721   -1.645451   -0.00325
O -2.911813    0.095477    1.19807
O -2.962184    0.207681   -1.11659
H -2.549838   -0.047694   -1.94331
H -2.448387   -0.210406    1.97942
O -2.033645   -1.819162   -0.07484
H -2.924244   -2.177251   -0.05405
H  3.001015    1.759617    0.00012
```

6.1.4 k_{2cat} (B3LYP)

```
Molecule 0: k2cat_B3LYP - Misc. small molecule
#n B3LYP/6-31G(d) Opt
E(RB3LYP) =  -216.51957507
Charge, multiplicity: 0, 1
Geometry:
C  1.522933    0.025843   -0.00006
B -0.051728    0.004250   -0.00040
H  1.918630   -0.508459   -0.87295
```

```
H  1.916799   -0.494075    0.88241
H  1.923838    1.043453   -0.00756
O -0.681231   -1.219966   -0.00006
H -1.648341   -1.174392    0.00074
O -0.730603    1.200368   -0.00001
H -1.695214    1.113949    0.00039
```

```
Molecule 50: k2cat_B3LYP - Transition State
#n B3LYP/6-31G(d) Opt=(TS,CalcFC,noeigentest)
E(RB3LYP) =  -1021.4129105
Charge, multiplicity: -1, 1
Geometry:
C -1.784257   -0.438454   -1.02238
C -0.853465   -0.183073   -0.05320
B  0.249293    1.942992   -0.12028
O  0.904163    1.872557   -1.33820
O -0.905153    2.720781   -0.00773
H -1.444338    2.554569   -0.79296
O  0.986819    1.821020    1.08630
H  0.810683   -0.544968   -0.51733
O  1.728791   -0.848855   -0.89411
C -1.407429   -0.589792    1.22118
H  0.342124    1.854149    1.80802
H  1.451996    1.067759   -1.37736
B  2.754731   -0.960286    0.00569
O  2.798087   -0.292170    1.20145
H  2.087585    0.387560    1.25133
C  3.981349   -1.911640   -0.36408
H -0.857052   -0.501863    2.15928
H -1.710998   -0.276296   -2.09344
S -3.307726   -1.144317   -0.42162
C -2.675009   -1.107619    1.19944
H -3.276624   -1.469308    2.02617
```

```
H  4.345335   -1.703427   -1.37928
H  3.664722   -2.964444   -0.36584
H  4.821277   -1.811389    0.33301
```

```
Molecule 50: k2cat_B3LYP - Intermediate
#n B3LYP/6-31G(d) Opt
E(RB3LYP) =  -804.87817685
Charge, multiplicity: -1, 1
Geometry:
O  2.194585   -0.753501    1.12880
B  1.634600    0.038520   -0.00481
O  2.164377   -0.530803   -1.26942
O  2.043697    1.464725    0.13607
C -0.010030   -0.059277   -0.00687
C -0.770352   -1.285987    0.00031
C -2.127964   -1.134744    0.00337
S -2.566064    0.555680   -0.00009
C -0.862242    1.007937   -0.00783
H  2.568144   -0.067305    1.69956
H  2.872749   -1.107605   -0.94706
H  2.626074    1.584760   -0.62729
H -0.291696   -2.262137    0.00458
H -2.897775   -1.897744    0.00878
H -0.591207    2.055610   -0.01066
```

```
Molecule 51: k2cat_B3LYP - Transition State
#n B3LYP/6-31G(d) Opt=(TS,CalcFC,noeigentest)
E(RB3LYP) =  -698.43493712
Charge, multiplicity: -1, 1
Geometry:
C -0.993251   -0.512266   -0.10630
B -0.392433    1.768019    0.01738
O  0.340750    1.974394   -1.13916
O -1.700318    2.254608    0.07631
```

```
H -2.108187    2.068699   -0.78113
O  0.273129    1.671083    1.26740
H  0.647683   -0.509726   -0.65049
O  1.630331   -0.582927   -0.99841
H -0.396530    1.374984    1.90230
H  1.031361    1.292844   -1.22912
B  2.607825   -0.603211   -0.04165
O  2.463691   -0.071278    1.21434
H  1.638546    0.461235    1.28988
C  4.010867   -1.272535   -0.40711
C -1.390771   -1.283054    0.97146
O -2.053612   -0.604737   -1.03290
C -3.038641   -1.390599   -0.51741
C -2.687346   -1.844065    0.71770
H -0.793850   -1.444747    1.86381
H -3.914923   -1.536484   -1.13726
H -3.277928   -2.492440    1.35567
H  3.898787   -2.360038   -0.52446
H  4.779330   -1.091564    0.35343
H  4.381833   -0.900833   -1.37187
```

```
Molecule 51: k2cat_B3LYP - Intermediate
#n B3LYP/6-31G(d) Opt
E(RB3LYP) =  -481.89718042
Charge, multiplicity: -1, 1
Geometry:
O  1.738162   -0.823932   -1.13465
B  1.310287    0.006947    0.03182
O  1.909146    1.342297   -0.16167
O  1.717573   -0.559716    1.32485
C -0.334209    0.120173    0.04587
C -1.212928    1.173913    0.04518
C -2.550472    0.653251    0.00494
```

```
C -2.417022   -0.701888   -0.02068
O -1.097220   -1.045562    0.00324
H  1.234567   -1.647883   -1.06498
H  1.997474    1.423946   -1.12200
H  2.684643   -0.487467    1.30511
H -0.914412    2.213586    0.07336
H -3.483256    1.206429   -0.00435
H -3.123948   -1.520729   -0.05225
```

```
Molecule 52: k2cat_B3LYP - Transition State
#n B3LYP/6-31G(d) Opt=(TS,CalcFC,noeigentest)
E(RB3LYP) =  -1021.4224803
Charge, multiplicity: -1, 1
Geometry:
C -0.894282   -0.280251    0.02877
B  0.003466    1.898256    0.00223
O  0.673076    1.943185   -1.21105
O -1.195489    2.602775    0.12703
H -1.676955    2.514871   -0.70706
O  0.738045    1.762204    1.21055
H  0.789242   -0.536993   -0.54576
O  1.713872   -0.750983   -0.93463
H  0.083418    1.642774    1.91446
H  1.274842    1.183141   -1.29734
B  2.748786   -0.799262   -0.03627
O  2.733963   -0.175927    1.18219
H  1.966335    0.436250    1.26784
C  4.054390   -1.619004   -0.44496
C -1.295815   -0.872577    1.21482
S -2.201682   -0.574954   -1.11894
C -3.211842   -1.438704    0.01214
C -2.576531   -1.517643    1.22234
H -0.660325   -0.853540    2.10187
```

```
H -4.181714   -1.839536   -0.26020
H -3.004259   -2.017831    2.09010
H  3.839911   -2.697077   -0.46675
H  4.890479   -1.454193    0.24423
H  4.381414   -1.354533   -1.45948
```

```
Molecule 52: k2cat_B3LYP - Intermediate
#n B3LYP/6-31G(d) Opt
E(RB3LYP) =  -804.88202172
Charge, multiplicity: -1, 1
Geometry:
O  1.996484    0.110802   -1.38196
B  1.516607    0.059795    0.03290
O  2.055630    1.147828    0.86030
O  1.917341   -1.268912    0.53414
C -0.130477    0.248181    0.06600
C -0.937078    1.362985    0.01947
C -2.344137    1.102480   -0.03658
C -2.632176   -0.236391   -0.03360
S -1.164005   -1.171101    0.05399
H  1.649799    0.933286   -1.75494
H  3.007373    0.963745    0.88667
H  2.119521   -1.773709   -0.26603
H -0.515715    2.365194    0.03978
H -3.106050    1.879817   -0.07171
H -3.606318   -0.710969   -0.05382
```

```
Molecule 54: k2cat_B3LYP - Transition State
#n B3LYP/6-31G(d) Opt=(TS,CalcFC,noeigentest)
E(RB3LYP) =  -546.98354556
Charge, multiplicity: -1, 1
Geometry:
C  1.044855    1.331894   -0.77217
B  1.605721   -0.718222    0.08866
```

```
O  1.005089   -1.656313   -0.74312
O  2.996404   -0.558065   -0.03506
H  3.215427   -0.786367   -0.94898
O  1.085074   -0.498101    1.39540
H -0.260716    0.465906   -0.86930
O -1.235696   -0.037801   -1.04945
H  1.506512    0.313812    1.71553
H  0.072047   -1.403780   -0.89100
B -2.094252   -0.081642    0.00552
O -1.695031   -0.051867    1.32335
H -0.715820   -0.133294    1.38815
C -3.668111   -0.183727   -0.27385
C  0.969167    2.403607    0.03808
H  1.650651    1.508742   -1.67849
H  0.374178    2.372780    0.95943
H  1.456213    3.373358   -0.13615
H -4.253332   -0.370823    0.63472
H -4.037931    0.746821   -0.72883
H -3.886765   -0.981288   -0.99727
```

```
Molecule 54: k2cat_B3LYP - Intermediate
#n B3LYP/6-31G(d) Opt
E(RB3LYP) =  -330.45008571
Charge, multiplicity: -1, 1
Geometry:
C  2.275340    0.046478   -0.00079
C  1.124154   -0.637824    0.00086
B -0.390469   -0.030097   -0.00236
O -1.077849   -0.405043   -1.27626
O -0.378567    1.457582    0.13682
O -1.171032   -0.609203    1.13827
H  2.264452    1.137393   -0.00758
H  3.267031   -0.420168    0.00707
```

```
H  1.216930  -1.734836   0.01079
H -1.918358  -0.751923  -0.94127
H -0.832235   1.732231  -0.67257
H -1.422861   0.189172   1.62426
```

```
Molecule 55: k2cat_B3LYP - Transition State
#n B3LYP/6-31G(d) Opt=(TS,CalcFC,noeigentest)
E(RB3LYP) =  -586.27859345
Charge, multiplicity: -1, 1
Geometry:
C  1.747634   1.958274   0.20557
C  0.808802   0.969094  -0.44336
B  1.695973  -0.922435  -0.02359
O  3.044295  -0.686364  -0.38579
O  1.525678  -1.062984   1.38192
H  0.582097  -1.197346   1.55008
H  3.082875  -0.834213  -1.34019
O  1.033063  -1.805930  -0.93496
H  0.089512  -1.826672  -0.71570
O -1.232140  -0.652344   0.34208
B -2.424131  -0.113695  -0.02604
O -2.519503   1.129806  -0.62017
C -3.773038  -0.928864   0.20399
H -1.624234   1.512877  -0.67840
C  0.281524   2.197822   0.36512
H  2.308704   2.663294  -0.41190
H  2.308639   1.595480   1.06513
H -0.146101   3.068389  -0.14116
H -0.175101   2.008614   1.33824
H -3.846137  -1.757198  -0.51536
H -0.392824  -0.052495   0.08573
H  0.715686   1.136456  -1.52568
H -4.667281  -0.306356   0.08341
```

```
H -3.795718   -1.385617    1.20157
```

```
Molecule 55: k2cat_B3LYP - Intermediate
#n B3LYP/6-31G(d) Opt
E(RB3LYP) =  -369.75287067
Charge, multiplicity: -1, 1
Geometry:
O -0.710401    0.106529    1.45523
B -0.732657   -0.038176   -0.02475
O -1.411619   -1.291478   -0.41741
O -1.472889    1.174937   -0.52120
C  0.789505   -0.101539   -0.64485
C  1.889084    0.758623   -0.05768
C  1.913267   -0.738770    0.14404
H -0.905465    1.043530    1.59598
H -2.234947   -1.265493    0.09455
H -1.501927    1.079636   -1.48366
H  0.882226   -0.249392   -1.72767
H  1.628267    1.375522    0.80162
H  2.649983    1.201096   -0.70458
H  1.662150   -1.114546    1.13404
H  2.691131   -1.309257   -0.36842
```

```
Molecule 56: k2cat_B3LYP - Transition State
#n B3LYP/6-31G(d) Opt=(TS,CalcFC,noeigentest)
E(RB3LYP) =  -508.89617880
Charge, multiplicity: -1, 1
Geometry:
C -1.080068    1.427743   -1.16729
B -1.680084   -0.264835    0.29482
O -1.176729    0.092045    1.54298
O -3.066196   -0.115151    0.09345
H -3.330575    0.666981    0.59641
O -1.119032   -1.395099   -0.37853
```

```
H  0.173901    0.992385   -0.27760
O  1.132737    0.957513    0.28628
H -1.534971   -1.430951   -1.25154
H -0.233964    0.328206    1.44305
B  2.016548   -0.023749   -0.03435
O  1.658469   -1.221727   -0.61168
H  0.676024   -1.299961   -0.62571
C  3.573418    0.194597    0.27963
H  4.185240   -0.685133    0.04494
H  3.969323    1.048087   -0.28949
H  3.724456    0.443438    1.33965
H -0.300545    1.958521   -1.74818
H -1.695047    0.920450   -1.92707
H -1.710250    2.226206   -0.74091
```

```
Molecule 56: k2cat_B3LYP - Intermediate
#n B3LYP/6-31G(d) Opt
E(RB3LYP) =  -292.36377005
Charge, multiplicity: -1, 1
Geometry:
C -1.606124   -0.118011    0.02261
B  0.044850    0.004461   -0.03041
O  0.661655   -1.049033   -0.88085
O  0.522585   -0.123666    1.38833
O  0.488786    1.293079   -0.62734
H -2.042050   -0.060042   -0.98744
H -2.083083    0.678303    0.62306
H -1.949469   -1.070652    0.46309
H  0.587551   -1.843488   -0.33362
H  1.473251    0.053503    1.30352
H  0.042089    1.965103   -0.09330
```

```
Molecule 57: k2cat_B3LYP - Transition State
#n B3LYP/6-31G(d) Opt=(TS,CalcFC,noeigentest)
```

```
E(RB3LYP) =  -625.59350177
Charge, multiplicity: -1, 1
Geometry:
C  0.864467    2.158028   -0.63079
C  1.199339    0.617551   -0.49899
B  0.847790   -1.639801   -0.10861
O  0.036971   -2.115171   -1.13786
O  2.214500   -1.979042   -0.16714
H  2.447309   -2.018470   -1.10458
O  0.327057   -1.588668    1.22100
H -0.392675    0.227155   -0.65468
O -1.449395    0.209694   -0.91919
C  2.078026    1.027241    0.72970
H  1.019516   -1.208201    1.78028
H -0.746886   -1.539553   -1.21730
B -2.357199    0.222214    0.09720
O -2.079257   -0.196480    1.37649
H -1.192085   -0.625663    1.40363
C -3.843495    0.734033   -0.20349
C  2.108554    2.489195    0.22495
H -4.507997    0.647572    0.66458
H -0.063111    2.425014   -0.10410
H  0.805597    2.591805   -1.63902
H  1.883511    0.389016   -1.33364
H  3.035809    0.512080    0.87868
H  1.508130    0.960997    1.67321
H  2.989923    2.667363   -0.40686
H  2.044208    3.308876    0.96172
H -3.836009    1.784652   -0.52663
H -4.288541    0.166333   -1.03286
```

```
Molecule 57: k2cat_B3LYP - Intermediate
#n B3LYP/6-31G(d) Opt
```

```
E(RB3LYP) =  -409.06949398
Charge, multiplicity: -1, 1
Geometry:
O  1.748458  -1.269510   0.43785
B  1.064075   0.002710   0.03760
O  1.102931   0.107958  -1.45984
O  1.808429   1.164402   0.61414
C -0.486425   0.004486   0.55930
C -1.480511   1.077827   0.00691
C -2.592245  -0.001651  -0.05107
C -1.477830  -1.077058   0.01882
H  2.538979  -0.931673   0.88316
H  1.598655  -0.688390  -1.69778
H  2.200929   1.558383  -0.17842
H -0.533617   0.010746   1.66143
H -1.177331   1.393361  -0.99902
H -1.678453   1.975845   0.60968
H -3.278288  -0.007496  -0.91448
H -3.203492   0.002128   0.86345
H -1.672543  -1.971477   0.62767
H -1.171699  -1.399403  -0.98473
```

```
Molecule 58: k2cat_B3LYP - Transition State
#n B3LYP/6-31G(d) Opt=(TS,CalcFC,noeigentest)
E(RB3LYP) =  -704.24328797
Charge, multiplicity: -1, 1
Geometry:
C -1.830766  -0.414543  -1.40896
C -0.490481  -0.395944  -0.64288
B -0.474080   1.839315   0.15981
O -1.778845   2.303768  -0.10972
O -0.228274   1.688878   1.54627
H  0.657519   1.321309   1.67089
```

```
H -1.775719    2.592451   -1.03206
O  0.492997    2.415223   -0.71668
H  1.371046    2.068718   -0.50491
O  2.068913    0.319058    0.37401
B  3.016452   -0.538710   -0.10600
O  2.733935   -1.557929   -0.98707
C  4.524552   -0.353259    0.35770
H  1.784740   -1.566829   -1.20550
C -0.479923   -1.625214    0.29765
H  4.594555   -0.293193    1.45140
H  1.110967    0.182974    0.04233
H  0.266981   -0.555892   -1.44646
C -1.661781   -1.581182    1.28029
H  0.453645   -1.688514    0.87743
H -0.552689   -2.591033   -0.25341
C -2.992900   -1.610893    0.51692
H -1.591151   -0.654927    1.86445
H -1.612041   -2.426473    1.98533
C -3.068971   -0.456907   -0.49248
H -3.844226   -1.566899    1.21410
H -3.074545   -2.574805   -0.01417
H -3.129004    0.493573    0.04447
H -3.983878   -0.552786   -1.10055
H -1.891536   -1.301551   -2.07845
H -1.909792    0.459665   -2.07189
H  5.173403   -1.165153    0.01140
H  4.931674    0.592008   -0.02740
```

```
Molecule 58: k2cat_B3LYP - Intermediate
#n B3LYP/6-31G(d) Opt
E(RB3LYP) =  -487.72682843
Charge, multiplicity: -1, 1
Geometry:
```

```
O   2.251858   -1.354964    0.37798
B   1.658339   -0.014467    0.06722
O   1.943696    0.306752   -1.36913
O   2.309928    1.020742    0.92687
C   0.033674   -0.029538    0.34326
C  -0.687690   -1.177958   -0.38032
C  -2.181948   -1.309018    0.02147
C  -2.788232    0.044169    0.46275
C  -2.161808    1.202844   -0.32458
C  -0.638051    1.313551   -0.04012
H   2.934826   -1.122056    1.02304
H   2.467946   -0.459088   -1.64310
H   2.846774    1.504923    0.28283
H  -0.112509   -0.187098    1.42827
H  -0.162575   -2.121566   -0.18884
H  -0.611297   -1.002130   -1.46404
H  -2.770053   -1.714196   -0.81848
H  -2.293234   -2.032922    0.84294
H  -3.883586    0.030566    0.34988
H  -2.590998    0.203635    1.53223
H  -2.333077    1.021894   -1.39739
H  -2.679026    2.147779   -0.09466
H  -0.141349    1.716533   -0.93291
H  -0.463055    2.041526    0.76352
```

6.1.5 k_{2cat} (M06L)

```
Molecule 0: k2cat_M06L - Misc. small molecule
#n M06L/6-311++G**
E(RM06L) =  -216.566280085
Charge, multiplicity: 0, 1
Geometry:
C  -1.522933   -0.025843   -0.00006
```

```
B   0.051728   -0.004250   -0.00040
H  -1.918630    0.508459   -0.87295
H  -1.916799    0.494075    0.88240
H  -1.923838   -1.043453   -0.00756
O   0.681231    1.219966   -0.00006
H   1.648341    1.174392    0.00073
O   0.730603   -1.200368   -0.00001
H   1.695214   -1.113949    0.00038
```

```
Molecule 50: k2cat_M06L - Transition State
#n M06L/6-311++G**
E(RM06L) =  -1021.55532247
Charge, multiplicity: -1, 1
Geometry:
C  -1.784257   -0.438454   -1.02238
C  -0.853465   -0.183073   -0.05320
B   0.249293    1.942992   -0.12028
O   0.904163    1.872557   -1.33820
O  -0.905153    2.720781   -0.00773
H  -1.444338    2.554569   -0.79296
O   0.986819    1.821020    1.08629
H   0.810683   -0.544968   -0.51733
O   1.728791   -0.848855   -0.89411
C  -1.407429   -0.589792    1.22117
H   0.342124    1.854149    1.80801
H   1.451996    1.067759   -1.37736
B   2.754731   -0.960286    0.00568
O   2.798087   -0.292170    1.20144
H   2.087585    0.387560    1.25132
C   3.981349   -1.911640   -0.36408
H  -0.857052   -0.501863    2.15927
H  -1.710998   -0.276296   -2.09344
S  -3.307726   -1.144317   -0.42162
```

```
C -2.675009   -1.107619    1.19943
H -3.276624   -1.469308    2.02616
H  4.345335   -1.703427   -1.37928
H  3.664722   -2.964444   -0.36584
H  4.821277   -1.811389    0.33300
```

```
Molecule 50: k2cat_M06L - Intermediate
#n M06L/6-311++G**
E(RM06L) =  -804.980896527
Charge, multiplicity: -1, 1
Geometry:
O -2.194585   -0.753501   -1.12880
B -1.634600    0.038520    0.00480
O -2.164377   -0.530803    1.26941
O -2.043697    1.464725   -0.13607
C  0.010030   -0.059277    0.00686
C  0.770352   -1.285987   -0.00031
C  2.127964   -1.134744   -0.00337
S  2.566064    0.555680    0.00008
C  0.862242    1.007937    0.00782
H -2.568144   -0.067305   -1.69956
H -2.872749   -1.107605    0.94705
H -2.626074    1.584760    0.62728
H  0.291696   -2.262137   -0.00458
H  2.897775   -1.897744   -0.00878
H  0.591207    2.055610    0.01065
```

```
Molecule 51: k2cat_M06L - Transition State
#n M06L/6-311++G**
E(RM06L) =  -698.583820506
Charge, multiplicity: -1, 1
Geometry:
C -0.993251   -0.512266   -0.10630
B -0.392433    1.768019    0.01737
```

```
O  0.340750    1.974394   -1.13916
O -1.700318    2.254608    0.07630
H -2.108187    2.068699   -0.78113
O  0.273129    1.671083    1.26739
H  0.647683   -0.509726   -0.65049
O  1.630331   -0.582927   -0.99841
H -0.396530    1.374984    1.90229
H  1.031361    1.292844   -1.22912
B  2.607825   -0.603211   -0.04165
O  2.463691   -0.071278    1.21433
H  1.638546    0.461235    1.28987
C  4.010867   -1.272534   -0.40711
C -1.390771   -1.283054    0.97145
O -2.053612   -0.604737   -1.03290
C -3.038641   -1.390599   -0.51741
C -2.687346   -1.844065    0.71769
H -0.793850   -1.444747    1.86380
H -3.914923   -1.536485   -1.13726
H -3.277928   -2.492440    1.35566
H  3.898787   -2.360037   -0.52446
H  4.779330   -1.091563    0.35342
H  4.381833   -0.900832   -1.37187
```

```
Molecule 51: k2cat_M06L - Intermediate
#n M06L/6-311++G**
E(RM06L) =  -482.008135218
Charge, multiplicity: -1, 1
Geometry:
O  1.738162   -0.823932   -1.13465
B  1.310287    0.006947    0.03182
O  1.909146    1.342297   -0.16167
O  1.717573   -0.559716    1.32485
C -0.334209    0.120173    0.04587
```

```
C  -1.212928    1.173913    0.04518
C  -2.550472    0.653251    0.00494
C  -2.417022   -0.701888   -0.02068
O  -1.097220   -1.045562    0.00324
H   1.234567   -1.647883   -1.06498
H   1.997474    1.423946   -1.12200
H   2.684643   -0.487467    1.30511
H  -0.914412    2.213586    0.07336
H  -3.483256    1.206429   -0.00435
H  -3.123948   -1.520729   -0.05225
```

```
Molecule 52: k2cat_M06L - Transition State
#n M06L/6-311++G**
E(RM06L) =  -1021.56629684
Charge, multiplicity: -1, 1
Geometry:
C  -0.894282   -0.280251    0.02877
B   0.003466    1.898256    0.00223
O   0.673076    1.943185   -1.21105
O  -1.195489    2.602775    0.12703
H  -1.676955    2.514871   -0.70706
O   0.738045    1.762204    1.21055
H   0.789242   -0.536993   -0.54576
O   1.713872   -0.750983   -0.93463
H   0.083418    1.642774    1.91446
H   1.274842    1.183141   -1.29734
B   2.748786   -0.799262   -0.03627
O   2.733963   -0.175927    1.18219
H   1.966335    0.436250    1.26784
C   4.054390   -1.619004   -0.44496
C  -1.295815   -0.872577    1.21482
S  -2.201682   -0.574954   -1.11894
C  -3.211842   -1.438704    0.01214
```

```
C -2.576531   -1.517643    1.22234
H -0.660325   -0.853540    2.10187
H -4.181714   -1.839536   -0.26020
H -3.004259   -2.017831    2.09010
H  3.839911   -2.697077   -0.46675
H  4.890479   -1.454192    0.24423
H  4.381414   -1.354533   -1.45948
```

```
Molecule 52: k2cat_M06L - Intermediate
#n M06L/6-311++G**
E(RM06L) =  -804.986276554
Charge, multiplicity: -1, 1
Geometry:
O -1.996484    0.110802    1.38195
B -1.516607    0.059795   -0.03290
O -2.055630    1.147828   -0.86030
O -1.917341   -1.268912   -0.53414
C  0.130477    0.248181   -0.06600
C  0.937078    1.362985   -0.01947
C  2.344137    1.102480    0.03657
C  2.632176   -0.236391    0.03359
S  1.164005   -1.171101   -0.05399
H -1.649799    0.933286    1.75493
H -3.007373    0.963745   -0.88667
H -2.119521   -1.773709    0.26602
H  0.515715    2.365194   -0.03978
H  3.106050    1.879817    0.07170
H  3.606318   -0.710969    0.05381
```

```
Molecule 54: k2cat_M06L - Transition State
#n M06L/6-311++G**
E(RM06L) =  -547.108493488
Charge, multiplicity: -1, 1
Geometry:
```

```
C  1.044855   1.331894  -0.77217
B  1.605721  -0.718222   0.08866
O  1.005089  -1.656313  -0.74312
O  2.996404  -0.558065  -0.03506
H  3.215427  -0.786367  -0.94898
O  1.085074  -0.498101   1.39540
H -0.260716   0.465906  -0.86930
O -1.235696  -0.037801  -1.04945
H  1.506512   0.313812   1.71553
H  0.072047  -1.403780  -0.89100
B -2.094252  -0.081642   0.00552
O -1.695031  -0.051867   1.32335
H -0.715820  -0.133294   1.38815
C -3.668111  -0.183727  -0.27385
C  0.969167   2.403607   0.03808
H  1.650651   1.508742  -1.67849
H  0.374178   2.372780   0.95943
H  1.456213   3.373358  -0.13615
H -4.253332  -0.370823   0.63472
H -4.037931   0.746821  -0.72883
H -3.886765  -0.981288  -0.99727
```

```
Molecule 54: k2cat_M06L - Intermediate
#n M06L/6-311++G**
E(RM06L) =  -330.538233263
Charge, multiplicity: -1, 1
Geometry:
C -2.275340   0.046478   0.00078
C -1.124154  -0.637824  -0.00086
B  0.390469  -0.030097   0.00235
O  1.077849  -0.405043   1.27625
O  0.378567   1.457582  -0.13682
O  1.171032  -0.609203  -1.13827
```

```
H -2.264452    1.137393    0.00757
H -3.267031   -0.420168   -0.00707
H -1.216930   -1.734836   -0.01079
H  1.918358   -0.751923    0.94126
H  0.832235    1.732231    0.67256
H  1.422861    0.189172   -1.62426
```

```
Molecule 55: k2cat_M06L - Transition State
#n M06L/6-311++G**
E(RM06L) =  -586.421329279
Charge, multiplicity: -1, 1
Geometry:
C  1.747634    1.958274    0.20556
C  0.808802    0.969094   -0.44336
B  1.695973   -0.922435   -0.02359
O  3.044295   -0.686364   -0.38579
O  1.525678   -1.062984    1.38191
H  0.582097   -1.197346    1.55007
H  3.082875   -0.834213   -1.34019
O  1.033063   -1.805930   -0.93496
H  0.089512   -1.826672   -0.71570
O -1.232140   -0.652344    0.34207
B -2.424131   -0.113695   -0.02604
O -2.519503    1.129806   -0.62017
C -3.773038   -0.928864    0.20398
H -1.624234    1.512877   -0.67840
C  0.281524    2.197822    0.36511
H  2.308704    2.663294   -0.41190
H  2.308639    1.595480    1.06512
H -0.146101    3.068389   -0.14116
H -0.175101    2.008614    1.33823
H -3.846137   -1.757198   -0.51536
H -0.392824   -0.052495    0.08572
```

```
H  0.715686   1.136456  -1.52568
H -4.667281  -0.306356   0.08340
H -3.795718  -1.385617   1.20156
```

```
Molecule 55: k2cat_M06L - Intermediate
#n M06L/6-311++G**
E(RM06L) =  -369.854177331
Charge, multiplicity: -1, 1
Geometry:
O  0.710401  -0.106529   1.45522
B  0.732657   0.038176  -0.02475
O  1.411619   1.291478  -0.41741
O  1.472889  -1.174937  -0.52120
C -0.789505   0.101539  -0.64485
C -1.889084  -0.758623  -0.05768
C -1.913267   0.738770   0.14403
H  0.905465  -1.043530   1.59597
H  2.234947   1.265493   0.09454
H  1.501927  -1.079636  -1.48366
H -0.882226   0.249392  -1.72767
H -1.628267  -1.375522   0.80161
H -2.649983  -1.201096  -0.70458
H -1.662150   1.114546   1.13403
H -2.691131   1.309257  -0.36842
```

```
Molecule 56: k2cat_M06L - Transition State
#n M06L/6-311++G**
E(RM06L) =  -509.019129698
Charge, multiplicity: -1, 1
Geometry:
C -1.080068   1.427743  -1.16728
B -1.680084  -0.264835   0.29482
O -1.176729   0.092045   1.54298
O -3.066196  -0.115151   0.09345
```

```
H -3.330575    0.666981    0.59641
O -1.119032   -1.395099   -0.37852
H  0.173901    0.992385   -0.27759
O  1.132737    0.957513    0.28628
H -1.534971   -1.430951   -1.25153
H -0.233964    0.328206    1.44305
B  2.016548   -0.023749   -0.03434
O  1.658469   -1.221727   -0.61167
H  0.676024   -1.299961   -0.62570
C  3.573418    0.194597    0.27963
H  4.185240   -0.685133    0.04494
H  3.969323    1.048087   -0.28948
H  3.724456    0.443438    1.33965
H -0.300545    1.958521   -1.74817
H -1.695047    0.920450   -1.92706
H -1.710250    2.226206   -0.74090
```

```
Molecule 56: k2cat_M06L - Intermediate
#n M06L/6-311++G**
E(RM06L) =  -292.451137772
Charge, multiplicity: -1, 1
Geometry:
C -1.606124   -0.118011    0.02260
B  0.044850    0.004461   -0.03041
O  0.661655   -1.049033   -0.88085
O  0.522585   -0.123666    1.38832
O  0.488786    1.293079   -0.62734
H -2.042050   -0.060042   -0.98744
H -2.083083    0.678303    0.62305
H -1.949469   -1.070652    0.46308
H  0.587550   -1.843488   -0.33362
H  1.473251    0.053502    1.30351
H  0.042089    1.965103   -0.09330
```

```
Molecule 57: k2cat_M06L - Transition State
#n M06L/6-311++G**
E(RM06L) =  -625.730101498
Charge, multiplicity: -1, 1
Geometry:
C  0.864467    2.158028   -0.63079
C  1.199339    0.617551   -0.49899
B  0.847790   -1.639801   -0.10861
O  0.036971   -2.115171   -1.13786
O  2.214500   -1.979042   -0.16714
H  2.447309   -2.018470   -1.10458
O  0.327057   -1.588668    1.22099
H -0.392675    0.227155   -0.65468
O -1.449395    0.209694   -0.91919
C  2.078026    1.027241    0.72969
H  1.019516   -1.208201    1.78027
H -0.746886   -1.539553   -1.21730
B -2.357199    0.222214    0.09719
O -2.079257   -0.196480    1.37648
H -1.192085   -0.625663    1.40362
C -3.843495    0.734033   -0.20349
C  2.108554    2.489195    0.22494
H -4.507997    0.647573    0.66457
H -0.063111    2.425014   -0.10410
H  0.805597    2.591805   -1.63902
H  1.883511    0.389016   -1.33364
H  3.035809    0.512080    0.87867
H  1.508130    0.960997    1.67320
H  2.989923    2.667363   -0.40686
H  2.044208    3.308876    0.96171
H -3.836009    1.784652   -0.52663
H -4.288541    0.166334   -1.03286
```

```
Molecule 57: k2cat_M06L - Intermediate
#n M06L/6-311++G**
E(RM06L) =  -409.168639743
Charge, multiplicity: -1, 1
Geometry:
O  1.748458   1.269510  -0.43785
B  1.064075  -0.002710  -0.03760
O  1.102931  -0.107958   1.45983
O  1.808429  -1.164402  -0.61414
C -0.486425  -0.004486  -0.55930
C -1.480511  -1.077827  -0.00691
C -2.592245   0.001651   0.05106
C -1.477830   1.077058  -0.01882
H  2.538979   0.931673  -0.88316
H  1.598655   0.688390   1.69777
H  2.200929  -1.558383   0.17841
H -0.533617  -0.010746  -1.66143
H -1.177331  -1.393361   0.99901
H -1.678453  -1.975845  -0.60968
H -3.278288   0.007496   0.91447
H -3.203492  -0.002128  -0.86345
H -1.672543   1.971477  -0.62767
H -1.171699   1.399403   0.98472
```

```
Molecule 58: k2cat_M06L - Transition State
#n M06L/6-311++G**
E(RM06L) =  -704.392587957
Charge, multiplicity: -1, 1
Geometry:
C -1.830766  -0.414543  -1.40896
C -0.490481  -0.395944  -0.64288
B -0.474080   1.839315   0.15981
O -1.778845   2.303768  -0.10972
```

```
O -0.228274    1.688878    1.54627
H  0.657519    1.321309    1.67089
H -1.775719    2.592451   -1.03206
O  0.492997    2.415223   -0.71668
H  1.371046    2.068718   -0.50491
O  2.068913    0.319058    0.37401
B  3.016452   -0.538710   -0.10600
O  2.733935   -1.557929   -0.98707
C  4.524552   -0.353259    0.35770
H  1.784740   -1.566829   -1.20550
C -0.479923   -1.625214    0.29765
H  4.594555   -0.293193    1.45140
H  1.110967    0.182974    0.04233
H  0.266981   -0.555892   -1.44646
C -1.661781   -1.581182    1.28029
H  0.453645   -1.688514    0.87743
H -0.552689   -2.591033   -0.25341
C -2.992900   -1.610893    0.51692
H -1.591151   -0.654927    1.86445
H -1.612041   -2.426473    1.98533
C -3.068971   -0.456907   -0.49248
H -3.844226   -1.566899    1.21410
H -3.074545   -2.574805   -0.01417
H -3.129004    0.493573    0.04447
H -3.983878   -0.552786   -1.10055
H -1.891536   -1.301551   -2.07845
H -1.909792    0.459665   -2.07189
H  5.173403   -1.165153    0.01140
H  4.931674    0.592008   -0.02740
```

```
Molecule 58: k2cat_M06L - Intermediate
#n M06L/6-311++G**
E(RM06L) =  -487.832853438
```

```
Charge, multiplicity: -1, 1
Geometry:
O  2.251858   1.354964  -0.37798
B  1.658339   0.014467  -0.06722
O  1.943696  -0.306752   1.36912
O  2.309928  -1.020742  -0.92687
C  0.033674   0.029538  -0.34326
C -0.687690   1.177958   0.38031
C -2.181948   1.309018  -0.02147
C -2.788232  -0.044169  -0.46275
C -2.161808  -1.202844   0.32457
C -0.638051  -1.313551   0.04011
H  2.934826   1.122056  -1.02304
H  2.467946   0.459088   1.64309
H  2.846774  -1.504923  -0.28283
H -0.112509   0.187098  -1.42827
H -0.162575   2.121566   0.18883
H -0.611297   1.002130   1.46403
H -2.770053   1.714196   0.81847
H -2.293234   2.032922  -0.84294
H -3.883586  -0.030566  -0.34988
H -2.590998  -0.203635  -1.53223
H -2.333077  -1.021894   1.39738
H -2.679026  -2.147779   0.09465
H -0.141349  -1.716533   0.93290
H -0.463055  -2.041526  -0.76352
```

6.1.6 k_3

```
Molecule 0: k3 - Misc. small molecule
#n M06L/6-311++G** SCRF=(Solvent=Water) Opt
E(RM06L) = -0.164563614639
Charge, multiplicity: 1, 1
```

```
Geometry:
H  0.000000    0.000000    0.00000
```

```
Molecule 60: k3 - Transition State
#n M06L/6-311++G** SCRF=(Solvent=Water) Opt=(TS,CalcFC,noeigentest)
E(RM06L) =  -477.736184489
Charge, multiplicity: -2, 1
Geometry:
C -1.182767   -1.108638   -0.00346
N -2.481293   -0.768523   -0.01857
N -2.441941    0.578800    0.00107
C -1.165035    1.058934    0.02207
H -3.305811    1.099409   -0.01163
C -0.284341   -0.012717    0.02465
H -0.934443   -2.165092   -0.01712
B  1.365282   -0.014943    0.15518
O  1.885241   -0.309533    1.40220
O  1.851333    1.389215   -0.32890
H  1.502849    1.521810   -1.21436
O  1.821281   -1.019550   -0.95512
H  2.692845   -1.286322   -0.64915
H -0.989196    2.126435    0.03377
```

```
Molecule 60: k3 - Intermediate
#n M06L/6-311++G** SCRF=(Solvent=Water) Opt
E(RM06L) =  -478.281086887
Charge, multiplicity: -1, 1
Geometry:
C -0.315140   -0.026593   -0.00085
C -1.182461    1.091870   -0.01143
N -2.483328    0.774634   -0.02025
N -2.470581   -0.572947   -0.01420
C -1.210548   -1.083688   -0.00262
H -3.344930   -1.075908   -0.01419
```

```
H -0.908512    2.141478   -0.01310
B  1.305034   -0.034467   -0.00549
O  1.894450    0.567137   -1.23226
O  1.842312    0.789036    1.12608
H  1.794112    1.519971   -1.17952
H  1.355818    0.575387    1.92477
O  1.745002   -1.442739    0.05806
H  2.704516   -1.444405    0.02082
H -1.054032   -2.153005    0.00432
```

```
Molecule 61: k3 - Transition State
#n M06L/6-311++G** SCRF=(Solvent=Water) Opt=(TS,CalcFC,noeigentest)
E(RM06L) =  -576.232903430
Charge, multiplicity: -2, 1
Geometry:
C -0.942017   -1.094365    0.01547
O -2.264210   -0.756569   -0.02287
N -2.349194    0.641314   -0.02419
C -1.092528    1.060260    0.00933
C -0.127819    0.002260    0.04227
B  1.540367   -0.035444    0.14301
O  2.065027   -0.782109    1.17483
O  2.042009    1.435418    0.20722
H  1.730133    1.891826   -0.57853
O  1.946531   -0.543377   -1.28526
H  2.869555   -0.791778   -1.17774
C -0.866755    2.534235    0.00492
C -0.650006   -2.543994    0.02021
H -1.817667    3.071242    0.03032
H -0.324990    2.850667   -0.89153
H -0.261169    2.843770    0.85878
H -0.895717   -3.016171   -0.93741
H -1.214377   -3.072947    0.79448
```

```
H  0.416652   -2.665868    0.21121
```

```
Molecule 61: k3 - Intermediate
#n M06L/6-311++G** SCRF=(Solvent=Water) Opt
E(RM06L) =  -576.777574185
Charge, multiplicity: -1, 1
Geometry:
C  0.167641   -0.031643    0.01440
C  0.847492    1.227471   -0.00225
N  2.161420    1.115179   -0.01409
O  2.406757   -0.265141   -0.00789
C  1.208681   -0.915098    0.01021
C  0.237314    2.583599   -0.01369
B -1.439850   -0.235523    0.02484
O -2.107384    0.432341   -1.13442
O -1.711548   -1.697505    0.00179
H -1.901775   -0.059964   -1.93157
H -2.664229   -1.815351    0.03201
O -1.998303    0.430580    1.22532
H -2.876618    0.744605    0.99855
C  1.306223   -2.392015    0.02188
H -0.464326    2.692357    0.81611
H -0.339654    2.736492   -0.92908
H  0.999681    3.361602    0.05418
H  0.303878   -2.812995    0.06566
H  1.882358   -2.746031    0.88216
H  1.809706   -2.765433   -0.87533
```

6.1.7 k_4

```
Molecule 0: k4 - Misc. small molecule
#n M06L/6-311++G** SCRF=(Solvent=Water) Opt
E(RM06L) =  -76.4487444811
```

```
Charge, multiplicity: 0, 1
Geometry:
O  0.000000   0.000000   0.11889
H -0.000000  -0.755103  -0.47558
H  0.000000   0.755103  -0.47558
```

```
Molecule 44: k4 - Transition State
#n M06L/6-311++G** SCRF=(Solvent=Water) Opt=(TS,CalcFC,noeigentest)
E(RM06L) =  -500.828814494
Charge, multiplicity: 0, 1
Geometry:
C  2.861946  -0.084099   0.00592
C  2.059729  -1.201662   0.00802
N  0.721908  -1.046701  -0.00184
C  0.062909   0.137097  -0.01310
C  0.869225   1.273710  -0.00774
C  2.253064   1.171550  -0.00015
H  3.937664  -0.195742   0.01348
H  2.435309  -2.215876   0.02000
H  0.386431   2.244245  -0.00578
H  2.867390   2.065825   0.00187
B -1.582784   0.026654  -0.00769
O -1.980148  -0.899020   1.06382
O -2.205751   1.329577   0.23600
H -2.000254  -0.436718   1.90469
H -2.094486   1.897919  -0.52881
O -1.892332  -0.584813  -1.30291
H -2.819719  -0.835889  -1.30586
H  0.132847  -1.875664   0.01888
```

```
Molecule 44: k4 - Reactant
#n M06L/6-311++G** SCRF=(Solvent=Water) Opt
E(RM06L) =  -424.378135228
Charge, multiplicity: 0, 1
```

```
Geometry:
O  2.320023  -1.223749   0.00001
B  1.743443   0.009658   0.00008
O  2.589139   1.079956   0.00004
C  0.171219   0.078842  -0.00000
C -0.591280   1.248288  -0.00006
C -1.979352   1.167214   0.00003
C -2.571159  -0.088116   0.00009
C -1.746167  -1.210475  -0.00006
N -0.415699  -1.143302  -0.00008
H  1.601320  -1.874992   0.00014
H  2.142998   1.929395  -0.00026
H -0.108700   2.221550  -0.00039
H -2.586851   2.066082   0.00017
H -3.649169  -0.202995   0.00016
H -2.179786  -2.208388  -0.00012
```

```
Molecule 60: k4 - Transition State
#n M06L/6-311++G** SCRF=(Solvent=Water) Opt=(TS,CalcFC,noeigentest)
E(RM06L) =  -478.728966888
Charge, multiplicity: 0, 1
Geometry:
C -0.273729   0.000207  -0.01056
C -1.138411  -1.093190  -0.00640
N -2.414047  -0.670449   0.00266
C -1.138696   1.093382  -0.00644
N -2.414223   0.670300   0.00264
H -3.282519   1.188219  -0.00557
H -0.938598  -2.153889  -0.01445
B  1.370719   0.000008  -0.02022
H -0.939371   2.154170  -0.01445
O  1.902733  -1.163298  -0.75088
O  1.903187   1.162724  -0.75146
```

```
H  1.743157   -1.956583   -0.23693
H  1.743154    1.956455   -0.23840
O  1.755348    0.000259    1.41042
H  2.715529    0.001401    1.45549
H -3.282187   -1.188640   -0.00575
```

```
Molecule 60: k4 - Reactant
#n M06L/6-311++G** SCRF=(Solvent=Water) Opt
E(RM06L) =  -402.305197822
Charge, multiplicity: 0, 1
Geometry:
O  2.248202   -1.131228    0.04472
B  1.490065    0.010486    0.00034
O  2.245278    1.154216   -0.04314
C -0.056007   -0.000360   -0.00064
C -0.939050   -1.109593   -0.04063
N -2.220899   -0.758971   -0.02797
N -2.180552    0.589865    0.02189
C -0.929208    1.081786    0.03902
H  1.728904   -1.936059    0.09186
H  1.724878    1.957966   -0.09392
H -0.692337   -2.162959   -0.08216
H -3.045622    1.109741    0.04478
H -0.758241    2.147714    0.08127
```

```
Molecule 61: k4 - Transition State
#n M06L/6-311++G** SCRF=(Solvent=Water) Opt=(TS,CalcFC,noeigentest)
E(RM06L) =  -577.221144891
Charge, multiplicity: 0, 1
Geometry:
C  0.131002    0.037903   -0.00863
C  0.910657   -1.137684   -0.00472
N  2.193315   -0.789773   -0.00030
C  1.060405    1.047485   -0.00009
```

```
O  2.335356   0.559579   0.00481
C  0.488293  -2.554322   0.00156
C  0.969998   2.516710   0.00244
B -1.506229   0.080839   0.00829
H -0.598172  -2.594238   0.02258
H  0.892746  -3.079464   0.86941
H  0.845354  -3.071010  -0.89208
H -0.075792   2.797544   0.10843
H  1.361471   2.934144  -0.92895
H  1.550272   2.943899   0.82317
O -1.926323  -0.523064   1.28434
O -2.057610   1.449942  -0.03131
H -2.790903  -0.170810   1.50791
H -2.091270   1.746442  -0.94306
O -1.911018  -0.701687  -1.18320
H -2.864669  -0.815996  -1.16703
H  3.063537  -1.305007   0.01987
```

```
Molecule 61: k4 - Reactant
#n M06L/6-311++G** SCRF=(Solvent=Water) Opt
E(RM06L) =  -500.799577832
Charge, multiplicity: 0, 1
Geometry:
C  0.400669  -2.583369  -0.00221
C  0.787005  -1.147657  -0.00070
N  2.062238  -0.835197  -0.00038
O  2.096970   0.570407   0.00065
C  0.840439   1.043339   0.00070
C  0.709650   2.513621   0.00172
C -0.060964   0.006827  -0.00006
B -1.604455   0.091357  -0.00002
O -2.436869  -0.998017   0.00463
O -2.195787   1.325571  -0.00496
```

```
H  1.285083   -3.220116   -0.00265
H -0.189543   -2.833905   -0.88844
H -0.189708   -2.835864    0.88337
H  1.194179    2.944482   -0.87806
H -0.339642    2.797056    0.00295
H  1.196211    2.943479    0.88087
H -1.989294   -1.847241    0.00929
H -3.155981    1.261442   -0.00386
```

```
Molecule 62: k4 - Transition State
#n M06L/6-311++G** SCRF=(Solvent=Water) Opt=(TS,CalcFC,noeigentest)
E(RM06L) =  -516.854461734
Charge, multiplicity: 0, 1
Geometry:
C  2.804373    0.099223   -0.00374
N  2.153814   -1.079381   -0.00110
C  0.805754   -1.137691    0.00378
C  0.054520    0.024106    0.00190
C  0.824991    1.191964    0.00671
N  2.169081    1.246080    0.00292
H  0.378305   -2.133465    0.01912
B -1.600937   -0.000619    0.02072
H  0.330597    2.161595    0.02507
H  2.695480   -1.938849    0.00132
O -2.078732   -1.126351    0.83838
O -2.130390    1.202879    0.67324
H -2.046997    1.952304    0.08126
H -2.086407   -1.922610    0.30491
O -1.968870   -0.115361   -1.40159
H -2.927425   -0.098936   -1.47130
H  3.886968    0.061207   -0.00893
```

```
Molecule 62: k4 - Reactant
#n M06L/6-311++G** SCRF=(Solvent=Water) Opt
```

```
E(RM06L) =  -440.421824262
Charge, multiplicity: 0, 1
Geometry:
O  2.322470   1.217441   0.00071
B  1.717572  -0.004360   0.00001
O  2.504349  -1.119701  -0.00065
C  0.153872  -0.020454   0.00002
C -0.620849  -1.181501   0.00043
N -1.956610  -1.184364   0.00041
C -2.531711   0.019065  -0.00004
N -1.921893   1.206100  -0.00042
C -0.587103   1.165688  -0.00037
H  3.282664   1.152536   0.00059
H  2.029894  -1.954456  -0.00135
H -0.155225  -2.167732   0.00087
H -3.620109   0.034100  -0.00010
H -0.075375   2.126499  -0.00068
```

```
Molecule 63: k4 - Transition State
#n M06L/6-311++G** SCRF=(Solvent=Water) Opt=(TS,CalcFC,noeigentest)
E(RM06L) =  -500.817403330
Charge, multiplicity: 0, 1
Geometry:
C  2.858408  -0.002190   0.00648
N  2.129251  -1.132764   0.03475
C  0.779392  -1.138355   0.03818
C  0.050069   0.038651   0.00544
C  0.805425   1.218169  -0.02333
C  2.198454   1.207130  -0.02081
H  0.321220  -2.119537   0.07729
H  2.776838   2.122044  -0.04024
B -1.588216   0.023750  -0.00239
H  0.281116   2.169309  -0.03912
```

```
H  2.618610   -2.020900    0.05931
O -2.137056   -1.063629    0.84035
O -2.037012    1.300555    0.56938
H -2.874161    1.147528    1.01180
H -2.245900   -1.850720    0.30331
O -1.953781   -0.164430   -1.42586
H -2.901982   -0.041384   -1.52017
H  3.932888   -0.116135    0.00977
```

```
Molecule 63: k4 - Reactant
#n M06L/6-311++G** SCRF=(Solvent=Water) Opt
E(RM06L) =  -424.377268537
Charge, multiplicity: 0, 1
Geometry:
O -2.346730   -1.208762   -0.00047
B -1.727444    0.008520    0.00001
O -2.513982    1.127427    0.00036
C -0.166040    0.030642    0.00003
C  0.588585    1.208714   -0.00022
C  1.974987    1.145741   -0.00024
C  2.577822   -0.106826   -0.00001
N  1.894798   -1.257939    0.00025
C  0.561735   -1.167390    0.00027
H -3.305643   -1.128940   -0.00047
H -2.028959    1.956275    0.00098
H  0.103953    2.182676   -0.00041
H  2.580977    2.044642   -0.00049
H  3.662605   -0.192359   -0.00003
H  0.023863   -2.113921    0.00047
```

```
Molecule 64: k4 - Transition State
#n M06L/6-311++G** SCRF=(Solvent=Water) Opt=(TS,CalcFC,noeigentest)
E(RM06L) =  -500.818414289
Charge, multiplicity: 0, 1
```

```
Geometry:
N   2.802047   -0.004622   -0.00277
C   2.152483   -1.185455    0.03520
C   0.777240   -1.199869    0.05652
C   0.033727   -0.008213    0.04257
C   0.774489    1.186848    0.00340
C   2.147677    1.176718   -0.01948
H   0.263870   -2.153843    0.09217
H   2.767001    2.062983   -0.04733
B  -1.598217    0.016621    0.00973
H   0.263216    2.142941   -0.00201
H   2.774371   -2.070442    0.05006
O  -2.052076    1.149691    0.83317
O  -2.036072    0.282857   -1.38526
H  -1.767921   -0.445120   -1.95151
H  -2.784918    0.858319    1.37673
O  -2.073568   -1.287668    0.49774
H  -2.973913   -1.421765    0.19376
H   3.815078   -0.003031   -0.01578
```

```
Molecule 64: k4 - Reactant
#n M06L/6-311++G** SCRF=(Solvent=Water) Opt
E(RM06L) =  -424.376256106
Charge, multiplicity: 0, 1
Geometry:
O   2.360683    1.212632   -0.00054
B   1.740078   -0.001611   -0.00000
O   2.514811   -1.126305    0.00051
C   0.172783   -0.012673    0.00000
C  -0.579980   -1.191303   -0.00027
C  -1.968316   -1.128281   -0.00030
N  -2.657288    0.017924    0.00001
C  -1.942380    1.149581    0.00029
```

```
C -0.553904    1.183735    0.00026
H  3.319807    1.134010   -0.00059
H  2.020709   -1.950091    0.00097
H -0.104816   -2.169054   -0.00061
H -2.557773   -2.042776   -0.00053
H -2.513681    2.075717    0.00055
H -0.036782    2.137799    0.00047
```

```
Molecule 65: k4 - Transition State
#n M06L/6-311++G** SCRF=(Solvent=Water) Opt=(TS,CalcFC,noeigentest)
E(RM06L) =  -632.392892636
Charge, multiplicity: 0, 1
Geometry:
N -2.040673    1.576318    0.00071
C -1.893988    0.245190   -0.00905
C -2.904006   -0.841552   -0.01756
N -2.124892   -2.076842   -0.01241
H -3.555416   -0.808153   -0.89770
H -3.568156   -0.809447    0.85300
C -0.875613   -1.758116   -0.00645
C -0.625798   -0.317659   -0.00553
C  0.543182    0.457967    0.00119
C  0.313904    1.845873    0.01218
C -0.953499    2.386147    0.01353
H -0.090076   -2.507380   -0.00667
B  2.043738   -0.204109    0.02131
H  1.159159    2.525537    0.01565
H -1.158620    3.447920    0.02251
O  2.268280   -0.651039    1.40786
O  3.084134    0.782460   -0.31823
H  2.751572   -1.479187    1.38593
H  2.974618    1.075729   -1.22583
O  2.006044   -1.322927   -0.94551
```

```
H  2.900021   -1.488077   -1.25386
H -2.965584    1.992215   -0.00044
```

```
Molecule 65: k4 - Reactant
#n M06L/6-311++G** SCRF=(Solvent=Water) Opt
E(RM06L) =  -555.976484667
Charge, multiplicity: 0, 1
Geometry:
O -2.242425   -1.712572    0.00046
B -2.186068   -0.345883    0.00000
C -0.779930    0.333253   -0.00000
C  0.433817   -0.382349   -0.00007
C  1.642164    0.373820   -0.00005
N  2.686981   -0.513317   -0.00024
C  2.186048   -1.793196   -0.00003
C  0.815364   -1.757991   -0.00009
N  1.762666    1.696747    0.00008
C  0.595049    2.351552    0.00019
C -0.655690    1.726923    0.00010
O -3.279449    0.477309   -0.00044
H -3.130439   -2.080761    0.00048
H  3.662921   -0.260837    0.00048
H  2.859091   -2.637042   -0.00001
H  0.160878   -2.614715   -0.00030
H  0.660201    3.437308    0.00029
H -1.547625    2.344995    0.00014
H -4.128163    0.026499   -0.00032
```

```
Molecule 66: k4 - Transition State
#n M06L/6-311++G** SCRF=(Solvent=Water) Opt=(TS,CalcFC,noeigentest)
E(RM06L) =  -821.587120276
Charge, multiplicity: 0, 1
Geometry:
C  0.100146   -0.219423   -0.00006
```

```
C   0.890270   -1.329593   -0.00004
N   2.238870   -1.051949   -0.00000
C   2.540896    0.238103    0.00001
S   1.136097    1.171962   -0.00003
B  -1.545944   -0.063048   -0.00000
H   0.573239   -2.362031   -0.00005
H   3.556208    0.603012    0.00005
O  -2.135304   -0.741119   -1.15613
O  -2.135015   -0.740870    1.15641
H  -1.836159   -0.312505   -1.95980
H  -1.835904   -0.311919    1.95991
O  -1.735123    1.401067   -0.00011
H  -2.674951    1.596051   -0.00032
H   2.953310   -1.772266    0.00001
```

```
Molecule 66: k4 - Reactant
#n M06L/6-311++G** SCRF=(Solvent=Water) Opt
E(RM06L) =  -745.147640261
Charge, multiplicity: 0, 1
Geometry:
O  -2.313323   -1.157729   -0.16558
B  -1.658554    0.030394   -0.00513
O  -2.490670    1.103036    0.15102
C  -0.110453    0.177527   -0.00511
C   0.642991    1.324515   -0.10626
N   2.000672    1.191119   -0.10104
C   2.319487   -0.071061    0.00564
S   0.988870   -1.158726    0.10332
H  -1.741046   -1.911024   -0.32471
H  -2.034891    1.930601    0.31695
H   0.224545    2.321241   -0.20232
H   3.337323   -0.439363    0.04067
```

```
Molecule 67: k4 - Transition State
```

```
#n M06L/6-311++G** SCRF=(Solvent=Water) Opt=(TS,CalcFC,noeigentest)
E(RM06L) =  -518.053145046
Charge, multiplicity: 0, 1
Geometry:
C -0.177533   -0.367778   -0.02230
N -1.105314    0.611541   -0.04360
N -2.351464    0.075301   -0.00180
C -2.246511   -1.262072    0.02210
C -0.901978   -1.572253    0.01325
H -3.133921   -1.874262    0.03625
C -0.927862    2.051408   -0.01205
H -0.469334   -2.558565    0.02808
B  1.461212   -0.113743    0.02125
H  0.103558    2.246133   -0.29970
H -1.127297    2.439558    0.98859
H -1.598465    2.524516   -0.72925
O  1.843766    0.402574    1.33710
O  2.120058   -1.414539   -0.11853
H -3.176172    0.658658   -0.04093
H  1.474766    1.276497    1.47779
H  2.407284   -1.532341   -1.02430
O  1.846335    0.866218   -1.01453
H  1.452998    0.650776   -1.86327
```

```
Molecule 67: k4 - Reactant
#n M06L/6-311++G** SCRF=(Solvent=Water) Opt
E(RM06L) =  -441.615516227
Charge, multiplicity: 0, 1
Geometry:
C -0.769907    2.049418   -0.16978
N -0.946162    0.615795   -0.05469
N -2.195029    0.121810   -0.01667
C -2.036541   -1.200462    0.09227
```

```
C -0.684656   -1.550358    0.11504
C  0.020061   -0.351835    0.02116
B  1.558865   -0.129914    0.01747
O  2.188714    1.057679    0.26425
O  2.416519   -1.165275   -0.23115
H -0.447460    2.485552    0.78016
H -1.729841    2.485131   -0.43558
H -0.048422    2.285677   -0.95311
H -2.906835   -1.838981    0.14465
H -0.266464   -2.542531    0.20156
H  1.606094    1.775199    0.51945
H  1.971327   -1.983525   -0.46178
```

```
Molecule 68: k4 - Transition State
#n M06L/6-311++G** SCRF=(Solvent=Water) Opt=(TS,CalcFC,noeigentest)
E(RM06L) =  -478.739101892
Charge, multiplicity: 0, 1
Geometry:
C -0.298274    0.108693   -0.01270
N -1.070991   -0.989097   -0.00094
N -2.383013   -0.666801    0.00677
C -2.482702    0.669198   -0.00206
C -1.199854    1.185755   -0.01448
H -3.452889    1.139109   -0.00053
H -0.938509    2.231123   -0.02787
B  1.352813    0.021731   -0.02139
H -0.791734   -1.960741   -0.01480
H -3.103820   -1.374910   -0.00001
O  1.803409   -0.971501   -1.00717
O  1.919758    1.299837   -0.45348
H  1.916198   -1.814879   -0.56626
H  1.683215    1.987796    0.17150
O  1.694132   -0.354323    1.36252
```

```
H  2.648085   -0.298843    1.46466
```

```
Molecule 68: k4 - Reactant
#n M06L/6-311++G** SCRF=(Solvent=Water) Opt
E(RM06L) =  -402.307780125
Charge, multiplicity: 0, 1
Geometry:
O -2.273292    1.117377   -0.00036
B -1.451246    0.027795    0.00017
O -2.004538   -1.220670    0.00037
C  0.095563    0.107841    0.00045
C  1.002288    1.162909    0.00040
C  2.271648    0.570597   -0.00008
N  2.192162   -0.764278   -0.00038
N  0.875722   -1.009704   -0.00021
H -1.812107    1.959704   -0.00119
H -2.966478   -1.200454   -0.00136
H  0.772558    2.217836    0.00108
H  3.242084    1.046245   -0.00034
H  0.550635   -1.966173    0.00045
```

```
Molecule 69: k4 - Transition State
#n M06L/6-311++G** SCRF=(Solvent=Water) Opt=(TS,CalcFC,noeigentest)
E(RM06L) =  -615.377520579
Charge, multiplicity: 0, 1
Geometry:
C  1.978409    1.204160   -0.01136
C  1.642253   -0.141523   -0.00710
N  0.334208   -0.482298   -0.02337
C -0.708329    0.382751   -0.03249
C -0.386581    1.733661   -0.03321
C  0.944469    2.134776   -0.02904
H  3.014331    1.511226   -0.00509
O  2.461216   -1.182749    0.00705
```

```
H -1.187307    2.462601   -0.03929
H  1.195289    3.190690   -0.03772
B -2.189111   -0.329716    0.02008
C  3.869796   -0.912049    0.02845
H  4.352350   -1.884787    0.03986
H  4.138085   -0.352479    0.92677
H  4.167083   -0.359253   -0.86489
O -2.017450   -1.761253   -0.34173
O -2.614804   -0.270593    1.42591
H -1.992553   -1.849640   -1.29771
H -3.539724   -0.022692    1.46685
O -3.051139    0.398794   -0.90788
H -3.870907   -0.084980   -1.03342
H  0.066754   -1.470268   -0.05040
```

```
Molecule 69: k4 - Reactant
#n M06L/6-311++G** SCRF=(Solvent=Water) Opt
E(RM06L) =  -538.932525887
Charge, multiplicity: 0, 1
Geometry:
C  2.529207   -1.879021   -0.00043
O  2.627420   -0.454429    0.00007
C  1.469980    0.235603    0.00029
C  1.576593    1.636555    0.00011
C  0.408912    2.368142    0.00001
C -0.819361    1.698518   -0.00011
C -0.834966    0.310401   -0.00005
B -2.123810   -0.588555    0.00001
O -3.408959   -0.130264   -0.00031
O -1.959529   -1.940911    0.00042
N  0.318527   -0.413086    0.00023
H  3.554738   -2.241762   -0.00057
H  2.003521   -2.235882    0.88783
```

```
H  2.003508   -2.235156   -0.88903
H  2.554185    2.103620    0.00011
H  0.447044    3.452401   -0.00001
H -1.744593    2.266200   -0.00022
H -3.475775    0.826935   -0.00059
H -1.006906   -2.118334    0.00037
```

```
Molecule 70: k4 - Transition State
#n M06L/6-311++G** SCRF=(Solvent=Water) Opt=(TS,CalcFC,noeigentest)
E(RM06L) =  -837.926876810
Charge, multiplicity: 0, 1
Geometry:
C  1.284382    1.785329    0.00168
C  1.194352    0.417042   -0.00726
N -0.021943   -0.170345   -0.04215
C -1.198622    0.484676   -0.05679
C -1.125351    1.882881   -0.04894
C  0.096411    2.527372   -0.02591
H  2.253918    2.262964    0.02576
C  2.386000   -0.499847    0.00736
H -2.049431    2.448843   -0.06674
H  0.148472    3.610399   -0.02836
B -2.519204   -0.500849    0.00837
F  2.423655   -1.262636   -1.10102
O -2.023495   -1.875531   -0.21895
O -3.035292   -0.494274    1.38084
H -2.167551   -2.109880   -1.13763
H -3.361971    0.380346    1.60377
O -3.449444   -0.013982   -0.99991
H -4.301057   -0.440640   -0.87668
H -0.147625   -1.192404   -0.07551
F  2.337915   -1.340481    1.05684
F  3.529283    0.183408    0.07662
```

```
Molecule 70: k4 - Reactant
#n M06L/6-311++G** SCRF=(Solvent=Water) Opt
E(RM06L) =  -761.486079853
Charge, multiplicity: 0, 1
Geometry:
O  3.923620   -0.092381    0.00001
B  2.679281   -0.641842    0.00000
O  2.498478   -1.990796   -0.00000
C  1.421925    0.298751   -0.00000
C  1.481756    1.696135   -0.00000
C  0.307401    2.434451   -0.00000
C -0.908871    1.760973    0.00000
C -0.876214    0.372154   -0.00000
C -2.144762   -0.441646    0.00000
F -2.223505   -1.244931   -1.08068
F -3.246568    0.328479   -0.00001
F -2.223513   -1.244897    1.08070
N  0.240359   -0.352426   -0.00000
H  4.622230   -0.754143    0.00001
H  1.549182   -2.179569   -0.00002
H  2.447813    2.189208   -0.00000
H  0.330765    3.518534   -0.00000
H -1.850823    2.294812    0.00000
```

6.1.8 k_5

```
Molecule 0: k5 - Misc. small molecule
#n M06L/6-311++G** SCRF=(Solvent=Water) Opt
E(RM06L) =  -76.4487444811
Charge, multiplicity: 0, 1
Geometry:
O  0.000000    0.000000    0.11889
H -0.000000   -0.755103   -0.47558
```

```
H  0.000000    0.755103   -0.47558
```

```
Molecule 64: k5 - Transition State
#n M06L/6-311++G** SCRF=(Solvent=Water) Opt=(TS,CalcFC,noeigentest)
E(RM06L) =  -501.206134814
Charge, multiplicity: 1, 1
Geometry:
C  2.234041    1.164850   -0.02052
C  0.876212    1.196234    0.21135
C  0.131080    0.011815    0.31392
C  0.834467   -1.192697    0.16866
C  2.192271   -1.199940   -0.06637
N  2.848452   -0.026907   -0.15218
B -1.848623    0.023552   -0.17808
O -2.109541   -1.238781   -0.65162
O -2.043331    1.228473   -0.80200
H -2.114292   -1.317696   -1.60831
H -1.940381    1.201819   -1.75632
O -2.198681    0.137311    1.31152
H -2.585598   -0.684249    1.64671
H  0.394781    2.163863    0.30291
H  2.863242    2.040012   -0.10764
H  3.847651   -0.041495   -0.32679
H  2.790003   -2.092618   -0.19061
H -1.065170    0.048112    1.26581
H  0.317712   -2.144754    0.22463
```

```
Molecule 64: k5 - Intermediate
#n M06L/6-311++G** SCRF=(Solvent=Water) Opt
E(RM06L) =  -424.823206983
Charge, multiplicity: 1, 1
Geometry:
C  0.210618   -0.000002   -0.00001
C -0.511585    1.199069    0.01794
```

```
C -0.511593   -1.199072   -0.01796
C -1.889038   -1.182309   -0.01941
N -2.531410    0.000004    0.00001
C -1.889029    1.182312    0.01941
B  1.789192   -0.000002   -0.00001
O  2.386484    1.218050   -0.03642
O  2.386484   -1.218052    0.03643
H  3.348142    1.213203   -0.04420
H  3.348140   -1.213200    0.04423
H  0.001125   -2.152536   -0.03229
H -2.509070   -2.067255   -0.03494
H -2.509054    2.067262    0.03495
H  0.001139    2.152531    0.03227
H -3.546492    0.000008    0.00002
```

```
Molecule 65: k5 - Transition State
#n M06L/6-311++G** SCRF=(Solvent=Water) Opt=(TS,CalcFC,noeigentest)
E(RM06L) =  -632.803970241
Charge, multiplicity: 1, 1
Geometry:
C  1.005272    2.362298    0.02117
C -0.247841    1.815482    0.20269
C -0.464714    0.427102    0.26909
C  0.675309   -0.379595    0.14006
C  1.948319    0.225662   -0.04583
N  2.089834    1.559154   -0.10035
B -2.265759   -0.249796   -0.18264
O -2.087743   -1.457421   -0.82733
O -2.981460    0.839644   -0.63174
H -2.091053   -1.403225   -1.78554
H -2.918194    0.988457   -1.57779
O -2.575249   -0.473963    1.31108
H -2.559545   -1.416502    1.52877
```

```
H -1.086811    2.497409    0.28355
H  1.203061    3.423423   -0.03229
H  3.004697    1.977921   -0.22866
H -1.485134   -0.072674    1.21797
C  0.922397   -1.786478    0.15596
C  2.264515   -1.962401   -0.01102
N  2.891755   -0.730752   -0.13489
H  3.883007   -0.594048   -0.26589
H  0.187420   -2.566554    0.26949
H  2.856292   -2.862540   -0.05848
```

```
Molecule 65: k5 - Intermediate
#n M06L/6-311++G** SCRF=(Solvent=Water) Opt
E(RM06L) =  -556.421221389
Charge, multiplicity: 1, 1
Geometry:
C -0.788397    0.324693    0.00009
C -0.668451    1.724455    0.00014
C  0.404251   -0.411776    0.00000
C  1.641829    0.289392    0.00002
N  1.697017    1.629513    0.00010
C  0.556612    2.356875    0.00014
B -2.216253   -0.339522   -0.00001
O -3.272168    0.518527   -0.00061
O -2.274424   -1.698080    0.00062
H -4.141834    0.108478   -0.00047
H -3.159782   -2.073714    0.00044
H  0.683911    3.429374    0.00014
H -1.558542    2.340352    0.00017
H  2.592347    2.106196    0.00011
C  0.752359   -1.797352   -0.00022
C  2.114358   -1.869431   -0.00019
N  2.656773   -0.593192    0.00005
```

```
H   3.642837    -0.378014    -0.00091
H   0.070671    -2.630503    -0.00021
H   2.772508    -2.723515    -0.00026
```

6.2 Optimised Geometries and Energies for Novel Molecules

6.2.1 k_1

```
Molecule 0: k1 - Misc. small molecule
#n M06L/6-311++G** SCRF=(Solvent=Water) Opt
E(RM06L) =  -76.4487444811
Charge, multiplicity: 0, 1
Geometry:
O   0.000000     0.000000     0.11889
H  -0.000000    -0.755103    -0.47558
H   0.000000     0.755103    -0.47558
```

```
Molecule 0: k1 - Misc. small molecule
#n M06L/6-311++G** SCRF=(Solvent=Water) Opt
E(RM06L) =  -76.8403699674
Charge, multiplicity: 1, 1
Geometry:
O  -0.000024     0.000000    -0.08797
H  -0.898204     0.206601     0.23464
H   0.628198     0.674286     0.23456
H   0.270199    -0.880887     0.23458
```

```
Molecule 134: k1 - Intermediate
#n M06L/6-311++G** SCRF=(Solvent=Water) Opt
E(RM06L) =  -486.155509585
Charge, multiplicity: 1, 1
Geometry:
C  -1.724918     1.262733     0.44597
C  -0.710504     0.542836    -0.24012
```

```
C -0.987613   -0.766123   -0.71708
C -2.225153   -1.326540   -0.49971
C -3.209492   -0.598309    0.18127
C -2.963157    0.693378    0.65232
H -3.739021    1.238772    1.17520
H -1.505474    2.262468    0.80599
H -2.443854   -2.325320   -0.85708
H -4.184328   -1.046785    0.34194
C  0.539654    1.152673   -0.40258
H -0.223733   -1.321830   -1.24799
C  1.722803    0.588464   -0.98131
B  2.546831   -0.214945    0.16533
O  3.864869    0.062866    0.23740
O  1.822021   -1.097667    0.88838
H  2.314962   -1.628062    1.52202
H  4.359348   -0.449507    0.88533
H  0.631274    2.156542    0.01407
H  2.392889    1.352903   -1.37222
H  1.518936   -0.180711   -1.73269
```

```
Molecule 134: k1 - Reactant
#n M06L/6-311++G** SCRF=(Solvent=Water) Opt
E(RM06L) =  -485.744953867
Charge, multiplicity: 0, 1
Geometry:
C -1.798109    1.258053   -0.00000
C -0.903793    0.177046   -0.00000
C -1.434971   -1.123084   -0.00000
C -2.806407   -1.327887    0.00000
C -3.681766   -0.241457    0.00000
C -3.172473    1.053502    0.00000
H -3.846075    1.904105    0.00000
H -1.399941    2.269129   -0.00000
```

```
H -3.199063   -2.339536    0.00000
H -4.754102   -0.406708    0.00000
C  0.530790    0.448385   -0.00000
H -0.767814   -1.979095   -0.00000
C  1.545196   -0.437196   -0.00000
H  0.766069    1.516224   -0.00000
B  3.046445   -0.051963   -0.00001
H  1.288357   -1.498022   -0.00000
O  3.516087    1.236511    0.00000
O  4.044705   -0.993171    0.00000
H  3.706317   -1.891013    0.00001
H  2.816885    1.893841    0.00000
```

```
Molecule 141: k1 - Intermediate
#n M06L/6-311++G** SCRF=(Solvent=Water) Opt
E(RM06L) =  -406.511222546
Charge, multiplicity: 1, 1
Geometry:
C -1.055323    1.077412    0.47181
C -0.102316   -0.028230    0.68864
C -0.776787   -1.140088    0.08876
O -1.873490   -0.783260   -0.47751
C -2.071158    0.596407   -0.24277
B  1.404926    0.080622    0.01489
O  2.071668   -1.085502    0.02128
O  1.912082    1.231070   -0.44820
H  2.967370   -1.025019   -0.32890
H  1.349301    2.009409   -0.42087
H -2.977207    0.989702   -0.66927
H -0.943107    2.093792    0.81489
H -0.484408   -2.179079    0.00315
H  0.214853   -0.223373    1.72329
```

```
Molecule 141: k1 - Reactant
```

```
#n M06L/6-311++G** SCRF=(Solvent=Water) Opt
E(RM06L) =  -406.120872634
Charge, multiplicity: 0, 1
Geometry:
C -0.980522    1.141575    0.00023
C -0.069891    0.026392   -0.00004
C -0.880012   -1.074497    0.00007
O -2.189868   -0.731421   -0.00014
C -2.235850    0.632923    0.00000
B  1.475906    0.009981    0.00004
O  2.111258   -1.202590   -0.00002
O  2.256769    1.136193   -0.00014
H  3.068551   -1.106694    0.00074
H  1.759115    1.957101   -0.00025
H -0.678774   -2.134438    0.00005
H -3.223094    1.064841   -0.00012
H -0.732948    2.193471    0.00030
```

6.2.2 k_2

```
Molecule 0: k2 - Misc. small molecule
#n M06L/6-311++G** SCRF=(Solvent=Water) Opt
E(RM06L) =  -76.4487444811
Charge, multiplicity: 0, 1
Geometry:
O  0.000000    0.000000    0.11889
H -0.000000   -0.755103   -0.47558
H  0.000000    0.755103   -0.47558
```

```
Molecule 106: k2 - Transition State
#n M06L/6-311++G** SCRF=(Solvent=Water) Opt=(TS,CalcFC,noeigentest)
E(RM06L) =  -616.103231916
Charge, multiplicity: -1, 1
```

```
Geometry:
C  0.337684  -0.147295  0.35502
B -1.774828  -0.272378 -0.73044
O -2.309964   0.984936 -0.42685
O -2.219114  -1.452793 -0.12954
H -1.415267   0.256318  3.04822
H -0.959625   0.062692  1.53184
O -1.771750   0.132722  2.16531
H -2.383066  -1.288031  0.80647
H -2.426018   1.071448  0.52750
O -1.326560  -0.447666 -2.04002
H -1.147494   0.407483 -2.43635
C  0.976810  -1.393257  0.39815
C  1.208909   0.925640  0.05533
N  2.534087   0.821608 -0.19700
C  2.343087  -1.544335  0.15519
C  3.074363  -0.403546 -0.14414
H  0.386933  -2.287505  0.61835
H  2.834721  -2.513840  0.19230
H  4.143598  -0.467272 -0.34849
C  0.660718   2.324543 -0.03105
H -0.069094   2.514289  0.76016
H  0.124499   2.475504 -0.97522
H  1.456023   3.071455  0.02435
```

```
Molecule 106: k2 - Intermediate
#n M06L/6-311++G** SCRF=(Solvent=Water) Opt
E(RM06L) =  -539.679561742
Charge, multiplicity: -1, 1
Geometry:
C  0.889169   0.912394 -0.00827
C  0.076728  -0.242986  0.00533
N  2.237404   0.885624 -0.01093
```

```
C  0.764593  -1.458688   0.02153
C  2.156023  -1.508846   0.01874
C  0.287390   2.287171   0.00341
H  0.259325   2.685328   1.02415
H -0.735878   2.270651  -0.36834
H  0.890274   2.977956  -0.59103
B -1.555953  -0.204149   0.00364
O -2.166450  -1.551562   0.18393
H -2.101818  -2.035757  -0.64148
O -2.033347   0.597275   1.15578
H -2.875998   0.224779   1.42557
O -1.965937   0.367350  -1.31262
H -2.925325   0.420679  -1.32739
H  0.190855  -2.383549   0.04127
C  2.845053  -0.305858   0.00183
H  2.693989  -2.452002   0.02741
H  3.934652  -0.290341  -0.00410
```

```
Molecule 134: k2 - Transition State
#n M06L/6-311++G** SCRF=(Solvent=Water) Opt=(TS,CalcFC,noeigentest)
E(RM06L) =  -638.141094986
Charge, multiplicity: -1, 1
Geometry:
C  0.181949   0.561287  -0.09831
C  1.621852   0.253418  -0.06801
C  2.198576  -0.801341  -0.79777
C  3.561929  -1.056337  -0.74537
C  4.403063  -0.265954   0.03982
C  3.854153   0.783395   0.77258
C  2.487340   1.034626   0.71728
H  1.556365  -1.425828  -1.41357
H  3.977231  -1.879325  -1.32051
H  5.468824  -0.467289   0.07867
```

```
H   4.493843    1.408073    1.38970
H   2.065761    1.856348    1.29298
C  -0.826416   -0.015215   -0.79331
B  -2.838282   -0.647769    0.45599
O  -3.829798   -0.609878   -0.51127
O  -2.779808    0.390000    1.38724
H  -2.951616    2.226357   -0.23812
H  -2.135470    1.219922   -1.06582
O  -2.850903    1.944342   -1.15299
H  -1.993880    0.304082    1.93248
H  -3.884012    0.297361   -0.85236
H  -0.063531    1.404104    0.56808
H  -0.469334   -0.851786   -1.42748
O  -2.331434   -1.886198    0.83804
H  -2.431903   -2.522563    0.12641
```

```
Molecule 134: k2 - Intermediate
#n M06L/6-311++G** SCRF=(Solvent=Water) Opt
E(RM06L) =  -561.720760941
Charge, multiplicity: -1, 1
Geometry:
C  -2.179599    1.256007   -0.01862
C  -1.270331    0.185752   -0.00707
C  -1.803992   -1.114697    0.00932
C  -3.175264   -1.328898    0.01612
C  -4.061731   -0.251297    0.00715
C  -3.554072    1.044618   -0.01050
C   0.168752    0.465795   -0.01186
C   1.192833   -0.403553    0.04242
B   2.767799   -0.033674   -0.00374
O   3.389659   -0.453626   -1.29157
H   3.309047   -1.406009   -1.36557
O   3.385974   -0.753124    1.14945
```

```
H   4.318846   -0.522893    1.16607
O   3.038172    1.419305    0.04059
H   2.629126    1.779584    0.82901
H  -1.789626    2.271045   -0.03270
H   0.410245    1.531719   -0.06553
H   0.927843   -1.467472    0.10178
H  -1.130940   -1.967109    0.01455
H  -3.559547   -2.344635    0.02747
H  -5.133281   -0.422553    0.01248
H  -4.230722    1.893899   -0.01837
```

```
Molecule 141: k2 - Transition State
#n M06L/6-311++G** SCRF=(Solvent=Water) Opt=(TS,CalcFC,noeigentest)
E(RM06L) =  -558.517875740
Charge, multiplicity: -1, 1
Geometry:
C   1.461748   -0.097193    1.07262
C   1.558289    0.461527   -1.03183
O   2.853900    0.087010   -0.65936
C   2.747183   -0.252548    0.65226
H   3.666282   -0.572325    1.12316
C   0.638264    0.371700   -0.02990
B  -1.534686   -0.727183   -0.12073
O  -2.169578   -0.067231   -1.15916
O  -1.925222   -0.388074    1.18521
H  -1.927423    1.958441    0.98270
H  -0.704993    1.649418    0.12495
O  -1.456696    2.319749    0.22428
H  -1.377831   -0.802349    1.85526
H  -2.262431    0.863737   -0.90829
H   1.130391   -0.303724    2.08658
H   1.506060    0.749641   -2.07494
O  -1.063248   -1.995152   -0.45001
```

```
H -0.702770   -2.458251    0.30777
```

```
Molecule 141: k2 - Intermediate
#n M06L/6-311++G** SCRF=(Solvent=Water) Opt
E(RM06L) =  -482.096285261
Charge, multiplicity: -1, 1
Geometry:
B  1.292043    0.020970    0.02286
O  1.721835   -0.941238    1.08012
H  2.679221   -1.013484    1.04498
O  1.779019    1.397698    0.24901
H  2.677203    1.448732   -0.08527
O  1.866626   -0.401774   -1.29399
H  1.697257   -1.338618   -1.41442
C -1.175493    1.083992    0.02820
C -0.323350    0.022721    0.03318
C -1.201748   -1.118740   -0.00451
C -2.481923   -0.664349   -0.03026
O -2.496968    0.695691   -0.00977
H -1.028250    2.153227    0.04759
H -0.904495   -2.159399   -0.00976
H -3.450168   -1.140068   -0.06005
```

```
Molecule 143: k2 - Transition State
#n M06L/6-311++G** SCRF=(Solvent=Water) Opt=(TS,CalcFC,noeigentest)
E(RM06L) =  -883.347891480
Charge, multiplicity: -1, 1
Geometry:
C  1.324221   -0.050323    0.30541
B  0.680281    2.113030   -0.68839
O  1.496264    2.893134    0.12104
O -0.696674    2.201108   -0.49090
H -0.818478    1.976421    1.71631
H  0.304916    0.979271    1.52722
```

```
O -0.105599    1.574098    2.22548
H -1.178007    1.502050   -0.94668
H  1.148727    2.862807    1.02272
O  1.128502    1.766593   -1.95897
H  2.085493    1.687872   -1.96551
C  1.026873   -1.237220   -0.39931
C  2.613316   -0.059881    0.85807
C  3.488514   -1.138631    0.71041
N  1.832006   -2.305390   -0.57044
C  3.050056   -2.237508   -0.01389
H  4.486536   -1.135192    1.14280
H  3.694642   -3.104474   -0.15756
H  2.960736    0.809372    1.42453
C -0.321115   -1.347687   -1.07920
C -1.455006   -1.127707   -0.11885
H -0.444762   -2.346096   -1.51070
H -0.391255   -0.623975   -1.89754
O -2.315249   -0.187544   -0.57238
C -3.443137    0.130823    0.28401
C -4.583984   -0.828163    0.06579
H -3.708488    1.152125    0.00557
H -3.098096    0.127074    1.32053
H -4.902909   -0.829006   -0.97846
H -5.437897   -0.532482    0.67882
H -4.302835   -1.844876    0.34555
O -1.610018   -1.713705    0.92982
```

```
Molecule 143: k2 - Intermediate
#n M06L/6-311++G** SCRF=(Solvent=Water) Opt
E(RM06L) =  -806.924956152
Charge, multiplicity: -1, 1
Geometry:
B  0.789793    1.763956    0.00604
```

```
O  0.513821    2.216119    1.40499
H  1.338822    2.268475    1.89172
O -0.549508    1.650057   -0.62726
H -1.216127    1.637874    0.06311
O  1.609895    2.732890   -0.74440
H  1.111103    3.551963   -0.78873
C  2.911215    0.245450   -0.51804
C  3.599033   -0.964890   -0.50463
C  2.960469   -2.073156    0.03158
H  4.608008   -1.050517   -0.89684
C  1.612621    0.348668   -0.00941
C  1.076259   -0.850095    0.50316
N  1.719417   -2.030138    0.53073
H  3.460518   -3.040041    0.06837
C -0.319943   -0.870796    1.06488
C -1.377459   -1.032567    0.00486
H -0.522314    0.037589    1.63587
H -0.425964   -1.724176    1.74413
O -2.532998   -0.457449    0.38908
C -3.620629   -0.533540   -0.56069
C -4.782982    0.223313    0.01726
H -3.856706   -1.586596   -0.74037
H -3.276718   -0.105985   -1.50770
H -5.107053   -0.210373    0.96543
H -5.626625    0.191474   -0.67453
H -4.525369    1.270754    0.18807
O -1.251217   -1.633826   -1.03955
H  3.382096    1.134096   -0.93071
```

```
Molecule 144: k2 - Transition State
#n M06L/6-311++G** SCRF=(Solvent=Water) Opt=(TS,CalcFC,noeigentest)
E(RM06L) =  -671.485866648
Charge, multiplicity: -1, 1
```

```
Geometry:
C -0.040463   -0.205008    0.44048
B -2.115118   -0.148179   -0.84349
O -2.736411    0.930792   -0.22137
O -2.582451   -1.431570   -0.56512
H -2.696178   -1.179992    1.92823
H -1.454198   -0.335425    1.57151
O -2.254801   -0.371908    2.20804
H -2.067313   -2.088873   -1.03878
H -2.908575    0.685593    0.70350
O -1.517367    0.061032   -2.08268
H -1.232907    0.973330   -2.17534
C  0.767303   -1.334164    0.33432
C  0.686231    1.000469    0.29584
C  2.070310    0.991876    0.06723
N  2.105491   -1.403957    0.11627
C  2.739629   -0.226757   -0.01536
C  4.218645   -0.278079   -0.26231
H  4.647159    0.720834   -0.36711
H  4.447272   -0.842228   -1.17166
H  4.737680   -0.784451    0.55732
N  0.009127    2.212676    0.42228
H -0.984489    2.129477    0.23190
H  0.433651    3.008190   -0.03551
H  0.302164   -2.324718    0.42267
H  2.627316    1.921329   -0.05129
```

```
Molecule 144: k2 - Intermediate
#n M06L/6-311++G** SCRF=(Solvent=Water) Opt
E(RM06L) =  -595.064433884
Charge, multiplicity: -1, 1
Geometry:
B  1.961007   -0.285613    0.00242
```

```
O   2.493641    0.752947   -0.92239
H   3.450473    0.754460   -0.83567
O   2.458626   -1.642981   -0.33816
H   2.060098   -1.930858   -1.16224
O   2.501701    0.024031    1.35117
H   2.299652   -0.709513    1.93564
C  -0.377009    1.028003   -0.00788
C  -1.780119    1.009917    0.01578
C  -2.461282   -0.201413    0.00494
H  -2.337064    1.944276    0.04207
C   0.330534   -0.199469   -0.04550
C  -0.475617   -1.331962   -0.05809
N  -1.822904   -1.384076   -0.03326
C  -3.959603   -0.246257    0.02994
N   0.310817    2.222350    0.05814
H   0.014225   -2.307484   -0.08970
H  -4.396215    0.752983    0.07487
H  -4.318428   -0.814457    0.89308
H  -4.350833   -0.748993   -0.85949
H  -0.195615    3.042417   -0.24337
H   1.260105    2.152431   -0.30140
```

```
Molecule 147: k2 - Transition State
#n M06L/6-311++G** SCRF=(Solvent=Water) Opt=(TS,CalcFC,noeigentest)
E(RM06L) =  -574.597532301
Charge, multiplicity: -1, 1
Geometry:
C   0.603399    0.362282   -0.20326
B  -1.362649   -0.783389    0.09383
O  -2.215374   -0.473720   -0.96003
O  -1.575040   -0.170286    1.33714
H  -1.956497    1.999040    0.58284
H  -0.848197    1.696869   -0.38661
```

```
O -1.647581    2.273473   -0.28867
H -0.720121   -0.094214    1.78454
H -2.412592    0.472813   -0.93481
O -0.794400   -2.060140    0.08880
H -0.751242   -2.402688   -0.80715
N  1.211912    0.418521    0.97292
C  2.555260    0.144033    0.76914
C  2.758210   -0.080841   -0.54817
O  1.559532    0.050357   -1.18304
H  3.279476    0.124864    1.57184
H  3.620724   -0.319703   -1.15017
```

```
Molecule 147: k2 - Intermediate
#n M06L/6-311++G** SCRF=(Solvent=Water) Opt
E(RM06L) =  -498.164251134
Charge, multiplicity: -1, 1
Geometry:
B  1.262067   -0.033459    0.02850
O  1.594663    0.917492    1.11439
H  2.551353    0.986620    1.16510
O  1.737967    0.464324   -1.28991
H  1.516245    1.394329   -1.37082
O  1.857084   -1.361577    0.20910
H  2.594636   -1.431848   -0.39973
C -0.364396   -0.155215    0.05026
N -1.165704   -1.189532    0.05592
C -2.453976   -0.678063   -0.00289
C -2.392900    0.670209   -0.04605
O -1.077357    1.025957   -0.01251
H -3.327576   -1.313338   -0.00952
H -3.116288    1.467101   -0.09545
```

6.2.3 k_{2Ar}

```
Molecule 0: k2Ar - Misc. small molecule
#n M06L/6-311++G** SCRF=(Solvent=Water) Opt
E(RM06L) =  -252.544612170
Charge, multiplicity: 0, 1
Geometry:
B -0.003606    0.020891    0.00008
O  0.050934   -1.349120   -0.00005
O -1.230075    0.626202    0.00003
H  0.935459   -1.721156    0.00008
H -1.942286   -0.017511    0.00002
O  1.067085    0.872581   -0.00014
H  1.921297    0.436903    0.00084
```

```
Molecule 101: k2Ar - Intermediate, BA detached
#n M06L/6-311++G** SCRF=(Solvent=Water) Opt
E(RM06L) =  -644.054456479
Charge, multiplicity: -1, 1
Geometry:
C  3.284355    0.000116    0.32616
C  2.530000   -1.177289    0.11029
C  1.182619   -1.207620   -0.27128
C  0.530060   -0.000016   -0.45022
O -0.824132   -0.000135   -0.90646
C -1.765215   -0.000012    0.03307
F -1.717846    1.078782    0.84569
F -1.717122   -1.078056    0.84658
F -2.958831   -0.000716   -0.56892
C  1.182319    1.207587   -0.27141
C  2.529691    1.177393    0.11014
H  3.003438   -2.155111    0.24042
H  0.643925   -2.139053   -0.42992
H  0.643733    2.139129   -0.42991
```

```
H  3.003179    2.155066    0.24043
```

```
Molecule 101: k2Ar - Intermediate
#n M06L/6-311++G** SCRF=(Solvent=Water) Opt
E(RM06L) =  -896.648888390
Charge, multiplicity: -1, 1
Geometry:
O -3.600087   -0.162911    1.62281
B -3.364376    0.013978    0.17071
O -3.989355    1.315823   -0.15716
O -4.037318   -1.098282   -0.56327
C -1.757775    0.012302   -0.14781
C -1.025552    1.202910   -0.26385
C  0.349399    1.219828   -0.48741
C  1.012942    0.008534   -0.59468
O  2.400977    0.007884   -0.89894
C  3.239769    0.001981    0.13901
F  4.485969    0.003496   -0.33618
F  3.102391    1.076044    0.94429
F  3.100949   -1.080230    0.93320
C  0.347130   -1.201724   -0.49598
C -1.027956   -1.180983   -0.27125
H -3.090548   -0.915463    1.92988
H -4.430430    1.223470   -1.00200
H -3.572221   -1.281382   -1.38155
H -1.557779    2.147990   -0.18705
H  0.902817    2.148423   -0.58442
H  0.897158   -2.131487   -0.59924
H -1.552388   -2.132433   -0.19646
```

```
Molecule 102: k2Ar - Intermediate, BA detached
#n M06L/6-311++G** SCRF=(Solvent=Water) Opt
E(RM06L) =  -2529.76083233
Charge, multiplicity: -1, 1
```

```
Geometry:
C -1.211455    0.254271   -0.00000
C -1.157188   -1.140659    0.00000
C -0.000375   -1.877815   -0.00000
C  1.156737   -1.141131   -0.00000
C  1.211465    0.253793   -0.00000
C  0.000214    0.959748   -0.00000
Cl -2.735747   -2.011485    0.00000
Cl  0.001102    2.692646    0.00000
Cl -2.718219    1.141049    0.00000
Cl  2.734902   -2.012767    0.00000
Cl  2.718175    1.140601    0.00000
```

```
Molecule 102: k2Ar - Intermediate
#n M06L/6-311++G** SCRF=(Solvent=Water) Opt
E(RM06L) =  -2782.31923890
Charge, multiplicity: -1, 1
Geometry:
C -1.041213   -1.226039   -0.00084
C  0.356732   -1.150181   -0.04527
C  1.074294    0.055412    0.00083
C  0.272266    1.200345   -0.04257
C -1.129229    1.178810    0.00734
C -1.791050   -0.048498    0.05743
B  2.764727    0.038129    0.13797
O  3.221544   -0.234292   -1.23104
H  4.173701   -0.361433   -1.20459
O  3.335480    1.268276    0.70893
H  3.227185    2.007722    0.11043
Cl  0.994602    2.788464   -0.25511
Cl -2.058398    2.640498    0.00622
Cl -3.514714   -0.111065    0.15683
Cl -1.865740   -2.749454   -0.02108
```

```
Cl  1.207660   -2.664216   -0.23629
O  3.165686   -1.000889    1.10268
H  3.565033   -0.532640    1.83865
```

```
Molecule 103: k2Ar - Intermediate, BA detached
#n M06L/6-311++G** SCRF=(Solvent=Water) Opt
E(RM06L) =  -13098.8047024
Charge, multiplicity: -1, 1
Geometry:
C  1.216987    0.165435   -0.00000
C  1.163067   -1.230608   -0.00000
C  0.000000   -1.952743   -0.00000
C -1.163067   -1.230609    0.00000
C -1.216987    0.165435   -0.00000
C -0.000000    0.871973   -0.00000
Br  2.859978   -2.233350   -0.00000
Br -0.000001    2.769888    0.00000
Br  2.878371    1.123645    0.00000
Br -2.859977   -2.233351    0.00000
Br -2.878371    1.123645   -0.00000
```

```
Molecule 103: k2Ar - Intermediate
#n M06L/6-311++G** SCRF=(Solvent=Water) Opt
E(RM06L) =  -13351.3553853
Charge, multiplicity: -1, 1
Geometry:
C  1.379373    0.246786   -0.00468
C  0.485776    1.336747   -0.01889
C -0.915440    1.194799   -0.00677
C -1.356921   -0.145225   -0.01628
C -0.504707   -1.258676   -0.00929
C  0.881679   -1.060200    0.00614
B -2.089392    2.437640    0.06545
O -2.895288    2.204928    1.28939
```

```
O -1.476104    3.757809    0.09224
H -2.215246    4.368549    0.03204
H -2.369402    2.480919    2.04326
O -2.967968    2.394718   -1.10986
H -3.598008    1.675866   -1.03096
Br  1.305784    3.070314   -0.06976
Br  2.061589   -2.544104    0.03790
Br  3.264979    0.499020    0.00390
Br -3.240298   -0.544088   -0.05109
Br -1.177028   -3.037101   -0.01371
```

```
Molecule 104: k2Ar - Intermediate, BA detached
#n M06L/6-311++G** SCRF=(Solvent=Water) Opt
E(RM06L) =  -323.986534044
Charge, multiplicity: -1, 1
Geometry:
C  1.506692   -1.373315    0.00000
C  0.105674   -1.236964    0.00000
C -0.570346    0.005927   -0.00000
C  0.158565    1.204868    0.00000
C  1.543489    1.124709   -0.00000
C  2.175037   -0.125512   -0.00000
C -1.989184    0.038908   -0.00000
N -3.155115    0.027633    0.00000
H -0.536360   -2.121849   -0.00000
H -0.355148    2.160730    0.00000
H  2.128545    2.042418    0.00000
H  3.269208   -0.106463    0.00000
```

```
Molecule 104: k2Ar - Intermediate
#n M06L/6-311++G** SCRF=(Solvent=Water) Opt
E(RM06L) =  -576.580058631
Charge, multiplicity: -1, 1
Geometry:
```

```
O -2.709931   -0.089926    1.27107
B -2.028337   -0.380372   -0.02644
O -1.915926   -1.834159   -0.27524
O -2.863061    0.291752   -1.04866
C -0.510507    0.217123   -0.03938
C  0.627906   -0.591091   -0.03230
C  1.922283   -0.043480   -0.00610
C  2.105215    1.346113    0.01185
C  0.983595    2.164604    0.00361
C -0.291256    1.603787   -0.02113
C  3.054078   -0.903907    0.00014
N  3.976287   -1.613682    0.00577
H -2.258917   -0.555288    1.97817
H -2.805304   -2.192272   -0.32141
H -3.635884    0.645217   -0.60455
H  0.512848   -1.671020   -0.05196
H  3.105708    1.764036    0.03009
H  1.109630    3.243057    0.01492
H -1.156946    2.263681   -0.03084
```

```
Molecule 105: k2Ar - Intermediate, BA detached
#n M06L/6-311++G** SCRF=(Solvent=Water) Opt
E(RM06L) =  -365.692721259
Charge, multiplicity: -1, 1
Geometry:
C -2.238712   -1.218870    0.09537
N -1.538634   -0.000007   -0.25500
C -2.238705    1.218886    0.09544
C -0.138587   -0.000021   -0.10997
C  0.600416    1.194323   -0.05059
C  1.998314    1.165490    0.03136
C  2.796698    0.000025    0.07618
C  1.998339   -1.165487    0.03136
```

```
C  0.600433   -1.194345   -0.05054
H -1.933384   -2.046335   -0.54912
H -2.074416   -1.530723    1.14036
H -3.308795   -1.068751   -0.05117
H -3.308866    1.068496   -0.05021
H -2.073613    1.531162    1.14017
H -1.934035    2.046196   -0.54958
H  0.088315    2.152563   -0.07253
H  2.478848    2.149779    0.06884
H  2.478874   -2.149758    0.06895
H  0.088328   -2.152586   -0.07236
```

```
Molecule 105: k2Ar - Intermediate
#n M06L/6-311++G** SCRF=(Solvent=Water) Opt
E(RM06L) =  -618.284640680
Charge, multiplicity: -1, 1
Geometry:
C -3.754664   -0.203088   -1.18225
N -3.143622    0.355754    0.01673
C -3.730691   -0.231296    1.21429
C -1.704978    0.231947    0.00194
C -0.921892    1.383220    0.01750
C  0.470097    1.290681    0.00400
C  1.144908    0.061874   -0.02934
C  0.326505   -1.081060   -0.04556
C -1.063674   -1.009502   -0.02804
B  2.778108   -0.054001    0.00826
O  3.290499   -0.326576    1.37084
O  3.221701   -1.193907   -0.85703
O  3.428031    1.225447   -0.35815
H -3.318353    0.257885   -2.07112
H -4.827376    0.010407   -1.17649
H -3.632033   -1.297184   -1.26845
```

```
H -3.278363    0.210533    2.10486
H -3.603664   -1.326740    1.27301
H -4.803789   -0.020550    1.23381
H -1.419496    2.349980    0.03915
H  1.054971    2.209287    0.01983
H  0.798738   -2.062958   -0.07390
H -1.658188   -1.920941   -0.04075
H  3.026610   -1.216836    1.60987
H  2.635344   -1.276743   -1.61122
H  3.024906    1.567213   -1.15752
```

```
Molecule 107: k2Ar - Intermediate, BA detached
#n M06L/6-311++G** SCRF=(Solvent=Water) Opt
E(RM06L) =  -1150.92959520
Charge, multiplicity: -1, 1
Geometry:
C -2.468649   -0.885688   -0.00000
C -2.450684    0.527002    0.00000
C -1.294757    1.310364    0.00000
C -0.050822    0.686881   -0.00000
Cl  1.404206    1.662777   -0.00000
C -0.000923   -0.705646   -0.00000
Cl  1.563087   -1.533602    0.00000
C -1.179327   -1.455365   -0.00000
H -3.393386    1.081047    0.00000
H -1.336644    2.397200    0.00000
H -1.042981   -2.539518   -0.00000
```

```
Molecule 107: k2Ar - Intermediate
#n M06L/6-311++G** SCRF=(Solvent=Water) Opt
E(RM06L) =  -1403.52062488
Charge, multiplicity: -1, 1
Geometry:
O  3.457144   -0.765724   -1.03581
```

```
B   2.792179    0.059549    0.00038
O   3.374213   -0.345122    1.30075
O   3.110946    1.498831   -0.17310
C   1.172050   -0.208466   -0.02184
C   0.647097   -1.511209   -0.00239
C  -0.717369   -1.768383    0.01020
C  -1.619457   -0.707469    0.00630
Cl  -3.331053   -1.039820    0.02371
C  -1.132623    0.598481   -0.01047
Cl  -2.231457    1.958049   -0.01528
C   0.240127    0.833183   -0.02549
H   2.808976   -1.238618   -1.55788
H   3.109476    0.293638    1.96578
H   2.791030    1.797257   -1.02705
H   1.328144   -2.360333    0.01042
H  -1.099901   -2.783525    0.02562
H   0.586679    1.863235   -0.03454
```

```
Molecule 108: k2Ar - Intermediate, BA detached
#n M06L/6-311++G** SCRF=(Solvent=Water) Opt
E(RM06L) =  -669.224272816
Charge, multiplicity: -1, 1
Geometry:
C  -2.518609   -0.043514    1.04868
S  -1.836226    0.032156   -0.63428
C  -0.083754    0.012797   -0.27879
C   0.627241    1.205189   -0.12327
C   1.999670    1.166663    0.14474
C   2.763853   -0.017166    0.27653
C   1.982274   -1.184923    0.10639
C   0.609938   -1.194454   -0.16361
H  -3.605794   -0.038550    0.96145
H  -2.206665   -0.959884    1.55239
```

```
H -2.204862    0.822840    1.63331
H  0.098528    2.153960   -0.21335
H  2.489498    2.139923    0.25348
H  2.457592   -2.168380    0.18316
H  0.067641   -2.131958   -0.28592
```

```
Molecule 108: k2Ar - Intermediate
#n M06L/6-311++G** SCRF=(Solvent=Water) Opt
E(RM06L) =  -921.822508899
Charge, multiplicity: -1, 1
Geometry:
C  4.095708   -1.103484    0.01378
S  3.428025    0.570636    0.00026
C  1.673612    0.303370   -0.00120
C  0.852898    1.437798    0.01212
C -0.531155    1.301036    0.01372
C -1.173151    0.053730   -0.00036
C -0.319686   -1.059672   -0.01705
C  1.071322   -0.957303   -0.01592
B -2.800778   -0.102130    0.01882
O -3.264158   -0.745397    1.27590
O -3.512279    1.189900    0.01548
O -3.231621   -0.901427   -1.17787
H  5.179791   -0.994856    0.02444
H  3.789002   -1.650348    0.90681
H  3.807796   -1.659122   -0.88003
H  1.304698    2.427001    0.02279
H -1.143092    2.201318    0.02941
H -0.748928   -2.062979   -0.02988
H  1.674582   -1.858777   -0.02676
H -2.760533   -1.548584    1.41784
H -3.437243    1.568527   -0.86155
H -2.463415   -1.239163   -1.64013
```

```
Molecule 109: k2Ar - Intermediate, BA detached
#n M06L/6-311++G** SCRF=(Solvent=Water) Opt
E(RM06L) =  -306.955785540
Charge, multiplicity: -1, 1
Geometry:
C  1.134714    1.391784   -0.00000
C -0.264563    1.209216    0.00000
C -0.894917   -0.039916    0.00000
O -2.269520   -0.170395   -0.00000
C -0.142451   -1.211630    0.00000
C  1.246379   -1.091255    0.00000
C  1.851371    0.169225   -0.00000
H -0.938839    2.076483    0.00000
H -2.648942    0.715403    0.00000
H -0.637466   -2.178291    0.00000
H  1.852637   -1.997570    0.00000
H  2.945569    0.182592   -0.00000
```

```
Molecule 109: k2Ar - Intermediate
#n M06L/6-311++G** SCRF=(Solvent=Water) Opt
E(RM06L) =  -559.552434347
Charge, multiplicity: -1, 1
Geometry:
C -2.524291    0.784369    0.00878
C -1.588089    1.816127    0.02009
C -0.223904    1.536350    0.00819
C  0.257120    0.217013   -0.01840
C -0.699917   -0.803371   -0.02980
C -2.067388   -0.532302   -0.01526
B  1.846507   -0.129958   -0.00377
O  2.213474   -1.303901   -0.85191
O  2.592553    1.020360   -0.55390
O  2.190968   -0.424104    1.42331
```

```
H -1.937381    2.845586    0.03677
H  0.488595    2.357175    0.01192
H -0.390745   -1.847459   -0.05657
O -2.926722   -1.602337   -0.02885
H  1.954079   -2.107344   -0.39652
H  3.470605    0.712081   -0.78877
H  3.122427   -0.657704    1.46469
H -3.590933    0.993881    0.01546
H -3.832553   -1.275695   -0.01892
```

```
Molecule 110: k2Ar - Intermediate, BA detached
#n M06L/6-311++G** SCRF=(Solvent=Water) Opt
E(RM06L) =  -568.830055420
Charge, multiplicity: -1, 1
Geometry:
C  2.826798   -0.006287    0.00000
C  2.152838    1.215198   -0.00000
C  0.743554    1.362872   -0.00000
C  0.084301    0.116909   -0.00000
C  0.718587   -1.138005   -0.00000
C  2.103919   -1.200957    0.00000
H  2.779964    2.112001    0.00000
H  2.612057   -2.160682    0.00000
H  3.915797   -0.038178    0.00000
C -1.411395    0.050288   -0.00000
H  0.135447   -2.058250   -0.00000
F -2.045243    1.234485   -0.00004
F -1.908202   -0.631371    1.08215
F -1.908208   -0.631447   -1.08210
```

```
Molecule 110: k2Ar - Intermediate
#n M06L/6-311++G** SCRF=(Solvent=Water) Opt
E(RM06L) =  -821.413554741
Charge, multiplicity: -1, 1
```

```
Geometry:
C  0.318010   2.991153   0.00952
C -0.702929   2.045947   0.01031
C -0.488504   0.657417   0.00039
C  0.875010   0.272056  -0.00811
C  1.912524   1.212219  -0.00485
C  1.641829   2.573766   0.00216
H -1.732385   2.390951   0.02954
H  2.456474   3.290563   0.00425
H  0.078478   4.051059   0.01721
C  1.297528  -1.171438  -0.00226
H  2.942463   0.876859  -0.00688
B -1.842980  -0.294401   0.00630
O -3.092801   0.517940   0.09027
O -1.851660  -1.062730  -1.26169
H -2.693773  -1.522651  -1.31245
H -3.276267   0.900239  -0.77022
O -1.866771  -1.131544   1.22365
H -2.792122  -1.235515   1.45753
F  0.855691  -1.877349  -1.06497
F  0.892122  -1.845539   1.09760
F  2.652307  -1.316617  -0.02277
```

```
Molecule 111: k2Ar - Intermediate, BA detached
#n M06L/6-311++G** SCRF=(Solvent=Water) Opt
E(RM06L) =  -5378.56983107
Charge, multiplicity: -1, 1
Geometry:
C -1.205146   1.679058  -0.00000
C -1.131243   0.288043  -0.00000
C -0.000000  -0.494093  -0.00000
C  1.131242   0.288042   0.00000
C  1.205146   1.679058   0.00000
```

```
C   0.000000    2.378458    0.00000
Br  -2.923496   -0.611320    0.00000
H   0.000000    3.464120    0.00000
H  -2.152725    2.208428   -0.00000
H   2.152725    2.208428    0.00000
Br   2.923496   -0.611320   -0.00000
```

```
Molecule 111: k2Ar - Intermediate
#n M06L/6-311++G** SCRF=(Solvent=Water) Opt
E(RM06L) =  -5631.13310378
Charge, multiplicity: -1, 1
Geometry:
C   1.120414    2.235121    0.09101
C   1.120812    0.843265    0.04857
C  -0.030192    0.028296    0.06705
C  -1.207778    0.793093    0.05693
C  -1.267409    2.185944    0.09988
C  -0.089025    2.912667    0.13492
Br   2.899772    0.103895   -0.10574
H  -0.112226    3.996367    0.17628
H   2.056995    2.780418    0.08336
H  -2.226892    2.689688    0.09969
Br  -2.973331   -0.002432   -0.08924
B   0.111975   -1.637632    0.11539
O   0.986284   -1.923401    1.26624
O  -1.126948   -2.394920    0.36226
H  -1.767234   -2.240295   -0.33527
H   1.401021   -2.776528    1.12499
O   0.687930   -2.039390   -1.19480
H   0.403943   -2.941316   -1.36145
```

```
Molecule 112: k2Ar - Intermediate, BA detached
#n M06L/6-311++G** SCRF=(Solvent=Water) Opt
E(RM06L) =  -691.321229514
```

```
Charge, multiplicity: -1, 1
Geometry:
C -2.420678    0.000006   -0.00000
C -1.633459    1.174362   -0.00000
C -0.232131    1.206904    0.00000
C  0.450638   -0.000016    0.00000
Cl  2.224427    0.000000    0.00000
C -0.232136   -1.206910   -0.00000
C -1.633485   -1.174343   -0.00000
H -2.121218    2.154152    0.00000
H  0.317364    2.144805    0.00000
H  0.317318   -2.144836   -0.00000
H -2.121230   -2.154142   -0.00000
```

```
Molecule 112: k2Ar - Intermediate
#n M06L/6-311++G** SCRF=(Solvent=Water) Opt
E(RM06L) =  -943.916030600
Charge, multiplicity: -1, 1
Geometry:
O -2.916917   -0.104003    1.37515
B -2.413507    0.000697   -0.01763
O -2.936042   -1.165573   -0.78060
O -2.959264    1.271465   -0.55091
C -0.776145    0.004562   -0.05515
C -0.030807   -1.184059   -0.01226
C  1.362546   -1.203295    0.01603
C  2.042068    0.007679   -0.00074
Cl  3.802557    0.010388    0.02738
C  1.359330    1.216373   -0.04394
C -0.033886    1.193926   -0.06909
H -2.549926   -0.888214    1.78795
H -2.416928   -1.292200   -1.57683
H -3.346071    1.756632    0.17932
```

```
H -0.557661   -2.137088   -0.00436
H  1.913937   -2.137239    0.04740
H  1.909363    2.151643   -0.06013
H -0.569479    2.140155   -0.10882
```

```
Molecule 113: k2Ar - Intermediate, BA detached
#n M06L/6-311++G** SCRF=(Solvent=Water) Opt
E(RM06L) =  -568.825484328
Charge, multiplicity: -1, 1
Geometry:
C -2.981327   -0.000020    0.02007
C -2.195102    1.177758    0.00466
C -0.799922    1.203330   -0.02248
C -0.092694    0.000022   -0.04264
C  1.389763    0.000014   -0.00824
F  1.938253    1.084812   -0.60634
F  1.938239   -1.084492   -0.60690
F  1.893837   -0.000333    1.26270
C -0.799890   -1.203307   -0.02248
C -2.195068   -1.177778    0.00467
H -2.688551    2.154132    0.01200
H -0.255268    2.145637   -0.03519
H -0.255208   -2.145601   -0.03520
H -2.688491   -2.154165    0.01198
```

```
Molecule 113: k2Ar - Intermediate
#n M06L/6-311++G** SCRF=(Solvent=Water) Opt
E(RM06L) =  -821.420655352
Charge, multiplicity: -1, 1
Geometry:
O  3.625594    1.194998   -0.64841
B  3.038977   -0.010991    0.00195
O  3.432550   -0.126423    1.42301
O  3.583758   -1.178638   -0.72585
```

```
C  1.402574   0.016334  -0.00868
C  0.667321  -1.178891   0.00966
C -0.722338  -1.202121   0.03021
C -1.429159   0.000490   0.04051
C -2.916376  -0.005078   0.00379
F -3.407571   0.018371  -1.26759
F -3.453955  -1.102100   0.58130
F -3.462157   1.065838   0.62303
C -0.736277   1.210237   0.01786
C  0.654225   1.202550  -0.00521
H  3.335106   1.984931  -0.18795
H  4.392217  -0.169405   1.45394
H  3.502832  -1.023100  -1.66899
H  1.210184  -2.121280   0.00069
H -1.261415  -2.144566   0.04437
H -1.284959   2.147198   0.02340
H  1.169255   2.161570  -0.02479
```

```
Molecule 114: k2Ar - Intermediate, BA detached
#n M06L/6-311++G** SCRF=(Solvent=Water) Opt
E(RM06L) =  -271.031497112
Charge, multiplicity: -1, 1
Geometry:
C  2.412164  -0.051291   0.00000
C  0.902174  -0.080422   0.00000
C  0.225052   1.147456  -0.00000
C -1.169776   1.198050   0.00000
C -1.880392   0.002743   0.00000
C -1.180495  -1.212727  -0.00000
C  0.224886  -1.331196  -0.00000
H  2.822699  -0.568590   0.87452
H  2.822700  -0.568611  -0.87451
H  2.813864   0.967378  -0.00001
```

```
H  0.790414   2.081094  -0.00000
H -1.686285   2.154857  -0.00000
H -2.970235   0.018159   0.00000
H -1.794835  -2.119971   0.00000
```

```
Molecule 114: k2Ar - Intermediate
#n M06L/6-311++G** SCRF=(Solvent=Water) Opt
E(RM06L) =  -523.628382165
Charge, multiplicity: -1, 1
Geometry:
C -0.208677   2.299738  -0.00614
C -0.851224   0.940662  -0.00341
C -2.247982   0.859590   0.01652
C -2.906840  -0.367494   0.00038
C -2.155639  -1.537271  -0.03298
C -0.763117  -1.455938  -0.04800
C -0.068541  -0.238052  -0.03359
B  1.563967  -0.216310  -0.01886
O  2.134747  -1.583815  -0.21718
O  2.056853   0.641889  -1.11868
O  2.078063   0.318945   1.28822
H -0.883301   3.062152   0.39363
H  0.065903   2.604738  -1.02138
H  0.717229   2.297346   0.57089
H -2.830398   1.779173   0.04822
H -3.992546  -0.405740   0.01802
H -2.650550  -2.505294  -0.04374
H -0.176471  -2.371947  -0.07183
H  2.599495  -1.811842   0.58912
H  2.925726   0.307755  -1.35227
H  1.459893   0.101638   1.98833
```

```
Molecule 115: k2Ar - Intermediate, BA detached
#n M06L/6-311++G** SCRF=(Solvent=Water) Opt
```

```
E(RM06L) =  -385.375096627
Charge, multiplicity: -1, 1
Geometry:
C -1.245557   -1.547209    0.00000
C -2.409531   -0.776936   -0.00000
C -2.441291    0.640663   -0.00000
C -1.271535    1.364139   -0.00000
C -0.030860    0.681909    0.00000
C  1.201633    1.383802    0.00000
C  2.403041    0.714264   -0.00000
C  2.419312   -0.697309    0.00000
C  1.234078   -1.398269   -0.00000
C -0.037517   -0.760496    0.00000
H -3.385726   -1.273128    0.00000
H -3.396862    1.165085    0.00000
H -1.279299    2.452202   -0.00000
H  1.181674    2.472506   -0.00000
H  3.337715    1.267766    0.00000
H  3.369088   -1.224816   -0.00000
H  1.242773   -2.486963   -0.00000
```

```
Molecule 115: k2Ar - Intermediate
#n M06L/6-311++G** SCRF=(Solvent=Water) Opt
E(RM06L) =  -637.969331900
Charge, multiplicity: -1, 1
Geometry:
O  2.252227   -1.136779    1.26730
B  2.120172   -0.347957    0.00427
O  3.271843    0.578318   -0.10191
O  2.128956   -1.303366   -1.15152
C  0.734724    0.518047    0.00257
C  0.756845    1.902740    0.00624
C -0.414306    2.692672    0.00277
```

```
C -1.651812    2.098390   -0.00140
C -1.756201    0.685818   -0.00061
C -3.019282    0.042026   -0.00217
C -3.117745   -1.328438   -0.00129
C -1.946341   -2.114892    0.00080
C -0.707178   -1.517275    0.00158
C -0.557969   -0.104559    0.00059
H  2.336891   -2.057093    1.01088
H  3.979419    0.194406    0.41942
H  2.950129   -1.149201   -1.62241
H  1.723244    2.400238    0.01431
H -0.333450    3.776950    0.00400
H -2.561756    2.693363   -0.00427
H -3.915418    0.658152   -0.00428
H -4.092205   -1.807390   -0.00227
H -2.027331   -3.197985    0.00140
H  0.190997   -2.124228   -0.00342
```

```
Molecule 116: k2Ar - Intermediate, BA detached
#n M06L/6-311++G** SCRF=(Solvent=Water) Opt
E(RM06L) =  -346.255005431
Charge, multiplicity: -1, 1
Geometry:
C  2.729329   -0.302792   -0.00002
O  1.720571    0.686705    0.00026
C  0.409175    0.252627    0.00000
C -0.546935    1.271112   -0.00012
C -1.890350    0.914967   -0.00008
C -2.264584   -0.434236    0.00019
C -1.344571   -1.506295   -0.00001
C  0.010609   -1.089793   -0.00016
H  3.680831    0.228226    0.00005
H  2.674559   -0.940492   -0.89001
```

```
H  2.674537   -0.940974    0.88962
H -0.225687    2.308688   -0.00029
H -2.645256    1.702069   -0.00034
H -3.339167   -0.641374    0.00054
H  0.799582   -1.843327   -0.00047
```

```
Molecule 116: k2Ar - Intermediate
#n M06L/6-311++G** SCRF=(Solvent=Water) Opt
E(RM06L) =  -598.852496544
Charge, multiplicity: -1, 1
Geometry:
C  3.002568   -1.886117    0.01326
O  3.050525   -0.470764    0.01560
C  1.853505    0.197095   -0.00420
C  1.942584    1.591815   -0.00917
C  0.772986    2.340799   -0.03414
C -0.471358    1.710763   -0.05251
C -0.586363    0.313790   -0.04662
B -2.040861   -0.414510    0.00359
O -2.918380    0.206062   -1.02745
O -1.908507   -1.873259   -0.22002
O -2.655900   -0.252040    1.36003
C  0.605048   -0.427643   -0.02280
H  2.481141   -2.273909    0.89525
H  2.509223   -2.271474   -0.88580
H  4.036752   -2.226446    0.02906
H  2.920591    2.062292    0.00228
H  0.839383    3.426099   -0.04296
H -1.374566    2.317685   -0.08065
H -3.826784    0.080277   -0.74246
H -2.566993   -2.126690   -0.86738
H -2.601375    0.671930    1.61513
H  0.531207   -1.510229   -0.02847
```

```
Molecule 117: k2Ar - Intermediate, BA detached
#n M06L/6-311++G** SCRF=(Solvent=Water) Opt
E(RM06L) =  -385.373925304
Charge, multiplicity: -1, 1
Geometry:
C -2.543580   0.819906   0.00000
C -2.475537  -0.614494   0.00000
C -1.309020  -1.353676  -0.00000
C -0.049099  -0.708598  -0.00000
C  1.182608  -1.408005  -0.00000
C  2.382191  -0.733401   0.00000
C  2.397676   0.679467   0.00000
C  1.215617   1.383835   0.00000
C -0.039103   0.722496  -0.00000
C -1.282593   1.416309  -0.00000
H -3.408414  -1.187611   0.00000
H -1.331967  -2.444292  -0.00000
H  1.163891  -2.495827  -0.00000
H  3.318582  -1.283078   0.00000
H  3.347997   1.205708   0.00000
H  1.226330   2.472238   0.00000
H -1.191384   2.509827   0.00000
```

```
Molecule 117: k2Ar - Intermediate
#n M06L/6-311++G** SCRF=(Solvent=Water) Opt
E(RM06L) =  -637.970062670
Charge, multiplicity: -1, 1
Geometry:
O -3.536483   0.865056  -0.62374
B -2.715675  -0.201134   0.01861
O -3.137664  -0.247219   1.43601
O -3.005467  -1.495332  -0.67089
C -1.119377   0.134171  -0.00530
```

```
C -0.671006    1.484764   -0.01680
C  0.660519    1.825772   -0.01899
C  1.663636    0.825611   -0.00883
C  3.047055    1.126919   -0.01688
C  3.988942    0.124849   -0.00366
C  3.584276   -1.227597    0.01866
C  2.247436   -1.550972    0.02593
C  1.251004   -0.543542    0.01123
C -0.136322   -0.839133    0.01505
H -3.560461    0.695990   -1.56722
H -4.096703   -0.193819    1.45042
H -2.220532   -1.822045   -1.11211
H -1.424254    2.269600   -0.03411
H  0.968400    2.869372   -0.03170
H  3.353397    2.169836   -0.03376
H  5.046414    0.369620   -0.01008
H  4.334473   -2.012244    0.02956
H  1.934061   -2.591972    0.04224
H -0.416476   -1.893752    0.04027
```

```
Molecule 118: k2Ar - Intermediate, BA detached
#n M06L/6-311++G** SCRF=(Solvent=Water) Opt
E(RM06L) =  -345.070215297
Charge, multiplicity: -1, 1
Geometry:
O -2.823782    0.366464   -0.00063
C -1.922297   -0.462515    0.00074
C -0.497533   -0.190079    0.00035
C  0.021847    1.115717    0.00015
C  1.396709    1.310983   -0.00012
C  2.362342    0.265819   -0.00028
C  1.777209   -1.025808   -0.00008
C  0.406128   -1.263727    0.00018
```

```
H -2.171554   -1.550522   -0.00076
H -0.674663    1.953365    0.00026
H  1.738363    2.350167   -0.00023
H  2.424558   -1.907284   -0.00016
H  0.007125   -2.279769    0.00032
```

```
Molecule 118: k2Ar - Intermediate
#n M06L/6-311++G** SCRF=(Solvent=Water) Opt
E(RM06L) =  -597.663915109
Charge, multiplicity: -1, 1
Geometry:
O  4.357288   -0.633242   -0.01935
C  3.574647    0.303750   -0.00203
C  2.120831    0.202723   -0.00552
C  1.465684   -1.038837   -0.03092
C  0.083394   -1.086921   -0.03299
C -0.714893    0.076516   -0.00459
B -2.339523   -0.035477    0.00507
O -2.784768   -0.759816    1.23819
O -2.846219   -0.763987   -1.19780
O -2.912395    1.312951   -0.03052
C -0.034537    1.304190    0.01777
C  1.352489    1.373502    0.01679
H  3.956154    1.350885    0.01916
H  2.062872   -1.946430   -0.05109
H -0.404431   -2.061267   -0.06189
H -2.077003   -1.301281    1.59068
H -2.838854   -1.705075   -1.01289
H -3.841700    1.228012   -0.25332
H -0.618406    2.220303    0.03442
H  1.862035    2.335464    0.03456
```

```
Molecule 119: k2Ar - Intermediate, BA detached
#n M06L/6-311++G** SCRF=(Solvent=Water) Opt
```

```
E(RM06L) =  -346.260363915
Charge, multiplicity: -1, 1
Geometry:
C -2.721837   0.319891   0.00000
O -1.763820  -0.714187  -0.00000
C -0.412289  -0.350614  -0.00000
C -0.045002   1.000152  -0.00000
C  1.314551   1.329793  -0.00000
C  2.263432   0.317400   0.00000
C  1.831957  -1.020694   0.00000
C  0.488191  -1.429748  -0.00000
H -2.640764   0.958961   0.88922
H -2.640787   0.958965  -0.88921
H -3.701258  -0.159558   0.00001
H -0.778146   1.802029  -0.00000
H  1.613125   2.375010  -0.00000
H  3.324442   0.562742   0.00000
H  2.619929  -1.781732   0.00000
```

```
Molecule 119: k2Ar - Intermediate
#n M06L/6-311++G** SCRF=(Solvent=Water) Opt
E(RM06L) =  -598.854324382
Charge, multiplicity: -1, 1
Geometry:
C  1.520073   2.698875  -0.01668
O  0.499711   1.718559  -0.01511
C  0.878133   0.390930  -0.00388
C  2.219300   0.006679   0.01254
C  2.543465  -1.351278   0.02314
C  1.533412  -2.302111   0.01404
C  0.200091  -1.880636  -0.00345
C -0.182683  -0.537277  -0.01220
B -1.765939  -0.081319   0.00806
```

```
O -2.128569    0.418775    1.36558
O -2.659438   -1.227701   -0.24527
O -2.031079    0.959262   -1.02255
H  2.143506    2.632855    0.88195
H  1.017481    3.664829   -0.03208
H  2.162663    2.613681   -0.90027
H  3.014317    0.743256    0.01841
H  3.587452   -1.650341    0.03729
H  1.776532   -3.360865    0.02236
H -0.588589   -2.631259   -0.00440
H -2.299710    1.357736    1.28209
H -2.408809   -1.638625   -1.07504
H -1.290894    1.573070   -1.01270
```

```
Molecule 120: k2Ar - Intermediate, BA detached
#n M06L/6-311++G** SCRF=(Solvent=Water) Opt
E(RM06L) =  -323.985743136
Charge, multiplicity: -1, 1
Geometry:
N -3.151108   -0.000007    0.00004
C -1.985620    0.000015    0.00005
C -0.568994   -0.000004    0.00001
C  0.145620    1.209748   -0.00000
C  1.536811    1.180348   -0.00004
C  2.322949   -0.000003   -0.00005
C  1.536812   -1.180347   -0.00002
C  0.145615   -1.209748    0.00000
H -0.403152    2.149634    0.00000
H  2.032453    2.155261   -0.00005
H  2.032444   -2.155265   -0.00003
H -0.403148   -2.149640    0.00002
```

```
Molecule 120: k2Ar - Intermediate
#n M06L/6-311++G** SCRF=(Solvent=Water) Opt
```

```
E(RM06L) =  -576.577841071
Charge, multiplicity: -1, 1
Geometry:
N -4.776522    0.003667    0.02518
C -3.612636    0.005147    0.01749
C -2.193268    0.007157    0.00817
C -1.483484   -1.205045    0.03162
C -0.098154   -1.184151    0.01778
C  0.641647    0.012317   -0.01247
B  2.279146   -0.020287    0.00304
O  2.759797   -0.909648   -1.09027
O  2.761082   -0.588217    1.27802
O  2.863611    1.342610   -0.07905
C -0.100120    1.203413   -0.03285
C -1.488233    1.219109   -0.02545
H -2.029361   -2.143047    0.05827
H  0.436688   -2.131595    0.03146
H  2.165528   -0.864133   -1.84128
H  3.293615    0.083383    1.70727
H  2.705230    1.719394   -0.94626
H  0.434525    2.150293   -0.05176
H -2.036738    2.155826   -0.04455
```

```
Molecule 121: k2Ar - Intermediate, BA detached
#n M06L/6-311++G** SCRF=(Solvent=Water) Opt
E(RM06L) =  -271.032117732
Charge, multiplicity: -1, 1
Geometry:
C  2.377638    0.046428   -0.00003
C  0.875678    0.001046    0.00001
C  0.130708    1.185259    0.00002
C -1.259980    1.118315    0.00000
C -1.903866   -0.124918   -0.00004
```

```
C -1.227190   -1.366808   -0.00000
C  0.179837   -1.218913    0.00003
H  2.771763    0.571986    0.87661
H  2.771680    0.572103   -0.87664
H  2.805472   -0.959498   -0.00011
H  0.638399    2.148657    0.00004
H -1.840064    2.041888    0.00005
H -2.998913   -0.103365   -0.00007
H  0.814715   -2.114229    0.00008
```

```
Molecule 121: k2Ar - Intermediate
#n M06L/6-311++G** SCRF=(Solvent=Water) Opt
E(RM06L) =  -523.631410045
Charge, multiplicity: -1, 1
Geometry:
C  3.146781   -1.515461    0.00908
C  2.101263   -0.438570   -0.01654
C  2.461359    0.910701    0.01880
C  1.476699    1.897089    0.02053
C  0.130891    1.542602   -0.01378
C -0.280224    0.200627   -0.05709
B -1.854947   -0.194994   -0.03157
O -2.624185    0.796008   -0.81626
O -1.979904   -1.582103   -0.56011
O -2.431230   -0.131815    1.35429
C  0.739071   -0.761045   -0.05790
H  3.309223   -1.891198    1.02557
H  2.855211   -2.375443   -0.59925
H  4.111589   -1.154051   -0.35454
H  3.513268    1.186963    0.04106
H  1.766660    2.945107    0.04384
H -0.627377    2.323157   -0.01662
H -3.534404    0.765785   -0.51270
```

```
H -2.900826   -1.843482   -0.48567
H -2.049196   -0.841282    1.87535
H  0.458106   -1.812961   -0.10106
```

```
Molecule 122: k2Ar - Intermediate, BA detached
#n M06L/6-311++G** SCRF=(Solvent=Water) Opt
E(RM06L) =  -2805.13069174
Charge, multiplicity: -1, 1
Geometry:
C  3.028758    0.000015    0.00000
C  2.241329    1.173788   -0.00000
C  0.839615    1.206197   -0.00000
C  0.152436   -0.000030   -0.00000
Br -1.774524   -0.000000    0.00000
C  0.839629   -1.206212    0.00000
C  2.241377   -1.173752    0.00000
H  2.728379    2.154109    0.00000
H  0.296304    2.147885    0.00000
H  0.296394   -2.147943    0.00000
H  2.728412   -2.154084    0.00000
```

```
Molecule 122: k2Ar - Intermediate
#n M06L/6-311++G** SCRF=(Solvent=Water) Opt
E(RM06L) =  -3057.72534668
Charge, multiplicity: -1, 1
Geometry:
O -3.603806   -0.101659    1.37933
B -3.105305    0.001707   -0.01516
O -3.631160   -1.164603   -0.77549
O -3.651302    1.272580   -0.54764
C -1.467968    0.004062   -0.05729
C -0.723455   -1.185030   -0.01645
C  0.670247   -1.205242    0.00835
C  1.354487    0.004600   -0.01003
```

```
Br  3.269125    0.005823    0.01648
C  0.669087    1.213403   -0.05160
C -0.724509    1.192523   -0.07312
H -3.237319   -0.886719    1.79097
H -3.116547   -1.290827   -1.57470
H -4.035747    1.758588    0.18327
H -1.250923   -2.137848   -0.00730
H  1.215215   -2.143271    0.03845
H  1.214311    2.151725   -0.06932
H -1.259017    2.139571   -0.11132
```

```
Molecule 123: k2Ar - Intermediate, BA detached
#n M06L/6-311++G** SCRF=(Solvent=Water) Opt
E(RM06L) =  -388.984774354
Charge, multiplicity: -1, 1
Geometry:
C  1.918253   -0.728446   -1.24853
C  1.414729    0.009621   -0.00002
C  2.016648    1.414404    0.00034
C  1.918263   -0.728986    1.24816
C -0.114730    0.033046    0.00000
C -0.839093   -1.166623    0.00002
C -2.236601   -1.172488    0.00004
C -3.049625   -0.012794   -0.00002
C -2.273317    1.166861   -0.00003
C -0.870859    1.208102    0.00000
H  3.012883   -0.766992   -1.26200
H  1.549397   -1.757151   -1.28396
H  1.588878   -0.224570   -2.16259
H  1.718125    1.986000   -0.88348
H  3.109134    1.352509    0.00025
H  1.718209    1.985448    0.88456
H  1.549524   -1.757751    1.28313
```

```
H  1.588771   -0.225562    2.16244
H  3.012893   -0.767364    1.26166
H -0.298600   -2.114926    0.00000
H -2.708476   -2.161499    0.00008
H -2.774295    2.141621   -0.00006
H -0.368454    2.174054    0.00008
```

```
Molecule 123: k2Ar - Intermediate
#n M06L/6-311++G** SCRF=(Solvent=Water) Opt
E(RM06L) =  -641.583927048
Charge, multiplicity: -1, 1
Geometry:
C -3.452242   -0.774392    1.23207
C -2.958352   -0.000422    0.00176
C -3.449732   -0.713082   -1.26625
C -3.572635    1.398457    0.03680
C -1.430030    0.038191    0.00096
C -0.693586    1.224875   -0.00820
C  0.703310    1.208699   -0.01888
C  1.449920    0.025409   -0.01693
B  3.081141   -0.015454    0.00903
O  3.479152   -0.151393    1.43112
O  3.688938    1.196498   -0.62032
O  3.638585   -1.176957   -0.72651
C  0.692800   -1.157093   -0.00628
C -0.698078   -1.157531    0.00011
H -3.125058   -0.292174    2.15821
H -4.546147   -0.818542    1.24449
H -3.079625   -1.802005    1.24115
H -3.123131   -0.183891   -2.16665
H -3.072015   -1.737393   -1.32568
H -4.543547   -0.760484   -1.28247
H -3.288804    1.991645   -0.83738
```

```
H -3.273059    1.952748    0.93121
H -4.663959    1.325033    0.04428
H -1.206593    2.181981   -0.00969
H  1.219701    2.169178   -0.03373
H  4.439304   -0.186611    1.45699
H  3.344598    1.984674   -0.19516
H  3.519872   -1.031955   -1.66729
H  1.219197   -2.110425   -0.01131
H -1.228088   -2.108368    0.00255
```

```
Molecule 124: k2Ar - Intermediate, BA detached
#n M06L/6-311++G** SCRF=(Solvent=Water) Opt
E(RM06L) =  -459.641517258
Charge, multiplicity: -1, 1
Geometry:
C  3.337386   -0.536691   -0.00019
O  1.923091   -0.738848    0.00009
C  1.176069    0.391696    0.00048
O  1.687765    1.498851    0.00013
C -0.266597    0.118553    0.00019
C -0.801026   -1.178172    0.00009
C -2.183112   -1.359733   -0.00015
C -3.137365   -0.310959   -0.00033
C -2.532749    0.971967   -0.00023
C -1.159063    1.199279    0.00001
H  3.651299    0.015164    0.88794
H  3.650887    0.015527   -0.88825
H  3.781106   -1.529789   -0.00050
H -0.125607   -2.030675    0.00021
H -2.528233   -2.398177   -0.00022
H -3.166846    1.863713   -0.00034
H -0.750719    2.208574    0.00009
```

```
Molecule 124: k2Ar - Intermediate
```

```
#n M06L/6-311++G** SCRF=(Solvent=Water) Opt
E(RM06L) =  -712.236037264
Charge, multiplicity: -1, 1
Geometry:
C   4.883328    0.753833   -0.00903
O   3.454873    0.835208   -0.00657
C   2.807487   -0.349385   -0.00029
O   3.397335   -1.414310    0.00444
C   1.341992   -0.192352   -0.00178
C   0.715112    1.059080    0.00166
C  -0.672690    1.141812   -0.00563
C  -1.494301    0.005006   -0.01458
B  -3.122340    0.091959   -0.01983
O  -3.603318   -0.544947   -1.25891
O  -3.669923   -0.636265    1.17138
O  -3.631289    1.489832   -0.02561
C  -0.836525   -1.238676   -0.01239
C   0.546190   -1.345146   -0.00878
H   5.239717    1.780976   -0.01993
H   5.241957    0.239152    0.88388
H   5.237703    0.221650   -0.89331
H   1.320974    1.959138    0.00788
H  -1.145825    2.121393   -0.00890
H  -4.526142   -0.302352   -1.36691
H  -3.017692   -0.666358    1.87277
H  -3.666833    1.810014    0.87734
H  -1.432180   -2.149604   -0.01490
H   1.035032   -2.314981   -0.01142
```

```
Molecule 126: k2Ar - Intermediate, BA detached
#n M06L/6-311++G** SCRF=(Solvent=Water) Opt
E(RM06L) =  -436.286621727
Charge, multiplicity: -1, 1
```

```
Geometry:
C -2.510606   -0.011646   -0.00001
C -1.820932   -1.223527   -0.00009
C -0.411004   -1.343880   -0.00008
C  0.205131   -0.091428    0.00000
C -0.421604    1.164215    0.00010
C -1.805812    1.196275    0.00009
N  1.704399   -0.034693   -0.00001
O  2.356812   -1.075838    0.00021
O  2.251208    1.075821   -0.00023
H -3.599497    0.005154   -0.00003
H -2.432000   -2.130323   -0.00016
H  0.156139    2.081688    0.00019
H -2.330633    2.146415    0.00017
```

```
Molecule 126: k2Ar - Intermediate
#n M06L/6-311++G** SCRF=(Solvent=Water) Opt
E(RM06L) =  -688.872741095
Charge, multiplicity: -1, 1
Geometry:
C -1.901253   -2.150036   -0.07452
C -0.528259   -1.924033   -0.05295
C  0.040987   -0.641994   -0.03799
C -0.903497    0.391388   -0.03461
C -2.286310    0.213312   -0.01040
C -2.790649   -1.077089   -0.04828
B  1.676701   -0.456293    0.09462
O  1.857893    0.517200    1.18979
O  2.339438   -1.719833    0.46918
H  2.801477    0.630339    1.32850
H  2.560526   -2.197909   -0.33241
O  2.282102   -0.040790   -1.19274
H  1.800296    0.732281   -1.49929
```

```
N -0.464634    1.796232   -0.06612
O  0.406129    2.121915   -0.86945
O -1.028252    2.593804    0.67918
H -2.283687   -3.166420   -0.09583
H  0.150160   -2.772701   -0.02262
H -2.941889    1.076639    0.01271
H -3.862537   -1.242034   -0.05625
```

```
Molecule 127: k2Ar - Intermediate, BA detached
#n M06L/6-311++G** SCRF=(Solvent=Water) Opt
E(RM06L) =  -384.396022671
Charge, multiplicity: -1, 1
Geometry:
C -2.478365    1.047895    0.00009
C -1.639173   -0.205661   -0.00054
O -2.186825   -1.308174    0.00013
C -0.172491   -0.068276   -0.00025
C  0.470825    1.180115   -0.00014
C  1.862544    1.255631    0.00011
C  2.731752    0.136050    0.00029
C  2.025974   -1.096384    0.00019
C  0.640332   -1.213892   -0.00006
H -2.262857    1.665300    0.87635
H -2.263585    1.665657   -0.87609
H -3.535481    0.786886    0.00048
H -0.122217    2.093309   -0.00026
H  2.287701    2.263841    0.00017
H  2.589287   -2.034655    0.00030
H  0.153370   -2.187802   -0.00013
```

```
Molecule 127: k2Ar - Intermediate
#n M06L/6-311++G** SCRF=(Solvent=Water) Opt
E(RM06L) =  -636.990487076
Charge, multiplicity: -1, 1
```

```
Geometry:
C -4.027783  1.128854  0.00247
C -3.241314 -0.155481 -0.00133
O -3.821232 -1.237133  0.00302
C -1.763100 -0.077359 -0.01072
C -1.010231 -1.261564 -0.02592
C  0.374581 -1.216804 -0.03588
C  1.086048 -0.001908 -0.03194
B  2.712803  0.032393  0.01185
O  3.197268 -1.050355 -0.88030
O  3.277758  1.343911 -0.40507
O  3.200281 -0.183300  1.40284
C  0.314686  1.169770 -0.01649
C -1.075243  1.144044 -0.00633
H -3.782671  1.735741  0.87816
H -5.094037  0.909953  0.00744
H -3.790502  1.735717 -0.87539
H -1.541239 -2.209151 -0.03125
H  0.935656 -2.148636 -0.05202
H  4.157239 -1.041446 -0.85648
H  3.104869  1.466958 -1.34084
H  2.467917 -0.180929  2.01887
H  0.828556  2.128670 -0.01791
H -1.628272  2.078869  0.00327
```

```
Molecule 128: k2Ar - Intermediate, BA detached
#n M06L/6-311++G** SCRF=(Solvent=Water) Opt
E(RM06L) =  -790.586615510
Charge, multiplicity: -1, 1
Geometry:
C -0.822646  1.938438  0.00000
C  0.503366  1.450934  0.00000
C -1.817254  0.935460 -0.00000
```

```
C -1.500426   -0.412723    0.00000
C  0.789490    0.090212   -0.00000
C -0.198355   -0.887121    0.00000
H  1.355091    2.133834   -0.00000
Cl  2.489972   -0.465378   -0.00000
H  0.021568   -1.946448    0.00000
F -2.505732   -1.350560   -0.00000
H -2.879645    1.187876   -0.00000
```

```
Molecule 128: k2Ar - Intermediate
#n M06L/6-311++G** SCRF=(Solvent=Water) Opt
E(RM06L) =  -1043.17722326
Charge, multiplicity: -1, 1
Geometry:
C  0.709553    0.057867   -0.03815
B  2.280300   -0.391851    0.00281
O  3.194768    0.681315   -0.46359
O  2.383866   -1.610905   -0.83204
H  3.274753   -1.957191   -0.73897
H  3.100099    0.787730   -1.41192
O  2.670003   -0.652591    1.41206
H  3.500697   -0.198628    1.56783
C -0.319643   -0.894993   -0.02408
C  0.340926    1.408816   -0.03057
C -0.995176    1.763616   -0.00821
C -1.649379   -0.498211    0.00072
C -2.026887    0.839899    0.00869
H -0.074868   -1.952065   -0.03904
Cl -2.918435   -1.718011    0.01743
H -3.062716    1.152267    0.02433
F -1.330378    3.082801   -0.00523
H  1.101868    2.183598   -0.04723
```

```
Molecule 129: k2Ar - Intermediate, BA detached
```

```
#n M06L/6-311++G** SCRF=(Solvent=Water) Opt
E(RM06L) =  -306.952829790
Charge, multiplicity: -1, 1
Geometry:
C  -2.001634    0.040364    0.00001
C  -1.183762    1.193166    0.00002
C   0.218671    1.185541   -0.00003
C   0.894364   -0.030934   -0.00002
O   2.271813   -0.116981    0.00006
C   0.161697   -1.213631   -0.00002
C  -1.238089   -1.150841    0.00000
H  -1.646546    2.185569    0.00022
H   0.790643    2.114699   -0.00023
H   2.630358    0.776900   -0.00021
H   0.693243   -2.164201    0.00015
H  -1.749686   -2.119104   -0.00019
```

```
Molecule 129: k2Ar - Intermediate
#n M06L/6-311++G** SCRF=(Solvent=Water) Opt
E(RM06L) =  -559.550140056
Charge, multiplicity: -1, 1
Geometry:
C   0.354925    0.029761    0.01415
C  -0.414630    1.198029    0.03703
C  -1.811354    1.187748    0.02942
C  -2.487514   -0.027843   -0.00360
C  -1.762060   -1.218849   -0.02469
C  -0.372046   -1.172349   -0.01365
B   1.982962    0.019576    0.02209
O   2.432556   -0.736569   -1.18692
H   3.352896   -0.508071   -1.34083
O   2.568492    1.387484    0.02732
H   2.208327    1.884246   -0.71067
```

```
O  2.485431   -0.645736    1.25706
H  3.080405   -1.345569    0.98493
O -3.856870   -0.115558   -0.01343
H -4.224896    0.774193    0.00312
H -2.298060   -2.163605   -0.04647
H  0.089298    2.163074    0.06881
H -2.376044    2.117517    0.05050
H  0.172470   -2.115607   -0.02394
```

```
Molecule 130: k2Ar - Intermediate, BA detached
#n M06L/6-311++G** SCRF=(Solvent=Water) Opt
E(RM06L) =  -568.826094147
Charge, multiplicity: -1, 1
Geometry:
C -2.165628   -1.372904    0.00000
C -2.835565   -0.124712   -0.00000
C -2.203081    1.122330   -0.00000
C -0.813474    1.199751   -0.00000
C -0.094130    0.006544    0.00000
C  1.392663    0.021354    0.00001
F  1.925693   -0.615624    1.08003
F  1.925695   -0.615358   -1.08019
F  1.930250    1.262455    0.00015
C -0.762597   -1.228641    0.00000
H -3.929955   -0.105906    0.00002
H -2.786908    2.041479   -0.00000
H -0.302836    2.156659   -0.00001
H -0.124158   -2.117817   -0.00001
```

```
Molecule 130: k2Ar - Intermediate
#n M06L/6-311++G** SCRF=(Solvent=Water) Opt
E(RM06L) =  -821.421840092
Charge, multiplicity: -1, 1
Geometry:
```

```
O   3.724663    0.276874   -0.73200
B   2.647669   -0.449186   -0.02322
O   2.457247   -1.762235   -0.66385
O   3.116380   -0.563131    1.39610
C   1.210345    0.322234   -0.06519
C   1.127550    1.721125   -0.04673
C  -0.089381    2.400264   -0.01845
C  -1.282662    1.689323   -0.01118
C  -1.232396    0.294682   -0.03534
C  -2.517055   -0.460331   -0.00049
F  -2.372026   -1.779587   -0.23178
F  -3.413688   -0.006424   -0.91301
F  -3.145731   -0.354728    1.20274
C  -0.007940   -0.371860   -0.05959
H   4.092959    0.933980   -0.13837
H   3.301195   -2.219639   -0.64188
H   2.408701   -0.920610    1.93668
H   2.049290    2.301004   -0.06063
H  -0.112309    3.486072   -0.00610
H  -2.237907    2.204552    0.00803
H   0.005647   -1.457470   -0.08308
```

```
Molecule 131: k2Ar - Intermediate, BA detached
#n M06L/6-311++G** SCRF=(Solvent=Water) Opt
E(RM06L) =  -691.333155468
Charge, multiplicity: -1, 1
Geometry:
C  -0.173755   -1.336671   -0.00000
C  -1.583822   -1.210027    0.00000
C  -2.266925    0.009965   -0.00000
C  -1.549976    1.205177   -0.00000
C  -0.157168    1.158931   -0.00000
C   0.432057   -0.102373   -0.00000
```

```
Cl  2.292948   -0.029774    0.00000
H -2.198744   -2.114904   -0.00000
H -3.355305    0.034544   -0.00000
H -2.059785    2.164233   -0.00000
H  0.431251    2.072277   -0.00000
```

```
Molecule 131: k2Ar - Intermediate
#n M06L/6-311++G** SCRF=(Solvent=Water) Opt
E(RM06L) =  -943.914285578
Charge, multiplicity: -1, 1
Geometry:
O  2.231791   -1.032406   -1.15809
B  1.687098   -0.278136    0.00014
O  2.231667   -1.032238    1.15854
O  2.144305    1.122273    0.00003
C  0.038533   -0.370379    0.00008
C -0.455130   -1.692658    0.00028
C -1.802985   -2.021819    0.00024
C -2.756956   -1.005610   -0.00002
C -2.333566    0.315037   -0.00021
C -0.965067    0.599361   -0.00015
Cl -0.585926    2.337785   -0.00040
H  1.954140   -0.591404   -1.96268
H  1.953113   -0.591730    1.96309
H  3.104544    1.083757    0.00018
H  0.286238   -2.488296    0.00048
H -2.113168   -3.062771    0.00040
H -3.818279   -1.233182   -0.00006
H -3.052414    1.127347   -0.00041
```

```
Molecule 133: k2Ar - Intermediate, BA detached
#n M06L/6-311++G** SCRF=(Solvent=Water) Opt
E(RM06L) =  -310.352716042
Charge, multiplicity: -1, 1
```

```
Geometry:
C  2.515794   -0.978839    0.00010
C  1.212605   -0.229723   -0.00011
C  0.000013   -0.924073   -0.00003
C -1.212598   -0.229714    0.00007
C -2.515787   -0.978857   -0.00005
C -1.176766    1.174032    0.00014
C -0.000002    1.956574   -0.00001
C  1.176744    1.174049   -0.00014
H  3.367732   -0.293830   -0.00226
H  2.614714   -1.630291   -0.87510
H  2.616585   -1.626361    0.87803
H -0.000008   -2.014921   -0.00024
H -3.367719   -0.293843   -0.00074
H -2.615352   -1.628788   -0.87632
H -2.615965   -1.627830    0.87684
H -2.156866    1.667594    0.00030
H  2.156861    1.667579   -0.00027
```

```
Molecule 133: k2Ar - Intermediate
#n M06L/6-311++G** SCRF=(Solvent=Water) Opt
E(RM06L) =  -562.953063593
Charge, multiplicity: -1, 1
Geometry:
C  2.340433   -2.552541   -0.01047
C  1.624951   -1.232559   -0.00202
C  2.348004   -0.035681   -0.00471
C  1.696858    1.199301   -0.00352
C  2.488386    2.475460   -0.00677
C  0.297208    1.216639   -0.00055
C -0.476916    0.048744    0.00337
B -2.106164    0.050976   -0.00799
O -2.673624   -0.587451   -1.22836
```

```
O -2.646525 -0.751472 1.13812
O -2.572064 1.450254 0.02076
C 0.229980 -1.164296 0.00445
H 1.642101 -3.387345 0.07964
H 2.908802 -2.697545 -0.93515
H 3.060333 -2.629098 0.81021
H 3.437166 -0.068294 -0.00549
H 3.171508 2.531092 0.84667
H 3.108092 2.564843 -0.90494
H 1.836430 3.350689 0.03233
H -0.208938 2.180934 0.00159
H -2.546413 -1.536184 -1.16080
H -2.173977 -0.512839 1.93822
H -3.531592 1.430130 -0.00019
H -0.328420 -2.102303 0.01510
```

```
Molecule 135: k2Ar - Intermediate, BA detached
#n M06L/6-311++G** SCRF=(Solvent=Water) Opt
E(RM06L) = -436.279232164
Charge, multiplicity: -1, 1
Geometry:
C -1.872990 -1.182478 -0.00000
C -2.660484 -0.000050 0.00000
C -1.873145 1.182433 0.00000
C -0.483215 1.213396 0.00000
C 0.208254 0.000049 -0.00000
C -0.483085 -1.213338 -0.00000
H 0.076188 -2.143478 -0.00000
H -2.369569 -2.156067 -0.00000
H -2.369816 2.155979 0.00000
H 0.075867 2.143619 0.00000
N 1.654734 0.000024 -0.00000
O 2.249640 1.083577 -0.00000
```

```
O  2.249383   -1.083613    0.00001
```

```
Molecule 135: k2Ar - Intermediate
#n M06L/6-311++G** SCRF=(Solvent=Water) Opt
E(RM06L) =  -688.871230971
Charge, multiplicity: -1, 1
Geometry:
C -0.287750    1.210918   -0.02651
C -1.030028    0.018050   -0.02328
C -0.295482   -1.183218   -0.00618
C  1.088637   -1.208664    0.00178
C  1.776771    0.005586   -0.00541
C  1.099747    1.223053   -0.01910
H  1.663957    2.147895   -0.02517
H -0.824096    2.155369   -0.03841
H -0.833773   -2.127801   -0.00236
H  1.644771   -2.138167    0.01218
N  3.230676   -0.000747   -0.00008
B -2.657509   -0.014141   -0.00941
O -3.124518   -0.955884   -1.05473
O -3.152347   -0.549330    1.29198
H -2.812392   -0.005999    2.00687
H -3.844169   -0.536414   -1.52805
O -3.157513    1.359386   -0.24233
H -4.051233    1.410957    0.10342
O  3.813036   -1.086920    0.01609
O  3.823641    1.079717   -0.01205
```

```
Molecule 136: k2Ar - Intermediate, BA detached
#n M06L/6-311++G** SCRF=(Solvent=Water) Opt
E(RM06L) =  -420.275962086
Charge, multiplicity: -1, 1
Geometry:
C -2.274104    0.814379    0.03795
```

```
C -2.221036   -0.597461    0.03385
C -1.045280   -1.380143   -0.00231
C  0.144406   -0.686913   -0.02745
C  0.160801    0.697209   -0.02731
C -1.001095    1.448204   -0.00133
O  1.483322    1.125709   -0.11940
C  2.249916   -0.036027    0.17113
O  1.439843   -1.170752   -0.12285
H -3.155039   -1.165560    0.06539
H -1.065562   -2.467623   -0.01209
H -0.905072    2.536532   -0.01282
H  3.145818   -0.054520   -0.45154
H  2.512888   -0.043972    1.24198
```

```
Molecule 136: k2Ar - Intermediate
#n M06L/6-311++G** SCRF=(Solvent=Water) Opt
E(RM06L) =  -672.871948405
Charge, multiplicity: -1, 1
Geometry:
O  3.226264    1.115111   -0.27353
B  2.481025   -0.127634   -0.01846
O  2.844156   -1.134113   -1.06851
O  2.889353   -0.684768    1.31650
C  0.886793    0.197053   -0.02914
C  0.377796    1.501037   -0.01022
C -0.996717    1.797769    0.01882
C -1.857681    0.723779    0.02891
C -1.385676   -0.580053    0.01321
C -0.039755   -0.870338   -0.01583
O -2.454801   -1.449191    0.07751
C -3.597836   -0.634912   -0.17428
O -3.233923    0.714968    0.10682
H  4.132603    0.868958   -0.46955
```

```
H  3.094035   -1.940425   -0.61463
H  2.160056   -1.173263    1.70233
H  1.082871    2.327536   -0.02904
H -1.365771    2.817955    0.03463
H  0.300660   -1.903225   -0.03757
H -4.416056   -0.937789    0.48061
H -3.883463   -0.723647   -1.23363
```

```
Molecule 137: k2Ar - Intermediate, BA detached
#n M06L/6-311++G** SCRF=(Solvent=Water) Opt
E(RM06L) =  -460.792303445
Charge, multiplicity: -1, 1
Geometry:
C -2.656619   -1.451347    0.00048
O -1.245509   -1.387012   -0.00060
C -0.689967   -0.128224   -0.00023
C -1.405167    1.062614   -0.00020
C -0.723039    2.292488   -0.00000
C  0.677209    2.429669    0.00013
C  1.350171    1.179938    0.00011
C  0.715526   -0.064848    0.00002
O  1.354054   -1.285815    0.00018
C  2.766635   -1.268546   -0.00002
H -3.085994   -0.975043   -0.88913
H -3.084562   -0.975793    0.89117
H -2.916500   -2.509211    0.00009
H -2.491935    1.037875   -0.00048
H -1.360516    3.182125    0.00008
H  2.440965    1.162696    0.00025
H  3.168317   -0.769786   -0.88981
H  3.168482   -0.770008    0.88982
H  3.084888   -2.310691   -0.00027
```

```
Molecule 137: k2Ar - Intermediate
```

```
#n M06L/6-311++G** SCRF=(Solvent=Water) Opt
E(RM06L) =  -713.391963612
Charge, multiplicity: -1, 1
Geometry:
C  0.984422   -1.655632   -0.03707
C -0.410854   -1.546197   -0.07274
C -1.061379   -0.312192   -0.07836
C -0.238333    0.828852   -0.05020
C  1.149310    0.749249   -0.01661
C  1.775372   -0.513924   -0.00791
B -2.676065   -0.171694   -0.02296
O -3.083576    1.103270   -0.65382
H -3.948378    1.336629   -0.30902
O -3.193925   -0.093633    1.38588
H -3.038803   -0.938692    1.81333
O -3.254676   -1.369689   -0.69627
H -4.207344   -1.341498   -0.57859
O  1.996545    1.824047    0.00788
C  1.399312    3.107489    0.00270
O  3.143083   -0.506047    0.02689
C  3.784584   -1.767330    0.04057
H  4.854068   -1.565821    0.07267
H  3.553286   -2.347622   -0.85990
H  3.500806   -2.354545    0.92136
H  2.218535    3.824370    0.02808
H  0.758869    3.258155    0.87884
H  0.802871    3.271530   -0.90164
H -1.002513   -2.457998   -0.10041
H  1.451019   -2.634662   -0.03451
H -0.716317    1.803153   -0.06207
```

```
Molecule 138: k2Ar - Intermediate, BA detached
#n M06L/6-311++G** SCRF=(Solvent=Water) Opt
```

```
E(RM06L) =  -420.340694216
Charge, multiplicity: -1, 1
Geometry:
O -2.297210    1.155496   -0.00001
C -1.645979    0.123896   -0.00000
O -2.261046   -1.087843    0.00003
C -0.185244    0.032693   -0.00002
C  0.571857    1.212968    0.00001
C  1.961992    1.150358   -0.00001
C  2.715373   -0.052350    0.00002
C  1.892111   -1.207349    0.00002
C  0.499344   -1.191701   -0.00005
H -3.212419   -0.913209    0.00008
H  0.045584    2.166012    0.00011
H  2.486323    2.110696   -0.00005
H  2.358894   -2.196984    0.00009
H -0.069052   -2.118819   -0.00017
```

```
Molecule 138: k2Ar - Intermediate
#n M06L/6-311++G** SCRF=(Solvent=Water) Opt
E(RM06L) =  -672.930508306
Charge, multiplicity: -1, 1
Geometry:
C  1.060080    0.017023   -0.02636
C  0.312421   -1.168596    0.07392
C -1.074549   -1.171941    0.08756
C -1.786930    0.033427    0.00496
C -1.062750    1.228410   -0.07973
C  0.325024    1.210846   -0.09920
B  2.687059   -0.024680   -0.01150
O  3.199235    1.288504   -0.46966
H  4.086949    1.393012   -0.12008
O  3.185571   -0.337371    1.36124
```

```
H  2.817191    0.296268    1.98135
O  3.159036   -1.127257   -0.88460
H  3.861228   -0.780840   -1.43639
C -3.261383    0.111773    0.01126
H -1.612883    2.162598   -0.14037
H  0.838234   -2.118174    0.14250
H -1.589674   -2.126036    0.18516
H  0.868225    2.148987   -0.17830
O -3.954785   -1.044836   -0.13101
H -3.352288   -1.784481   -0.28248
O -3.892026    1.141762    0.13299
```

```
Molecule 139: k2Ar - Intermediate, BA detached
#n M06L/6-311++G** SCRF=(Solvent=Water) Opt
E(RM06L) =  -345.069968497
Charge, multiplicity: -1, 1
Geometry:
O -2.904051   -0.395144   -0.00019
C -1.966077    0.389197    0.00023
C -0.530798    0.087135    0.00010
C  0.301257    1.227572    0.00003
C  1.679765    1.088292   -0.00005
C  2.211722   -0.203184   -0.00006
C  1.364379   -1.315763    0.00000
C -0.046083   -1.253091    0.00008
H -2.186648    1.487526   -0.00035
H -0.146375    2.222768    0.00005
H  2.329871    1.958182   -0.00010
H  3.293573   -0.335050   -0.00013
H  1.857003   -2.293222   -0.00001
```

```
Molecule 139: k2Ar - Intermediate
#n M06L/6-311++G** SCRF=(Solvent=Water) Opt
E(RM06L) =  -597.662597034
```

```
Charge, multiplicity: -1, 1
Geometry:
O  1.291043   2.892462   0.02540
C  0.506440   1.952205  -0.00285
C  0.901915   0.536114  -0.00334
C  2.272226   0.230593   0.01719
C  2.700411  -1.085864   0.01432
C  1.746094  -2.104194  -0.01469
C  0.388831  -1.794630  -0.03910
C -0.088209  -0.475984  -0.03000
B -1.699361  -0.157856  -0.02734
O -2.511102  -1.401791  -0.00780
O -2.010122   0.572184  -1.26400
O -2.060059   0.705039   1.14739
H -0.583065   2.130145  -0.03500
H  2.986589   1.049285   0.03400
H  3.759443  -1.323707   0.03043
H  2.066226  -3.142942  -0.02233
H -0.330862  -2.609416  -0.07505
H -2.283293  -1.948545   0.74707
H -2.946849   0.785944  -1.24758
H -2.415714   0.155924   1.84811
```

```
Molecule 140: k2Ar - Intermediate, BA detached
#n M06L/6-311++G** SCRF=(Solvent=Water) Opt
E(RM06L) =  -309.121726521
Charge, multiplicity: -1, 1
Geometry:
C  2.929918   0.316206  -0.00000
C  1.895689  -0.536362  -0.00000
C  0.470548  -0.219788   0.00000
C -0.469994  -1.260906   0.00000
C -1.840515  -0.993322  -0.00000
```

```
C -2.404505    0.304428    0.00000
C -1.411562    1.318112   -0.00000
C -0.036223    1.089954    0.00000
H  3.952802   -0.042556    0.00000
H  2.795289    1.394019   -0.00000
H  2.119327   -1.604873    0.00000
H -0.104524   -2.289555    0.00000
H -2.500258   -1.867263   -0.00000
H -1.719893    2.368997    0.00000
H  0.657120    1.931302    0.00000
```

```
Molecule 140: k2Ar - Intermediate
#n M06L/6-311++G** SCRF=(Solvent=Water) Opt
E(RM06L) =  -561.717951457
Charge, multiplicity: -1, 1
Geometry:
C  4.496809   -0.615117   -0.00344
C  3.586585    0.366764    0.00152
C  2.131610    0.234015    0.00204
C  1.469981   -1.006666    0.00546
C  0.085077   -1.070549    0.00269
C -0.731193    0.076910   -0.00322
B -2.351838   -0.060048   -0.00845
O -2.774552   -0.869263    1.18358
O -2.954992    1.285066    0.01078
O -2.828201   -0.776641   -1.23023
C -0.055818    1.303567   -0.00594
C  1.334913    1.386534   -0.00268
H  4.221794   -1.665712   -0.00785
H  5.558066   -0.395074   -0.00358
H  3.946366    1.396478    0.00464
H  2.051960   -1.925257    0.00911
H -0.389541   -2.051487    0.00435
```

```
H -3.385265   -0.333614    1.69196
H -3.835706    1.204258   -0.36183
H -3.037830   -1.674882   -0.96824
H -0.640879    2.220294   -0.01246
H  1.824401    2.359184   -0.00542
```

```
Molecule 142: k2Ar - Intermediate, BA detached
#n M06L/6-311++G** SCRF=(Solvent=Water) Opt
E(RM06L) =  -345.068976422
Charge, multiplicity: -1, 1
Geometry:
C -1.784468    1.224012    0.00000
C -2.233788   -0.117172    0.00000
C -1.403279   -1.252921   -0.00000
C -0.027576   -1.103976   -0.00000
C  0.498957    0.197167   -0.00000
C -0.374943    1.301780    0.00000
H  0.643763   -1.958258   -0.00000
H -3.309830   -0.319467    0.00000
H -1.839137   -2.251031    0.00000
C  1.935630    0.432584   -0.00000
O  2.813761   -0.419389    0.00000
H  2.212859    1.513686   -0.00000
H  0.119049    2.281337    0.00000
```

```
Molecule 142: k2Ar - Intermediate
#n M06L/6-311++G** SCRF=(Solvent=Water) Opt
E(RM06L) =  -597.663631568
Charge, multiplicity: -1, 1
Geometry:
C -0.610940    0.228705   -0.02853
C -0.306580    1.595923   -0.03973
C  1.005674    2.082750   -0.03044
C  2.071206    1.199293   -0.01261
```

```
C  1.810777  -0.178994  -0.00314
C  0.487438  -0.638386  -0.00887
B -2.142386  -0.321131  -0.00579
O -2.194375  -1.570709  -0.79169
O -3.121690   0.612064  -0.62803
H -3.112938  -1.742225  -1.01121
H -3.320948   1.314058  -0.00543
O -2.478032  -0.519208   1.43730
H -3.384670  -0.832765   1.49671
H  3.099233   1.548311  -0.00830
H -1.125415   2.313696  -0.06572
H  1.187937   3.153686  -0.04019
C  2.897619  -1.156080   0.01281
O  4.090027  -0.899301   0.02158
H  2.552864  -2.216065   0.01714
H  0.317262  -1.715086  -0.00410
```

```
Molecule 148: k2Ar - Intermediate, BA detached
#n M06L/6-311++G** SCRF=(Solvent=Water) Opt
E(RM06L) =  -1150.95198731
Charge, multiplicity: -1, 1
Geometry:
C -0.000004   0.831505   0.00000
C  1.129113   0.044694   0.00000
Cl  2.771547   0.875232  -0.00000
C  1.204659  -1.345408   0.00000
C  0.000008  -2.044530   0.00000
C -1.204647  -1.345419   0.00000
C -1.129123   0.044692  -0.00000
Cl -2.771547   0.875230   0.00000
H  2.154169  -1.870591  -0.00000
H -0.000024  -3.129911   0.00000
H -2.154177  -1.870569   0.00000
```

```
Molecule 148: k2Ar - Intermediate
#n M06L/6-311++G** SCRF=(Solvent=Water) Opt
E(RM06L) =  -1403.51970419
Charge, multiplicity: -1, 1
Geometry:
O -1.017480   -1.913753    1.28716
B -0.386526   -1.610082   -0.01985
O -1.265584   -1.862090   -1.19084
O  0.722147   -2.536010   -0.17953
C  0.061712    0.019375   -0.01112
C -0.905255    1.037618   -0.01543
Cl -2.639730    0.611490    0.08726
C -0.671210    2.405303   -0.06554
C  0.643616    2.847292   -0.10345
C  1.667892    1.915188   -0.06411
C  1.359470    0.556043   -0.00919
Cl  2.792485   -0.480219    0.10638
H -1.674953   -1.249546    1.50583
H -2.076803   -1.354595   -1.13360
H  0.566230   -3.008477   -0.99833
H -1.499665    3.103543   -0.07035
H  0.866089    3.907612   -0.14886
H  2.704865    2.230196   -0.06870
```

```
Molecule 149: k2Ar - Intermediate, BA detached
#n M06L/6-311++G** SCRF=(Solvent=Water) Opt
E(RM06L) =  -2805.14212367
Charge, multiplicity: -1, 1
Geometry:
C -0.804415   -1.333277    0.00000
C -0.198282   -0.103430    0.00000
Br  1.833842   -0.014250   -0.00000
C -0.785898    1.159052    0.00000
```

```
C -2.179255    1.204081    0.00000
C -2.896701    0.009024    0.00000
C -2.215347   -1.211650    0.00000
H -0.201505    2.075071    0.00000
H -2.688897    2.163364    0.00000
H -3.985116    0.034497    0.00000
H -2.829548   -2.116978    0.00000
```

```
Molecule 149: k2Ar - Intermediate
#n M06L/6-311++G** SCRF=(Solvent=Water) Opt
E(RM06L) =  -3057.72499435
Charge, multiplicity: -1, 1
Geometry:
O -1.534442   -1.522305    1.13468
B -1.745748   -0.609071   -0.02916
O -3.138536   -0.077883   -0.00487
O -1.576876   -1.368812   -1.26911
C -0.647851    0.619878   -0.02831
C  0.743671    0.477255    0.00014
Br  1.569996   -1.271937    0.00520
C  1.640849    1.543721    0.02047
C  1.151379    2.845733    0.01447
C -0.223174    3.051748   -0.01797
C -1.083229    1.957430   -0.04178
H -2.064679   -1.219113    1.87363
H -3.285274    0.468760    0.77006
H -2.135955   -2.147357   -1.20827
H  2.709758    1.359270    0.04052
H  1.842962    3.682231    0.03206
H -0.623808    4.061338   -0.02917
H -2.155163    2.135444   -0.08290
```

```
Molecule 150: k2Ar - Intermediate, BA detached
#n M06L/6-311++G** SCRF=(Solvent=Water) Opt
```

```
E(RM06L) =  -2805.13463217
Charge, multiplicity: -1, 1
Geometry:
C -2.238033   1.349843   0.00002
C -0.828123   1.257492   0.00001
C -0.166968   0.031090   0.00000
C -0.840482  -1.183575  -0.00000
C -2.234690  -1.137096   0.00001
C -2.890939   0.095938   0.00002
Br  1.785239  -0.014305  -0.00001
H -0.304324  -2.126096  -0.00001
H -2.793048  -2.073447   0.00000
H -3.984597   0.061713   0.00003
H -0.205983   2.156345   0.00001
```

```
Molecule 150: k2Ar - Intermediate
#n M06L/6-311++G** SCRF=(Solvent=Water) Opt
E(RM06L) =  -3057.72704085
Charge, multiplicity: -1, 1
Geometry:
C -1.255152   0.304684  -0.04849
C -0.020623  -0.358750  -0.03006
C  1.171117   0.357090  -0.01144
C  1.196816   1.746572  -0.01316
C -0.023888   2.419224  -0.03442
C -1.219787   1.707356  -0.05118
Br  2.838967  -0.596328   0.00889
H  2.135853   2.288013  -0.00184
H -0.027792   3.505988  -0.04098
H -2.160458   2.254231  -0.07518
H -0.002882  -1.444811  -0.03909
B -2.663757  -0.514750   0.00306
O -3.235397  -0.464552   1.38429
```

```
H -3.264469    0.449299    1.67738
O -3.601168    0.112138   -0.96463
H -4.493698   -0.083821   -0.66953
O -2.441962   -1.944342   -0.30576
H -3.094292   -2.206695   -0.95572
```

6.2.4 k_{2cat} (B3LYP)

```
Molecule 0: k2cat_B3LYP - Misc. small molecule
#n B3LYP/6-31G(d) Opt
E(RB3LYP) =  -216.51957507
Charge, multiplicity: 0, 1
Geometry:
C  1.522933    0.025843   -0.00006
B -0.051728    0.004250   -0.00040
H  1.918630   -0.508459   -0.87295
H  1.916799   -0.494075    0.88241
H  1.923838    1.043453   -0.00756
O -0.681231   -1.219966   -0.00006
H -1.648341   -1.174392    0.00074
O -0.730603    1.200368   -0.00001
H -1.695214    1.113949    0.00039
```

```
Molecule 134: k2cat_B3LYP - Transition State
#n B3LYP/6-31G(d) Opt=(TS,CalcFC,noeigentest)
E(RB3LYP) =  -778.12409149
Charge, multiplicity: -1, 1
Geometry:
C  0.341477   -0.898259   -0.22314
B -4.101306   -1.465262    0.08847
O -4.877093   -2.624100    0.04322
O -3.256936   -1.301409    1.15089
H -2.804725   -0.424685    1.02085
```

```
H -5.396372   -2.569495   -0.77104
O -4.226201   -0.561703   -0.93605
H -3.593711    0.192781   -0.74381
O -2.406806    1.010112    0.13553
B -1.797036    2.178008    0.11710
O -0.797736    2.513450    1.07618
C -2.036033    3.334018   -0.97875
H -0.751199    1.739505    1.66050
H -3.075215    3.345461   -1.33101
H -0.697703   -0.580382   -0.17361
H -1.782226    4.337064   -0.60996
H -1.407664    3.153618   -1.86448
C  1.325013   -0.012393   -0.01200
C  2.775911   -0.262279   -0.04140
C  3.657300    0.811678    0.18168
C  5.040463    0.634500    0.16426
C  5.583573   -0.628099   -0.07725
C  4.723721   -1.708489   -0.30001
C  3.343518   -1.528790   -0.28205
H  3.239804    1.798065    0.37029
H  5.694788    1.485640    0.34008
H  6.661501   -0.771347   -0.09141
H  5.133534   -2.698706   -0.48808
H  2.692615   -2.381193   -0.45491
H  0.533048   -1.946315   -0.44814
H  1.033768    1.014140    0.21079
```

```
Molecule 134: k2cat_B3LYP - Intermediate
#n B3LYP/6-31G(d) Opt
E(RB3LYP) =  -561.52152861
Charge, multiplicity: -1, 1
Geometry:
C  1.184445    0.414887   -0.01083
```

```
B  2.778668   0.050571  -0.00323
O  3.427986   0.756853   1.14087
H  3.778352   0.020928   1.66268
O  2.984199  -1.416438   0.14314
H  3.501260  -1.630248  -0.64620
C  0.160544  -0.462829  -0.00906
C -1.281664  -0.185593  -0.00427
C -2.200598  -1.257528  -0.00131
C -3.579094  -1.050445   0.00253
C -4.099140   0.245804   0.00338
C -3.208520   1.325660   0.00035
C -1.832961   1.115522  -0.00355
H -1.808158  -2.272956  -0.00162
H -4.252005  -1.907047   0.00494
H -5.174230   0.413395   0.00640
H -3.593675   2.344584   0.00095
H -1.160142   1.968585  -0.00588
H  0.421582  -1.523377  -0.00837
O  3.395340   0.525973  -1.27227
H  4.145113   1.045391  -0.94586
H  0.930290   1.483929  -0.00821
```

```
Molecule 141: k2cat_B3LYP - Transition State
#n B3LYP/6-31G(d) Opt=(TS,CalcFC,noeigentest)
E(RB3LYP) =  -698.42786429
Charge, multiplicity: -1, 1
Geometry:
C -1.594239  -1.021280   1.09230
C -2.763828  -1.656604   0.80649
O -3.045967  -1.554032  -0.52602
C -1.982915  -0.819145  -1.05128
H -1.147045  -0.968581   2.08188
H -3.490181  -2.207261   1.39122
```

```
H -2.068343   -0.668614   -2.12124
C -1.046925   -0.443808   -0.13007
B -0.304919    1.822272   -0.07304
O  0.405585    1.914454   -1.25834
O -1.565330    2.422005    0.01114
H -2.027037    2.232227   -0.81682
O  0.389318    1.753915    1.16358
H  0.604406   -0.528267   -0.56249
O  1.583594   -0.670674   -0.92163
H -0.282872    1.620007    1.84757
H  1.055357    1.189211   -1.31194
B  2.583080   -0.683798    0.01051
O  2.493221   -0.078129    1.23831
H  1.689026    0.487462    1.29132
C  3.947044   -1.434325   -0.34803
H  4.736160   -1.259947    0.39279
H  4.319344   -1.118940   -1.33227
H  3.782180   -2.518999   -0.42023
```

```
Molecule 141: k2cat_B3LYP - Intermediate
#n B3LYP/6-31G(d) Opt
E(RB3LYP) =  -481.89317689
Charge, multiplicity: -1, 1
Geometry:
C  0.334322    0.010372    0.00516
B -1.303152    0.008303    0.00398
O -1.833065    0.823346   -1.13010
H -2.285435    0.155338   -1.66449
O -1.788048   -1.398992   -0.13822
H -2.370415   -1.485635    0.62992
O -1.813657    0.595113    1.27216
H -2.491421    1.205309    0.94552
C  1.170057   -1.063966    0.00630
```

```
O  2.512508   -0.711050    0.00067
C  2.517853    0.657087   -0.00340
C  1.244841    1.137184   -0.00155
H  0.983099   -2.127949    0.00766
H  3.497584    1.117626   -0.00799
H  0.958009    2.182386   -0.00561
```

6.2.5 k_{2cat} (M06L)

```
Molecule 0: k2cat_M06L - Misc. small molecule
#n M06L/6-311++G**
E(RM06L) =  -216.566280085
Charge, multiplicity: 0, 1
Geometry:
C -1.522933   -0.025843   -0.00006
B  0.051728   -0.004250   -0.00040
H -1.918630    0.508459   -0.87295
H -1.916799    0.494075    0.88240
H -1.923838   -1.043453   -0.00756
O  0.681231    1.219966   -0.00006
H  1.648341    1.174392    0.00073
O  0.730603   -1.200368   -0.00001
H  1.695214   -1.113949    0.00038
```

```
Molecule 134: k2cat_M06L - Transition State
#n M06L/6-311++G**
E(RM06L) =  -778.263353150
Charge, multiplicity: -1, 1
Geometry:
C  0.341477   -0.898259   -0.22314
B -4.101306   -1.465262    0.08846
O -4.877093   -2.624100    0.04321
O -3.256936   -1.301409    1.15088
```

```
H -2.804725   -0.424685    1.02084
H -5.396372   -2.569495   -0.77104
O -4.226201   -0.561703   -0.93605
H -3.593711    0.192781   -0.74381
O -2.406806    1.010112    0.13552
B -1.797036    2.178008    0.11709
O -0.797736    2.513450    1.07617
C -2.036033    3.334018   -0.97875
H -0.751199    1.739505    1.66049
H -3.075215    3.345461   -1.33101
H -0.697703   -0.580382   -0.17361
H -1.782226    4.337064   -0.60996
H -1.407664    3.153618   -1.86448
C  1.325013   -0.012393   -0.01200
C  2.775911   -0.262279   -0.04140
C  3.657300    0.811678    0.18167
C  5.040463    0.634500    0.16425
C  5.583573   -0.628099   -0.07725
C  4.723721   -1.708489   -0.30001
C  3.343518   -1.528790   -0.28205
H  3.239804    1.798065    0.37028
H  5.694788    1.485640    0.34007
H  6.661501   -0.771347   -0.09141
H  5.133534   -2.698706   -0.48808
H  2.692615   -2.381193   -0.45491
H  0.533048   -1.946315   -0.44814
H  1.033768    1.014140    0.21078
```

```
Molecule 134: k2cat_M06L - Intermediate
#n M06L/6-311++G**
E(RM06L) =  -561.631226440
Charge, multiplicity: -1, 1
Geometry:
```

```
C  1.184445  -0.414887   0.01083
B  2.778668  -0.050571   0.00323
O  3.427986  -0.756853  -1.14087
H  3.778352  -0.020928  -1.66268
O  2.984199   1.416438  -0.14314
H  3.501260   1.630248   0.64620
C  0.160544   0.462829   0.00906
C -1.281664   0.185593   0.00427
C -2.200598   1.257528   0.00131
C -3.579094   1.050445  -0.00253
C -4.099140  -0.245804  -0.00338
C -3.208520  -1.325660  -0.00035
C -1.832961  -1.115522   0.00355
H -1.808158   2.272956   0.00162
H -4.252005   1.907047  -0.00494
H -5.174230  -0.413395  -0.00640
H -3.593675  -2.344584  -0.00095
H -1.160142  -1.968585   0.00588
H  0.421582   1.523377   0.00837
O  3.395340  -0.525973   1.27227
H  4.145113  -1.045391   0.94586
H  0.930290  -1.483929   0.00821
```

```
Molecule 141: k2cat_M06L - Transition State
#n M06L/6-311++G**
E(RM06L) =  -698.577253613
Charge, multiplicity: -1, 1
Geometry:
C -1.594239  -1.021280   1.09230
C -2.763828  -1.656604   0.80649
O -3.045967  -1.554032  -0.52601
C -1.982915  -0.819145  -1.05127
H -1.147045  -0.968581   2.08188
```

```
H -3.490181   -2.207261    1.39122
H -2.068343   -0.668614   -2.12123
C -1.046925   -0.443808   -0.13006
B -0.304919    1.822272   -0.07303
O  0.405585    1.914454   -1.25833
O -1.565330    2.422005    0.01114
H -2.027037    2.232227   -0.81681
O  0.389318    1.753915    1.16358
H  0.604406   -0.528267   -0.56248
O  1.583594   -0.670674   -0.92162
H -0.282872    1.620007    1.84757
H  1.055357    1.189211   -1.31193
B  2.583080   -0.683798    0.01051
O  2.493221   -0.078129    1.23831
H  1.689026    0.487462    1.29132
C  3.947044   -1.434325   -0.34802
H  4.736160   -1.259947    0.39279
H  4.319344   -1.118940   -1.33226
H  3.782180   -2.518999   -0.42022
```

```
Molecule 141: k2cat_M06L - Intermediate
#n M06L/6-311++G**
E(RM06L) =  -482.003557808
Charge, multiplicity: -1, 1
Geometry:
C -0.334322   -0.010372    0.00515
B  1.303152   -0.008303    0.00397
O  1.833065   -0.823346   -1.13010
H  2.285435   -0.155338   -1.66449
O  1.788048    1.398992   -0.13822
H  2.370415    1.485635    0.62991
O  1.813657   -0.595113    1.27215
H  2.491421   -1.205309    0.94551
```

```
C -1.170057    1.063966    0.00629
O -2.512508    0.711050    0.00066
C -2.517853   -0.657087   -0.00340
C -1.244841   -1.137184   -0.00155
H -0.983099    2.127949    0.00765
H -3.497584   -1.117626   -0.00799
H -0.958009   -2.182386   -0.00561
```

6.2.6 k_3

The k_3 mechanism is not relevant to any of the novel molecules.

6.2.7 k_4

```
Molecule 0: k4 - Misc. small molecule
#n M06L/6-311++G** SCRF=(Solvent=Water) Opt
E(RM06L) =  -76.4487444811
Charge, multiplicity: 0, 1
Geometry:
O  0.000000    0.000000    0.11889
H -0.000000   -0.755103   -0.47558
H  0.000000    0.755103   -0.47558
```

```
Molecule 106: k4 - Transition State
#n M06L/6-311++G** SCRF=(Solvent=Water) Opt=(TS,CalcFC,noeigentest)
E(RM06L) =  -540.142471842
Charge, multiplicity: 0, 1
Geometry:
C -0.008556   -0.170992   -0.01052
B  1.643651   -0.142354   -0.01168
O  2.100189    1.244838   -0.06416
O  2.159140   -0.883794   -1.18101
H  2.303176   -1.798496   -0.93036
H  3.058086    1.224726   -0.12917
```

```
O  2.129581  -0.886585   1.17954
H  1.825180  -0.455035   1.98063
C -0.606870  -1.436510  -0.00925
C -0.888346   0.919963  -0.00742
N -2.228960   0.685773  -0.00613
C -1.984812  -1.637590  -0.00664
C -2.800834  -0.532981  -0.00582
H  0.044933  -2.306960  -0.00613
H -2.421915  -2.627574  -0.00470
H -3.881364  -0.560306  -0.00397
C -0.493550   2.354436  -0.00429
H  0.112162   2.583023  -0.88156
H  0.138796   2.572528   0.85655
H -1.362681   3.014221   0.01385
H -2.845369   1.491597  -0.00499
```

```
Molecule 106: k4 - Reactant
#n M06L/6-311++G** SCRF=(Solvent=Water) Opt
E(RM06L) =  -463.699062506
Charge, multiplicity: 0, 1
Geometry:
C  0.626115   0.934354   0.00048
C -0.150609  -0.248021  -0.00002
C  0.549792  -1.460537  -0.00049
C  1.936762  -1.481964  -0.00059
C  2.596940  -0.262376  -0.00012
N  1.971136   0.918729   0.00043
C  0.013564   2.301235   0.00055
H  0.800059   3.055426   0.00080
H -0.626137   2.453588   0.87273
H -0.625859   2.453850  -0.87176
B -1.714069  -0.305237  -0.00013
O -2.271543  -1.558562   0.00148
```

```
H -3.232318   -1.574141    0.00120
O -2.446946    0.849015   -0.00163
H -3.398950    0.715328   -0.00095
H  2.492379   -2.412982   -0.00099
H  3.685149   -0.224422   -0.00012
H -0.009400   -2.391334   -0.00080
```

```
Molecule 125: k4 - Transition State
#n M06L/6-311++G** SCRF=(Solvent=Water) Opt=(TS,CalcFC,noeigentest)
E(RM06L) =  -614.174969839
Charge, multiplicity: 0, 1
Geometry:
N  0.183117   -1.668830    0.00586
C -0.116606   -0.354281   -0.01236
C  0.978899    0.520362   -0.01584
C  2.281241    0.022946   -0.02094
C  2.510446   -1.348947   -0.00183
C  1.423005   -2.194172    0.01894
B -1.736568   -0.013679    0.02117
O -2.005437    0.540027    1.35181
O -2.444181   -1.311036   -0.10916
H -2.517554    1.345801    1.26612
H -2.627191   -1.485919   -1.03513
O -1.999320    0.896998   -1.08850
H -2.946398    1.000372   -1.21115
C  0.738450    1.981068    0.00271
O  1.631331    2.802649   -0.03798
H -0.326147    2.275275    0.05933
H  1.496036   -3.273283    0.04337
H  3.110046    0.724304   -0.03340
H  3.510678   -1.760127    0.00117
H -0.650197   -2.267175    0.00943
```

```
Molecule 125: k4 - Reactant
```

```
#n M06L/6-311++G** SCRF=(Solvent=Water) Opt
E(RM06L) =  -537.724185659
Charge, multiplicity: 0, 1
Geometry:
N   0.285861   -1.690551    0.00037
C   0.374903   -0.338896    0.00005
C  -0.786818    0.455716   -0.00026
C  -2.039351   -0.169386   -0.00047
C  -2.110527   -1.548233   -0.00038
C  -0.914890   -2.265244    0.00019
B   1.891178    0.120633   -0.00002
O   2.262549    1.430188   -0.00057
H   3.219685    1.535628   -0.00072
O   2.829127   -0.863291    0.00033
H   2.358026   -1.713558    0.00077
H  -0.927526   -3.352917    0.00040
H  -2.932373    0.447412   -0.00073
H  -3.061475   -2.068003   -0.00066
C  -0.735088    1.933468   -0.00006
O  -1.731208    2.632676    0.00086
H   0.273633    2.381000   -0.00080
```

```
Molecule 132: k4 - Transition State
#n M06L/6-311++G** SCRF=(Solvent=Water) Opt=(TS,CalcFC,noeigentest)
E(RM06L) =  -807.392196026
Charge, multiplicity: 0, 1
Geometry:
N   2.768752    0.047668    0.32914
C   1.499479    0.162823   -0.12773
C   0.873595   -1.036089   -0.50174
C   1.574701   -2.234940   -0.39613
C   2.885407   -2.272511    0.07423
C   3.477073   -1.089285    0.44659
```

```
C -0.544914 -1.033695 -0.98344
C -1.531075 -0.746477 0.12318
H 4.483014 -1.003429 0.83374
H 1.087943 -3.161541 -0.68687
H 3.433443 -3.201232 0.15645
B 0.999190 1.736561 -0.13297
H -0.802314 -2.009033 -1.40891
H -0.698139 -0.292331 -1.76870
O -2.741951 -0.487656 -0.38464
C -3.778795 -0.187773 0.57988
C -5.047043 0.074561 -0.18140
H -3.457836 0.679011 1.16580
H -3.872405 -1.034323 1.26608
H -4.932267 0.919848 -0.86269
H -5.853523 0.309335 0.51544
H -5.347403 -0.798934 -0.76343
O -1.289161 -0.786831 1.30932
O 1.335097 2.328532 1.18165
O -0.427377 1.760551 -0.43661
H -0.732789 2.670567 -0.43709
H 0.707729 2.026765 1.84312
O 1.862287 2.414862 -1.11112
H 2.053175 3.296955 -0.78385
H 3.192427 0.926531 0.62238
```

```
Molecule 132: k4 - Reactant
#n M06L/6-311++G** SCRF=(Solvent=Water) Opt
E(RM06L) = -730.942894279
Charge, multiplicity: 0, 1
Geometry:
N -2.915968 0.534059 0.27082
C -1.629358 0.415347 -0.13526
C -1.068543 -0.834511 -0.44996
```

```
C -1.876058   -1.964770   -0.33264
C -3.192825   -1.833661    0.08292
C -3.668192   -0.560114    0.37478
B -0.905725    1.811599   -0.19373
O  0.392061    1.929507   -0.59728
H  0.697849    2.842026   -0.58014
O -1.622307    2.911760    0.16928
H -2.511659    2.604947    0.41110
H -4.694575   -0.414885    0.70446
H -1.469725   -2.944605   -0.56803
H -3.840000   -2.697996    0.18060
C  0.358116   -0.969604   -0.89271
C  1.359267   -0.640750    0.18916
O  2.585033   -0.509464   -0.33285
C  3.639493   -0.184836    0.60574
C  4.921702   -0.077726   -0.16930
H  3.677913   -0.966481    1.36973
H  3.374101    0.752378    1.10439
H  5.165813   -1.020226   -0.66324
H  5.741245    0.171136    0.50710
H  4.861204    0.704176   -0.92877
O  1.113020   -0.531242    1.36964
H  0.575202   -0.331770   -1.75282
H  0.558965   -1.995837   -1.21806
```

```
Molecule 143: k4 - Transition State
#n M06L/6-311++G** SCRF=(Solvent=Water) Opt=(TS,CalcFC,noeigentest)
E(RM06L) =  -807.385161827
Charge, multiplicity: 0, 1
Geometry:
O  0.341125   -2.132648   -0.63397
B  1.596342   -1.683705    0.00562
O  1.485586   -2.202407    1.38216
```

```
O  2.803884  -2.150956  -0.69561
C  1.687868  -0.039884  -0.02535
C  0.664460   0.750857   0.50251
C -0.533404   0.198737   1.20432
C -1.830648   0.546025   0.51107
O -2.717428  -0.438013   0.63701
C -4.017664  -0.227762   0.01753
C -3.974027  -0.586057  -1.44431
O -2.039320   1.599542  -0.05452
N  0.724482   2.100470   0.38912
C  1.740096   2.764630  -0.19404
C  2.791786   2.041423  -0.70512
C  2.749672   0.650726  -0.62172
H  0.380117  -1.928759  -1.57018
H  2.291155  -1.997045   1.85999
H  2.789374  -3.110741  -0.70471
H -0.422959  -0.876377   1.31386
H -0.600291   0.626616   2.21325
H -4.314078   0.810380   0.17902
H -4.685169  -0.879487   0.58117
H -4.970467  -0.483610  -1.87858
H -3.648221  -1.618508  -1.58558
H -3.297365   0.071892  -1.99258
H  1.659510   3.841968  -0.22364
H  3.619414   2.562502  -1.16915
H  3.562300   0.065299  -1.04064
H -0.076006   2.634784   0.71583
```

```
Molecule 143: k4 - Reactant
#n M06L/6-311++G** SCRF=(Solvent=Water) Opt
E(RM06L) =  -730.940836535
Charge, multiplicity: 0, 1
Geometry:
```

```
C -2.987298    0.491529    0.27265
C -1.646126    0.455202   -0.12714
C -1.112761   -0.817400   -0.42046
N -1.824253   -1.951775   -0.33516
C -3.104147   -1.869257    0.04255
C -3.733433   -0.674598    0.35979
B -0.861094    1.806877   -0.21321
O  0.394481    1.830850   -0.75593
H  0.809596    2.697748   -0.77747
O -1.511466    2.913768    0.26107
H -1.017937    3.735314    0.19106
H -4.773778   -0.662157    0.66462
H -3.647507   -2.810903    0.09416
C  0.315221   -1.007867   -0.84709
C  1.319701   -0.625473    0.20936
O  2.551011   -0.557828   -0.31388
C  3.610120   -0.202896    0.60773
C  4.898500   -0.184266   -0.16450
H  3.622591   -0.933189    1.42179
H  3.370947    0.772420    1.04260
H  5.116899   -1.163921   -0.59399
H  5.722143    0.084504    0.49922
H  4.864496    0.547337   -0.97410
O  1.075035   -0.433027    1.38025
H  0.545560   -0.443366   -1.75336
H  0.471446   -2.064246   -1.08470
H -3.440362    1.448546    0.51295
```

```
Molecule 144: k4 - Transition State
#n M06L/6-311++G** SCRF=(Solvent=Water) Opt=(TS,CalcFC,noeigentest)
E(RM06L) =  -595.538227345
Charge, multiplicity: 0, 1
Geometry:
```

```
N  -0.244538   2.282329  -0.00006
C   0.381171   1.095328  -0.00004
C  -0.375326  -0.119563  -0.00008
B  -2.018170  -0.300658   0.00002
O  -2.463812  -1.098017   1.15441
O  -2.610029   1.067572   0.00002
O  -2.464049  -1.098035  -1.15427
C   0.366933  -1.269065  -0.00011
N   1.728114  -1.288407  -0.00009
C   2.468107  -0.148277  -0.00000
C   3.947966  -0.288856   0.00004
C   1.798652   1.046191   0.00001
H  -1.262646   2.281690   0.00005
H  -2.222058  -0.641747   1.96202
H  -3.566712   0.990448   0.00057
H  -2.223464  -0.641182  -1.96190
H  -0.102790  -2.245993  -0.00014
H   2.205878  -2.180104  -0.00008
H   4.288926  -0.840660   0.87994
H   4.289032  -0.840256  -0.88006
H   4.427645   0.687969   0.00029
H   2.374290   1.965260   0.00007
H   0.275814   3.143710   0.00011
```

```
Molecule 144: k4 - Reactant
#n M06L/6-311++G** SCRF=(Solvent=Water) Opt
E(RM06L) =  -519.083732862
Charge, multiplicity: 0, 1
Geometry:
C  -0.108993   1.028584   0.01962
C   0.581375  -0.214816   0.01662
C  -0.221941  -1.357346   0.00914
N  -1.557788  -1.400775  -0.00058
```

```
C -2.192249   -0.208094   -0.00701
C -1.512842    0.999740    0.00168
B  2.123694   -0.373676   -0.00291
O  2.639746   -1.643000    0.02555
H  3.599022   -1.690812   -0.00054
O  2.903451    0.759528   -0.05807
H  3.852420    0.609426   -0.07972
H -2.069233    1.933408   -0.00145
C -3.689609   -0.234861   -0.02421
N  0.546105    2.221848    0.08364
H  1.538552    2.231941   -0.09951
H  0.034384    3.059883   -0.14173
H  0.273452   -2.327857    0.00641
H -4.054678   -1.261399   -0.03902
H -4.085544    0.286980   -0.89987
H -4.105087    0.267824    0.85367
```

```
Molecule 145: k4 - Transition State
#n M06L/6-311++G** SCRF=(Solvent=Water) Opt=(TS,CalcFC,noeigentest)
E(RM06L) =  -595.542076114
Charge, multiplicity: 0, 1
Geometry:
O -2.737553    0.416960   -1.34419
B -2.261435    0.067650    0.00092
O -2.748352    1.109075    0.92060
O -2.694314   -1.303082    0.32342
C -0.621430   -0.029651    0.03987
C  0.272406    1.021131    0.03203
C  1.663153    0.812729    0.00463
N  2.524729    1.849649   -0.00770
C  2.159575   -0.524706   -0.00833
C  3.625814   -0.799597   -0.03264
C  1.238411   -1.537119    0.00666
```

```
N -0.088473   -1.278873    0.02763
H -2.708156    1.370504   -1.44838
H -2.424254    0.946678    1.80885
H -3.612096   -1.412948    0.06794
H  3.521323    1.712747   -0.01810
H  3.827720   -1.870237   -0.03798
H  4.128924   -0.371767    0.84090
H  4.100665   -0.368073   -0.92004
H  1.529287   -2.580680    0.00350
H -0.116646    2.034396    0.04877
H -0.769998   -2.033581    0.05173
H  2.190783    2.798936    0.00657
```

```
Molecule 145: k4 - Reactant
#n M06L/6-311++G** SCRF=(Solvent=Water) Opt
E(RM06L) =  -519.083474493
Charge, multiplicity: 0, 1
Geometry:
N  0.454230   -1.335120    0.00318
C  0.885164   -0.047795   -0.00122
C  0.004187    1.025217   -0.00311
C -1.380410    0.804743   -0.00144
C -1.832548   -0.531269    0.00006
C -0.866398   -1.531785    0.00367
B  2.443225    0.090792    0.00026
O  3.035656    1.319990   -0.00287
H  3.996111    1.263942   -0.00162
O  3.191819   -1.050300    0.00477
H  2.568701   -1.794919    0.00666
H -1.199464   -2.570317    0.00692
C -3.290869   -0.849275   -0.00508
H  0.388972    2.041301   -0.00488
H -3.795397   -0.416134   -0.87569
```

```
H -3.798736   -0.452403    0.88166
H -3.459964   -1.926550   -0.02320
N -2.271051    1.848297   -0.06435
H -1.922392    2.756851    0.20331
H -3.210759    1.675481    0.26118
```

```
Molecule 146: k4 - Transition State
#n M06L/6-311++G** SCRF=(Solvent=Water) Opt=(TS,CalcFC,noeigentest)
E(RM06L) =  -729.916289155
Charge, multiplicity: 0, 1
Geometry:
O -2.951530   -0.189426   -1.14546
B -2.258390   -0.782406    0.00734
O -2.216582   -2.245662   -0.16752
O -2.824994   -0.335287    1.28838
C -0.668429   -0.377607    0.03048
C -0.103426    0.916154    0.01847
O -1.000103    1.921605   -0.00058
C -0.493504    3.252256   -0.02560
C  1.270516    1.091740    0.01310
C  2.118104   -0.026894    0.00798
O  3.438923    0.224470    0.00353
C  4.314126   -0.901511   -0.00796
C  1.563464   -1.294076    0.00127
N  0.212228   -1.382292    0.01448
H -2.996044    0.762109   -1.02094
H -3.098475   -2.608435   -0.25853
H -3.783144   -0.352752    1.22673
H  0.104283    3.461611    0.86627
H -1.363759    3.903544   -0.04127
H  0.110800    3.426562   -0.92082
H  1.726459    2.073959    0.00787
H  4.162165   -1.505494   -0.90714
```

```
H  5.322792   -0.496808   -0.00731
H  4.166884   -1.520239    0.88194
H  2.116787   -2.219778   -0.01353
H -0.233216   -2.302177   -0.00455
```

```
Molecule 146: k4 - Reactant
#n M06L/6-311++G** SCRF=(Solvent=Water) Opt
E(RM06L) =  -653.470397900
Charge, multiplicity: 0, 1
Geometry:
N -0.404861   -1.724385    0.00008
C -0.990889   -0.505347   -0.00018
C -0.194184    0.652756   -0.00061
C  1.197217    0.561871   -0.00060
C  1.759798   -0.711997   -0.00007
C  0.913532   -1.831862    0.00031
B -2.552705   -0.528886   -0.00010
O -3.347983    0.580161   -0.00001
H -2.803992    1.377290   -0.00047
O -3.176990   -1.744673    0.00034
H -2.487609   -2.424513    0.00092
H  1.349514   -2.827727    0.00081
O  3.083551   -0.965040   -0.00000
C  3.968586    0.150768    0.00011
H  4.973812   -0.263405    0.00086
H  3.826312    0.765912    0.89344
H  3.827381    0.765245   -0.89384
O -0.862237    1.835804   -0.00111
C -0.102883    3.042152    0.00113
H -0.827704    3.852392    0.00255
H  0.522409    3.112828   -0.89306
H  0.522287    3.109614    0.89565
H  1.817356    1.447432   -0.00111
```

```
Molecule 147: k4 - Transition State
#n M06L/6-311++G** SCRF=(Solvent=Water) Opt=(TS,CalcFC,noeigentest)
E(RM06L) =  -498.623786788
Charge, multiplicity: 0, 1
Geometry:
O -1.869434   -0.795627    1.08141
B -1.351028    0.007542   -0.01676
O -1.726960    1.404278    0.15212
O -1.737377   -0.434479   -1.35390
C  0.314798   -0.012912    0.01234
N  1.122287    1.037291   -0.03023
C  2.450164    0.640712   -0.02684
C  2.410230   -0.700110    0.02373
O  1.092656   -1.094682    0.04720
H -1.482088   -1.673279    1.10326
H -1.761531    1.614342    1.08768
H -2.173223   -1.286002   -1.29846
H  0.766831    1.985151   -0.06058
H  3.271808    1.333373   -0.05819
H  3.155115   -1.474389    0.05158
```

```
Molecule 147: k4 - Reactant
#n M06L/6-311++G** SCRF=(Solvent=Water) Opt
E(RM06L) =  -422.179294695
Charge, multiplicity: 0, 1
Geometry:
C  0.105209    0.078077    0.00003
B -1.449642    0.002417   -0.00003
O -2.138369   -1.169108   -0.00010
H -1.571587   -1.944957    0.00057
O -2.177077    1.151309    0.00000
H -1.610742    1.929779    0.00007
N  0.847302    1.153037    0.00004
```

```
C   2.147808    0.697369   -0.00004
C   2.139544   -0.658967   -0.00004
O   0.847097   -1.070478    0.00006
H   2.995293    1.364922   -0.00003
H   2.895546   -1.425752   -0.00014
```

6.2.8 k_5

The k_5 mechanism is not relevant to any of the novel molecules.

References

(1) Cox, P. A.; Leach, A. G.; Campbell, A. D.; Lloyd-Jones, G. C. Protodeboronation of Heteroaromatic, Vinyl, and Cyclopropyl Boronic Acids: pH–Rate Profiles, Autocatalysis, and Disproportionation. *Journal of the American Chemical Society* **2016**, *138*, 9145–9157.
